# Discovery of TNG908: A Selective, Brain Penetrant, MTA-Cooperative PRMT5 Inhibitor That Is Synthetically Lethal with *MTAP*-Deleted Cancers

**DOI:** 10.1021/acs.jmedchem.4c00133

**Published:** 2024-04-10

**Authors:** Kevin M. Cottrell, Kimberly J. Briggs, Douglas A. Whittington, Haris Jahic, Janid A. Ali, Charles B. Davis, Shanzhong Gong, Deepali Gotur, Lina Gu, Patrick McCarren, Matthew R. Tonini, Alice Tsai, Erik W. Wilker, Hongling Yuan, Minjie Zhang, Wenhai Zhang, Alan Huang, John P. Maxwell

**Affiliations:** Tango Therapeutics, Boston, Massachusetts 02215, United States

## Abstract

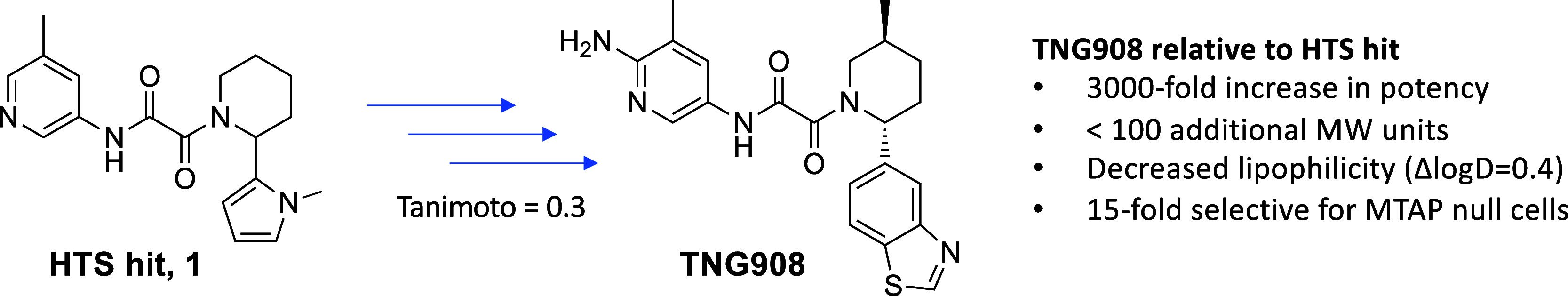

It has been shown that PRMT5 inhibition by small molecules can selectively kill cancer cells with homozygous deletion of the *MTAP* gene if the inhibitors can leverage the consequence of *MTAP* deletion, namely, accumulation of the MTAP substrate MTA. Herein, we describe the discovery of TNG908, a potent inhibitor that binds the PRMT5·MTA complex, leading to 15-fold-selective killing of *MTAP*-deleted (MTAP-null) cells compared to *MTAP*intact (MTAP WT) cells. TNG908 shows selective antitumor activity when dosed orally in mouse xenograft models, and its physicochemical properties are amenable for crossing the blood–brain barrier (BBB), supporting clinical study for the treatment of both CNS and non-CNS tumors with *MTAP* loss.

## Introduction

Large-scale DNA sequencing coupled with functional genomics studies have played a pivotal role in characterizing the cancer genome, revealing the significance of deletion events that promote tumor growth through the loss of tumor suppressor genes. Initiatives like The Cancer Genome Atlas Program (TCGA) have provided a comprehensive map of genetic alterations across human cancers, showing that deletion events often extend beyond the tumor suppressor gene locus, leading to the codeletion of neighboring genes. Although these passenger events may not confer a direct fitness advantage to the tumor, they can create collateral vulnerabilities that can be exploited therapeutically. One example is the collateral vulnerability to PRMT5 inhibition conferred by the loss of *methylthioadenosine phosphorylase* (*MTAP*), a gene which is frequently codeleted with the well-described tumor suppressor gene, *CDKN2A*.^1−3^*MTAP* encodes the protein MTAP, a critical enzyme in the methionine salvage pathway, a process that recycles methionine from a byproduct of polyamine synthesis, methylthioadenosine (MTA).

Loss of *CDKN2A* occurs in 10–15% of all human cancers and with greater frequency in histologies such as malignant peripheral nerve sheath tumors, glioblastoma (GBM), mesothelioma, urothelial carcinoma, esophageal squamous cell carcinoma, pancreatic adenocarcinoma, melanoma, nonsmall cell lung cancer, head and neck cancer, and cholangiocarcinoma.^4−6^ Due to its proximity to *CDKN2A* on chromosome 9p21, *MTAP* is frequently codeleted. Loss of MTAP causes the accumulation of its substrate, MTA, which has been demonstrated by multiple groups to function as an *S*-adenosyl-l-methionine (SAM)-competitive PRMT5 inhibitor.^1−3^

PRMT5 is a type II arginine methyltransferase that regulates essential cellular functions by conferring symmetric dimethylation marks on proteins involved in transcription, splicing, and cellular homeostasis.^7−9^ Due to its implication in the regulation of cell cycle progression, apoptosis, and the DNA-damage response, PRMT5 is considered an essential gene. However, data from genome-wide genetic perturbation screens using shRNA have revealed a selective requirement for PRMT5 activity in *MTAP*-deleted (MTAP-null) cancer cell lines.^1−3^ The accumulation of MTA caused by *MTAP* deletion in these cell lines partially inhibits PRMT5, rendering those cells particularly sensitive to additional PRMT5 inhibition.

**Figure 1:**
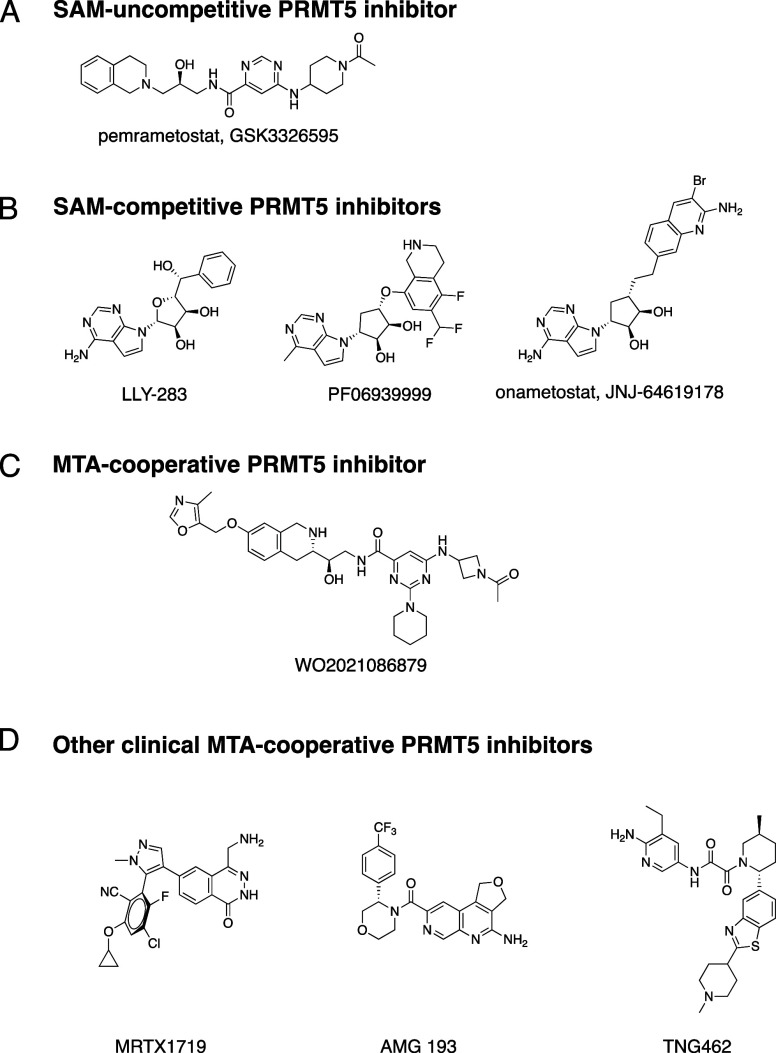
(A) Example of a SAM-uncompetitive, substrate-competitive PRMT5 inhibitor. (B) Examples of SAM-competitive PRMT5 inhibitors. (C) Example compound from the first published disclosure of MTA-cooperative, substrate-competitive PRMT5 inhibitors. (D) Other clinical MTA-cooperative PRMT5 inhibitors.

Despite the genetic evidence for a synthetic lethal relationship between PRMT5 and *MTAP* deletion, the first generation of PRMT5 inhibitors that were developed do not demonstrate selectivity for MTAP-null cancer cell lines (Figure 1A and 1B). The lack of selectivity can be explained by the mechanisms of action of these inhibitors as they are either SAM cooperative or SAM competitive. SAM is the universal methyl donor utilized by methyltransferases, including PRMT5, to methylate their substrates. SAM-cooperative or SAM-competitive inhibitors of PRMT5 are not expected to be selective for MTAP-null cancers because they inhibit PRMT5 equally in MTAP-null and MTAP WT cells, ultimately leading to a molecule with limited therapeutic index.

We set out to design a compound that can utilize the accumulation of MTA by binding to PRMT5 in an MTA-cooperative, substrate-competitive manner, thereby achieving selective PRMT5 inhibition and killing of MTAP-null tumor cells while sparing MTAP-containing normal cells. Given the strong prevalence of *MTAP* deletion in the GBM patient population as well as in cancers that metastasize to the brain, we further aimed to design a compound with the physicochemical properties to enable it to cross the blood–brain barrier (BBB). To this end, we utilized high-throughput screening (HTS) and structure-based drug design (SBDD) to discover TNG908, a potent and selective, brain penetrant small molecule inhibitor of PRMT5 that acts via an MTA-cooperative mechanism and is currently in Phase I/II clinical trials (NCT05275478). Other early clinical compounds that are MTA-cooperative PRMT5 inhibitors are also shown in Figure 1D.^10−12^

## Results and Discussion

### Hit Finding

**Figure 2:**
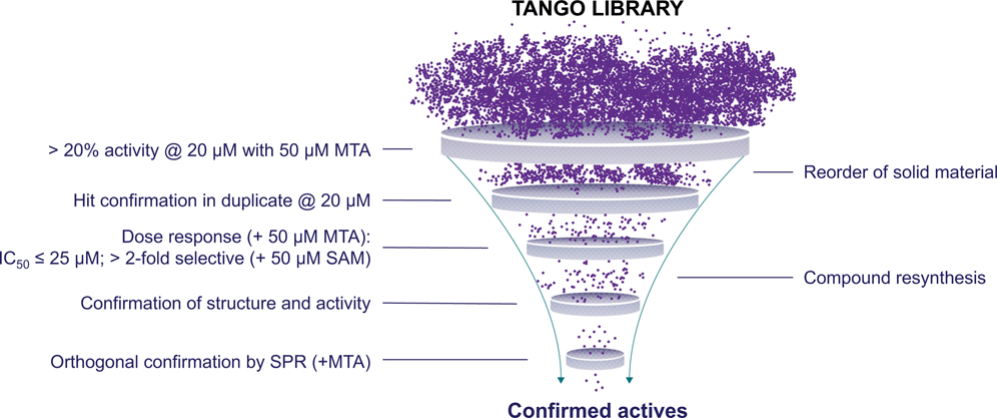
Hit triage strategy for compounds profiled in a high-throughput screen with a peptide displacement assay.

Having demonstrated proof of concept for selective killing of MTAP-null vs MTAP WT cells in a previous chemical series^13,14^ (Figure 1C), we deployed several hit-finding strategies to identify lead-like starting points for drug design, including DEL and fragment screens. However, the approach that offered the most appealing actives was a high-throughput screen of a 560k compound library at 20 μM using a peptide displacement assay. Fluorescence anisotropy (FA) was used to detect displacement of a TAMRA-labeled histone H4 peptide from PRMT5 in the presence of 50 μM MTA. Five hundred seventeen compounds, representing a 0.09% hit rate, had >20% activity at 20 μM. Hit triage proceeded through the following process by eliminating compounds that (1) had a dose response IC_50_ value > 25 μM in the peptide displacement assay with MTA, (2) had selectivity less than 2-fold in the presence of SAM (50 μM) versus MTA, (3) lost activity in the MTA-containing peptide displacement assay following resynthesis and structural confirmation, (4) did not confirm binding in an orthogonal surface plasmon resonance (SPR) assay with biotinylated PRMT5/MEP50 immobilized on a streptavidin sensor chip with 50 μM MTA in the running buffer (Figure 2).

**Figure 3:**
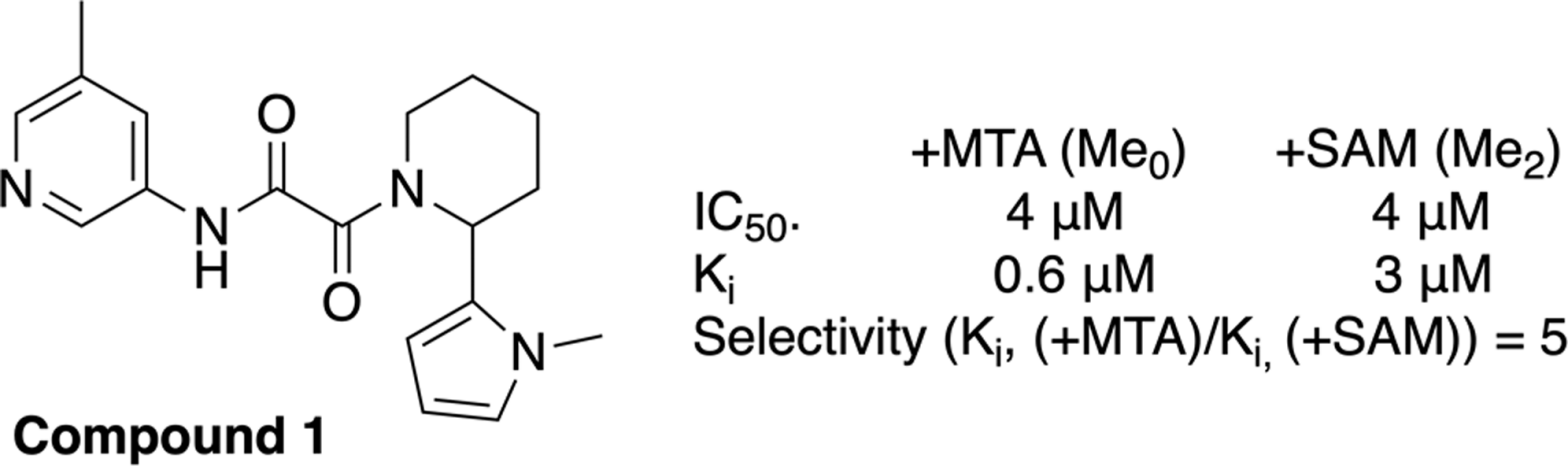
High-throughput screening hit compound **1** and associated PRMT5 biochemical assay data with MTA and SAM (see Supporting Information).

After completion of this process, 21 compounds, which represented 11 unique structural series, remained. Following extensive efforts at hit expansion, several of the series failed to demonstrate SAR tractability and were eliminated from further consideration. We focused our attention on the remaining three series with the most promising early SAR, and herein, we describe the development of one of those series that led to the discovery of TNG908, represented by hit compound **1** (Figure 3), which had a *K*_i_ with MTA of 600 nM in the peptide displacement assay with 5× selectivity and a *K*_d_ of 400 nM in the SPR assay with MTA.

### Medicinal Chemistry

The low molecular weight (326 Da) and good properties (cLogP = 2, PSA = 67, rotatable bonds = 3) of compound **1** were appealing starting point characteristics. Despite appearing as a structural alert in known databases^15−17^ due to the presence of the dicarbonyl moiety, we recognized the oxamide as an unreactive, nonelectrophilic group, stable in biological media, and a viable substructure for medicinal chemistry optimization,^18^ in contrast to ketoamides and oxamates which are highly susceptible to nucleophilic attack and hydrolysis, respectively.

Racemic **1** was separated by SFC to give enantiopure isomers compounds **1R** and **1S**. The stereochemistry of the compounds was initially assigned arbitrarily and later confirmed absolutely. Differential activity of the **1R** and **1S** isomers in the peptide displacement biochemical assay (+MTA) of 2 and 33 μM, respectively, demonstrated the preference for *R* stereochemistry at the 2 position of the piperidine. SAR exploration was initiated with the following approach: (1) systematic preparation of matched molecular pairs to investigate the importance of individual atoms or groups to molecular shape, electronics, and ultimately to binding,^19−21^ (2) ring forming and breaking to explore the limitations and benefits of conformational restrictions, (3) exploration of isosteres, and (4) investigation of tolerance to substitution around the molecule primarily via a “methyl walk”. These approaches were applied to the core structure to gain potency with minimal increase in molecular weight and rotatable bond count before growing the molecule to gain further potency. This work focuses on a portion of these efforts, the remainder of which will be reported in subsequent publications.

A broad assessment of the 2 position of the piperidine to replace the pyrrole was performed, and a representative subset of these compounds is shown in Table 1. There was a significant tolerance for replacement by a variety of substituents. For example, **8** has a similar potency to **1** with a lower cLogP and is more selective. **10** is more potent and selective than **1** and does not have the aromatic ring. Smaller substituents such as the methyl in **9**, however, lose significant activity. For early SAR development, we fixed the 2 position of the piperidine as phenyl, as exemplified in compound **2R**.

**Table 1:**
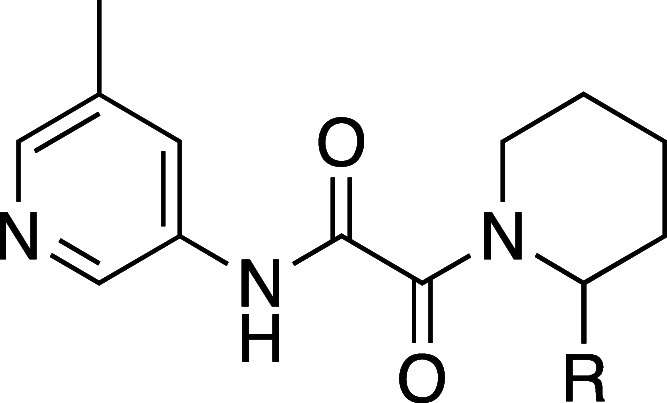

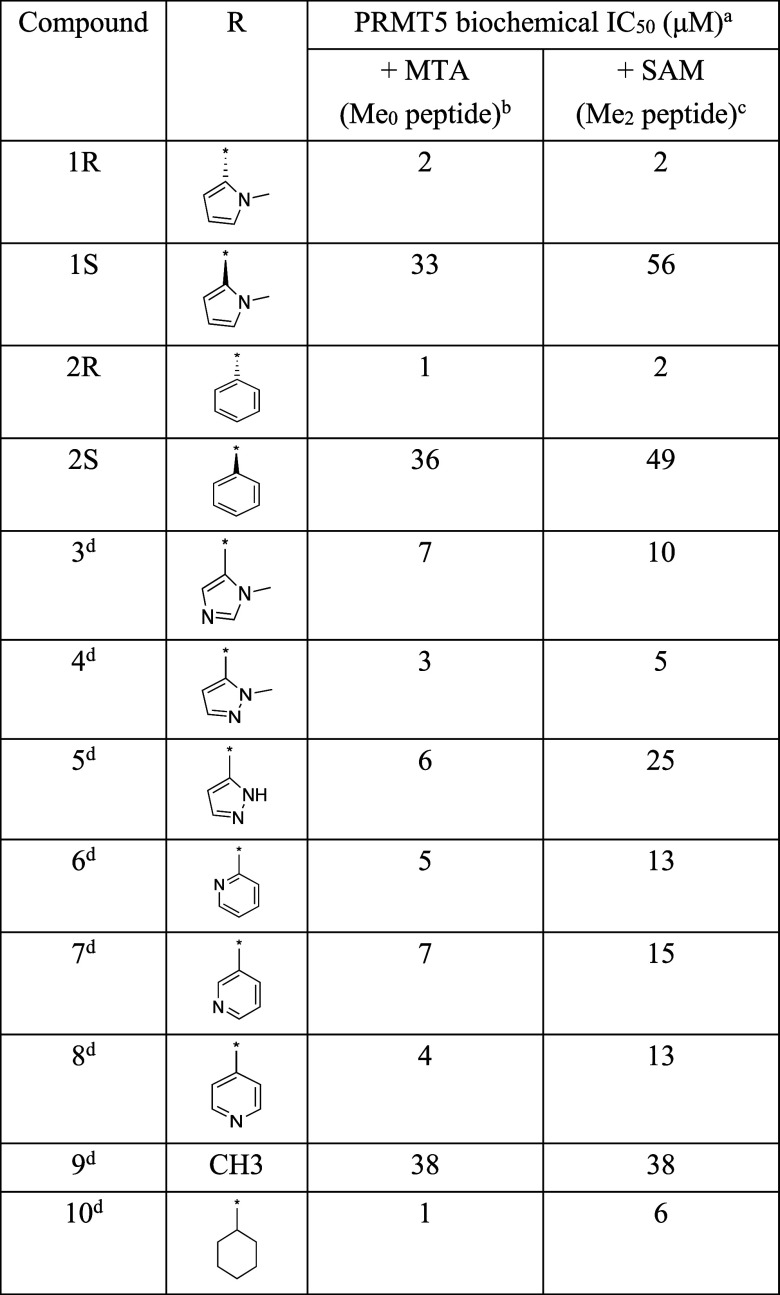
Compounds To Explore the Piperidine 2 Position, **1–10**

a Detection of displacement of a TAMRA-labeled peptide by fluorescence anisotropy.

b Unmethylated peptide (see Supporting Information).

c Dimethylated peptide used to prevent turnover in the assay (see Supporting Information). Conversion of IC_50_ to *K*_i_ by the Cheng–Prusoff equation^22^ was used for selectivity comparisons.

d Racemic.

**Figure 4:**
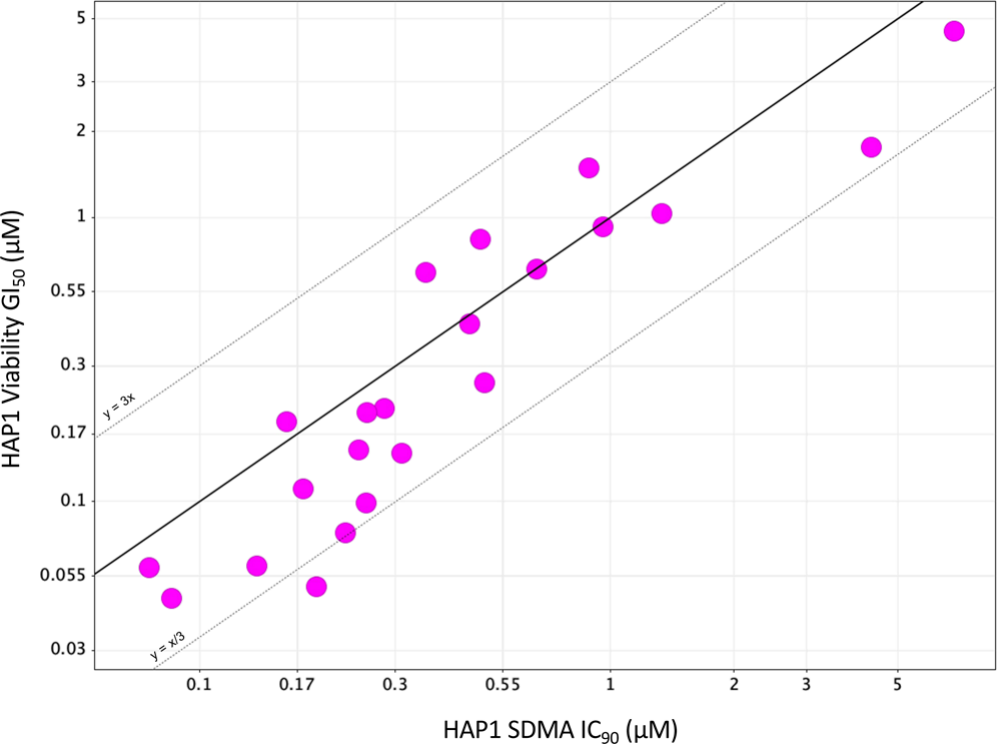
Demonstration of the correlation of HAP1 cellular viability GI_50_ and HAP1 cellular SDMA IC_90_ for compounds in this manuscript, representing the trend in the series.

At this point we performed a methyl scan: the preparation of a series of compounds that are matched molecular pairs to the starting analog, where a methyl group is substituted systematically in each position. This was done to probe tolerance to substitution, identify potential binding pockets, restrict conformations, and remove the hydrogen-bond donor. Most methylations either reduced activity or had no effect (compounds **11**, **14**, **17**, **18**, **19**, **20**, and **21**), though several methylations showed excellent potency gains (Table 2). For example, a cis methyl at the 6 position of the piperidine (**15**) improved potency in the biochemical assay but only 3-fold, which was not sufficient to give cellular activity. However, a methyl group in the 5 position of the piperidine trans to the 2-phenyl substituent (**16**) gave a 20-fold increase in biochemical potency with MTA and maintained selectivity. This level of potency approached the sensitivity limits of the biochemical assay, so for more potent compounds, we followed the SAR with two routine cellular assays. Using an isogenic pair of HAP1 MTAP WT and MTAP-null cell lines, PRMT5-dependent symmetric dimethylarginine (SDMA) was quantified by in-cell western (ICW) assay, and cell viability was measured using a 7-day CellTiter Glo assay. An observation throughout the program was the requirement to achieve PRMT5 SDMA IC_90_ to confer viability effects. SDMA IC_90_ consistently correlates with viability GI_50_ (Figure 4). **16** provided the first demonstration of cellular activity in MTAP-null cells (MTAP-null SDMA IC_50_ = 3.5 μM, MTAP WT SDMA IC_50_ > 10 μM) in the series. Dimethylation of the piperidine with the combined substitutions from **15** and **16** gave **22**, which was equipotent to the monomethyl analogs (**15** and **16**). On the other side of the molecule, adding a methyl to the 6 position of the pyridine (**12**) improved potency 16-fold and provided selective cellular activity (MTAP-null SDMA IC_50_ = 3.5 μM, MTAP WT SDMA IC_50_ > 10 μM).

**Table 2:**
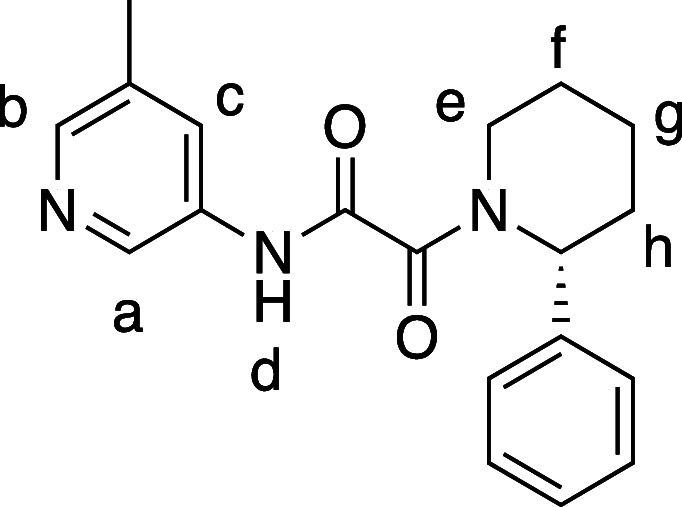
Biochemical and Cellular Potency of Select Compounds in a Methyl Scan of Compound **2R**

		biochemical IC_50_ (μM)^a^		SDMA IC_50_ HAP1 (μM)^d^	
compound	methyl position	MTA (Me_0_)^b^	SAM (Me_2_)^c^	MTAP-null	MTAP WT
**11**	a	>125	>125		
**12**	b	0.06	0.20	3.5	>10
**13**	c	0.9	11		
**14**	d	>125	>125		
**15**	e, cis	0.3	0.5		
**16**	f, trans	0.05	0.08	3.5	>10
**17**	f, cis	2	3		
**18**	g, trans	0.5	6		
**19**	g, cis	0.5	3		
**20**	h trans	3	5		
**21**	h cis	4	40		
**22**	e, f (2*R*,3*S*,6*R*)	0.2	0.6	4.5	>10

a Detection of displacement of a TAMRA-labeled peptide by fluorescence anisotropy.

b Unmethylated peptide (see Supporting Information).

c Dimethylated peptide used to prevent turnover in the assay (see Supporting Information). Conversion of IC_50_ to *K*_i_ by the Cheng–Prusoff equation^20^ was used for selectivity comparisons.

d Inhibition of PRMT5 determined by an SDMA in-cell western assay in the HAP1 *MTAP*-isogenic cell line pair following 24 h compound treatment.

**Figure 5:**
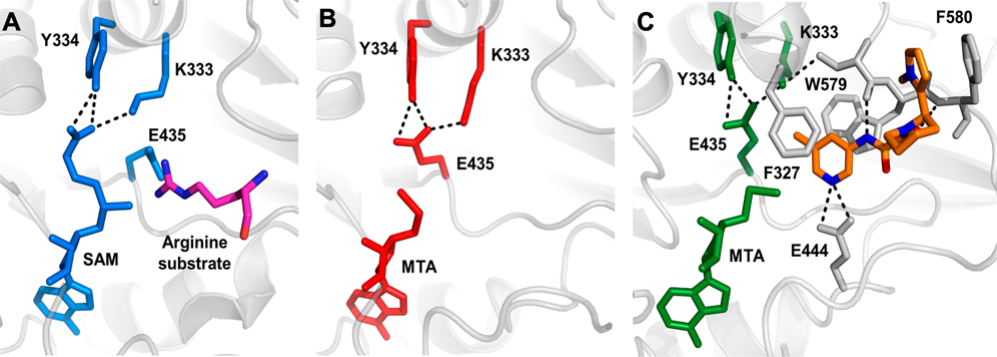
X-ray cocrystal structure of (A) SAM bound to PRMT5 with a substrate arginine side chain depicted. Glu435 rotates toward substrate side chain (PDB 4X61 and 5FA5). (B) X-ray cocrystal structure of MTA bound to PRMT5. E435 rotates to fill space previously filled by SAM (PDB 8VEO). (C) X-ray cocrystal structure of **1** (orange) bound to PRMT5·MTA (PDB 8VET). Only density for compound **1R** was observed. Pyridine sits in a π-stack “sandwich” with Phe327 and Trp579. Pyridine nitrogen hydrogen bonds with Glu444 and oxamide NH, and both carbonyls engage in hydrogen bonds. Pyrazole π-stacks with Phe580. Glu435 rotamer present with MTA alone is reinforced by steric pressure from the 3-methyl of **1** and engages Tyr334 and Lys333, precluding SAM binding.

Soon after the discovery of the *trans*-2-phenyl-5-methylpiperidine we obtained the cocrystal structure of **1** with PRMT5-MTA (Figure 5C). Only density for **1R** was observed. Examination of the structure revealed several key interactions that were important for future design. The pyridine makes van der Waals contacts with MTA and has π-stacking interactions with the aromatic side chains of Phe327 and Trp579, and the nitrogen of the ring is hydrogen bonded with the acid of Glu444. The 5-methyl group on the pyridine points toward Lys333 and Glu435 and locks Glu435 into the preferred rotamer as when bound with MTA alone^1^ (Figure 5B), sterically blocking Glu435 from moving into the rotamer required for SAM binding (Figure 5A), and likely contributing to the observed selectivity similar to what has been reported by other groups.^11,23^ There is a small pocket near the 6 position of the pyridine, providing rationale as to why the 6-methylpyridine analog **12** improved potency (vide infra). The NH of the oxamide forms a hydrogen bond with the carbonyl of Ser578, while the two carbonyls of the oxamide are also engaged in hydrogen bonds, one with the backbone NH of Phe580 and the other mediated through a water to Leu312. The piperidine and its pyrrole substituent occupy an adjacent hydrophobic pocket, and the pyrrole π-stacks with Phe580.

Leveraging this new understanding of how **1** binds, we utilized SBDD with an early focus on exploring around the pyridine ring given its proximity to the MTA/SAM pocket. We hypothesized that productive interactions in that region of the pocket might offer opportunities to improve potency and reinforce the Glu435 rotamer, improving selectivity between MTAP-null and MTAP WT cells.

The 6 position of the pyridine is directed toward the backbone carbonyl of Glu435. Having already made the dimethyl compound (**12**) in the methyl scan, we knew substitution in the 6 position was tolerated, and we hypothesized that a 6-amino group could make a more productive interaction with the carbonyl. **23** (Table 3) was prepared and resulted in a 200-fold improvement in cellular potency relative to its matched molecular pair (**16**) as well as selective viability effects (HAP1 MTAP-null ICW 17 nM, HAP1 MTAP-null viability 420 nM, 14-fold viability selectivity).

**Table 3:**
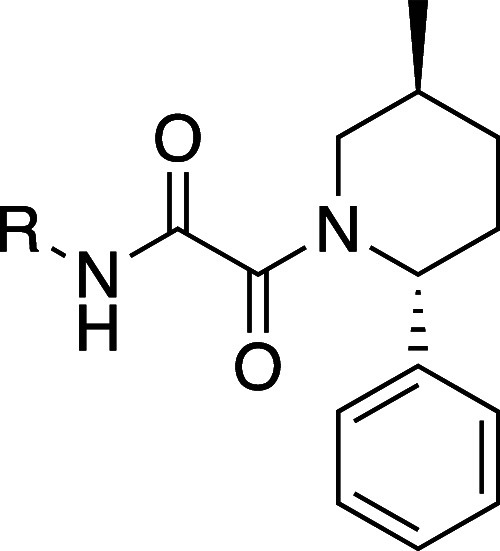

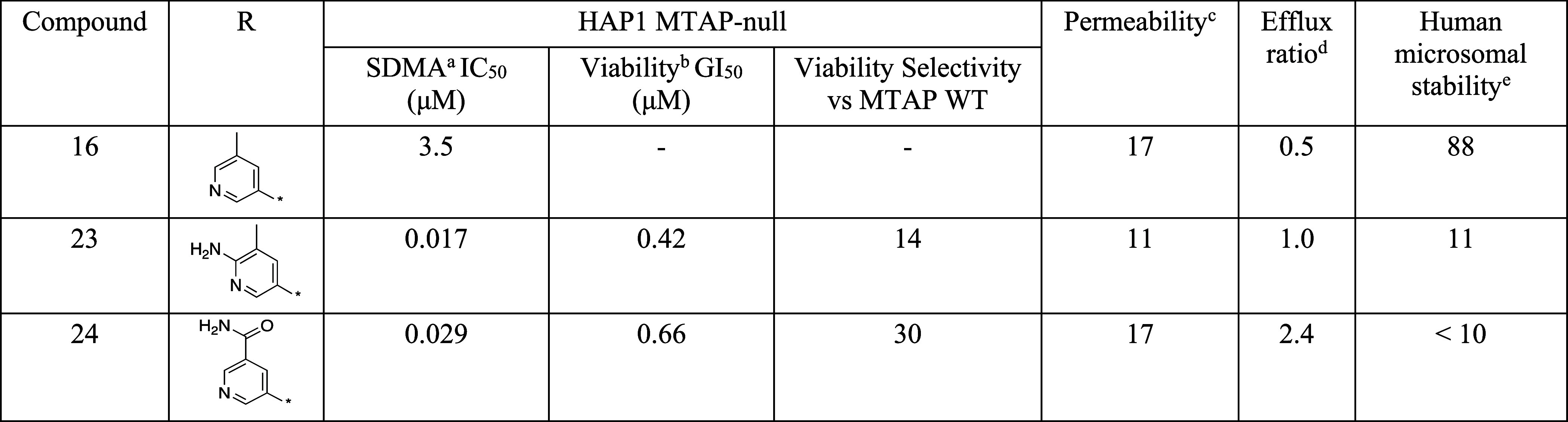
Characterization of Cellular Potency and In Vitro ADMET Properties of Compounds **23** and **24**

a Inhibition of PRMT5 determined by an SDMA in-cell western assay in the HAP1 *MTAP*-isogenic cell line pair following compound treatment for 24 h.

b Viability growth inhibition assessed after 7 days of compound treatment using a CellTiter-Glo luminescence-based assay in the same HAP1 *MTAP*-isogenic cells.

c MDCKII-WT A–B (10^–6^ cm/s).

d MDCKII-Mdr1 cells (A–B)/(B–A).

e Human liver microsomes, Cl_int_, μL/min/mg.

With the observation that the methyl group on the pyridine of **1** vectors toward Glu435 and Lys333, we prepared carboxamide analog **24**, attempting to engage the side chains of these residues. Gratifyingly, **24** gave a 120-fold improvement in potency and improved selectivity (HAP1 MTAP-null ICW 29 nM, HAP1 MTAP-null viability 660 nM, 30× viability selectivity) over the baseline methyl analog **16**. Importantly, the substitutions of both **23** and **24** provided improvements in selectivity and human microsomal stability while maintaining good permeability and efflux ratios (Table 3).

**Figure 6:**
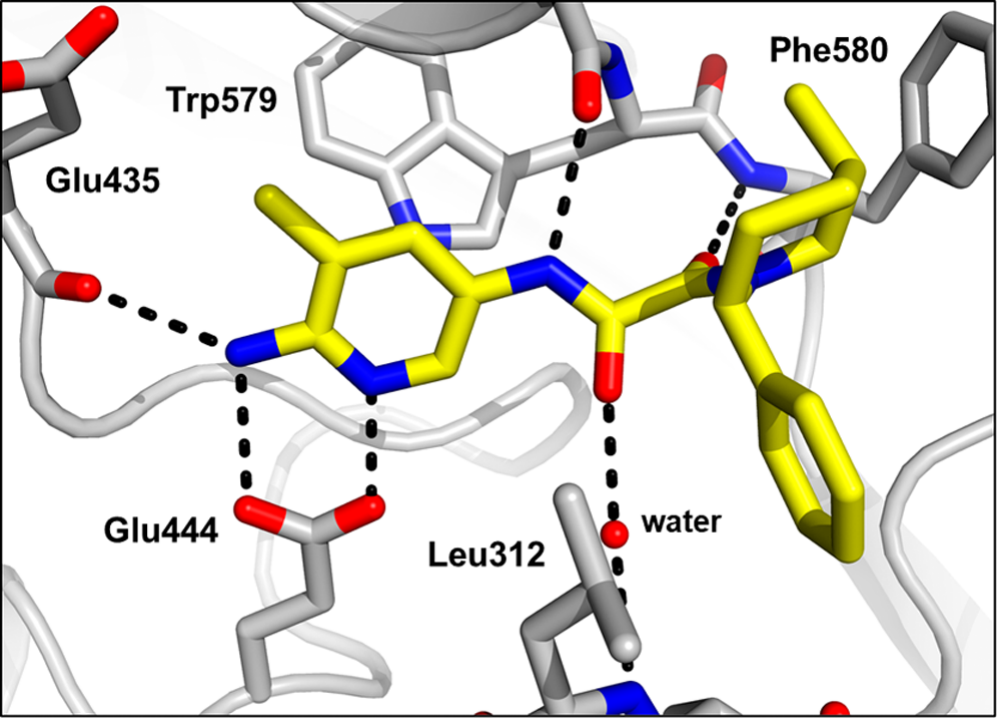
X-ray cocrystal structure of **23** (yellow) bound to PRMT5-MTA (PDB 8VEU). Aminopyridine engages the Glu435 backbone carbonyl and Glu444 side chain acid in hydrogen bonds.

**Figure 7:**
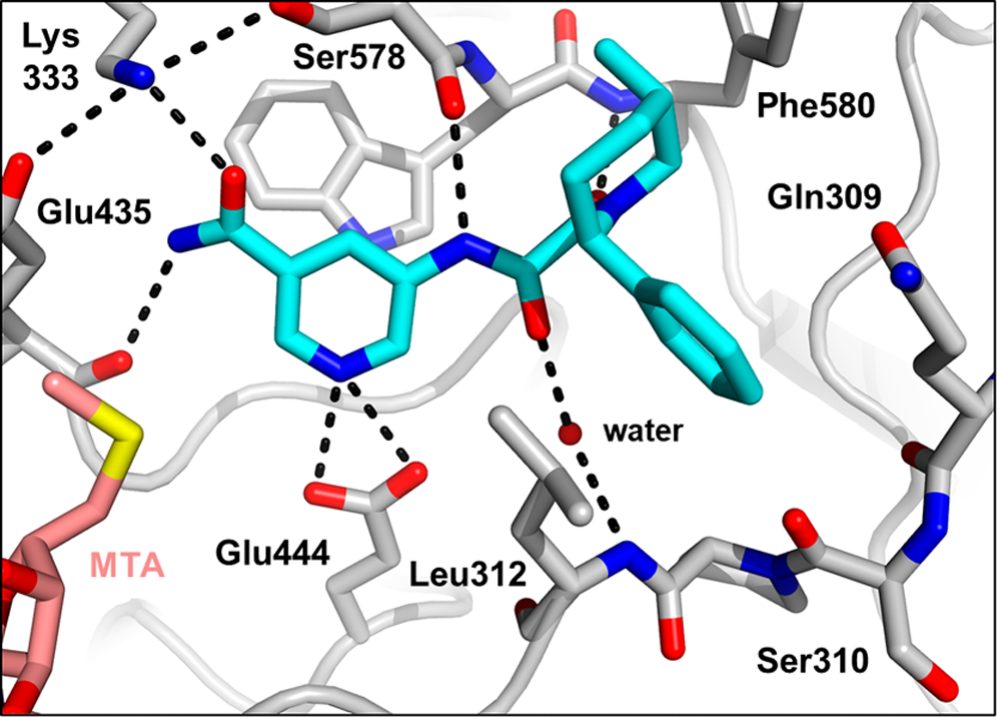
X-ray cocrystal structure of **24** (blue) bound to PRMT5-MTA (PDB 8VEW). Amide carbonyl engages Lys333 and amide NH_2_ engages the Glu435 backbone carbonyl in hydrogen bonds.

Crystal structures of **23** and **24** bound to the PRMT5·MTA complex were obtained (Figures 6 and 7). The structure of **23** confirmed that the NH_2_ at the 6 position of the pyridine makes strong hydrogen-bonding interactions with the backbone carbonyl of Glu435 and with the side chain of Glu444. The structure of **24** revealed that the amide NH_2_ makes a hydrogen bond with the backbone carbonyl of Glu435, while the amide carbonyl makes a hydrogen bond with the amino side chain of Lys333, which normally interacts with the carboxy terminus of SAM. In both cases, the locked rotamer of Glu435 that is incompatible with SAM binding is reinforced, providing insight into the increased selectivity. Another interesting observation was that the *trans*-2-phenyl, 5-methyl-substituted piperidine in the X-ray structures of **23** and **24** is rotated nearly 180° in the pocket relative to the 2-pyrrole-substituted piperidine in **1**, orienting the phenyl ring toward the solvent. This substitution-dependent rotation of the piperidine side of the molecule was observed throughout the program.

While developing the SAR we also explored replacements of the oxamide moiety. Cyclization from the pyridine onto the oxamide provided several active compounds with similar potency, but the properties of these cyclized compounds suffered relative to the oxamides. Select analogs are shown in Table 4. For example, despite promising potency, many were active against hERG (exemplified by compounds **25** and **26)**, had low solubility (exemplified by **27**), or had poor metabolic stability (exemplified by compounds **26** and **27**). Some effort to find a balance of properties was undertaken, but considering the suboptimal properties in early cyclized compounds and the rapid progress being made in the oxamide series, we decided to prioritize the oxamide series.

**Table 4:**
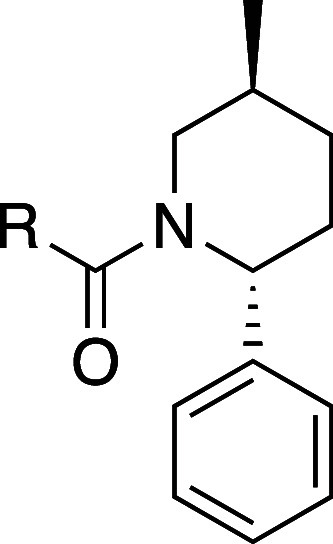

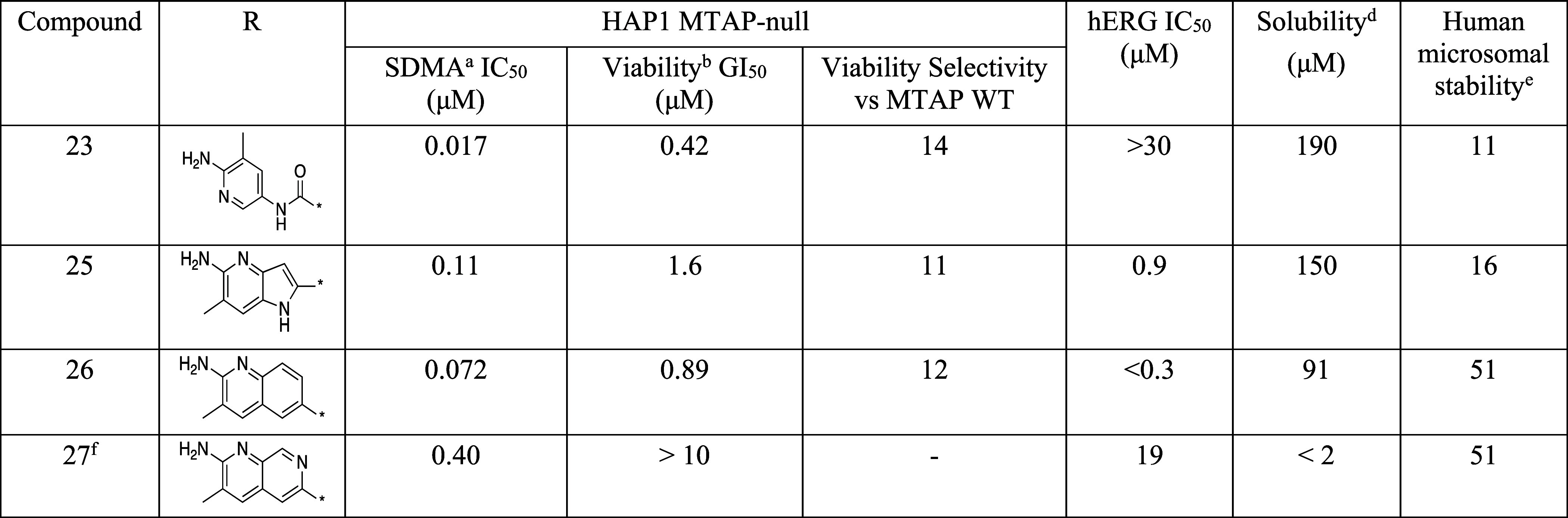
Characterization of Cellular and hERG Potency, Solubility, and Metabolic Stability of Oxamide Isosteres, Compounds **25–27**

a Inhibition of PRMT5 determined by an SDMA in-cell western assay in the HAP1 *MTAP*-isogenic cell line pair following compound treatment for 24 h.

b Viability growth inhibition assessed after 7 days compound treatment using a CellTiter-Glo luminescence-based assay in the same HAP1 *MTAP*-isogenic cells.

c hERG channel IC_50_ measured using the automated patch clamp method (SyncroPatch 384PE).

d Kinetic solubility measured in a phosphate buffer system at pH 7.4.

e Human liver microsomes, Cl_int_, μL/min/mg.

f Racemic.

Further examination of the crystal structures of **23** and **24** revealed the phenyl rings on the piperidine approach the backbone carbonyl of Ser310. We hypothesized that this carbonyl could be leveraged for potency through a well-placed hydrogen-bond interaction. To test the hypothesis, two analogs with a *para*-hydroxy were prepared (**28** and **29**, Table 5). **28** (HAP1 MTAP-null viability 60 nM, 23-fold viability selectivity) was 7-fold more potent than the baseline phenyl (**23**), and **29** (HAP1 MTAP-null viability 190 nM, 75-fold viability selectivity) was 3-fold more potent than the baseline phenyl (**24**) and more selective.

**Table 5:**
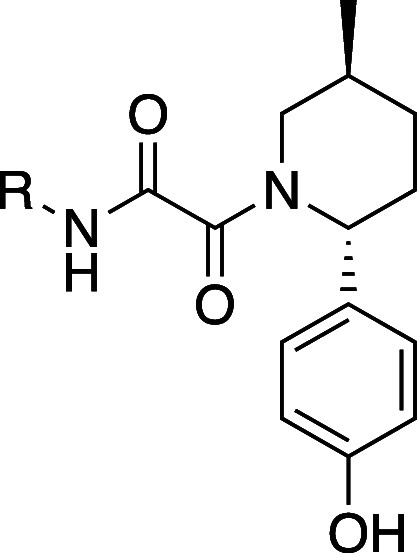

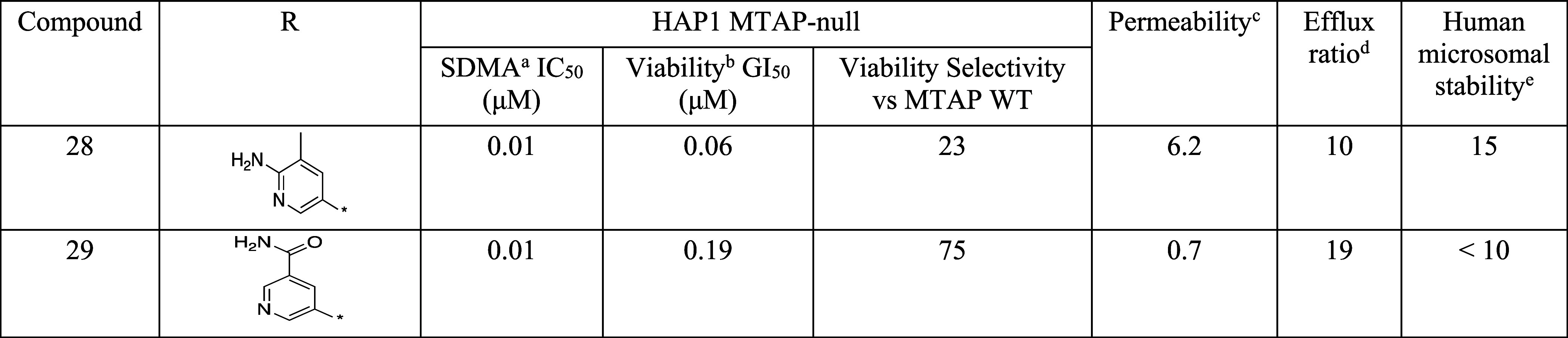
Characterization of Cellular Activity, Permeability, Efflux, and Metabolic Stability of Compounds **28** and **29**

a Inhibition of PRMT5 determined by an SDMA in-cell western assay in the HAP1 *MTAP*-isogenic cell line pair following compound treatment for 24 h.

b Viability growth inhibition assessed after 7 days of compound treatment using a CellTiter-Glo luminescence-based assay in the same HAP1 *MTAP*-isogenic cells.

c MDCKII-WT A–B (10^–6^ cm/s).

d MDCKII-Mdr1 cells (A–B)/(B–A).

e Human liver microsomes, Cl_int_, μL/min/mg.

**Figure 8:**
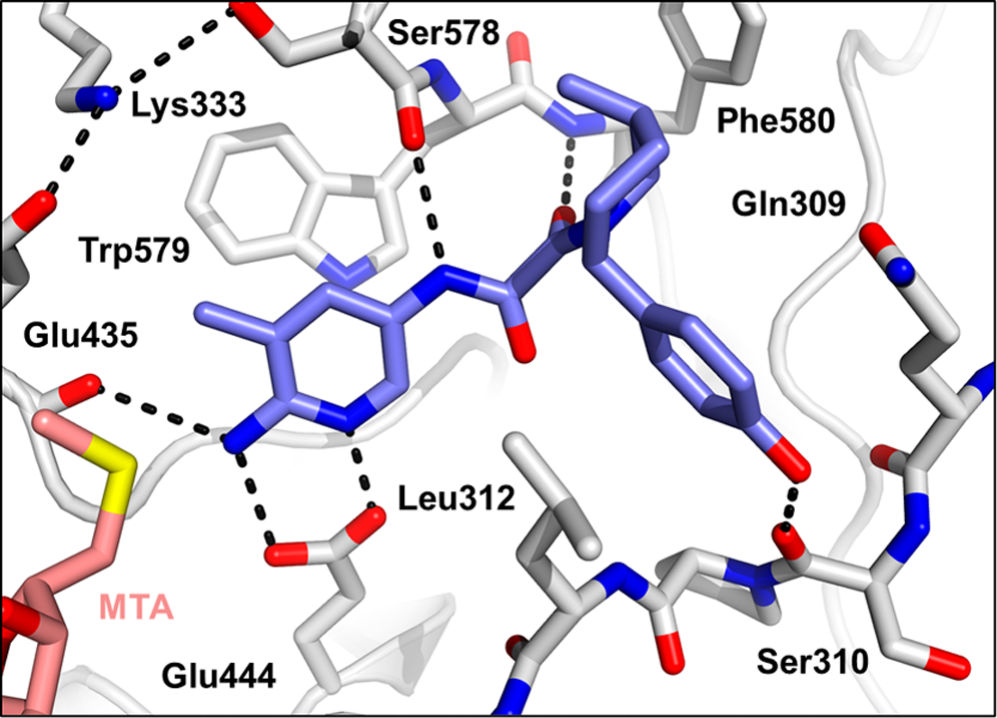
X-ray cocrystal structure of compound **28** (purple) bound to PRMT5-MTA (PDB 8VEX). Phenol hydrogen bonds with the backbone carbonyl of Ser310.

Crystal structures confirmed that the OH of the phenol made a hydrogen bond with the carbonyl of Ser310 (Figure 8). While the *para*-phenol ring improved potency, it also had the undesired effects of reducing permeability and increasing efflux in MDCKII-Mdr1 cell lines, properties that were not in line with the goal of developing a BBB penetrant molecule. To gain the potency benefits of the interaction but reduce the property liabilities, we explored phenol isosteres.

A series of compounds (representative set in Table 6) was prepared in both the amine and the carboxamide subseries. Many analogs showed good potency improvements relative to the unsubstituted phenyl in the aminopyridine series. However, despite the carboxamide analogs generally having greater selectivity, phenol isosteres were less tolerated in this series and most analogs either reduced potency or had only modest improvements relative to the unsubstituted phenyl. Crystallography substantiated the persistence of an H bond to the Ser310 carbonyl with these compounds.

**Table 6:**
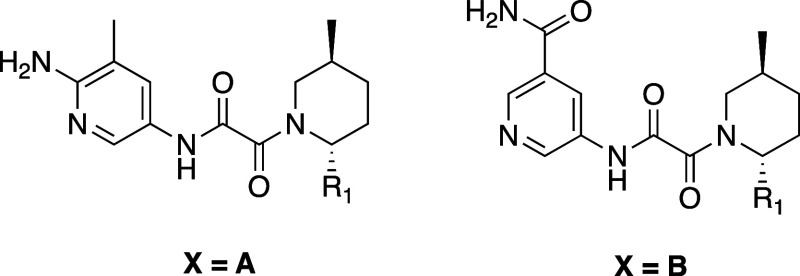

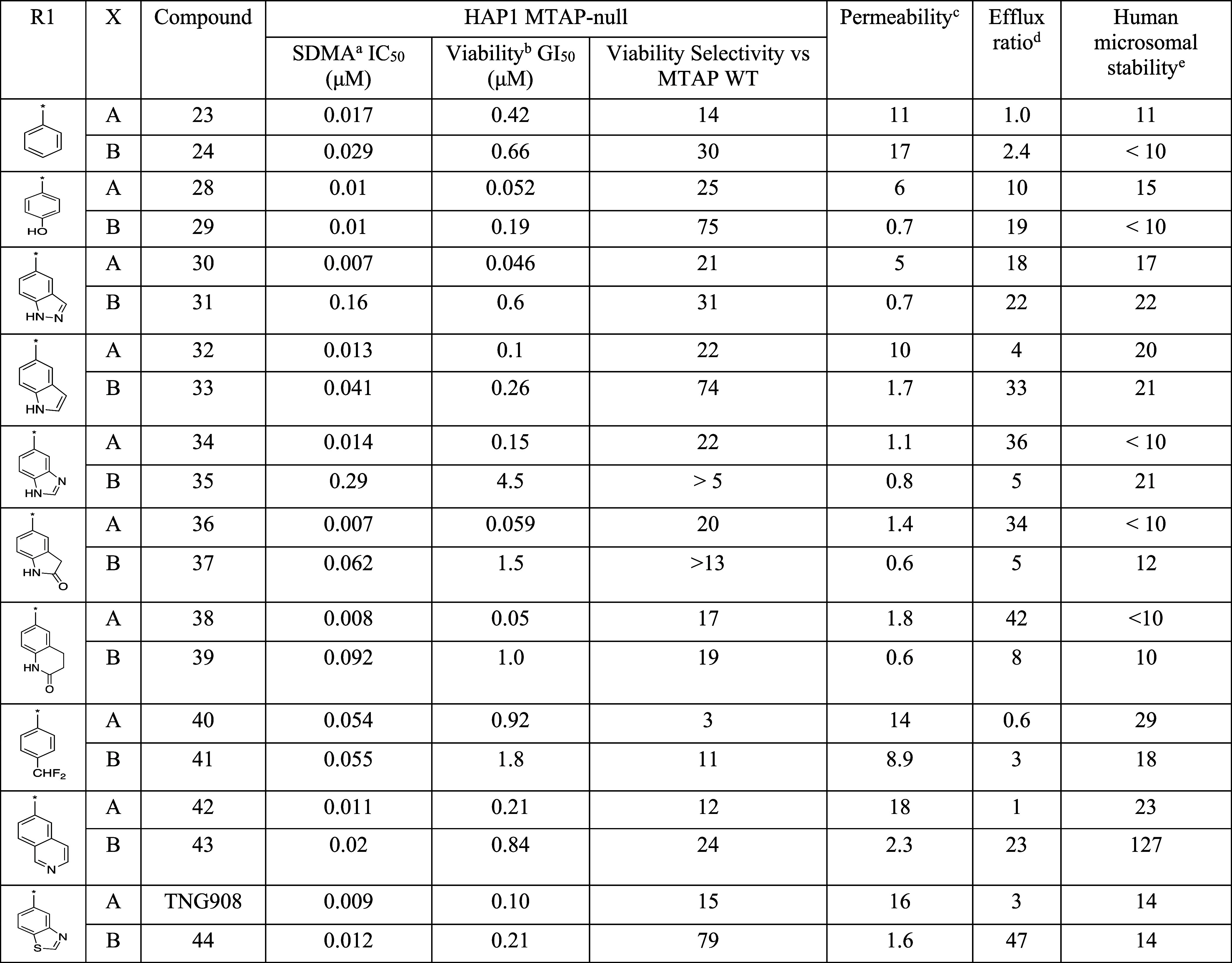
Characterization of Cellular Activity, Permeability, Efflux, and Metabolic Stability of Compounds **30–44** and TNG908

a Inhibition of PRMT5 determined by an SDMA in-cell western assay in the HAP1 *MTAP*-isogenic cell line pair following compound treatment for 24 h.

b Viability growth inhibition assessed after 7 days of compound treatment using a CellTiter-Glo luminescence-based assay in the same HAP1 *MTAP*-isogenic cells.

c MDCKII-WT A–B (10^–6^ cm/s).

d MDCKII-Mdr1 cells (A–B)/(B–A).

e Human liver microsomes, Cl_int_, μL/min/mg.

In general, as with the phenol, the isosteric compounds with the additional NH group exhibited lower permeability and higher efflux than the baseline phenyl compounds **23** and **24** (permeability MDCKII-WT A–B = 10.6 and 17.1 × 10^–6^ cm/s and efflux ratios = 1.0 and 2.4, respectively), more so in the carboxamide series (Table 6). For example, all NH-containing phenol isosteres in the carboxamide series had permeability in the MDCKII-WT assay < 2 × 10^–6^ cm/s (A–B) and efflux ratio > 20.

Of notable interest, however, was the benzothiazole analog, prepared in an attempt to gain the less common interaction of a C–S σ* orbital with the carbonyl lone pair. Such an interaction had the potential to achieve potency gains without the addition of an H-bond donor group, thereby improving permeability and efflux relative to the phenol.^24^ The incorporation of the benzothiazole gave a potency improvement similar to compounds incorporating the traditional NH (TNG908 HAP1 MTAP-null viability 100 nM, 15-fold viability selectivity and **44** HAP1 MTAP-null viability 210 nM, 79-fold viability selectivity). In the case of carboxamide compound **44**, efflux was still high (efflux ratio = 44). However, TNG908, with its lower PSA (101 vs 118) and fewer hydrogen-bond donors relative to the carboxamide, had high permeability (MDCKII-WT A–B = 16.4 cm/s), low efflux (efflux ratio = 3), and good metabolic stability (human microsomal stability CL_int_ = 14 μL/min/mg).

**Figure 9:**
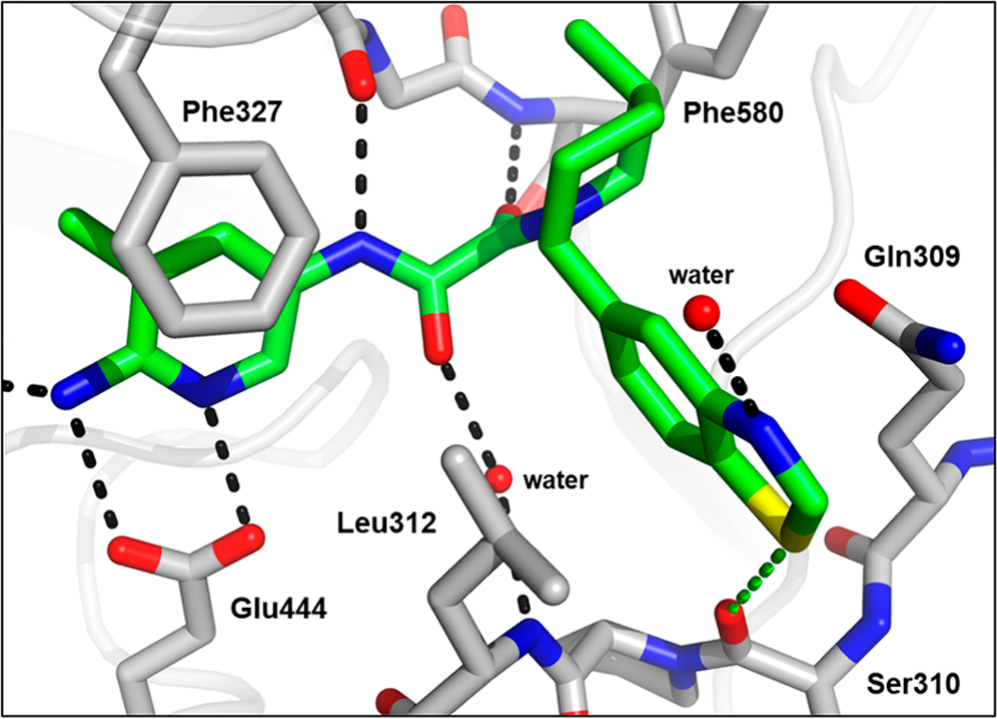
X-ray cocrystal structure of TNG908 (green) bound to PRMT5-MTA (PDB 8VEY). Benzothiazole hydrogen bonds with water, and the C–S σ* orbital interacts with the carbonyl lone pair of Ser310.

An X-ray structure of TNG908 (Figure 9) confirmed that the carbonyl oxygen is located 3.4 Å from the sulfur atom and 2.6 Å from the H at the 7 position of the benzothiazole with a O–S–C angle of 167°, and the N of the benzothiazole engages a water molecule in a hydrogen bond, all likely contributing to the potency gains relative to the unsubstituted phenyl.^25,26^

Other non-traditional hydrogen-bond donors^27,28^ were tested in an attempt to avoid the polarity and associated lower permeability and higher efflux of traditional NH hydrogen-bond donors but were not as successful as the benzothiazole of TNG908. A CHF_2_ group was synthesized (**40** and **41**), but the potency was worse than the unsubstituted phenyl. An aromatic CH hydrogen bond was explored with **42** and **43**. Though **42** did provide a 2-fold potency gain relative to the unsubstituted phenyl with high permeability (MDCKII-WT A–B = 18.2 cm/s) and low efflux (efflux ratio = 1), it was less metabolically stable (human microsomal stability CL_int_ = 23 μL/min/mg), and the potency was not sufficient for further consideration.

Considering the greater selectivity in the carboxamide subseries, we explored ways to improve the potency and reduce the impact of the high polarity of the carboxamide on permeability and efflux. We prepared a series of 2-methoxy-3-carboxamido pyridine analogs, represented by compounds **45**–**47**, in an attempt to mask the amide NH with an intramolecular hydrogen bond, a selection of which is shown in Table 7. This substitution improved the permeability and reduced efflux while increasing the selectivity and potency relative to the carboxamide alone; however, metabolic stability suffered. These observations were generally consistent throughout the series, and therefore, we eventually deprioritized the subseries.

**Table 7:**
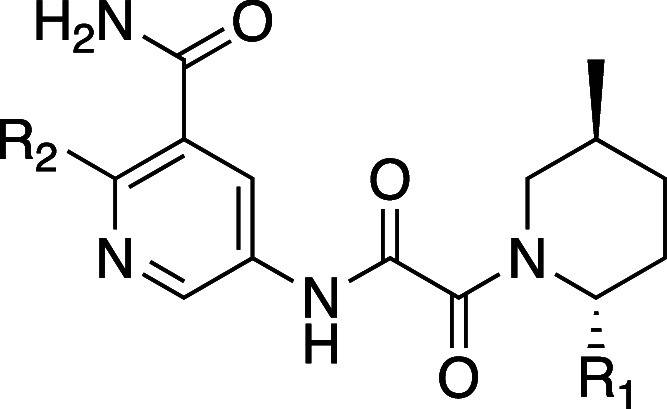

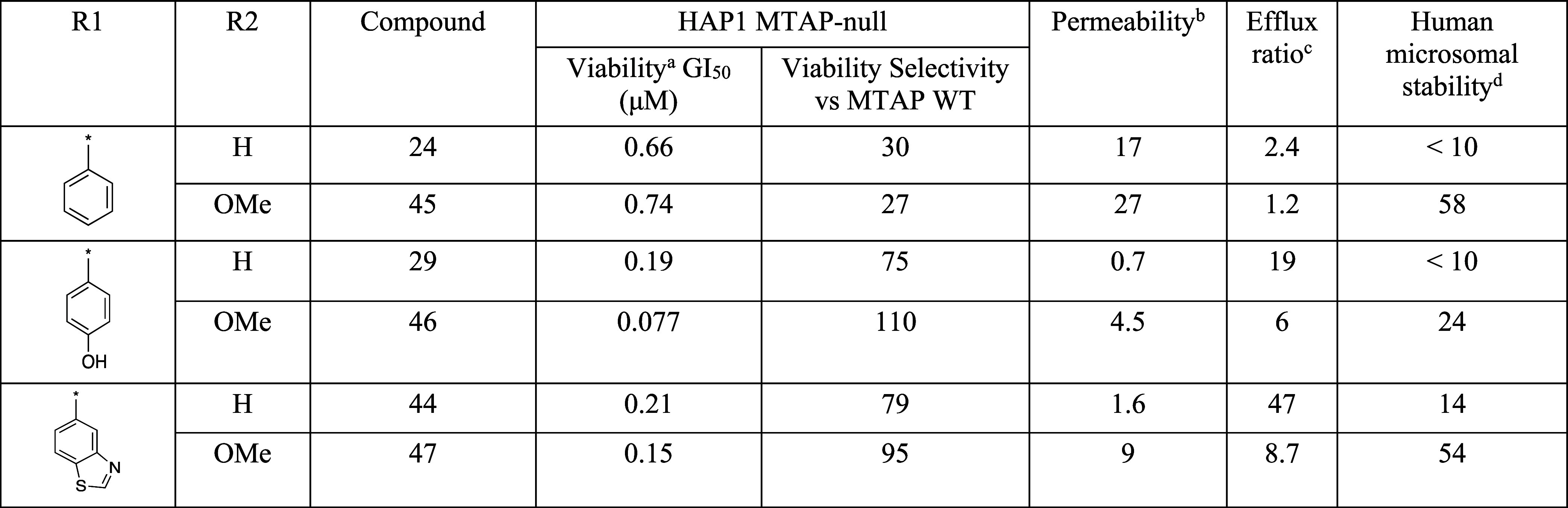
Characterization of Cellular Activity, Permeability, Efflux, and Metabolic Stability of Compounds **45–47**

a Viability growth inhibition assessed after 7 days of compound treatment using a CellTiter-Glo luminescence-based assay in HAP1 *MTAP*-isogenic cells.

b MDCKII-WT cells, A–B (10^–6^ cm/s).

c MDCKII-Mdr1 cells (A–B)/(B–A).

d Human liver microsomes, Cl_int_, μL/min/mg.

At this point we had a series of compounds with good potency and selectivity, and with TNG908 we also had a desirable ADME-PK profile of high permeability (MDCKII A–B = 16 cm/s), low efflux (MDCKII-Mdr1 efflux = 3), and good human microsomal stability (14 μL/min/mg). Human hepatocyte intrinsic clearance was also low (9 mL/min/kg), leading to the decision to further profile TNG908.

### Biochemical and Biophysical Characterization

TNG908 and other potent MTA-cooperative inhibitors reached the detection limit of our SAR-driving assays, so more sensitive biochemical and biophysical methods to measure potency were developed. First, we performed double titrations of PRMT5 and TNG908 ± 50 μM MTA using the FA assays, allowing survey of lower PRMT5 and compound concentrations. TNG908 binds apo-PRMT5 with a *K*_D_ 1.9 ± 0.9 nM and to the PRMT5·MTA complex with a *K*_D_ = 0.3 ± 0.1 nM, a 6-fold increase in potency in the presence of MTA (Table 8). We also developed a radioactive biochemical FlashPlate assay using ^3^H-SAM and biotinylated histone H4-peptide substrate to measure inhibition. The *K*_M_s for SAM and the biotinylated H4 peptide in the assay are 0.2 and 0.125 μM, respectively, and the measured *K*_i_ of MTA is 0.25 μM. Since TNG908 is a competitive inhibitor of the H4 peptide substrate, we measured its activity in an assay using 10 μM H4 peptide and the presence or absence of MTA at a concentration equivalent to its *K*_i_ (0.25 μM). Through competition experiments with the H4 peptide, we further resolved the increased potency of TNG908 due to MTA cooperative binding and the resulting enzyme inhibition, calculating the apparent *K*_i_ values of TNG908 using the Cheng–Prusoff equation.^20^ TNG908 inhibited apo-PRMT5 with an IC_50_ of 262 ± 52 nM and a *K*_i,app_ = 3.2 nM but showed 12-fold stronger potency for the PRMT5·MTA complex with an IC_50_ of 21.2 ± 9.3 nM and *K*_i,app_ = 0.26 nM (Table 8). To further assess the binding kinetics and potency of TNG908, we carried out reversibility studies utilizing a combination of size exclusion column separation and FlashPlate assay experiments. TNG908 was preincubated with apo-PRMT5 or the PRMT5·MTA complex for 2 h at room temperature and then passed through Zeba spin columns to remove excess compound and MTA. The flow-through contained either PRMT5·TNG908 binary complex or PRMT5·MTA·TNG908 ternary complex. The recovery of PRMT5 enzyme activity due to inhibitor dissociation was followed in time course by FlashPlate assay, indicating that TNG908 behaved as a reversible, tight-binding inhibitor for both apo-PRMT5 and PRMT5·MTA complexes over the course of the assay. Inhibition due only to MTA during the assay is assumed to be negligible due to the low concentration of enzyme and spin column removal of excess MTA. Compared to a DMSO control, approximately 70% enzyme activity was recovered from PRMT5·TNG908 binary complex compared to 30% of recovered enzyme activity from PRMT5·MTA·TNG908 ternary complex, reflecting that TNG908 preferentially binds to PRMT5·MTA complex. The observed linear kinetic behavior of TNG908 suggested that a new equilibrium between the compound and PRMT5 had been established, allowing estimation of TNG908 *K*_i_ to either apo PRMT5 or PRMT5·MTA complex using the equation *K*_i,app_ = (*E*_total_ – *E*·*I*) × (*I*_total_ – *E*·*I*)/*E*·*I* (see Supporting Information). The estimated *K*_i,app_ of TNG908 to apo PRMT5 is 9.3 ± 2.5 nM, while the estimated *K*_i,app_ of TNG908 to PRMT5·MTA complex is 0.73 ± 0.11 nM, suggesting a 12.7-fold potency selectivity for PRMT5·MTA complex. By considering the competition of 1 μM H4 peptide substrate in the assay, the real *K*_i_’s were calculated again using the Cheng–Prusoff equation, yielding a TNG908 *K*_i_ for apo PRMT5 = 1.03 nM and TNG908 *K*_i_ for the PRMT5·MTA complex = 0.081 nM. All potencies measured by the various biochemical approaches are within 3–4-fold of one another, indicative of the highly potent MTA-cooperative inhibition of TNG908.

**Table 8:** Biochemical Characterization of TNG908 with and without MTA in Double-Titration and Radioactive FlashPlate Assays

assay		PRMT5·MTA	apo-PRMT5
double titration	*K*_D_, nM	0.3 ± 0.1	1.9 ± 0.9
radioactive FlashPlate	IC_50_, nM	21.2 ± 9.3	262 ± 52
	*K*_i,app_, nM	0.26	3.2

## Pharmacokinetic Profiling of TNG908 in Preclinical Species

The PK properties of TNG908 were evaluated in Sprague–Dawley (SD) rats, beagle dogs, and cynomolgus monkeys (Table 9). Following a 1 mg/kg dose of TNG908 to SD rats (*n* = 3), the clearance was 38.2 mL/min/kg, volume of distribution was 1.5 L/kg, and half-life was 2 h. Oral administration of a 3 mg/kg dose of TNG908 to SD rats (*n* = 3) resulted in a *C*_max_ of 0.60 μg/mL and AUC_inf_ of 2.5 h-μg/mL, with >100% bioavailability. The greater than 100% bioavailability could result from experimental or biological variability as well as the dosing of the PO arm (3 mg/kg) 3-fold higher than the IV arm (1 mg/kg). The PK could be nonlinear between 1 and 3 mg/kg in the rat. Following a 1 mg/kg IV dose of TNG908 to beagle dogs (*n* = 3), the clearance was 8.6 mL/min/kg, volume of distribution was 1.2 L/kg, and half-life was 4 h. Oral administration of a 3 mg/kg dose of TNG908 to beagle dogs resulted in a *C*_max_ of 0.54 μg/mL and AUC_inf_ of 2.4 h-μg/mL, with 41% bioavailability. Following a 1 mg/kg IV dose of TNG908 to cynomolgus monkeys (*n* = 3), the clearance was 16.2 mL/min/kg, volume of distribution was 1.0 L/kg, and half-life was 1 h. Oral administration of a 3 mg/kg dose of TNG908 to cynomolgus monkeys (*n* = 3) resulted in a *C*_max_ of 0.087 μg/mL and AUC_inf_ of 0.7 h-μg/mL, with 21% bioavailability.

**Table 9:** In Vivo PK Characterization of TNG908

species	clearance (mL/min/kg)	*V*_ss_ (L/kg)	*T*_1/2_ (h)	*F* (%)	*K*_p,uu,CSF_^d^
rat^a^	38.2	1.5	2	>100	
dog^b^	8.6	1.2	4	41	
cynomolgus monkey^c^	16.2	1.0	1	21	0.9

a IV/PO dosing in Sprague–Dawley rat (vehicle, IV, 1 mg/mL solution of 1% v/v DMSO/99% of 20% w/v HP-β-CD in saline; PO, 3 mg/mL solution of 1% v/v DMSO/99% of 20% w/v HP-β-CD in water, *n* = 3 per arm).

b IV/PO dosing in beagle dog (vehicle, IV, 1 mg/mL solution of 1% v/v DMSO/99% of 20% w/v HP-β-CD in saline; PO, 3 mg/mL solution of 1% v/v DMSO/99% of 20% w/v HP-β-CD in water, *n* = 3 per arm).

c IV/PO dosing in cynomolgus monkey (vehicle, IV, 1 mg/mL solution of 1% v/v DMSO/99% of 20% w/v HP-β-CD in saline; PO, 3 mg/mL solution of 1% v/v DMSO/99% of 20% w/v HP-β-CD in water, *n* = 3 per arm).

d PO dosing in cynomolgus monkeys (vehicle, PO, 0.5% methylcellulose in water, *n* = 3), *K*p,uu,CSF determined by measuring CSF concentration and taking the ratio to free unbound plasma concentrations.

Considering the high permeability and low efflux of TNG908, we used a highly validated nonhuman primate model^29^ to further evaluate its potential to cross the BBB in vivo by measuring the concentration of TNG908 in cerebral spinal fluid (CSF) in cynomolgus monkeys and comparing to unbound plasma concentrations. This is a nonterminal experiment without the need to sacrifice monkeys for each time point of brain data collection. Following a 10 mg/kg PO dose of TNG908 to cynomolgus monkeys, the *K*_p,uu,CSF_ (the ratio of CSF, AUC, and unbound plasma AUC) was determined to be 0.9 (see Supporting Information). These data along with terminal unbound brain exposure from a preclinical toxicology study (data not shown) support the use of TNG908 to treat patients with *MTAP*-deleted GBM or other *MTAP*-deleted CNS malignancies.

## TNG908 Drives Pharmacodynamic Activity and Antitumor Efficacy in MTAP-null Xenograft Models Representing GBM, Lung, and Colorectal Cancer

**Figure 10:**
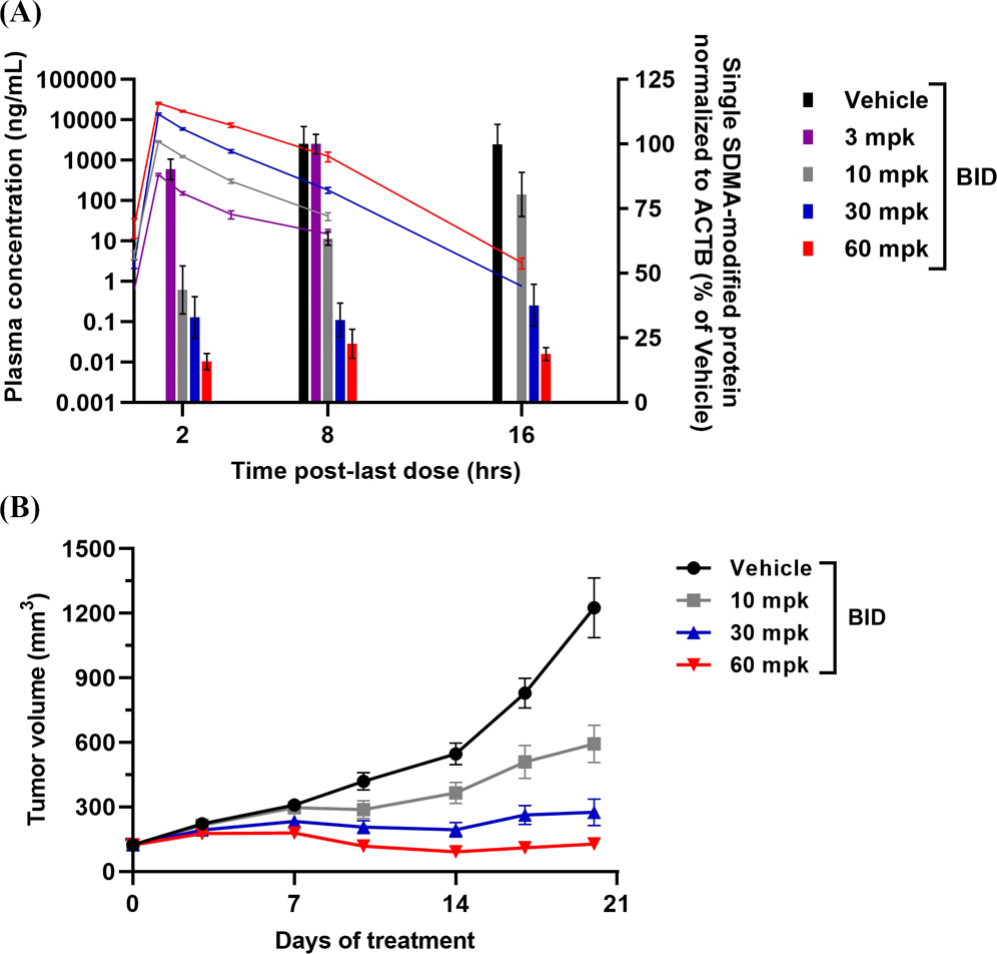
TNG908 treatment drives dose-dependent PD and antitumor activity in the LN18 MTAP-null GBM xenograft model. (A) PK/PD analysis of TNG908 following compound treatment for 10 days. PRMT5 activity by quantification of a single SDMA-modified protein substrate and total TNG908 plasma concentrations were determined at the indicated time points post last dose. *n* = 4 tumors/time point/dose level. Data are presented as mean ± SEM for the PD analyses. (B) Tumor growth inhibition curves for the LN18 xenograft model treated with either vehicle or TNG908 dosed at 10, 30, or 60 mg/kg BID for 21 days. *n* = 8 mice/group. Data are presented as mean ± SEM.

TNG908 was evaluated for pharmacodynamic activity and antitumor efficacy in the LN18 *MTAP*-deleted glioblastoma cell line-derived xenograft model implanted subcutaneously in NOG mice. Following a study to determine the maximum tolerated dose (MTD) in the NOG mouse strain, oral administration of TNG908 at well-tolerated doses (10, 30, or 60 mg/kg BID) resulted in dose-proportional plasma exposures with maximal concentrations observed 1 h after the last dose as well as dose-dependent PRMT5 inhibition as determined by the reduction of a single SDMA-modified protein (Figure 10A). Similarly, treatment with TNG908 drove dose-dependent antitumor activity with 100% tumor growth inhibition observed at 60 mg/kg BID following 21 days of dosing (Figure 10B).

**Figure 11:**
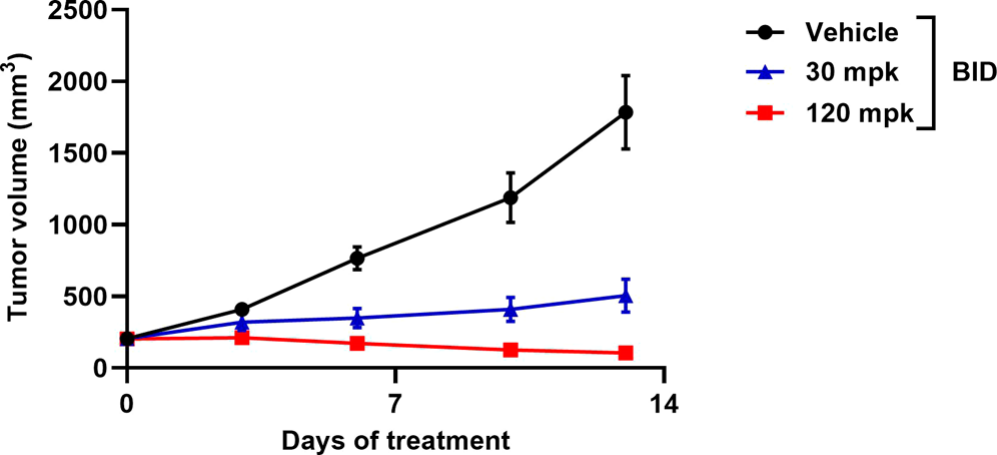
Tumor growth inhibition curves for the LU99 NSCLC cell line-derived xenograft mouse model with either vehicle or TNG908 dosed at 30 or 120 mg/kg BID for 21 days. *n* = 5 mice/group.

We also evaluated TNG908 in the LU99 *MTAP*-deleted cell derived xenograft (CDX) model representing NSCLC implanted subcutaneously in BALB/c nude mice. Following a study to determine the maximum tolerated dose (MTD) in BALB/c nude mice, oral administration of TNG908 at well-tolerated doses (30 or 120 mg/kg BID) in the LU99 MTAP-null NSCLC CDX model resulted in strong, dose-dependent antitumor activity including a 48% tumor regression at the 120 mg/kg BID dose level (Figure 11).

**Figure 12:**
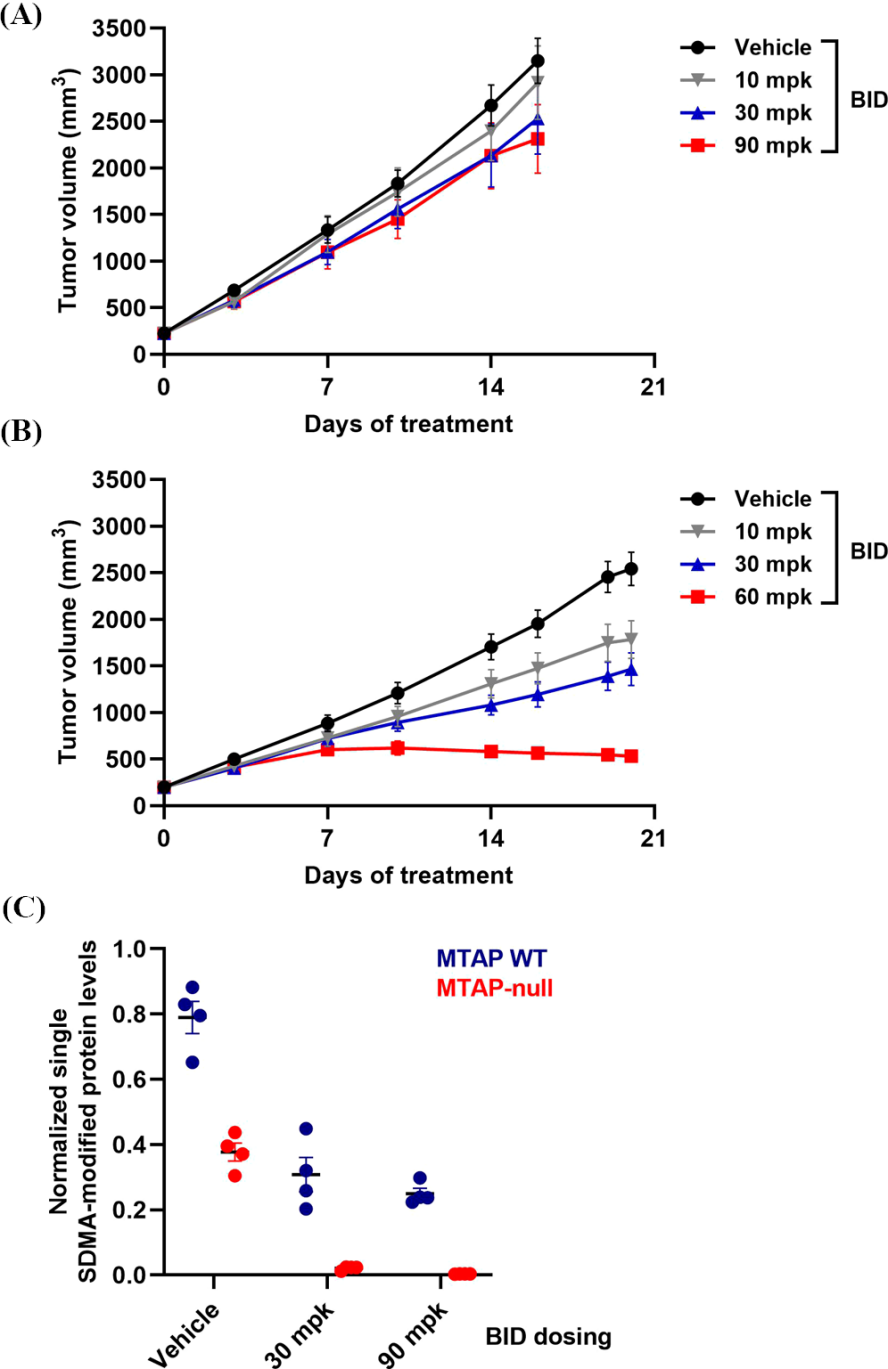
TNG908 PD and antitumor activity is selective for MTAP-null cells in vivo. (A) HCT116 MTAP WT tumor growth inhibition curves with TNG908 dosed at 10, 30, or 90 mg BID for 17 days. (B) HCT116 MTAP-null tumor growth inhibition curves with TNG908 dosed at 10, 30, or 90 mg BID for 21 days. (C) Terminal PD analyses from A and B demonstrating PRMT5 activity by quantification of a single SDMA-modified protein substrate 8 h post last dose. *n* = 4 tumors/time point/dose level. Data are presented as mean ± SEM for all data.

TNG908 was determined, in vitro, to be 15-fold selective for MTAP-null cells relative to isogenic MTAP WT cells (Table 6). The potency of TNG908 in the HCT116 MTAP-isogenic cell line pair is 0.130 μM in the MTAP-null cell line and 3.1 μM in the MTAP WT cell line. Therefore, TNG908 was determined to be ∼25× selective in the HCT116 MTAP-isogenic cell line pair. To further evaluate the selectivity of TNG908 in vivo, TNG908 was evaluated in the HCT116 *MTAP*-isogenic xenograft models. HCT116 is an endogenously MTAP WT model, so the isogenic pair was engineered by knocking out *MTAP* using CRISPR-based technology. Oral administration of TNG908 at well-tolerated doses (10, 30, or 90 mg/kg BID) resulted in dose-dependent antitumor activity in the MTAP-null xenograft model, whereas the MTAP WT model was largely spared (85% TGI vs 29% TGI, respectively) (Figure 12A and 12B). Terminal PD analyses further exemplified the selectivity and on-target behavior of TNG908 as PRMT5 activity was inhibited >90% in the MTAP-null xenograft models, while in the isogenic MTAP WT xenograft model, PRMT5 inhibition did not exceed the basal inhibition caused by MTAP deletion in the MTAP-null xenograft model (Figure 12C).

Collectively, these data demonstrate that TNG908 drives on-target, dose- and concentration-dependent antitumor activity selectively in *MTAP*-deleted xenograft models representing multiple tumor histologies, which is consistent with the 15-fold selectivity demonstrated in vitro. Indeed, single-agent TNG908 was able to drive tumor regressions in the LU99 *MTAP*-deleted xenograft model, which highlights the potential for TNG908 to drive meaningful clinical responses in *MTAP*-deleted tumors while maintaining a large therapeutic index.

## Further in Vitro Profiling of TNG908

TNG908 was profiled for off-target activity against a panel of 39 methyltransferases at 1 and 10 μM and in an in vitro toxicology safety panel (SAFETYscan E/IC_50_) of 78 known off-target binding and functional assays at 10 μM and showed no significant activity other than PRMT5-MEP50 (see Supporting Information). TNG908 also showed no activity in a hERG syncropatch assay (IC_50_ > 30 μM).

## Synthesis

**Scheme 1:**
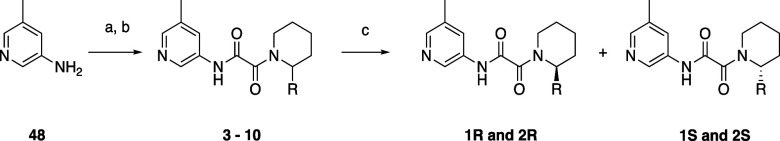
Synthesis of Compounds **1–10** Reagents and conditions: (a) DIPEA (1.5 equiv), ACN, 25 °C, 30 min; 2,2,2-trifluoroethyl-2-chloro-2-oxoacetate; (b) substituted piperidine (1.1 equiv), 25 °C, 1 h, 100 °C, 16 h; (c) chiral separation of enantiomers.

Initially, to explore the SAR of the 2 position of the piperidine, 2,2,2-trifluoroethyl-2-chloro-2-oxoacetate was reacted with aminopyridine **48** to generate a common intermediate used to prepare compounds **1**–**10** in parallel by reaction of the trifluoroethylester with 2-substituted piperidines (Scheme 1).

The functionalized pyridines of compounds **23** and **24** were prepared from 3-methyl-5-nitropyridine-2-amine **49** and 5-aminonicotinic acid **53**, respectively. **49** was Boc protected followed by nitro reduction with 10% Pd/C and subsequent conversion to **52** with 2,2,2-trifluoroethyl 2-chloro-2-oxoacetate. Acid **53** was converted to amide **54**, which was reacted with 2,2,2-trifluoroethyl 2-chloro-2-oxoacetate, and then hydrolyzed to acid **56**.

**Scheme 2:**
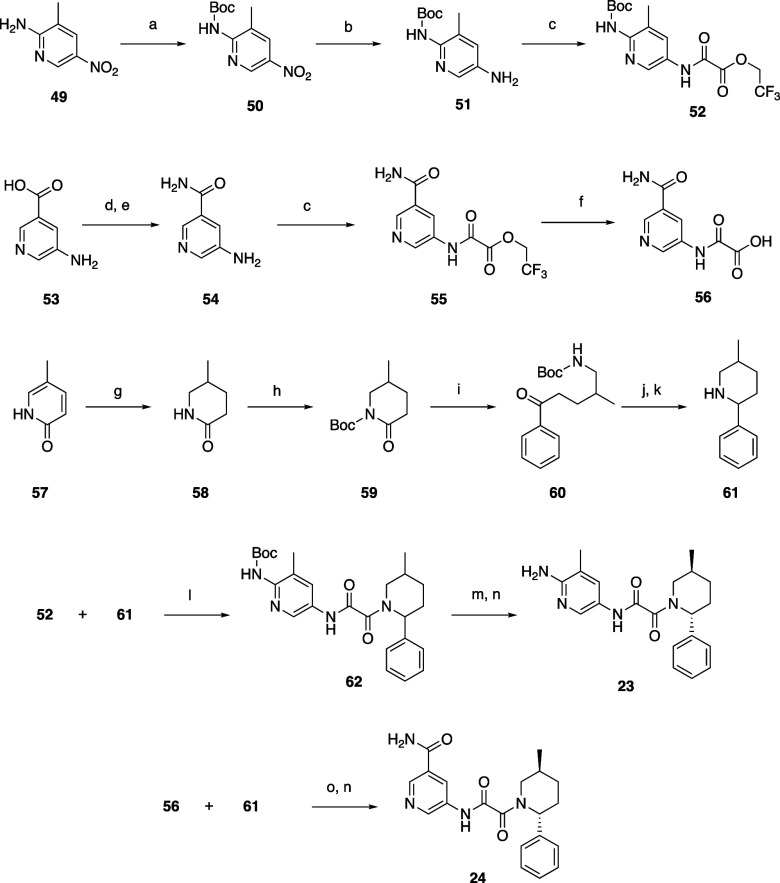
Synthesis of Compounds **23** and **24** Reagents and conditions: (a) Boc_2_O (1.05 equiv), NaH (1.05 equiv), DMF, 0 °C, 46% yield; (b) 10% Pd/C, H_2_, MeOH, 25 °C, 94% yield; (c) 2,2,2-trifluoroethyl 2-chloro-2-oxoacetate (1.15 equiv), DIPEA (1.5 equiv), ACN, 0–25 °C, 100% yield for **52**; (d) thionyl chloride (1.1 equiv), MeOH, reflux; (e) 25% NH_3_ (aq), 86% yield over 2 steps; (f) LiOH·H_2_O, THF, H_2_O, 91% yield; (g) 10% Pd/C, H_2_, MeOH, 50 psi, 80 °C; (h) Boc_2_O (2 equiv), TEA (3 equiv), DMAP (1 equiv), DCM, 20 °C, 90% yield over 2 steps; (i) 3 M PhMgBr in Et_2_O (1.2 equiv), THF, from −60 to 60 °C, 73% yield; (j) TFA (3 equiv), DCE, 20–50 °C; (k) NaBH_4_ (3 equiv), 0–25 °C, 86% yield over 2 steps; (l) n-BuLi (3.3 equiv), THF, from −78 to 25 °C, 94% yield; (m) 4 M HCl in dioxane, DCM; (n) SFC separation of isomers, 35% yield over 2 steps for **23**; (o) HATU (1.1 equiv), DMSO, 25 °C, 35% yield after separation.

One method to prepare amine **61** was to reduce pyridin-2(1*H*)-one **57** with 10% Pd/C at 50 atm of H_2_. The racemic mixture was then Boc protected followed by ring opening with phenyl Grignard reagent, BOC deprotection, and dehydrative cyclization of the amine onto the ketone. The resultant imine was selectively reduced with sodium borohydride to give a mixture of predominantly trans isomers of piperidine **61**. **61** was coupled to oxamate **52** and **56** to form the desired oxamides **23** and **24** after deprotection of **62** with TFA (Scheme 2). The borohydride reduction typically gave a 9:1 ratio of trans:cis isomers, and the isomers were readily separated by SFC or chiral HPLC. The desired trans isomer was determined in early analogs by 2D NMR, small molecule crystal structures, and protein–ligand complexes. The stereochemistry of subsequent analogs was assigned arbitrarily with key analogs undergoing absolute confirmation. Eventually, a chiral synthesis of lactam **59** was developed, which provided only the desired trans isomer, which was easily purified from the minor amount of undesired cis isomer formed during the imine reduction.

**Scheme 3:**
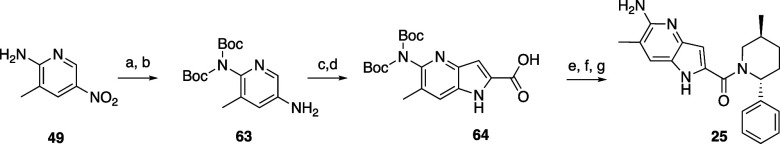
Synthesis of Compound **25** Reagents and conditions: (a) Boc_2_O (2 equiv), DMAP (1 equiv), DCM, 20 °C; (b) 10% Pd/C, H_2_, MeOH, 20 °C, 95% yield over 2 steps; (c) NIS (1.08 equiv), AcOH, 20 °C; (d) 2-oxopropanoic acid, Pd_2_(OAc)_2_, PPh_3_, TEA, DMF, 100 °C, 14% yield over 2 steps; (e) **61** (1.2 equiv), HATU (1.2 equiv), DIPEA (3 equiv), DCM, 20 °C; (f) TFA (10 equiv), DCM, 20 °C; (g) SFC chiral separation of isomers, 9% yield over 3 steps.

To synthesize **25**, an example of an oxamide isostere, **49** was bis-Boc protected and the nitro group reduced to the amine. Regioselective iodination followed by palladium-catalyzed cyclization with 2-oxopropanoic acid gave pyrrolopyridine acid **64**, which was coupled to **61** with HATU and deprotected, and the racemate was separated by SFC to yield **25** (Scheme 3).

**Scheme 4:**
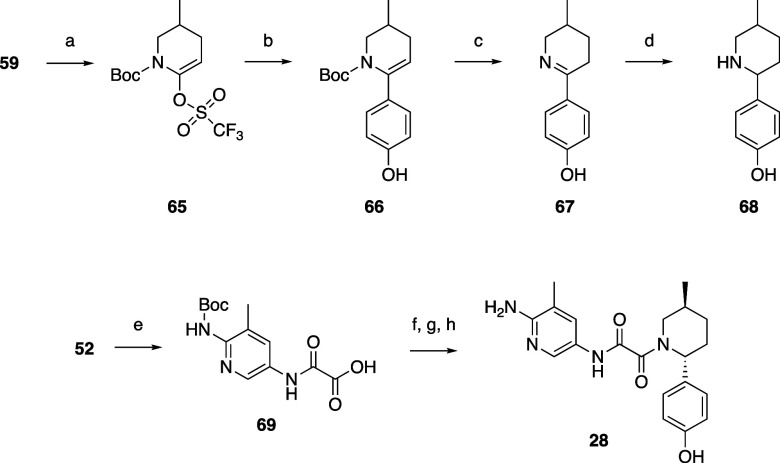
Representative Example of the Synthesis of Compounds **28–44** Reagents and conditions: (a) 1,1,1-trifluoro-*N*-phenyl-*N*-(trifluoromethylsulfonyl)methanesulfonamide (1.25 equiv), LHMDS (1 equiv), THF, from −78 to −25 °C, 95% yield; (b) (4-hydroxyphenyl)boronic acid (1.25 equiv), Na_2_CO_3_ (3 equiv), Pd(dppf)Cl_2_·DCM (0.05 equiv), dioxane, H_2_O, 90 °C, 45% yield; (c) TFA (2.3 equiv), DCM, 25 °C; (d) NaBH_4_ (1.18 equiv), MeOH, 0 °C; (e) LiOH·H_2_O (2 equiv), THF, MeOH, H_2_O, 5 °C, 98% yield; (f) **68** (1 equiv), HATU (1 equiv), TEA (10 equiv), DMF, 25 °C; (g) dioxane, H_2_O, 95 °C; (h) chiral HPLC separation of isomers, 2% over 4 steps.

**Scheme 5:**
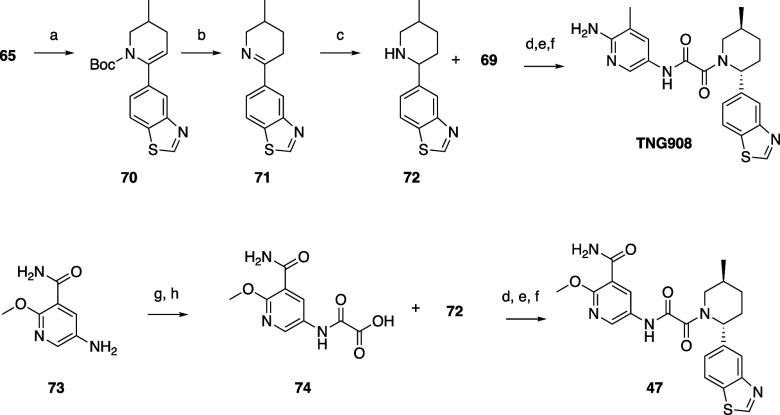
Synthesis of TNG908 and Compounds **45–47** Reagents and conditions: (a) 5-(4,4,5,5-tetramethyl-1,3,2-dioxaborolan-2-yl)-1,3-benzothiazole (1.25 equiv), Na_2_CO_3_ (3 equiv), Pd(dppf)Cl_2_·DCM (0.05 equiv), dioxane, H_2_O, 80 °C, 80% yield; (b) TFA (16 equiv), DCM, 25 °C, 100% yield; (c) NaBH_4_ (1.5 equiv), MeOH, 0 °C, 99% yield; (d) HATU (1 equiv), TEA (6 equiv), DMF, 25 °C; (e) 4 M HCl in dioxane, 25 °C, 59% yield over 2 steps for TNG908; (f) chiral HPLC separation of enantiomers, 48% yield for TNG908; (g) 2,2,2-trifluoroethyl 2-chloro-2-oxoacetate (1.05 equiv), TEA (1 equiv), THF, 0–25 °C; (h) LiOH·H_2_O (2 equiv), THF, MeOH, 25 °C, 65% yield over 2 steps.

Compounds **28**–**44** were prepared via coupling of oxamic acids **69** (prepared by hydrolysis of oxamate **52**) or **56** with the appropriate piperidine using HATU and Hünig’s base in DMF (Scheme 4). The piperidines were prepared by Suzuki coupling of boronates or boronic acids with Boc-protected triflate **65** prepared from **59**. Deprotection with TFA gave imines which could be stereoselectively reduced with sodium borohydride to give a mixture of predominantly trans isomers (typically 9:1 ratio of trans:cis) of the piperidine, as in **23** and **24**. As before, the cis isomers could be removed when separating the trans enantiomers by SFC or chiral HPLC before or after preparing the target oxamide by coupling to the appropriate oxamic acid (**56** or **69**) with deprotection as necessary. TNG908 (Scheme 5) was prepared similarly. Compounds **45–47** were also prepared similarly from 5-amino-2-methoxynicotinamide **73**, which was commercially available from Enamine. **73** was reacted with 2,2,2-trifluoroethyl 2-chloro-2-oxoacetate and hydrolyzed to **74** with lithium hydroxide, which could be used to prepare the desired oxamide targets.

## Conclusions

In summary, a series of compounds was discovered that inhibits PRMT5 cooperatively with MTA and includes compounds that are potent and selective in MTAP-null cells vs MTAP WT cells. The series was initiated from a high-throughput biochemical screening hit and was guided by both ligand- and structure-based drug design, leading to TNG908, which has a Tanimoto coefficient of 0.3 relative to the original hit. Potency was increased by nearly 2000-fold with the addition of only 83 Da, no additional rotatable bonds, and a reduction in lipophilicity as measured in LogD (2.4 vs 2.8) while maintaining high permeability, low efflux, and a high *K*_p,uu,CSF_ = 0.9. TNG908 has low to moderate clearance and moderate to high bioavailability across species, strong efficacy across a panel of xenograft models including those representing glioblastoma and nonsmall cell lung cancer, and selective efficacy in vivo in a colorectal cancer model. TNG908 was nominated as a development candidate and is currently in a Phase I/II clinical study (NCT05275478).

## Experimental Section

### General Procedures

All chemicals were provided by Enamine Ltd., WuXi AppTec, or other commercial suppliers and used as received unless otherwise indicated. All solvents were treated according to standard methods. All reactions were monitored by LC-MS analysis using Agilent 1260 LC/MSD instruments, with an Agilent Poroshell 120 SB-C18 4.6 × 30 mm 2.7 μm column, column temperature = 60 °C, mobile phase = (A) water (0.1% formic acid) and (B) acetonitrile (0.1% formic acid), flow rate = 1.5 mL/min, gradient = 0.01 min, 1% B; 5.00 min, 100% B; 5.99 min, 100% B, MS ionization mode = electrospray ionization (ESI), MS scan range = 83–1000 *m*/*z*, UV detection = 215, 254, and 280 nm unless otherwise specified. Thin-layer chromatography (TLC) with precoated silica gel GF254 (0.2 mm) was used, and the results were visualized using either UV light or KMnO_4_ stain. Proton nuclear magnetic resonance (^1^H NMR) spectra were recorded at 400, 500, or 600 MHz on Varian or Bruker instrumentation; chemical shifts were calibrated using residual nondeuterated solvents CHCl_3_ (δ = 7.26 ppm), DMSO (δ = 2.50 ppm), or MeOH (δ = 3.31 ppm) and expressed in δ ppm. Coupling constants (*J*), when given, are reported in hertz (Hz). Multiplicities are reported using the following abbreviations: s = singlet, d = doublet, dd = doublet of doublets, t = triplet, q = multiplet (range of multiplets is given), br = broad signal, dt = doublet of triplets. ^19^F NMR spectra were recorded at 376 MHz (Varian). ^13^C NMR spectra were recorded at 101, 126, or 151 MHz (Varian). ^13^C NMR chemical shifts are reported relative to the central CHCl_3_ (δ = 77.16 ppm), DMSO (δ = 39.52 ppm), or MeOH (δ = 49.00 ppm), and chemical shifts are reported in parts per million (ppm). All final compounds were purified by reverse-phase high-performance liquid chromatography (HPLC), supercritical fluid chromatography (SFC), or silica gel chromatography (100–200 mesh). HPLC was done with an Agilent 1260 HPLC instrument (Agilent Technologies, Germany) equipped with a G7161A Preparative Binary Pump, a G7157A Prep Autosampler, a G7115A DAD WR, and a G7159B Preparative Fraction Collector. The Open Lab CDS software (version C.01.10) was used for instrument control, data acquisition, and data handling. SFC was done with a Waters 100q Prep SFC System. Chiral HPLC analytical analysis was done with an Agilent 1200 HPLC instrument (Agilent Technologies, Germany) equipped with a G1379B degasser, a G1312A Binary Pump, a G1329A ALS autosampler, and a G1315A diode array detector. Chiral SFC analytical analysis was done with an Agilent 1260 SFC instrument (Agilent Technologies, Germany) equipped with a G1379B degasser, a G1312B Binary Pump, a G1313A ALS autosampler, a G1316A thermostated column compartment, a G1315D diode array detector, and an Aurora SFC system. Melting points were taken using OptiMelt Automated Melting Point System Digital Image Processing Technology SRS Stanford Research Systems, 2 °C/min (5 °C/min at high melting point). Optical rotation was measured with a polarimeter from Anton Paar GmbH MCP 300 (accuracy ±0.003°) used to measure the angle of optical rotation. Standard conditions for analysis: solution concentration 0.5 g/100 mL (methanol solvent), wavelength 589 nm, and temperature 21 °C. All oxamides exist as rotamers in the ^1^H NMR spectra. All compounds are >95% pure by HPLC.

#### *tert*-Butyl (3-Methyl-5-nitropyridin-2-yl)carbamate (**50**)

To a solution of 3-methyl-5-nitropyridin-2-amine (**49**) (60 g, 391.8 mmol, 1 equiv) in DMF (525 mL), sodium hydride, 60% dispersion in mineral oil (16.5 g, 412.88 mmol, 1.05 equiv), was added portionwise at 0 °C. The resulting mixture was stirred for 0.5 h, and solution of di-*tert*-butyl dicarbonate (89.8 g, 411.4 mmol, 1 equiv) in DMF (75 mL) was added dropwise. The resulting mixture was stirred at 25 °C for 18 h. The mixture was quenched with water (1000 mL); the precipitate was filtered off and dried in vacuo to obtain 100 g of crude product, which was purified by column chromatography (CHCl_3_:methyl *tert*-butyl ether as the eluent) to obtain **50** as a white powder (46 g, 181.64 mmol, 46% yield). ^1^H NMR (400 MHz, DMSO-*d*_6_): δ 1.49 (s, 9H), 2.32 (s, 3H), 8.37 (s, 1H), 8.97 (s, 1H), 9.59 (s, 1H). LCMS (ESI): [M – CH_2_C(CH_3_) + H]^+^*m*/*z* calcd 197.04; found 198.2. *R*_t_ = 1.272 min.

#### *tert*-Butyl (5-Amino-3-methylpyridin-2-yl)carbamate (**51**)

To a solution of **50** (46 g, 181.64 mmol, 1 equiv) in methanol (600 mL) was added 10% palladium on activated carbon (4.6 g, 43.22 mmol, 0.24 equiv). The resulting mixture was stirred under H_2_ atmosphere for 24 h. The catalyst was filtered, and the solvent was removed in vacuo. The residue was dissolved in DCM (500 mL), dried over sodium sulfate, and evaporated in vacuo to obtain **51** as a white solid (38 g, 170.2 mmol, 94% yield). ^1^H NMR (400 MHz, DMSO-*d*_6_): δ 1.40 (s, 9H), 2.03 (s, 3H), 3.30 (brs, 2H), 6.80 (s, 1H), 7.54 (s, 1H), 8.52 (s, 1H). LCMS (ESI): [M + H]^+^*m*/*z* calcd 223.2; found 224.2. *R*_t_ = 0.67 min.

#### 2,2,2-Trifluoroethyl 2-((6-((*tert*-Butoxycarbonyl)amino)-5-methylpyridin-3-yl)amino-2-oxoacetate (**52**)

To a solution of **51** (17.6 g, 78.83 mmol, 1 equiv) and DIPEA (20.60 mL, 118.24 mmol, 1.5 equiv) in ACN (250 mL) was added 2,2,2-trifluoroethyl 2-chloro-2-oxoacetate (17.27 g, 90.65 mmol, 1.15 equiv) dropwise at 0 °C under argon. The reaction mixture was then stirred for 24 h at 20 °C. The solvent was evaporated in vacuo, and the residue was diluted with H_2_O (575 mL). The precipitate was filtered off, washed with water, and dried in vacuo to provide **52** (30 g, 79.5 mmol, 100% yield), which was used without further purification. ^1^H NMR (DMSO-*d*_6_, 400 MHz): δ 1.42 (s, 9H), 2.15 (s, 3H), 4.96 (q, 2H), 7.93 (s, 1H), 8.49 (s, 1H), 9.03 (s, 1H), 11.06 (s, 1H). LCMS (ESI): [M – CH_2_C(CH_3_) + H]^+^*m*/*z* calcd 321.06; found 322.0. *R*_t_ = 1.274 min.

#### 2-((6-((*tert*-Butoxycarbonyl)amino)-5-methylpyridin-3-yl)amino)-2-oxoacetic Acid (**69**)

A mixture of **52** (30 g, 79.51 mmol, 1 equiv) and lithium hydroxide monohydrate (6.67 g, 159.02 mmol, 2 equiv) in THF (120 mL), MeOH (120 mL), and H_2_O (120 mL) and was stirred at 5 °C. After 2 h, the volatile organic solvents were removed under reduced pressure. The residue was acidified with sodium hydrogen sulfate monohydrate (21.96 g, 159.02 mmol, 2 equiv) to pH 5, and the precipitate was filtered off, washed with water, and dried in vacuo to provide **69** (23 g, 77.89 mmol, 98% yield). ^1^H NMR (DMSO-*d*_6_, 400 MHz): δ 1.44 (s, 9H), 2.16 (s, 3H), 7.97 (s, 1H), 8.51 (s, 1H), 8.98 (s, 1H), 10.70 (s, 1H). LCMS(ESI): [M – CH_2_C(CH_3_) + H]^+^*m*/*z*. calcd 295.29; found 240.0. *R*_t_ = 0.829 min.

#### 5-Methylpiperidin-2-one (**58**)

To a solution of 5-methyl-1*H*-pyridin-2-one (**57**) (25 g, 0.229 mol, 1 equiv) in MeOH (200 mL) was added 10% palladium on carbon (4 g, 10 wt % of Pd with 50 wt % of H_2_O) under nitrogen atmosphere. The suspension was degassed and purged with H_2_ 3 times. The mixture was stirred under hydrogen (50 psi) at 80 °C for 24 h. The mixture was filtered, and the filtrate was concentrated under reduced pressure to give **58** (26 g, crude) as a colorless oil, which was directly used without further purification.

#### *tert*-Butyl 5-Methyl-2-oxopiperidine-1-carboxylate (**59**)

To a solution of **58** (26 g, 0.230 mol, 1 equiv) in DCM (100 mL) were added TEA (96 mL, 0.689 mol, 3 equiv) and DMAP (28 g, 0.229 mol, 1 equiv); then, di-*tert*-butyl dicarbonate (106 mL, 0.461 mol, 2 equiv) was added slowly. The mixture was stirred at 20 °C for 12 h, and the resulting mixture was quenched by addition of H_2_O (100 mL). The organic layer was separated, and the aqueous phase was extracted with DCM (100 mL × 2). The combined organic layer was washed with saturated NH_4_Cl aqueous solution (500 mL × 3), dried over anhydrous Na_2_SO_4_, filtered, and concentrated under reduced pressure to give a crude product, which was purified by flash chromatography (ISCO; 220 g AgelaFlash silica flash column, petroleum Et_2_O/EtOAc with EtOAc from 0 to 10%, 100 mL/min) to afford **59** (44 g, 90% yield) as a colorless oil. ^1^H NMR (400 MHz, methanol-*d*_4_) δ 3.79 (ddd, *J* = 12.5, 4.8, 1.8 Hz, 1H), 3.16 (dd, *J* = 12.4, 10.4 Hz, 1H), 2.42–2.57 (m, 2H), 1.83–2.01 (m, 2H), 1.51 (s, 9H), 1.46 (dd, *J* = 6.7, 3.6 Hz, 1H), 1.04 (d, *J* = 6.5 Hz, 3H). LCMS (ESI) [M + H – Bu]^+^*m*/*z*: calcd 158.1; found 157.8.

#### *tert*-Butyl 3-Methyl-6-(((trifluoromethyl)sulfonyl)oxy)-3,4-dihydropyridine-1(2*H*)-carboxylate (**65**)

LHMDS (480.56 g, 574.38 mmol, 20% purity, 1.25 equiv) was added dropwise under argon at −78 °C to a solution of **59** (98 g, 459.51 mmol, 1 equiv) in THF (500 mL). The resulting solution was stirred at −78 °C for 1.5 h; then, 1,1,1-trifluoro-*N*-phenyl-*N*-(trifluoromethylsulfonyl)methanesulfonamide (188.78 g, 528.43 mmol, 1.15 equiv) was added. The reaction mixture was allowed to warm to 20 °C, stirred for 12 h, then diluted with H_2_O (300 mL) and MTBE (700 mL). The organic layer was separated; the aqueous layer was extracted with MTBE (300 mL). The combined organic extracts were washed with 10% aqueous sodium hydroxide solution (3 × 300 mL), dried over potassium carbonate, and evaporated in vacuo. The residue was diluted with a hexane/MTBE mixture and stirred for 0.5 h. The resulting cloudy solution was decanted from an oily residue, filtered through a short pad of silica gel, and evaporated in vacuo to afford **65** (150 g, 434.36 mmol, 95% yield) as a light-yellow oil, which was used as is.

#### *tert*-Butyl 6-(Benzo[*d*]thiazol-5-yl)-3-methyl-3,4-dihydropyridine-1(2*H*)-carboxylate (**70**)

A mixture of **65** (7.72 g, 22.36 mmol, 1 equiv), 5-(4,4,5,5-tetramethyl-1,3,2-dioxaborolan-2-yl)-1,3-benzothiazole (7.3 g, 27.95 mmol, 1.25 equiv), [1,1′-bis(diphenylphosphino)ferrocene]dichloropalladium(II) complex with DCM (913 mg, 1.12 mmol, 0.05 equiv), and sodium carbonate (7.11 g, 67.09 mmol, 3 equiv) in dioxane (120 mL) and water (40 mL) was stirred at 80 °C under argon atmosphere for 18 h. After cooling to room temperature, the reaction mixture was filtered. The filter cake was washed with dioxane (500 mL) and discarded. The filtrate was concentrated under reduced pressure, and the residue was purified by silica gel flash chromatography eluting with a 0–100% MTBE–hexane gradient to give **70** (5.9 g, 17.85 mmol, 80% yield). ^1^H NMR (DMSO-*d*_6_, 500 MHz): δ 0.95–1.03 (m, 12H), 1.86 (m, 1H), 1.90 (s, 1H), 2.50 (m, 1H), 3.0 (t, 1H), 3.97 (d, 1H), 5.41 (s, 1H). 7.37 (d, 1H), 7.78 (s, 1H), 7.99 (d, 1H), 9.27 (s, 1H). LCMS (ESI): [M + H]^+^*m*/*z* calcd 330.2; found 331.2. *R*_t_ = 1.435 min.

#### 5-(5-Methyl-3,4,5,6-tetrahydropyridin-2-yl)benzo[*d*]thiazole (**71**)

**70** (5.9 g, 17.85 mmol, 1 equiv) was stirred in TFA (22 mL) at 20 °C for 1 h and then evaporated in vacuo. Crushed ice (10 g) was added to the residue, and the pH was adjusted to pH 8 with a 10% aqueous solution of sodium hydroxide. The resulting mixture was extracted with EtOAc (2 × 30 mL). The combined organic extracts were dried over sodium sulfate and evaporated in vacuo to afford **71** (4.1 g, 17.80 mmol, 100% yield) as a yellow solid, which was used directly in the next step. ^1^H NMR (DMSO-*d*_6_, 500 MHz): δ 0.95 (m, 3H), 1.35 (m, 1H), 1.65 (m, 1H), 1.89 (m, 1H), 2.67 (m, 1H), 2.87 (d, 1H), 3.19 (t, 1H). 3.95 (d, 1H), 8.02 (d, 1H), 8.14 (d, 1H), 8.43 (s, 1H), 9.41 (s, 1H). LCMS (ESI): [M + H]^+^*m*/*z* calcd 230.1; found 231.2. *R*_t_ = 0.828 min.

#### *rac*-5-((2*R*,5*S*)-5-Methylpiperidin-2-yl)benzo[*d*]thiazole (**72**)

Sodium borohydride (1.01 g, 26.70 mmol, 1.5 equiv) was added to a stirred solution of **71** (4.1 g, 17.80 mmol, 1 equiv) in MeOH (90 mL) at 0 °C. The resulting mixture was stirred for 1 h and then evaporated in vacuo. The residue was diluted with H_2_O (50 mL) and extracted with DCM (2 × 75 mL). The combined organic extracts were dried over sodium sulfate and evaporated in vacuo to afford **72** (4.1 g, 17.65 mmol, 99% yield) as a yellow oil, which was used directly in the next step. ^1^H NMR (DMSO-*d*_6_, 400 MHz): δ 0.82(d, 3H), 1.05 (m, 1H), 1.34 (m, 1H), 1.52 (m, 1H), 1.75 (m, 2H), 2.26 (t, 1H), 3.00 (d, 1H). 3.61 (d, 1H), 7.46 (d, 1H), 8.03 (m, 2H), 9.31 (s, 1H). LCMS (ESI): [M + H]^+^*m*/*z* calcd 232.1; found 233.0. *R*_t_ = 0.691 min.

#### *N*-(6-Amino-5-methylpyridin-3-yl)-2-((2*R*,5*S*)-2-(benzo[*d*]thiazol-5-yl)-5-methylpiperidin-1-yl)-2-oxoacetamide TNG908

HATU (491 mg, 1.29 mmol, 1 equiv) was added portionwise at room temperature to a suspension of **69** (381 mg, 1.29 mmol, 1 equiv), **72** (300 mg, 1.29 mmol, 1 equiv), and TEA (1.08 mL, 7.75 mmol, 6 equiv) in DMF (10 mL). The clear solution was stirred at 25 °C for 18 h, and the solvents were evaporated in vacuo. The residue was dissolved in EtOAc (100 mL), washed with H_2_O (3 × 50 mL), and evaporated in vacuo to give *tert*-butyl *N*-[5-[[2-[2-(1,3-benzothiazol-5-yl)-5-methyl-1-piperidyl]-2-oxo-acetyl]amino]-3-methyl-2-pyridyl]carbamate (700 mg, crude). ^1^H NMR (DMSO-*d*_6_, 400 MHz): δ 1.01 (d, 3H), 1.39 (m, 13H), 2.09 (m, 8H), 5.71 (m, 1H), 7.43 (m, 2H), 8.12 (m, 1H), 8.43 (s, 1H), 9.03 (s, 1H), 9.38 (m, 1H), 11.00 (s, 1H). LCMS(ESI): [M + H]^+^*m*/*z* calcd 509.2; found 510.2. *R*_t_ = 1.319 min. A 4.0 M hydrogen chloride solution in dioxane (3.41 mL, 13.74 mmol, 10 equiv) was carefully added at room temperature to a solution of the crude material (700 mg, 1.37 mmol, 1 equiv) in DCM (10 mL). The reaction mixture was then stirred for 12 h at room temperature, and the solvents were evaporated in vacuo. The residue was purified by RP-HPLC (column: YMC Triart C18 100 × 20 mm, 5 μm; 40–40–90% 0–1–5 min 0.1% NH_3_–methanol as mobile phase) to give racemic *N*-(6-amino-5-methyl-3-pyridyl)-2-[2-(1,3-benzothiazol-5-yl)-5-methyl-1-piperidyl]-2-oxo-acetamide (331 mg, 0.808 mmol, 59% yield). LCMS(ESI): [M + H]^+^*m*/*z* calcd 409.2; found 410.2. *R*_t_ = 2.176 min. The enantiomers were separated by chiral HPLC (column: IC II, hexane–IPA–MeOH, 50–25–25, 12 mL/min as mobile phase) to give two individual enantiomers *N*-(6-amino-5-methyl-3-pyridyl)-2-[(2*S*,5*R*)-2-(1,3-benzothiazol-5-yl)-5-methyl-1-piperidyl]-2-oxo-acetamide (161 mg, 0.393 mmol, 49% yield) [α]_D_^21^ = −176.7° (*c* = 0.1 g/100 mL, EtOH) and TNG908 (160 mg, 0.390 mmol, 48% yield) [α]_D_^21^ = +191.5° (*c* = 0.1 g/100 mL, EtOH). RT (IC, hexane–IPA–MeOH, 50–25–25, 0.6 mL/min) = 47.098 min. ^1^H NMR (600 MHz, DMSO-*d*_6_) δ 0.98–1.06 (m, 3H), 1.30–1.42 (m, 1H), 1.66–1.75 (m, 1H), 1.82–1.91 (m, 1H), 1.95–2.04 (m, 3H), 2.06–2.23 (m, 1H), 2.26–2.35 (m, 1H), 2.76–3.27 (m, 1H), 3.38–4.06 (m, 1H), 5.26–5.60 (m, 1H), 5.60–5.76 (m, 2H), 7.39–7.46 (m, 1H), 7.50 (s, 1H), 7.92–8.01 (m, 1H), 8.01–8.06 (m, 1H), 8.13–8.20 (m, 1H), 9.37–9.43 (m, 1H), 10.50–10.70 (m, 1H). LCMS (ESI): [M + H]^+^*m*/*z* calcd 409.2; found 410.0. *R*_t_ = 1.992 min. Melting point (instrument CAS-TJ-MPA-02) 167.3 °C. HRMS (ESI, + vw ion): *m*/*z* calcd for C_21_H_24_N_5_O_2_S^+^ [M + H^+^] 410.1645; found 410.1652.

### Biochemical Fluorescence Anisotropy Peptide Displacement Assay

A fluorescence anisotropy (FA) assay was established to measure binding of C-terminal 5′-TAMRA-labeled histone H4 peptide (1–21) with PRMT5/MEP50. The test compound competes with the peptide to bind to PRMT5/MEP50 protein. Assay buffer: 30 mM Bicine (pH 8.0), 150 mM NaCl, 1.5 mM DTT, 0.003% Tween-20. The two peptides utilized for these studies were Me_0_: Ac-SGRGKGGKGLGKGGAKRHRKV-K(5-TAMRA)-NH2 and Me_2_: Ac-SGR(Sym Me_2_)GKGGKGLGKGGAKRHRKV-K(5-TAMRA)-NH2. Me_0_ peptide is not methylated and was used to determine the compound potency in the absence of cofactor and in the presence of 50 μM 5′-methylthioadenosine (MTA). Me_2_ peptide is symmetrically methylated at Arginine 3 and was used to determine the compound potency in the presence of 50 μM *S*-adenosyl-l-methionine (SAM). Inhibitor potency was assessed at equilibrium by measuring the dose-dependent displacement of a fixed concentration of the peptide from PRMT5/MEP50. Following incubation at RT for 30 min, the plate was read on an Envision plate reader. For data analysis, fluorescence anisotropy (FA) detected equals 1000 × (*S* – *G* × *P*)/(*S* + *G* × 2 × *P*), where *S* = detector 2 or channel 2 signal, *P* = detector 1 or channel 1 signal, *G* = *G* factor. Fluorescence anisotropy is normalized to percent inhibition using percent inhibition = (signal – Min_AVG_)/(Max_AVG_ – Min_AVG_) × 100, where Min_AVG_ = the average value of the min value and Max_AVG_ = the average value of the max value. Curves are fit by XL-Fit as percent inhibition vs log [compound concentration] using a 4-parameter logistic model 205 *y* = *A* + ((*B* – *A*)/(1 + ((*C*/*x*)∧*D*))) with fixed 0% and 100% inhibition limits to calculate the IC_50_. *A*: bottom = 0%. *B*: top = 100%. *C*: relative IC_50_. *D*: Hill slope. The apparent *K*_i_ values of TNG908 were calculated using the Cheng–Prusoff equation for a competitive inhibitor.

### HAP1 MTAP WT and MTAP-null In-Cell Western Assay

A HAP1 *MTAP*-isogenic cell line pair was acquired from Horizon Discovery (HZGHC004894c005) and maintained in DMEM (high glucose) + 10% FBS in a humidified, 10% CO_2_ tissue culture incubator. The SAM-cooperative PRMT5 inhibitor, GSK3326595, was sourced from Selleck Chemicals and maintained as a 10 mM DMSO stock. All test compounds were maintained as 10 mM DMSO stocks.

On day 0, MTAP WT or MTAP-null cells are seeded in a 384-well plate and incubated in a humidified, 5% CO_2_ tissue culture incubator for 16–24 h. On day 1, the test compounds are dispensed to wells at defined concentrations using a Tecan D300e digital dispenser (*n* = 4), and the volume of DMSO is normalized to the highest class volume. Each plate includes wells dosed with defined concentrations of GSK3326595 as a plate control. The compounds are incubated with cells for 24 h in a humidified, 5% CO_2_ tissue culture incubator.

On day 2, the compound-treated cells are fixed with a final concentration of 4% formaldehyde. The cells are then washed/permeabilized with 1 × PBS + 0.1% Triton X-100 and then blocked with 5% goat serum/1 × TBS. The fixed cells are then incubated overnight at 4 °C with a primary SDMA antibody cocktail (Cell Signaling 13222).

On day 3, the cells are washed with 1 × PBS + 0.1% Triton X-100 and then incubated at room temperature for 1 h with a NIR fluorescent secondary antibody cocktail that also contains DRAQ5 (LiCor 926-32211 and VWR 10761-508). The cells are washed with 1 × PBS + 0.1% Triton X-100 and then washed again with ddH_2_O. The plates are then imaged using a NIR fluorescent imager (LiCor Odyssey).

For data analysis, the SDMA signal is normalized to the DRAQ5 signal. Assay background is determined by the signal from wells treated with 1 μM GSK3326595 and subtracted from every well. The data are plotted as percent of the DMSO control wells for the MTAP WT and the MTAP-null cell lines independently and fitted to the 4-parameter logistic (4-PL) Hill equation with maximal effect constrained to 0. The fit was performed using GraphPad Prism or the default IC_50_ fitting procedure in Dotmatics Studies 5.4 as part of a customized data analysis protocol.

### HAP1 MTAP WT and MTAP-null Viability Assay

A HAP1 *MTAP*-isogenic cell line pair was acquired from Horizon Discovery (HZGHC004894c005) and maintained in DMEM (high glucose) + 10% FBS in a humidified 5% or 10% CO_2_ tissue culture incubator. All test compounds are maintained as 10 mM DMSO stocks.

On day 0, MTAP WT and MTAP-null cells are seeded in a 96-well plate and incubated in a humidified 5% or 10% CO_2_ tissue culture incubator for 16–24 h. On day 1, the test compounds are dispensed to wells at defined concentrations using a Tecan D300e digital dispenser (*n* = 3), and the volume of DMSO is normalized to the highest class volume (0.2%). The compound-treated plates are incubated for 7 days in a humidified 5% or 10% CO_2_ tissue culture incubator.

On day 7, the plates are removed from the tissue culture incubator and allowed to equilibrate to room temperature. Then, either a 1/2 volume CellTiter-Glo Luminescent Cell Viability Assay reagent (Promega G7572) is added to each well or the media is removed from every well and a 1:3 dilution of CellTiter-Glo 2.0 Cell Viability Assay reagent (Promega G9241) in 1 × PBS is added. Ten minutes after addition, the luminescent signal is detected by an Envision plate reader. The data are plotted as percent of the DMSO control wells for the MTAP WT and the MTAP-null cell lines independently and fitted to the 4-parameter logistic (4-PL) Hill equation with maximal effect constrained to 0. The fit was performed using GraphPad Prism or the default IC_50_ fitting procedure in Dotmatics Studies 5.4 as part of a customized data analysis protocol.

### In Vivo Pharmacology

All protocols were approved by the Institutional Animal Care and Use Committee at Pharmaron (Beijing, China) following the guidance of the Association of Assessment and Accreditation of Laboratory Animal Care.

After 7 days of acclimatization, 10 million LN-18, LU99, HCT116 MTAP WT, or HCT116 MTAP-null tumor cells were injected subcutaneously into the right flank of each animal. Once the tumors reached 200–300 mm^3^ in size, animals were randomized to treatment groups. TNG908 was administered by oral gavage twice daily in a 5% DMA/20% Captisol solution. Tumor volume was measured using calipers and calculated as (length × width × width)/2.

For data analysis, tumor growth inhibition was calculated using the following equation: percent TGI = [1 – (mean treated TV_final_ – mean treated TV_initial_)/(mean vehicle TV_final_ – mean vehicle TV_initial_)] × 100. Tumor regression was calculated as follows: percent tumor regression= [mean TV_final_ – mean TV_initial_] × 100.

### Western Blotting

Protein lysates were generated by lysis of frozen tumor tissue using RIPA buffer. Samples were normalized by protein concentration using a Pierce Rapid Gold BCA Protein Assay Kit (A53225). SDS-PAGE was run using Invitrogen NuPAGE 4–12% Bis-Tris Midi Protein Gels (WG1402BOX). Antibodies SDMA (CST#13222), ACTB (CST#3700) were used at 1:1000 dilution.

## Supporting Information Available

General experimental and chemical procedures; NMR spectra and HPLC/LCMS traces of final compounds; analytical data for TNG908, small molecule crystal structure of TNG908, biochemical fluorescence anisotropy peptide displacement assay, double titration *K*_i_ measurement of TNG908, biochemical methyltransferase FlashPlate assay, reversibility measurement using Zeba spin column activity recovery assay, methyltransferase and Eurofins SAFETYscan panel for TNG908, MDR1-MDCKII assay, human liver microsomes assay, kinetic solubility assay, hERG assay, in vivo PK, cocrystal structure determination, small molecule binding by SPR (PDF)
Molecular formula strings (CSV)

## Abbreviations Used

BID: bis in die (twice daily)
CNS: central nervous system
hERG: human Ether-a-go-go-Related Gene
IV: intravenous
MDCK: Madin–Darby Canine Kidney cell
MDR1: multidrug resistance 1
MTA: methylthioadenosine
MTAP: methylthioadenosine phosphorylase
PO: per os (oral)
PRMT5: protein arginine methyltransferase 5
PSA: polar surface area
SAR: structure–activity relationship
SFC: supercritical fluid chromatography
shRNA: short hairpin ribonucleic acid
TAMRA: tetramethylrhodamine

## References

[ref1] Mavrakis K. J.; McDonald E. R.; Schlabach M. R.; Billy E.; Hoffman G. R.; deWeck A.; Ruddy D. A.; Venkatesan K.; Yu J.; McAllister G.; Stump M.; deBeaumont R.; Ho S.; Yue Y.; Liu Y.; Yan-Neale Y.; Yang G.; Lin F.; Yin H.; Gao H.; Kipp D. R.; Zhao S.; McNamara J. T.; Sprague E. R.; Zheng B.; Lin Y.; Cho Y. S.; Gu J.; Crawford K.; Ciccone D.; Vitari A. C.; Lai A.; Capka V.; Hurov K.; Porter J. A.; Tallarico J.; Mickanin C.; Lees E.; Pagliarini R.; Keen N.; Schmelzle T.; Hofmann F.; Stegmeier F.; Sellers W. R. Disordered Methionine Metabolism in MTAP/CDKN2A-Deleted Cancers Leads to Dependence on PRMT5. Science 2016, 351 (6278), 1208–1213. 10.1126/science.aad5944. 26912361

[ref2] Marjon K.; Cameron M. J.; Quang P.; Clasquin M. F.; Mandley E.; Kunii K.; McVay M.; Choe S.; Kernytsky A.; Gross S.; Konteatis Z.; Murtie J.; Blake M. L.; Travins J.; Dorsch M.; Biller S. A.; Marks K. M. MTAP Deletions in Cancer Create Vulnerability to Targeting of the MAT2A/PRMT5/RIOK1 Axis. Cell Reports 2016, 15 (3), 574–587. 10.1016/j.celrep.2016.03.043. 27068473

[ref3] Kryukov G. V.; Wilson F. H.; Ruth J. R.; Paulk J.; Tsherniak A.; Marlow S. E.; Vazquez F.; Weir B. A.; Fitzgerald M. E.; Tanaka M.; Bielski C. M.; Scott J. M.; Dennis C.; Cowley G. S.; Boehm J. S.; Root D. E.; Golub T. R.; Clish C. B.; Bradner J. E.; Hahn W. C.; Garraway L. A. MTAP Deletion Confers Enhanced Dependency on the PRMT5 Arginine Methyltransferase in Cancer Cells. Science 2016, 351 (6278), 1214–1218. 10.1126/science.aad5214. 26912360 PMC4997612

[ref4] Cerami E.; Gao J.; Dogrusoz U.; Gross B. E.; Sumer S. O.; Aksoy B. A.; Jacobsen A.; Byrne C. J.; Heuer M. L.; Larsson E.; Antipin Y.; Reva B.; Goldberg A. P.; Sander C.; Schultz N. The CBio Cancer Genomics Portal: An Open Platform for Exploring Multidimensional Cancer Genomics Data. Cancer Discov 2012, 2 (5), 401–404. 10.1158/2159-8290.CD-12-0095. 22588877 PMC3956037

[ref5] Gao J.; Aksoy B. A.; Dogrusoz U.; Dresdner G.; Gross B.; Sumer S. O.; Sun Y.; Jacobsen A.; Sinha R.; Larsson E.; Cerami E.; Sander C.; Schultz N. Integrative Analysis of Complex Cancer Genomics and Clinical Profiles Using the CBioPortal. Sci. Signal 2013, 6 (269), pl1–pl1. 10.1126/scisignal.2004088. 23550210 PMC4160307

[ref6] Lee W.; Teckie S.; Wiesner T.; Ran L.; Prieto Granada C. N; Lin M.; Zhu S.; Cao Z.; Liang Y.; Sboner A.; Tap W. D; Fletcher J. A; Huberman K. H; Qin L.-X.; Viale A.; Singer S.; Zheng D.; Berger M. F; Chen Y.; Antonescu C. R; Chi P. PRC2 Is Recurrently Inactivated through EED or SUZ12 Loss in Malignant Peripheral Nerve Sheath Tumors. Nat. Genet. 2014, 46 (11), 1227–1232. 10.1038/ng.3095. 25240281 PMC4249650

[ref7] Blanc R. S.; Richard S. Arginine Methylation: The Coming of Age. Mol. Cell 2017, 65 (1), 8–24. 10.1016/j.molcel.2016.11.003. 28061334

[ref8] Musiani D.; Bok J.; Massignani E.; Wu L.; Tabaglio T.; Ippolito M. R.; Cuomo A.; Ozbek U.; Zorgati H.; Ghoshdastider U.; Robinson R. C.; Guccione E.; Bonaldi T. Proteomics Profiling of Arginine Methylation Defines PRMT5 Substrate Specificity. Sci. Signal 2019, 12 (575), eaat8388 10.1126/scisignal.aat8388. 30940768

[ref9] Stopa N.; Krebs J. E.; Shechter D. The PRMT5 Arginine Methyltransferase: Many Roles in Development, Cancer and Beyond. Cell. Mol. Life Sci. 2015, 72 (11), 2041–2059. 10.1007/s00018-015-1847-9. 25662273 PMC4430368

[ref10] Belmontes B.; Slemmons K.; Liu S. The Discovery and Preclinical Characterization of AMG 193, a First-in-Class MTA-Cooperative PRMT5 Inhibitor with Broad Activity against MTAP-Null Cancers. Mol. Cancer Ther. 2023, 22, B177 10.1158/1535-7163.TARG-23-B177.

[ref11] Smith C. R.; Aranda R.; Bobinski T. P.; Briere D. M.; Burns A. C.; Christensen J. G.; Clarine J.; Engstrom L. D.; Gunn R. J.; Ivetac A.; Jean-Baptiste R.; Ketcham J. M.; Kobayashi M.; Kuehler J.; Kulyk S.; Lawson J. D.; Moya K.; Olson P.; Rahbaek L.; Thomas N. C.; Wang X.; Waters L. M.; Marx M. A. Fragment-Based Discovery of MRTX1719, a Synthetic Lethal Inhibitor of the PRMT5•MTA Complex for the Treatment of MTAP-Deleted Cancers. J. Med. Chem. 2022, 65 (3), 1749–1766. 10.1021/acs.jmedchem.1c01900. 35041419

[ref12] Cottrell K. M. Discovery of TNG462: A Highly Potent and Selective MTA-Cooperative PRMT5 Inhibitor Synthetic Lethal for MTAP Deleted Cancers. 2023 American Chemical Society National Meeting, San Francisco, CA; American Chemical Society, 2023.

[ref13] Cottrell K. M.; Maxwell J. P.; Whittington D. A. Compounds and Methods of Use. US11077101B1, 2021.

[ref14] Cottrell K. M.; Maxwell J. P. Compounds and Methods of Use. WO2021086879, 2021.

[ref15] Pearce B. C.; Sofia M. J.; Good A. C.; Drexler D. M.; Stock D. A. An Empirical Process for the Design of High-Throughput Screening Deck Filters. J. Chem. Inf Model 2006, 46 (3), 1060–1068. 10.1021/ci050504m. 16711725

[ref16] Huggins D. J.; Venkitaraman A. R.; Spring D. R. Rational Methods for the Selection of Diverse Screening Compounds. ACS Chem. Biol. 2011, 6 (3), 208–217. 10.1021/cb100420r. 21261294 PMC4765079

[ref17] Rishton G. M. Reactive Compounds and in Vitro False Positives in HTS. Drug Discovery Today 1997, 2 (9), 382–384. 10.1016/S1359-6446(97)01083-0.

[ref18] Giugliano R. P.; Ruff C. T.; Braunwald E.; Murphy S. A.; Wiviott S. D.; Halperin J. L.; Waldo A. L.; Ezekowitz M. D.; Weitz J. I.; Spinar J.; Ruzyllo W.; Ruda M.; Koretsune Y.; Betcher J.; Shi M.; Grip L. T.; Patel S. P.; Patel I.; Hanyok J. J.; Mercuri M.; Antman E. M. 48. Edoxaban versus Warfarin in Patients with Atrial Fibrillation. N. Engl. J. Med. 2013, 369 (22), 2093–2104. 10.1056/NEJMoa1310907. 24251359

[ref19] Birch A. M.; Kenny P. W.; Simpson I.; Whittamore P. R. O. Matched Molecular Pair Analysis of Activity and Properties of Glycogen Phosphorylase Inhibitors. Bioorg. Med. Chem. Lett. 2009, 19 (3), 850–853. 10.1016/j.bmcl.2008.12.003. 19103484

[ref20] Papadatos G.; Alkarouri M.; Gillet V. J.; Willett P.; Kadirkamanathan V.; Luscombe C. N.; Bravi G.; Richmond N. J.; Pickett S. D.; Hussain J.; Pritchard J. M.; Cooper A. W. J.; Macdonald S. J. F. Lead Optimization Using Matched Molecular Pairs: Inclusion of Contextual Information for Enhanced Prediction of HERG Inhibition, Solubility, and Lipophilicity. J. Chem. Inf Model 2010, 50 (10), 1872–1886. 10.1021/ci100258p. 20873842

[ref21] Dossetter A. G.; Griffen E. J.; Leach A. G. Matched Molecular Pair Analysis in Drug Discovery. Drug Discovery Today 2013, 18 (15–16), 724–731. 10.1016/j.drudis.2013.03.003. 23557664

[ref22] Yung-Chi C.; Prusoff W. H. Relationship between the Inhibition Constant (KI) and the Concentration of Inhibitor Which Causes 50 per Cent Inhibition (I50) of an Enzymatic Reaction. Biochem. Pharmacol. 1973, 22 (23), 3099–3108. 10.1016/0006-2952(73)90196-2. 4202581

[ref23] Fragment-Based Discovery of MRTX9768, a Synthetic Lethal-Based Inhibitor Designed to Bind the PRMT5•MTA Complex and Selectively Target MTAPDEL Tumors; American Association for Cancer Research, 2021.

[ref24] Beno B. R.; Yeung K.-S.; Bartberger M. D.; Pennington L. D.; Meanwell N. A. A Survey of the Role of Noncovalent Sulfur Interactions in Drug Design. J. Med. Chem. 2015, 58 (11), 4383–4438. 10.1021/jm501853m. 25734370

[ref25] Koebel M. R.; Cooper A.; Schmadeke G.; Jeon S.; Narayan M.; Sirimulla S. S···O and S···N Sulfur Bonding Interactions in Protein-Ligand Complexes: Empirical Considerations and Scoring Function. J. Chem. Inf. Model. 2016, 56 (12), 2298–2309. 10.1021/acs.jcim.6b00236. 27936771

[ref26] Zhang X.; Gong Z.; Li J.; Lu T. Intermolecular Sulfur···Oxygen Interactions: Theoretical and Statistical Investigations. J. Chem. Inf. Model. 2015, 55 (10), 2138–2153. 10.1021/acs.jcim.5b00177. 26393532

[ref27] Pierce A. C.; ter Haar E.; Binch H. M.; Kay D. P.; Patel S. R.; Li P. CH···O and CH···N Hydrogen Bonds in Ligand Design: A Novel Quinazolin-4-Ylthiazol-2-Ylamine Protein Kinase Inhibitor. J. Med. Chem. 2005, 48 (4), 1278–1281. 10.1021/jm0492249. 15715498

[ref28] Erickson J. A.; McLoughlin J. I. Hydrogen Bond Donor Properties of the Difluoromethyl Group. J. Org. Chem. 1995, 60 (6), 1626–1631. 10.1021/jo00111a021.

[ref29] Nagaya Y.; Nozaki Y.; Kobayashi K.; Takenaka O.; Nakatani Y.; Kusano K.; Yoshimura T.; Kusuhara H. Utility of Cerebrospinal Fluid Drug Concentration as a Surrogate for Unbound Brain Concentration in Nonhuman Primates. Drug Metab. Pharmacokinet. 2014, 29 (5), 419–426. 10.2133/dmpk.DMPK-14-RG-026. 24806821

