# Supplementary material for: Discovery of TNG908: A Selective, Brain Penetrant, MTA-Cooperative PRMT5 Inhibitor That Is Synthetically Lethal with MTAP-Deleted Cancers
Source: J Med Chem. 2024 Apr 10;67(8):6064–80. doi: 10.1021/acs.jmedchem.4c00133 (PMC11056935; doi:10.1021/acs.jmedchem.4c00133)
Supplement: Supplementary file 1 — jm4c00133_si_001.pdf [file jm4c00133_si_001.pdf]

SUPPORTING INFORMATION

DISCOVERY OF TNG908: A SELECTIVE, BRAIN PENETRANT MTA-COOPERATIVE PRMT5 INHIBITOR THAT IS SYNTHETIC LETHAL WITH *MTAP*-DELETED CANCERS

Kevin M. Cottrell[*‡], Kimberly J. Briggs[‡], Douglas A. Whittington[‡], Haris Jahic[‡], Janid A. Ali[‡], Charles B. Davis[‡], Shanzhong Gong[‡], Deepali Gotur[‡], Lina Gu[‡], Patrick McCarren[‡], Matthew R. Tonini[‡], Alice Tsai[‡], Erik W. Wilker[‡], Hongling Yuan[‡], Minjie Zhang[‡], Wenhai Zhang[‡], Alan Huang[‡], and John P. Maxwell[‡].

[‡]Tango Therapeutics, Boston, MA 02215, United States
[*]Corresponding Author kcottrell@tangotx.com

Table of Contents

General experimental and chemical procedures....S1
NMR Spectra and HPLC/LCMS traces of final compounds....S40
Analytical data for TNG908....S108
Small molecule crystal structure of TNG908....S118
Biochemical fluorescence anisotropy peptide displacement assay....S127
Double titration Ki measurement of TNG908....S127
Biochemical methyltransferase FlashPlate assay....S127
Reversibility measurement using Zeba™ spin column activity recovery assay....S128
Methyltransferase panel for TNG908....S130
Eurofins SAFETYscan panel for TNG908....S131
MDR1-MDCKII assay....S133
Human liver microsomes assay....S133
Kinetic solubility assay....S133
hERG assay....S134
In vivo PK....S135
SDMA quantification in PKPD studies....S137
Co-crystal structure determination....S138
Small molecule binding by Surface plasmon resonance (SPR) S139

General experimental and chemical procedures

All chemicals were provided by Enamine Ltd., WuXi Apptech, or other commercial suppliers and used as received unless otherwise indicated. All solvents were treated according to standard methods. All reactions were monitored and analysis of final compounds performed by LC-MS using Agilent 1260 LC/MSD instruments, with an Agilent Poroshell 120 SB-C18 4.6 x 30mm 2.7 µm column, column Temperature: 60°C, mobile phase: A – H_2O (0.1% formic acid), B – ACN (0.1% formic acid), flow rate: 1.5 mL/min, gradient: 0.01 min – 1% B, 5.00 min – 100% B, 5.99 min – 100% B, MS Ionization mode: Electrospray ionization (ESI), MS Scan range: 83 – 1000 m/z, UV detection: 215 nm, 254 nm, 280 nm unless otherwise specified. Thin-layer chromatography (TLC) with pre-coated silica gel GF254 (0.2 mm) was used and the results were visualized using either UV light or $KMnO_4$ stain. Proton nuclear magnetic resonance (^{1}H-NMR) spectra were recorded at 400, 500 or 600 MHz on Varian or Bruker instrumentation; chemical shifts were calibrated using residual non-deuterated solvents $CHCl_3$ (δ = 7.26 ppm), DMSO (δ = 2.50 ppm) or MeOH (δ = 3.31 ppm) and expressed in δ ppm. Coupling constants (*J*), when given, are reported in hertz. Multiplicities

are reported using the following abbreviations: s = singlet, d = doublet, dd = doublet of doublets, t = triplet, q = multiplet (range of multiplets is given), br = broad signal, dt = doublet of triplets. ^{19}F NMR spectra were recorded at 376 MHz (Varian), ^{13}C NMR spectra were recorded at 101, 126 or 151 MHz (Varian). ^{13}C NMR chemical shifts for ^{13}C NMR are reported relative to the central $CHCl_3$ (δ = 77.16 ppm), DMSO (δ = 39.52 ppm) or MeOH (δ = 49.00 ppm) and chemical shifts are reported in parts per million (ppm). All final compounds were purified by reverse phase high-performance liquid chromatography (HPLC) or supercritical fluid chromatography (SFC) or silica gel chromatography (100-200 mesh). HPLC was done with an Agilent 1260 HPLC instrument (Agilent Technologies, Germany) equipped with a G7161A Preparative Binary Pump, a G7157A Prep Autosampler, a G7115A DAD WR and a G7159B Preparative Fraction Collector. The Open Lab CDS software (version C.01.10 was used for instrument control, data acquisition and data handling). SFC was done with a Waters 100q Prep SFC System. Chiral HPLC analytical analysis was done with an Agilent 1200 HPLC instrument (Agilent Technologies, Germany) equipped with a G1379B degasser, a G1312A Binary Pump, a G1329A ALS autosampler, a G1315A Diode Array Detector. Chiral SFC analytical analysis was done with an Agilent 1260 SFC instrument (Agilent Technologies, Germany) equipped with a G1379B degasser, a G1312B Binary Pump, a G1313A ALS autosampler, a G1316A thermostatted column compartment, a G1315D Diode Array Detector and an Aurora SFC systems. Melting points were taken using OptiMelt Automated Melting Point System Digital Image Processing Technology SRS Stanford Pesearch Systems, 2°C/min (5 °C/min at high melting point). Optical rotation was measured with Polarimeter Anton Paar GmbH MCP 300 (Accuracy: ±0.003°) used to measure the angle of optical rotation. Standard conditions for analysis: solution concentration 0.5 g/100 mL (solvent: MeOH), wavelength 589 nm, temperature 21 °C. All oxamides exist as rotamers in ^{1}H NMR spectra. All compounds are > 95% pure by HPLC.

Compounds 1, 1R, 1S, 2R, 2S, 3-10, 16, 17

General procedure A. Diisopropylethylamine (1.5 eq) was added to a solution of 5-methylpyridin-3-amine **48** (1.0 eq) in ACN (0.5 M). The resulting mixture was stirred for 30 min at room temperature followed by the dropwise addition of 2,2,2-trifluoroethyl-2-chloro-2-oxoacetate (1.0 eq). The mixture was stirred for additional 30 min and substituted piperidine (1.1 eq) was added. The reaction mixture was stirred for 1h at room temperature and then for 16 h at 100 °C. The resulting mixture was allowed to cool to room temperature, concentrated under reduced pressure, and the residue was dissolved in DMSO. The resulting solution was purified by reverse phase HPLC (Waters Sunfire C18 19 x 100 mm 5 µm column and H2O-MeCN) to afford the final product, followed by chiral chromatography to separate isomers where appropriate.

The following compounds were prepared by general procedure A.

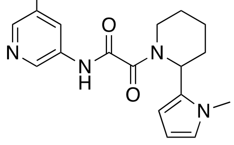

1

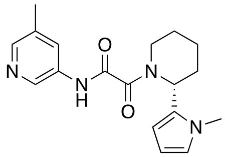

1R

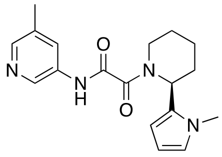
1S

2-(2-(1-methyl-1H-pyrrol-2-yl)piperidin-1-yl)-N-(5-methylpyridin-3-yl)-2-oxoacetamide (1), (R)-2-(2-(1-methyl-1H-pyrrol-2-yl)piperidin-1-yl)-N-(5-methylpyridin-3-yl)-2-oxoacetamide (1R), (S)-2-(2-(1-methyl-1H-pyrrol-2-yl)piperidin-1-yl)-N-(5-methylpyridin-3-yl)-2-oxoacetamide (1S).
Piperidine: 2-(1-methyl-1H-pyrrol-2-yl)piperidine. White powder, 15% yield. ^{1}H NMR (500 MHz, DMSO-*d*$_6$): δ 1.67 (m, 2H), 1.78 (d, 1H), 1.94 (m, 1H), 2.16 (m, 2H), 2.46 (s, 3H), 3.09 (m, 1H), 3.54 (s, 3H), 3.62 (m, 1H), 5.72 (m, 1H), 5.90 (m, 1H), 6.11 (m, 1H), 6.55 (m, 1H), 7.40 (m, 1H), 7.52 (m, 1H), 8.25 (d, 1H), 10.91 (m, 1H). LCMS (ESI): [M+H]$^+$ m/z: calcd for $C_{18}H_{23}N_4O_2^+$ 327.4; found 327.0; Rt = 4.022 min. Racemic mixture (**1**) (80 mg, 0.2 mmol) was separated by chiral chromatography (column: OJ-H (250 x 20 mm, 5 μm), mobile phase: 80:10:10 hexanes, IPA, MeOH, 13 mL/min) to give **1R** (22.0 mg, 0.068 mmol, 34% yield) as a yellow solid. ^{1}H NMR (500 MHz, $CDCl_3$): δ 1.71 (m, 1H), 1.79 (m, 2H), 2.01 (m, 1H), 2.20 (m, 1H), 2.26 (m, 1H), 2.37 (s, 3H), 3.13 (m, 1H), 3.56 (m, 3H), 4.85 (m, 1H), 5.97 (m, 2H), 6.33 (m, 1H), 6.61 (m, 1H), 8.04 (m, 1H), 8.26 (s, 1H), 8.51 (s, 1H), 9.26 (m, 1H). LCMS (ESI): [M+H]+ m/z calcd 326.2; found 327.0; Rt=4.022 min. Rt(OJ-H, hexanes:IPA:MeOH, 80:10:10, 0.6 mL/min) = 16.67 min and **1S** (16.6 mg, 0.051 mmol, 26% yield) as a yellow solid. ^{1}H NMR (500 MHz, $CDCl_3$): δ 1.65 (m, 1H), 1.77 (m, 2H), 1.96 (m, 1H), 2.13 (m, 1H), 2.23 (m, 1H), 2.33 (s, 3H), 3.16 (m, 1H), 3.52 (m, 3H), 4.73 (m, 1H), 5.99 (m, 2H), 6.29 (m, 1H), 6.60 (m, 1H), 7.99 (m, 1H), 8.22 (s, 1H), 8.47 (s, 1H), 9.40 (m, 1H). LCMS (ESI): [M+H]+ m/z calcd 326.2; found 327.0; Rt=4.022 min. Rt (OJ-H, hexanes:IPA:MeOH, 80:10:10, 0.6 mL/min) = 13.722 min.

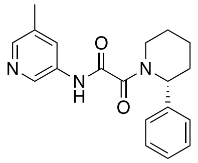
2R

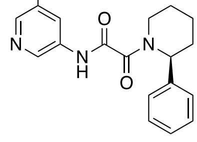
2S

(R)-N-(5-methylpyridin-3-yl)-2-oxo-2-(2-phenylpiperidin-1-yl)acetamide (2R), (S)-N-(5-methylpyridin-3-yl)-2-oxo-2-(2-phenylpiperidin-1-yl)acetamide (2S).
Piperidine: 2-phenylpiperidine. Chiral separation was performed using IC (250 x 20 mm, 5 μm), Hexane-IPA-MeOH, 70-15-15, 12 mL/min, 12 mg per injection affording N-(5-methyl-3-pyridyl)-2-oxo-2-[(2R)-2-phenyl-1-piperidyl]acetamide **2R** as white solid in 4% yield. ^{1}H NMR (DMSO-*d*$_6$, 500 MHz): δ 1.65 (m, 4H), 1.98 (m, 1H), 2.34 (m, 3H), 3.06 (m, 2H), 4.07 (m, 1H), 5.50 (m, 1H), 7.25 (t, 1H), 7.34 (m, 4H), 7.99 (d, 1H), 8.08 (d, 1H), 8.56 (d, 1H), 10.92 (d, 1H). LCMS (ESI): [M+H]+ m/z: calcd 323.2; found 324.0; Rt = 4.293 min. RT (IC, Hexane-IPA-MeOH, 70-15-15, 0.6 mL/min) = 36.713 min and N-(5-methyl-3-pyridyl)-2-oxo-2-[(2S)-2-phenyl-1-piperidyl]acetamide **2S** as beige solid in 4% yield ^{1}H NMR (DMSO-*d*$_6$, 500 MHz): δ 1.64 (m, 4H), 1.97 (m, 1H), 2.34 (m, 3H), 3.05 (m, 1H), 4.07 (m, 1H), 5.51 (m, 1H), 7.25 (t, 1H), 7.31 (d, 1H), 7.37 (m, 3H), 7.99 (d, 1H), 8.08 (d, 1H), 8.56 (d, 1H), 10.92 (d, 1H). LCMS (ESI): [M+H]+ m/z: calcd 323.2; found 324.0; Rt = 4.293 min. RT (IC, Hexane-IPA-MeOH, 70-15-15, 0.6 mL/min) = 29.646 min.

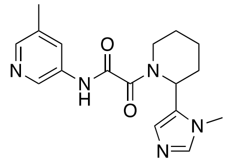

2-(2-(1-methyl-1H-imidazol-5-yl)piperidin-1-yl)-N-(5-methylpyridin-3-yl)-2-oxoacetamide (3).
Piperidine: 2-(1-methyl-1H-imidazol-5-yl)piperidine. White solid, 22% yield. ^{1}H NMR (400 MHz, DMSO-d_6 + CCl_4) δ 1.70 (m, 3H), 2.01 (m, 3H), 2.17 (m, 1H), 2.35 (s, 3H), 3.58 (m, 3H), 3.70 (m, 1H), 5.77 (m, 1H), 7.00 (s, 1H), 7.48 (s, 1H), 7.97 (s, 1H), 8.10 (s, 1H), 8.54 (s, 1H), 10.81 (s, 1H). LCMS (ESI): [M+H]+ m/z: calcd 327.2; found 328.2; Rt = 0.670 min, column: Agilent Poroshell 120 SB-C18 4.6 x 30 mm 2.7 µm, 60 °C, Mobile phase: A – H_2O (0.1% formic acid), B – ACN (0.1% formic acid), flow rate: 3 mL/min, gradient: 0.01 min – 1% B, 1.5 min – 100% B, 1.73 min – 100% B, MS Ionization mode: Electrospray ionization (ESI), MS Scan range: 83 – 600 m/z, UV detection: 215 nm, 254nm, 280 nm.

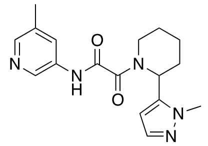

2-(2-(1-methyl-1H-pyrazol-5-yl)piperidin-1-yl)-N-(5-methylpyridin-3-yl)-2-oxoacetamide (4).
Piperidine: 2-(1-methyl-1H-pyrazol-5-yl)piperidine. White solid, 9% yield. ^{1}H NMR (500 MHz, DMSO-d_6 + CCl_4) δ 1.72 (m, 2H), 1.80 (m, 1H), 1.98 (m, 2H), 2.15 (m, 1H), 2.36 (m, 3H), 3.05 (m, 1H), 3.94 (m, 4H), 5.65 (m, 1H), 6.32 (m, 1H), 7.26 (m, 1H), 8.01 (m, 1H), 8.10 (m, 1H), 8.55 (m, 1H), 10.88 (m, 1H). LCMS (ESI): [M+H]+ m/z: calcd 327.3; found 328.2; Rt = 0.904 min, column: Agilent Poroshell 120 SB-C18 4.6 x 30 mm 2.7 µm, 60 °C, Mobile phase: A – H_2O (0.1% formic acid), B – ACN (0.1% formic acid), flow rate: 3 mL/min, gradient: 0.01 min – 1% B, 1.5 min – 100% B, 1.73 min – 100% B, MS Ionization mode: Electrospray ionization (ESI), MS Scan range: 83 – 600 m/z, UV detection: 215 nm, 254nm, 280 nm.

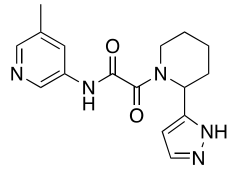

2-(2-(1H-pyrazol-5-yl)piperidin-1-yl)-N-(5-methylpyridin-3-yl)-2-oxoacetamide (5).
Piperidine: 2-(1H-pyrazol-5-yl)piperidine. Light yellow solid, 17 % yield. ^{1}H NMR ($CDCl_3$, 500 MHz): δ 1.61 (m, 1H), 1.76 (m, 4H), 2.00 (m, 1H), 2.36 (m, 3H), 3.01 (m, 2H), 4.60 (m, 1H), 6.00 (m, 1H), 6.28 (d, 1H), 7.58 (s, 1H), 8.07 (d, 1H), 8.24 (s, 1H), 8.49 (m, 1H), 9.50 (m, 1H). LCMS (ESI): [M+H]+ m/z: calcd 313.3; found 314.2; Rt = 2.096 min.

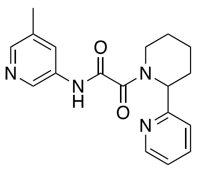

N-(5-methylpyridin-3-yl)-2-oxo-2-(2-(pyridin-2-yl)piperidin-1-yl)acetamide (6). Piperidine: 2-(piperidin-2-yl)pyridine. White solid, 17% yield. ^{1}H NMR (500 MHz, DMSO-d_6 + CCl_4) δ 1.52 (m, 1H), 1.66 (m, 3H), 1.84 (m, 1H), 2.33 (m, 3H), 2.73 (m, 2H), 3.16 (m, 0.5H), 4.13 (m, 1H), 5.50 (m, 1H), 7.24 (m, 1H), 7.39 (m, 1H), 7.77 (m, 1H), 7.97 (m, 1H), 8.07 (m, 1H), 8.56 (m, 2H), 10.86 (m, 1H). LCMS (ESI): [M+H]+ m/z: calcd 324.3; found 325.2; Rt = 0.932 min, column: Agilent Poroshell 120 SB-C18 4.6 x 30 mm 2.7 μm, 60 °C, Mobile phase: A – H_2O (0.1% formic acid), B – ACN (0.1% formic acid), flow rate: 3 mL/min, gradient: 0.01 min – 1% B, 1.5 min – 100% B, 1.73 min – 100% B, MS Ionization mode: Electrospray ionization (ESI), MS Scan range: 83 – 600 m/z, UV detection: 215 nm, 254nm, 280 nm.

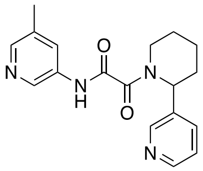

N-(5-methylpyridin-3-yl)-2-oxo-2-(2-(pyridin-3-yl)piperidin-1-yl)acetamide (7). Piperidine: 3-(piperidin-2-yl)pyridine. White solid, 10% yield. ^{1}H NMR (500 MHz, DMSO-d_6) δ 1.55 (m, 1H), 1.67 (m, 2H), 1.76 (m, 1H), 2.00 (m, 1H), 2.34 (m, 3H), 2.59 (m, 2H), 4.10 (m, 1H), 5.56 (m, 1H), 7.36 (m, 1H), 7.72 (m, 1H), 7.98 (m, 1H), 8.09 (m, 1H), 8.46 (m, 1H), 8.56 (m, 2H), 10.94 (s, 1H). LCMS (ESI): [M+H]+ m/z: calcd 324.3; found 325.2; Rt = 0.757 min, column: Agilent Poroshell 120 SB-C18 4.6 x 30 mm 2.7 μm, 60 °C, Mobile phase: A – H_2O (0.1% formic acid), B – ACN (0.1% formic acid), flow rate: 3 mL/min, gradient: 0.01 min – 1% B, 1.5 min – 100% B, 1.73 min – 100% B, MS Ionization mode: Electrospray ionization (ESI), MS Scan range: 83 – 600 m/z, UV detection: 215 nm, 254nm, 280 nm.

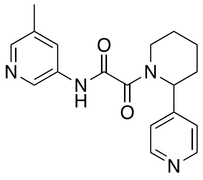

N-(5-methylpyridin-3-yl)-2-oxo-2-(2-(pyridin-4-yl)piperidin-1-yl)acetamide (8). Piperidine: 4-(piperidin-2-yl)pyridine. White solid, 14% yield. ^{1}H NMR, δ 1.45 (m, 1H), 1.69 (m, 3H), 1.98 (m, 1H), 2.30* (s, 1H), 2.36 (s, 2H), 2.49 (m, 2H), 3.84 (d, 0.65H), 4.41* (d, 0.35H), 5.28* (s, 0.35H), 5.72 (d, 0.65H), 7.29 (d, 1.2H), 7.32* (d, 0.8H), 7.93* (s, 0.35H), 8.01 (s, 0.65H), 8.06* (s, 0.35H), 8.10 (s, 0.65H), 8.55 (m, 3H), 10.93* (s, 0.35H), 10.95 (s, 0.65H). LCMS (ESI): [M+H]+ m/z: calcd 324.3; found 325.2; Rt = 0.753 min, column: Agilent Poroshell 120 SB-C18 4.6 x 30 mm 2.7 μm, 60 °C, Mobile phase: A – H_2O (0.1% formic acid), B – ACN (0.1% formic acid), flow rate: 3 mL/min, gradient: 0.01 min – 1% B, 1.5 min – 100% B, 1.73 min – 100% B, MS Ionization mode: Electrospray ionization (ESI), MS Scan range: 83 – 600 m/z, UV detection: 215 nm, 254 nm, 280 nm.

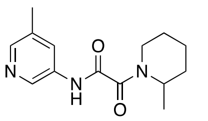

2-(2-methylpiperidin-1-yl)-N-(5-methylpyridin-3-yl)-2-oxoacetamide (9).
Piperidine: 2-methylpiperidine. White solid, 39 % yield. ^{1}H NMR (DMSO-d_6, 500 MHz): δ 1.28 (dd, 3H), 1.54 (m, 2H), 1.71 (m, 4H), 2.34 (s, 3H), 2.81 (m, 1H), 3.18 (m, 1H), 4.15 (m, 2H), 7.96 (s, ^{1}H), 8.07 (s, ^{1}H), 8.53 (s, 1H), 10.71 (m, 1H). LCMS (ESI): [M+H]+ m/z: calcd 261.2; found 262.2; Rt = 0.898 min, column: Agilent Poroshell 120 SB-C18 4.6 x 30 mm, 2.7 µm, 60 °C, Mobile phase: A – H_2O (0.1% formic acid), B – ACN (0.1% formic acid), flow rate: 3 mL/min, gradient: 0.01 min – 1% B, 1.5 min – 100% B, 1.73 min – 100% B, MS Ionization mode: Electrospray ionization (ESI), MS Scan range: 83 – 600 m/z, UV detection: 215 nm, 254 nm, 280 nm.

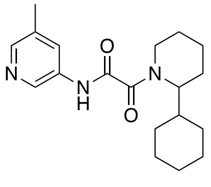

2-(2-cyclohexylpiperidin-1-yl)-N-(5-methylpyridin-3-yl)-2-oxoacetamide (10).
Piperidine: 2-cyclohexylpiperidine. White solid, 5% yield. ^{1}H NMR (DMSO-d_6, 400 MHz): δ 1.42 (m, 18H), 2.29 (s, 3H), 3.52 (m, 1H), 4.24 (m, 1H), 7.91 (s, 1H), 8.17 (s, 1H), 8.60 (d, 1H), 10.88 (brs, 1H). LCMS (ESI): [M+H]+ m/z: calcd 329.2; found 330.2; Rt = 3.213 min.

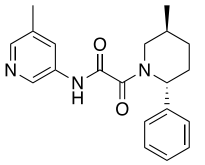

2-((2*R*,5*S*)-5-methyl-2-phenylpiperidin-1-yl)-*N*-(5-methylpyridin-3-yl)-2-oxoacetamide (16).
Piperidine: 5-methyl-2-phenylpiperidine. Beige solid, 2% yield as two steps including separation of isomers. ^{1}H NMR (500 MHz, $CDCl_3$) δ 1.12 (d, 3H), 1.42 (m, 1H), 1.95 (m, 2H), 2.27 (m, 2H), 2.36 (m, 3H), 3.21 (m, 1H), 4.56 (m, 1H), 6.16 (m, 1H), 7.30 (m, 3H), 7.39 (m, 2H), 8.04 (m, 1H), 8.25 (m, 1H), 8.50 (m, 1H), 9.33 (m, 1H). LCMS (ESI): [M+2H]+ m/z: calcd 337.18; found 339.03; Rt = 4.740 min. RT (OJ-H, Hexane-IPA-MeOH, 70-15-15, 0.6 mL/min) = 13.646 min.

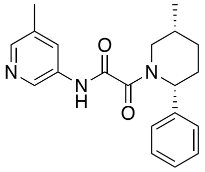

2-((2*R*,5*R*)-5-methyl-2-phenylpiperidin-1-yl)-*N*-(5-methylpyridin-3-yl)-2-oxoacetamide (17).
Piperidine: 5-methyl-2-phenylpiperidine. Grey solid, 1% yield over 2 steps including chiral separation of isomers. ^{1}H NMR (500 MHz, $CDCl_3$) δ 0.87 (m, 3H), 1.30 (m, 1H), 1.79 (m, 2H), 2.04 (m, 1H), 2.36 (m, 3H), 2.53 (m, 1H), 2.66 (m, 1H), 4.64 (m, 1H), 6.23 (m, 1H), 7.30 (m, 3H), 7.40 (m, 2H), 8.06 (m, 1H), 8.26 (m, 1H), 8.51 (m, 1H), 9.27 (m, 1H). LCMS (ESI): [M+2H]+ m/z: calcd 337.18; found 339.03; Rt = 4.808 min. RT (OJ-H, Hexane-IPA-MeOH, 70-15-15, 0.6 mL/min) = 10.295 min.

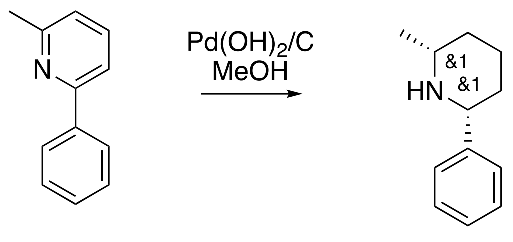

Intermediate 15a

rac-(2R,6R)-2-methyl-6-phenylpiperidine, intermediate 15a
To a solution of 2-methyl-6-phenyl-pyridine (3 g, 17.73 mmol) in MeOH (50 mL) added palladium hydroxide on carbon 20% (0.3 g, 17.73 mmol). The reaction flask was evacuated and backfilled with H_2 and the mixture was left to stir overnight. The suspension was filtered through a thin pad of silica gel then the filtrate was concentrated. The residue was purified by column chromatography, Combiflash Companion; 80 g SiO2;$CHCl_3$/ACN with ACN from 0 to 18%, flow rate=60 mL/min, Rv=10-14 cv.) to give (2R,6R)-2-methyl-6-phenyl-piperidine (0.68 g, 3.88 mmol, 22% yield) as a pale-yellow oil.

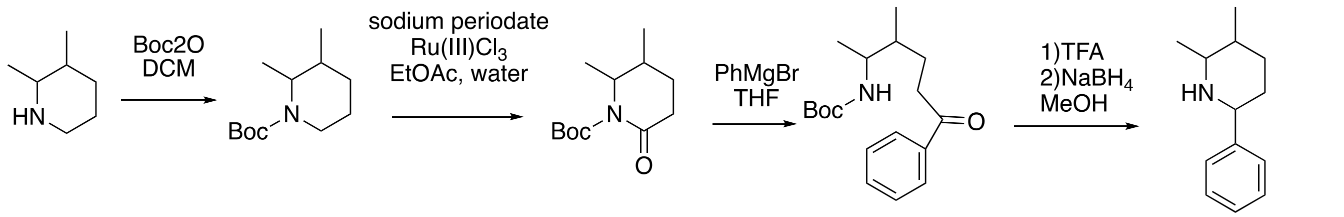

Intermediate 22a

tert-butyl 2,3-dimethylpiperidine-1-carboxylate
tert-butoxycarbonyl tert-butyl carbonate (10.12 g, 46.38 mmol) was added to a solution of 2,3-dimethylpiperidine (5 g, 44.17 mmol) in CH_2Cl_2 (50 mL) and the resulitng mixture was stirred at 20 °C for 12 h. The resulting mixture was evaporated to dryness to obtain tert-butyl 2,3-dimethylpiperidine-1-carboxylate (9.2 g, 43.13 mmol, 98% yield). LCMS (ESI): [M+H-tBu]+ m/z: calcd 157.3; found 158.2; Rt = 1.521 min, column: Agilent Poroshell 120 SB-C18 4.6 x 30 mm 2.7 µm, 60 °C, Mobile phase: A-H_2O (0.1% formic acid), B-ACN (0.1% formic acid), flow rate: 3 mL/min, Gradient: 0.01 min - 1% B, 1.5 min - 100% B, 1.73 min - 100% B, MS Ionization mode: ESI, MS Scan range: 83-600 m/z, UV detection: 215 nm, 254 nm, 280 nm.

tert-butyl 2,3-dimethyl-6-oxopiperidine-1-carboxylate
The a solution of tert-butyl 2,3-dimethylpiperidine-1-carboxylate (8.2 g, 38.44 mmol) in EtOAc (80 mL) was added a solution of sodium periodate (32.89 g, 153.76 mmol) in H_2O (80 mL). Ruthenium(III) chloride hydrate (43.33 mg, 192.20 umol) was then added and the resulting biphasic mixture was stirred at 20 °C for 12 h. The resulting mixture was filtered. The filter cake was washed with EtOAc (2 x 15 mL). The organic phase was separated, washed with brine, dried over sodium sulfate and evaporated to obtain tert-butyl 2,3-dimethyl-6-oxo-piperidine-1-carboxylate (8.25 g, crude) which was used in next step without purification. LCMS (ESI): [M+H-tBu]+ m/z: calcd 171.3; found 172.2; Rt = 1.328 min, column: Agilent Poroshell 120 SB-C18 4.6 X 30 mm 2.7 µm, 60 °C, Mobile phase: A-H_2O (0.1% formic acid), B-ACN (0.1% formic acid), flow rate: 3 mL/min, Gradient: 0.01min-1%B,

1.5 min - 100% B, 1.73 min -100% B, MS Ionization mode: ESI, MS Scan range: 83-600 m/z, UV detection: 215 nm, 254 nm, 280 nm.

tert-butyl (3-methyl-6-oxo-6-phenylhexan-2-yl)carbamate
Bromo(phenyl)magnesium (7.18 g, 39.60 mmol, 1 M in THF solution) was added dropwise to a solution of tert-butyl 2,3-dimethyl-6-oxo-piperidine-1-carboxylate (9 g, 39.60 mmol) in THF (100 mL) at -78 °C under Ar atmosphere. The resulting mixture was left to warm to room temperature and stirred for 12 h. The resulting mixture was poured into sat. aq. NH_4Cl solution and extracted with EtOAc (2 x 150 mL). The combined organic extracts were washed with brine, dried over sodium sulfate and evaporated to obtain tert-butyl N-(1,2-dimethyl-5-oxo-5-phenyl-pentyl)carbamate (10 g, 32.74 mmol, 83% yield) which was used in next step without purification. LCMS (ESI): [M+H-Boc]+ m/z: calcd 205.41; found 206.2; Rt = 1.552 min, column: Agilent Poroshell 120 SB-C18 4.6X30mm 2.7 μm, 60 °C, Mobile phase: A-H_2O (0.1% formic acid), B-ACN (0.1% formic acid), flow rate: 3 mL/min, Gradient: 0.01 min -1% B, 1.5 min - 100% B, 1.73 min - 100% B, MS Ionization mode: ESI, MS Scan range: 83-600 m/z, UV detection: 215 nm, 254 nm, 280 nm.

2,3-dimethyl-6-phenylpiperidine, intermediate **22a**
tert-butyl N-(1,2-dimethyl-5-oxo-5-phenyl-pentyl)carbamate (10 g, 32.74 mmol) was dissolved in trifluoroacetic acid (12.61 mL, 163.71 mmol) and the resulting mixture was stirred for 1 h. 50% aq. NaOH solution was added until the solution reached pH 11 - 12. The resulting mixture was extracted with CH_2Cl_2 (4 x 40 mL) and the combined organic layer was evaporated to dryness. The residue was redissolved in MeOH (100 mL) and sodium borohydride (1.24 g, 32.74 mmol) was added. The resulting mixture was stirred at 20 °C for 1 h and evaporated. 50% aq. NaOH solution was added to the residue. The resulting mixture was extracted with CH_2Cl_2 (4 x 40 mL) and the combined organic layer was evaporated to dryness to obtain 2,3-dimethyl-6-phenyl-piperidine (3 g, 15.85 mmol, 48% yield) which was used in next step without purification. LCMS (ESI): [M+H]+ m/z: calcd 189,3; found 190.2; Rt = 0.854 min, column: Agilent Poroshell 120 SB-C18 4.6 x 30mm 2.7 μm, 60 °C, Mobile phase: A-H_2O (0.1% formic acid), B-ACN (0.1% formic acid), flow rate: 3 mL/min, Gradient: 0.01 min - 1% B, 1.5 min - 100% B, 1.73 min - 100% B, MS Ionization mode: ESI, MS Scan range: 83-600 m/z, UV detection: 215 nm, 254 nm, 280 nm.

Compounds 15, 20, 21, 22

General Procedure B: To a stirred solution of amine in THF (0.36 M) at –78 °C, n-butyllithium (2.5 M in Hexane, 3 eq) was added under argon atmosphere. The resulting mixture was stirred at the same temperature for 5 minutes. After 5 minutes, 2,2,2-trifluoroethyl 2-[(5-methyl-3-pyridyl)amino]-2-oxo-acetate (1 eq) dissolved in THF (0.36 M) was added to the reaction mixture. The resulting mixture was allowed to warm to room temperature and stirred 1 h at the same temperature after which it was quenched with saturated NH_4Cl aq solution and extracted with EtOAc. The organic phase was dried over Na_2SO_4, concentrated under reduced pressure and the crude product was purified by reverse phase HPLC (Waters Sunfire C18 19 x 100mm 5 μm column and H2O-ACN) to give racemic product which was separated by chiral chromatography when appropriate.

The following compounds were prepared with General Procedure B:

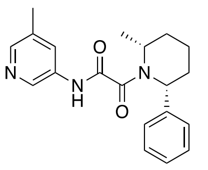

2-((2R,6R)-2-methyl-6-phenylpiperidin-1-yl)-N-(5-methylpyridin-3-yl)-2-oxoacetamide (15). Amine: intermediate 15a. White solid, 2% yield. ^{1}H NMR(DMSO- d_6, 500 MHz): δ 0.79 (d, 3H), 1.55 (d, 1H), 1.67 (m, 1H), 1.90 (m, 3H), 2.35 (s, 3H), 2.60 (m, 1H), 4.46 (m, 1H), 5.80 (m, 1H), 7.21 (s, 1H), 7.35 (m, 4H), 8.00 (s, 1H), 8.09 (s, 1H), 8.56 (s, 1H), 10.88 (s, 1H). LCMS (ESI): [M+H]+ m/z: calcd 337.2; found 338.0; Rt = 4.299 min. Chiral HPLC: Rt = 14.81 min (Column: IA; Mobile phase: Hexane-MeOH-IPA, 60-20-20; Flow Rate: 0.6 mL/min).

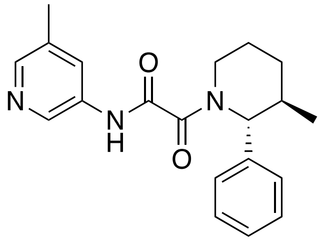

2-((2*R*,3*R*)-3-methyl-2-phenylpiperidin-1-yl)-*N*-(5-methylpyridin-3-yl)-2-oxoacetamide (20). Amine: 3-methyl-2-phenylpiperidine. Off-white solid, 9% yield. ^{1}H NMR ($CDCl_3$, 500 MHz): δ 0.88 (d, 1H), 0.95 (d, 2H), 1.81 (m, 3H), 1.97 (m, 1H), 2.25 (m, 1H), 2.35 (s, 2H), 2.38 (s, 1H), 3.16 (t, 0.3H), 3.43 (t, 0.7H), 4.43 (d, 0.3H), 4.96 (d, 0.7H), 5.68 (d, 0.7H), 6.34 (d, 0.3H), 7.33 (m, 3H), 7.52 (m, 2H), 8.00 (m, 1H), 8.24 (s, 0.7H), 8.26 (s, 0.3H), 8.44 (s,0.3H), 8.46 (s, 0.7H), 8.99 (s, 0.3H), 9.11 (s, 0.7H). LCMS (ESI): [M+H]+ m/z: calcd 337.2; found 338.2; Rt = 3.184 min.

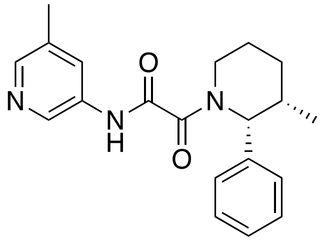

2-((2R,3S)-3-methyl-2-phenylpiperidin-1-yl)-N-(5-methylpyridin-3-yl)-2-oxoacetamide (21). Amine: 3-methyl-2-phenylpiperidine. Off-white solid, 14% yield. ^{1}H NMR ($CDCl_3$, 500 MHz): δ 1.21 (m, 3H), 1.50 (m, 2H), 1.89 (m, 1H), 2.02 (m, 1H), 2.36 (m, 3H), 2.75 (m, 1H), 3.18 (m, 0.6H), 3.53 (m, 0.4H), 4.45 (d, 0.4H), 4.98 (d, 0.6H), 5.48 (s, 0.6H), 6.20 (s, 0.4H), 7.27 (m, 1H), 7.38 (m, 4H), 8.05 (s, 1H), 8.25 (m, 1H), 8.46 (s,0.40H), 8.52 (s, 0.6H), 9.26 (s, 1H).
LCMS (ESI): [M+H]+ m/z: calcd 337.2; found 338.2; Rt = 3.283 min.

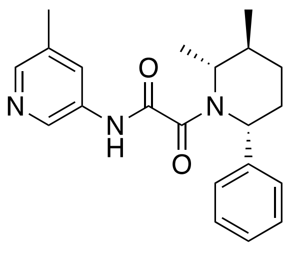

2-((2R,3S,6R)-2,3-dimethyl-6-phenylpiperidin-1-yl)-N-(5-methylpyridin-3-yl)-2-oxoacetamide (22).

Yellow solid, 1% yield over 2 steps including chiral separation. ^{1}H NMR (DMSO-d_6 + CCl_4, 400 MHz): δ 0.76 (m, 3H), 1.11 (m, 3H), 1.51 (m, 1H), 1.71 (m, 1H), 1.98 (m, 1H), 2.12 (m, 1H), 2.34 (m, 3H), 2.61 (m, 1H), 3.84 (m, 1H), 5.57 (m, 1H), 7.21 (m, 1H), 7.31 (m, 2H), 7.43 (m, 2H), 7.97 (m, 1H), 8.10 (m, 1H), 8.57 (m, 1H), 10.89 (m, 1H). LCMS (ESI): [M+H]+ m/z: calcd 351.2; found 352.2; Rt = 4.788 min. Chiral HPLC: Rt = 14.78 min (Column: Chiralcel OJ-H; Mobile phase: Hexane-MeOH-IPA, 70-15-15; Flow Rate: 0.6 mL/min).

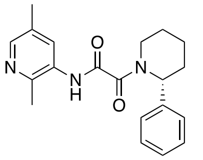

(R)-N-(2,5-dimethylpyridin-3-yl)-2-oxo-2-(2-phenylpiperidin-1-yl)acetamide (11).
2,2,2-trifluoroethyl 2-chloro-2-oxo-acetate (120 mg, 630 μmol) was added dropwise to the solution of 2,5-dimethylpyridin-3-amine (100 mg, 630 μmol, HCl) and DIPEA (285 mg, 2.21 mmol, 384 μL) in ACN (4 mL). The resulting mixture was stirred for 30 min followed by the addition of (2R)-2-phenylpiperidine (125 mg, 630 μmol, HCl). Then, the reaction mixture was stirred at 100 °C for 24 h. After the completion of the reaction, the resulting suspension was allowed to cool down to room temperature and filtered. The obtained filtrate was purified by HPLC (0-2-8 min 13-20-35% H_2O / ACN / 0.1% NH_4OH, 30 mL/min (loading pump 4 mL ACN), target mass 337 column: XBRIDGE C18 100×19mm, 5 μM) and then repurified by HPLC (0-2-8- min 3-10-30% H_2O / ACN / 0.1% FA, 30 mL/min (loading pump 4 mL ACN), target mass 337 column: Chromatorex C18 100×19 mm, 5 μM) to afford N-(2,5-dimethyl-3-pyridyl)-2-oxo-2-[(2R)-2-phenyl-1-piperidyl]acetamide, **11** (3.20 mg, 9.48 μmol, 1.50% yield) as a beige solid. ^{1}H NMR (600 MHz, DMSO-d_6) δ 1.48 (m, 5H), 1.87 (m, 1H), 2.25 (d, 3H), 2.38 (d, 3H), 3.01 (td, 1H), 3.99 (dd, 1H), 5.43 (dd, 1H), 7.28 (t, 1H), 7.32 (d, 1H), 7.40 (m, 3H), 7.58 (dd, 1H), 8.14 (dd, 1H), 10.49 (d, 1H). LCMS (ESI): [M+H]+ m/z: calcd 338.22; found 338.2; Rt = 1.060 min, column: Agilent Poroshell 120 SB-C18 4.6 x 30mm, 2.7 μm, 60 °C, Mobile phase: A – H_2O (0.1% formic acid), B – ACN (0.1% formic acid), flow rate: 3 mL/min, gradient: 0.01 min – 1% B, 1.5 min – 100% B, 1.73 min – 100% B, MS Ionization mode: Electrospray ionization (ESI), MS Scan range: 83 – 600 m/z, UV detection: 215 nm, 254 nm, 280 nm.

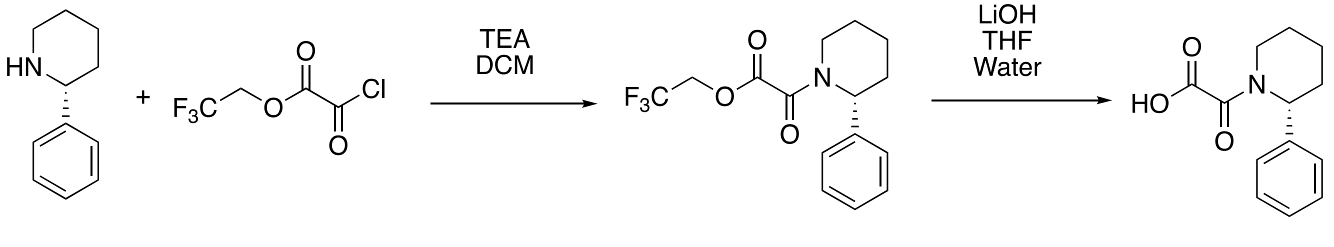

Intermediate 12a

2,2,2-trifluoroethyl (R)-2-oxo-2-(2-phenylpiperidin-1-yl)acetate
To a solution of (2R)-2-phenylpiperidine (1.23 g, 6.22 mmol, HCl) and TEA (1.89 g, 18.7 mmol, 2.60 mL) in DCM (19.7 mL), 2,2,2-trifluoroethyl 2-chloro-2-oxo-acetate (1.30 g, 6.84 mmol, 887 µL) was added portion-wise at 0 °C and the resulting mixture was stirred overnight. H_2O (10 mL) was added to the reaction mixture and the organic layer was separated. The aqueous layer was extracted with DCM (10 mL) and combined organic layers were dried over Na_2SO_4, filtered and concentrated in vacuo to obtain 2,2,2-trifluoroethyl 2-oxo-2-[(2R)-2-phenyl-1-piperidyl]acetate (1.50 g, 4.76 mmol, 77% yield) as a yellow gum. LCMS (ESI): [M+H]+ m/z: calcd 316.12; found 316.0; Rt = 1.361 min.

(R)-2-oxo-2-(2-phenylpiperidin-1-yl)acetic acid, intermediate 12a
2,2,2-trifluoroethyl 2-oxo-2-[(2R)-2-phenyl-1-piperidyl]acetate (300 mg, 952 µmol) was dissolved in THF (5 mL) and H_2O (1 mL) was added. Lithium hydroxide monohydrate, 98% (79.9 mg, 1.90 mmol) was then added and the resulting mixture was stirred overnight. The reaction mixture was concentrated in vacuo and the residue was re-dissolved in H_2O (10 mL). The aqueous mixture was washed with MTBE (2 × 10 mL) and then acidified with $NaHSO_4$. The resulting mixture was extracted with DCM (2 × 10 mL) and combined organic layers were dried over Na_2SO_4, filtered, and concentrated in vacuo to obtain 2-oxo-2-[(2R)-2-phenyl-1-piperidyl]acetic acid, **intermediate 12a** (150 mg, 643 µmol, 67.6% yield) as a light-yellow solid. LCMS (ESI): [M+H]+ m/z: calcd 234.12; found 234.2; Rt = 0.813 min.

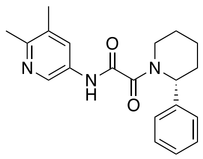

(R)-N-(5,6-dimethylpyridin-3-yl)-2-oxo-2-(2-phenylpiperidin-1-yl)acetamide (12).
DIPEA (186.68 µL, 1.07 mmol, 2.5eq) was added to the solution of 5,6-dimethylpyridin-3-amine (52.37 mg, 0.43 mmol) and **intermediate 12a** (0.1 g, 0.43 mmol) in DMF (10 mL) . The resulting mixture was stirred for 5 min followed by the addition of HATU (179.31 mg, 0.47 mmol, 1.1eq). The reaction mixture was stirred overnight at room temperature. The resulting suspension was concentrated under reduced pressure and the residue was purified by preparative 12 as a white solid (99.6 mg, 0.30 mmol, 69% yield). ^{1}H NMR (400 MHz, DMSO-d_6 + CCl_4) δ 1.44 (m, 2H), 1.62 (m, 2H), 1.82 (m, 1H), 2.23 (m, 3H), 2.37 (m, 3H), 2.98 (m, 2H), 3.67 (m, 1H), 5.68 (m, 1H), 7.33 (m, 3H), 7.41 (m, 2H), 7.86 (m, 1H), 8.53 (m, 1H), 10.97 (m, 1H). LCMS (ESI): [M]+ m/z: calcd 337.4; found 338.0; Rt = 3.808 min.

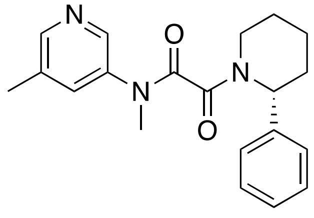

(R)-N-methyl-N-(5-methylpyridin-3-yl)-2-oxo-2-(2-phenylpiperidin-1-yl)acetamide (14).
N,5-dimethylpyridin-3-amine (100 mg, 630 µmol, HCl) was dissolved in DMF (5 mL) and DIPEA (407 mg, 3.15 mmol, 549 µL) was added, followed by **intermediate 12a** (147 mg, 630 µmol). HATU (360 mg, 946 µmol) was then added dropwise and the reaction mixture was stirred overnight. The reaction mixture was evaporated in vacuo and purified by HPLC (0-2-8 min 23-30-50% H_2O / MeOH / 0.1% NH_4OH 30 mL/min (loading pump 4 mL MeOH), target mass 337 column: XBRIDGE C18 100×19 mm, 5 µM) to obtain N-methyl-N-(5-methyl-3-

pyridyl)-2-oxo-2-[(2R)-2-phenyl-1-piperidyl]acetamide (10.3 mg, 30.5 µmol, 4.84% yield) as a yellow gum. ^{1}H NMR (600 MHz, DMSO-d_6) δ 1.29 (d, 2H), 1.52 (t, 3H), 2.28 (m, 1H), 2.30 (m, 3H), 2.33 (s, 1H), 2.50 (m, 2H), 3.40 (m, 2H), 5.34 (m, 1H), 7.24 (m, 5H), 7.72 (m, 1H), 8.43 (dd, 1H), 8.53 (dd, 1H). LCMS (ESI): [M+H]+ m/z: calcd 338.22; found 338.4; Rt = 3.084 min.

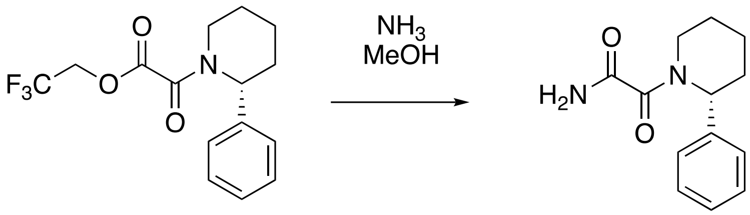

intermediate 13a

(R)-2-oxo-2-(2-phenylpiperidin-1-yl)acetamide, intermediate 13a
2,2,2-trifluoroethyl 2-oxo-2-[(2R)-2-phenyl-1-piperidyl]acetate (1.50 g, 4.76 mmol) was dissolved in MeOH (5 mL) and NH_3/MeOH (15 mL) was added. The resulting solution was stirred overnight. The reaction mixture was concentrated in vacuo to obtain 2-oxo-2-[(2R)-2-phenyl-1-piperidyl]acetamide, **intermediate 13a** (1.10 g, 4.74 mmol, 99.5% yield) as a beige solid. LCMS (ESI): [M+H]+ m/z: calcd 233.13; found 233.0; Rt = 1.042 min.

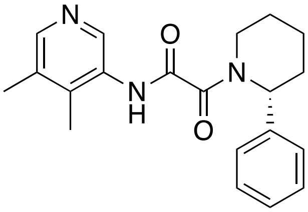

(R)-N-(4,5-dimethylpyridin-3-yl)-2-oxo-2-(2-phenylpiperidin-1-yl)acetamide (13).
To an 8 mL vial **intermediate 13a** (150 mg, 646 µmol), 3-bromo-4,5-dimethyl-pyridine (120 mg, 646 µmol), copper (2.05 mg, 32.3 µmol), copper (I) iodide (61.5 mg, 323 µmol, 10.9 µL), (1R,2R)-N1,N2-dimethylcyclohexane-1,2-diamine (68.9 mg, 484 µmol), cesium carbonate (421 mg, 1.29 mmol) and dioxane (5 mL) were charged. The resulting mixture was purged with Ar for 5 min. Then the vial was sealed and heated at 100 °C for 16 h. The reaction mixture was cooled and filtered. The filter cake was rinsed with DCM (10 mL) and the filtrate was concentrated in vacuo. The resulting crude product was stirred with SiliaMets (1 g) in MeOH (5 mL) for 12 h at rt. The suspension was filtered, the precipitate was washed with additional MeOH (2 × 2 mL) and solvent was evaporated. The residue was performed to HPLC (0-2-8 min 17-25-45% H_2O / ACN / 0.1% NH_4OH 30 mL/min (loading pump 4 mL ACN), target mass 337 column: XBRIDGE C18 100×19 mm, 5 µM) to obtain N-(4,5-dimethyl-3-pyridyl)-2-oxo-2-[(2R)-2-phenyl-1-piperidyl]acetamide (104 mg, 307 µmol, 48% yield) as a brown solid.
^{1}H NMR (600 MHz, DMSO-d_6) δ 1.53 (m, 4H), 1.86 (m, 1H), 2.07 (d, 3H), 2.22 (d, 3H), 2.45 (m, 1H), 2.94 (dd, 1H), 4.00 (m, 1H), 5.52 (m, 1H), 7.35 (m, 5H), 8.23 (m, 2H), 10.57 (d, 1H). LCMS (ESI): [M+H]+ m/z: calcd 338.22; found 338.2; Rt = 2.681 min.

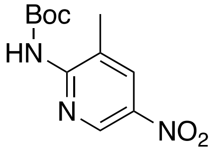

Tert-butyl (3-methyl-5-nitropyridin-2-yl)carbamate (50).
To a solution of 3-methyl-5-nitro-pyridin-2-amine **49** (60 g, 391.8 mmol, 1eq) in DMF (525 mL), sodium hydride, 60% dispersion in mineral oil (16.5 g, 412.88 mmol, 1.05eq) was added portionwise at 0 °C. The resulting mixture was stirred for 0.5 h and a solution of di-tertbutyl decarbonate (89.8 g, 411.4 mmol, 1eq) in DMF (75 mL) was added dropwise. The resulting mixture was stirred at 25 °C for 18 h. The mixture was quenched with H_2O (1000 mL), the precipitate was filtered off and dried in vacuo to obtain 100g crude product which was purified by column chromatography ($CHCl_3$: MTBE as eluent) to obtain **50** as a white powder (46 g, 181.64 mmol, 46% yield). ^{1}H NMR (400 MHz, DMSO-*d6*): δ 1.49 (s, 9H), 2.32 (s, 3H), 8.37 (s, 1H), 8.97 (s, 1H), 9.59 (s, 1H). LCMS (ESI): [M-$CH_2C(CH_3)$+H]+ m/z: calcd 197.04; found 198.2; Rt = 1.272 min.

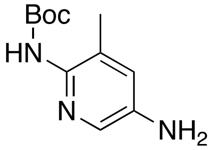

Tert-butyl (5-amino-3-methylpyridin-2-yl)carbamate (51).
To a solution of **50** (46 g, 181.64 mmol, 1eq) in MeOH (600 mL) was added 10% palladium on activated carbon (4.6 g, 43.22 mmol, 0.24eq). The resulting mixture was stirred under H_2 atmosphere for 24 h. The catalyst was filtered and the solvent was removed in vacuo. The residue was dissolved in DCM (500 mL), dried over sodium sulfate, and evaporated in vacuo to obtain **51** as a white solid (38 g, 170.2 mmol, 94% yield). ^{1}H NMR (400 MHz, DMSO- *d6*): δ 1.40 (s, 9H), 2.03 (s, 3H), 3.30 (brs, 2H), 6.80 (s, 1H), 7.54 (s, 1H), 8.52 (s, 1H). LCMS (ESI): [M+H]+ m/z: calcd 223.2; found 224.2; Rt = 0.67 min.

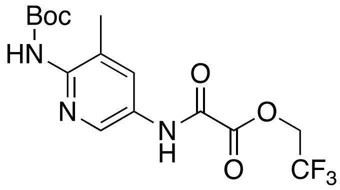

2,2,2-trifluoroethyl 2-((6-((tert-butoxycarbonyl)amino)-5-methylpyridin-3-yl)amino-2-oxoacetate (52).
To a solution of **51** (17.6 g, 78.83 mmol, 1 eq) and diisopropylethylamine (20.60 mL, 118.24 mmol, 1.5 eq) in ACN (250 mL) was added 2,2,2-trifluoroethyl 2-chloro-2-oxoacetate (17.27 g, 90.65 mmol, 1.15 eq) dropwise at 0 °C under argon. The reaction mixture was then stirred for 24 h at 20 °C, then the solvent was evaporated in vacuo and the residue was diluted with H_2O (575 mL). The precipitate was filtered off, washed with H_2O and dried in vacuo to provide **52** (30 grams, 79.5 mmol, 100% yield) which was used without further purification. ^{1}H NMR (DMSO- *d6*, 400 MHz): δ 1.42 (s, 9H), 2.15 (s, 3H), 4.96 (q, 2H), 7.93 (s, 1H), 8.49 (s, 1H), 9.03 (s, 1H), 11.06 (s, 1H). LCMS (ESI): [M-$CH_2C(CH_3)$+H]+ m/z: calcd 321.06; found 322.0; Rt = 1.274 min.

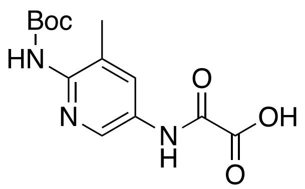

2-((6-((tert-butoxycarbonyl)amino)-5-methylpyridin-3-yl)amino)-2-oxoacetic acid (69).
A mixture of **52** (30 g, 79.51 mmol, 1 eq) and lithium hydroxide, monohydrate (6.67 g, 159.02 mmol, 2 eq) in THF (120 mL), MeOH (120 mL), H_2O (120 mL) was stirred at 5 °C. After 2 h the volatile organic solvents were removed under reduced pressure. The residue was acidified with sodium hydrogen sulfate, monohydrate (21.96 g, 159.02 mmol, 2 eq) to pH 5 and the precipitate was filtered off, washed with H_2O and dried in vacuo to provide **69** (23 g, 77.89 mmol, 98% yield). ^{1}H NMR(DMSO-*d6*, 400 MHz): δ 1.44 (s, 9H), 2.16 (s, 3H), 7.97 (s, 1H), 8.51 (s, 1H), 8.98 (s, 1H), 10.70 (s, 1H). LCMS (ESI): [M-$CH_2C(CH_3)$+H]+ m/z: calcd 295.29; found 240.0; Rt = 0.829 min.

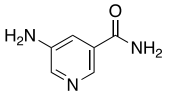

5-aminonicotinamide (54).
To a stirred solution of **53** (50 g, 361.99 mmol, 1 eq) in MeOH (500 mL), thionyl chloride (47.37 g, 398.19 mmol, 1.1 eq) was added dropwise at 25 °C. The reaction mixture was refluxed for 16 h (70 °C), then the resulting mixture was allowed to cool to room temperature, concentrated in vacuo, and the residue was diluted with H_2O (500 mL). The obtained solution was neutralized with the aqueous solution of $NaHCO_3$. The aqueous mixture was extracted with DCM (3x600 mL). The combined organic layers were dried over anhydrous Na_2SO_4, filtered off, and the filtrate was concentrated in vacuo to dryness to afford methyl 5-aminonicotinate (49 g, 322.05 mmol, 89% yield). ^{1}H NMR (500 MHz, $CDCl_3$) δ 3.86 (s, 2H), 3.93 (s, 3H), 7.57 (s, 1H), 8.26 (s, 1H), 8.65 (s, 1H).
Methyl 5-aminonicotinate (49 g, 322.05 mmol, 1 eq) was dissolved in 300 mL of aqueous solution of NH_3 and stirred for 16 h. The resulting mixture was evaporated to dryness to afford **54** (43 g, 313.55 mmol, 97% yield). ^{1}H NMR (500 MHz, DMSO-*d6*) δ 5.46 (s, 2H), 7.29 (s, 2H), 7.91 (s, 1H), 8.01 (s, 1H), 8.18 (s, 1H).

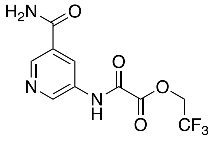

2,2,2-trifluoroethyl 2-((5-carbamoylpyridin-3-yl)amino)-2-oxoacetate (55).
To a solution of **54** (0.65g, 4.74 mmol, 1 eq) and DIPEA (1.24 mL, 7.11 mmol, 1.5 eq) in dry THF (15 mL), 2,2,2-trifluoroethyl 2-chloro-2-oxo-acetate (993mg, 5.21 mmol, 1.1 eq) was added dropwise at 0 ^{o}C. The reaction mixture was stirred for 12 h at room temperature and then filtered. The filtrate was concentrated under vacuum to obtain **55** (1.7g, crude). The crude product was used in the next step without further purification. LCMS (ESI): [M+H]+ m/z calcd 291.0; found 292.2; Rt=0.815 min

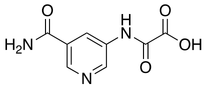

2-((5-carbamoylpyridin-3-yl)amino)-2-oxoacetic acid (56).
To a suspension of **55** (1.1 g, 3.78 mmol, 1 eq) and lithium hydroxide monohydrate (174mg, 4.16mmol, 1.1 eq) were stirred in THF (10 mL) and H_2O (2.5 mL) overnight. The mixture was then diluted with 10 mL THF and the precipitate was filtered and air dried to afford **56** as a white solid (0.74g, 3.44mmol, 91% yield). ^{1}H NMR (400 MHz, DMSO-d_6) δ 7.56 (s,1H), 8.12 (s, 1H), 8.66 (m, 2H), 8.98 (s, 1H), 10.58 (m, 1H).

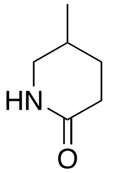

5-methylpiperidin-2-one (58).
To a solution of 5-methyl-1H-pyridin-2-one **57** (25 g, 0.229 mol, 1 eq) in MeOH (200 mL) was added 10% palladium on carbon (4 g, 10 wt% of Pd with 50 wt% H_2O) under N_2 atmosphere. The suspension was degassed and purged with H_2 3 times. The mixture was stirred under H_2 (50 psi) at 80 °C for 24 h. The mixture was filtered, and the filtrate was concentrated under reduced pressure to give **58** (26 g, crude) as a colorless oil, which was directly used without further purification.

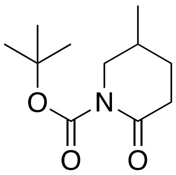

tert-butyl 5-methyl-2-oxopiperidine-1-carboxylate (59).
To a solution of **58** (26 g, 0.230 mol, 1 eq) in DCM (100 mL) was added TEA (96 mL, 0.689 mol, 3 eq) and DMAP (28 g, 0.229 mol, 1 eq). Then di-tert-butyl dicarbonate (106 mL, 0.461 mol, 2 eq) was added slowly. The mixture was stirred at 20 °C for 12 h and the resulting mixture was quenched by addition of H_2O (100 mL). The organic layer was separated, and the aqueous phase was extracted with DCM (100 mL x 2). The combined organic layer was washed with saturated NH_4Cl aqueous solution (500 mL x 3), dried over anhydrous Na_2SO_4, filtered and concentrated under reduced pressure to give a crude product, which was purified by flash chromatography (ISCO®; 220 g AgelaFlash® Silica Flash Column, petroleum ether/EtOAc with EtOAc from 0-10%, 100 mL/min) to afford **59** (44 g, 90% yield) as a colorless oil. ^{1}H NMR (400 MHz, MeOD-d_4) δ 3.79 (ddd, J = 12.5, 4.8, 1.8 Hz, 1H), 3.16 (dd, J = 12.4, 10.4 Hz, 1H), 2.42 - 2.57 (m, 2H), 1.83 - 2.01 (m, 2H), 1.51 (s, 9H), 1.46 (dd, J = 6.7, 3.6 Hz, 1H), 1.04 (d, J = 6.5 Hz, 3H); LCMS (ESI) $[M+H-Bu]^+$ m/z: calcd 158.1, found 157.8.

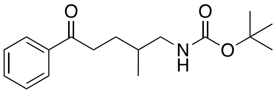

tert-butyl (5-oxo-5-phenylpentyl)carbamate (60).

To a solution of **59** (30 g, 140.66 mmol, 1eq) in tetrahydrofuran (300 mL) was added phenylmagnesium bromide (260.25 mL, 211.00 mmol, 1.5 eq) dropwise at -78 °C over 1 h. The mixture was warmed to room temperature and quenched by addition of saturated NH_4Cl aqueous solution and then extracted with DCM (50 mL x 3). The combined organic layer was dried over anhydrous Na_2SO_4, filtered and concentrated under reduced pressure to give a crude product **60**, as a light yellow oil which was used in the next step as is.(10 g, 73% yield) as a yellow oil. LCMS (ESI) $[M+H-Boc]^+$ m/z: calcd 191.2, found 192.2; Rt=1.486. ^1H NMR (500 MHz, $CDCl_3$) δ 0.96 (d, 3H), 1.45 (s, 9H), 1.68 (m, 1H), 1.72 (m, 1H), 1.85 (m, 1H), 3.05 (m, 4H), 4.71 (m, 1H), 7.45 (t, 2H), 7.55 (t, 1H), 7.96 (d, 2H).

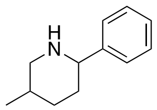

5-methyl-2-phenylpiperidine (61).
To a solution of **60** (41 g, 140.7 mmol, 1 eq) was added TFA (54.20 mL, 703.54 mmol, 5 eq). The mixture was stirred at 20 °C for 1 h. 50% w/v NaOH solution was added to the mixutre until the pH was 13-14. The product was extracted with 4 X 20 mL DCM and the organic layers were combined, dried with $MgSO_4$ and evaporated. The material was dissolved in a mixture of MeOH (500mL)/H_2O (100mL) and added to a flask followed by $NaBH_4$ (5.32g, 140.71 mmol, 1 eq) and the reaction mixture was stirred at 20 °C overnight. The resulting mixture was quenched by addition of 2N HCl/H_2O until pH to 1-3. The mixture was extracted with DCM (30 mL). The aqueous solution was adjusted to pH 13-14 with 50% NaOH aqueous solution and extracted with DCM (30 mL x 3) and these washes were combined and dried over anhydrous Na_2SO_4, filtered, and concentrated under reduced pressure to give **61** (21.2 g, crude). ^1H NMR (400 MHz, $CDCl_3$) δ 0.87 (d, 3H), 1.14 (m, 1H), 1.56 (m, 2H), 1.81 (m, 3H), 2.40 (t, 1H), 3.11 (d, 1H), 3.53 (d, 1H), 7.29 (m, 5H).; LCMS (ESI) $[M+H]^+$m/z: calcd 175.2; found 176.2. Rt =0.779min.

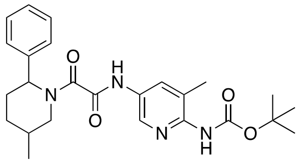

tert-butyl (3-methyl-5-(2-(5-methyl-2-phenylpiperidin-1-yl)-2-oxoacetamido)pyridin-2-yl)carbamate (62).
To a solution of **61** (222.97 mg, 1.27 mmol, 1 eq) in THF (15 mL) was added butyllithium (2.4 mL, 3.3 eq) dropwise at -78 °C under argon. The reaction mixture was stirred at -78 °C for 20 min, then **52** (0.48 g, 1.27 mmol, 1 eq) was added portion-wise. The resulting solution was stirred at -78 °C for 30 min. and at room temperature for 1 h. The resulting solution was cooled to -50 °C and quenched with saturated NH_4Cl (aq) (30 ml). The organic layer was separated, and the aqueous layer was extracted with EtOAc (20 mL × 2). The combined organic layers were washed with brine, dried over Na_2SO_4 and concentrated to dryness to give **62** (0.54 g crude, 1.19 mmol, 94%). ^1H NMR (400 MHz, $CDCl_3$) δ 0.87 (d, 3H), 1.16 (m, 4H), 1.48 (s, 9H), 1.62 (m, 2H), 1.82 (m, 2H), 2.27 (m, 2H), 3.72 (m, 2H), 6.68 (m, 1H), 7.19 (m, 5H), 8.04 (m, 1H). LCMS (ESI): $[M+H]^+$ m/z: calcd 452.2; found 453.2; Rt = 1.419 min.

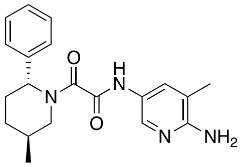

N-(6-amino-5-methylpyridin-3-yl)-2-((2R,5S)-5-methyl-2-phenylpiperidin-1-yl)-2-oxoacetamide (23).
4.0M hydrogen chloride solution in dioxane (5.5 mL, 21.94 mmol, 18.5 eq) was added to a solution of **62** (0.54 g, 1.19 mmol, 1eq) in DCM (10 mL). The reaction mixture was stirred at 25 °C for 6 h, then evaporated in vacuo to obtain 0.4 g crude product which was purified by prep HPLC (50-75% MeOH/H_2O over 0-10.5 min, flow 30 mL/min) to afford racemic product (0.167 g, 0.43 mmol, 36% yield, HCl salt). Chiral separation of 22.0 mg (0.062 mmol) was performed using OJ-H Chiralpak column, Hexane-IPA-MeOH as a solvent mixture, 70-15-15, Flow rate=15 ml/min, to afford the following isomers as yellow solids: N-(6-amino-5-methyl-3-pyridyl)-2-[(2S,5S)-5-methyl-2-phenyl-1-piperidyl]-2-oxo-acetamide (3.4 mg, 9.65 umol, 15% yield; RT = 17.308 min), N-(6-amino-5-methyl-3-pyridyl)-2-[(2R,5R)-5-methyl-2-phenyl-1-piperidyl]-2-oxo-acetamide (2.7 mg, 7.66 umol, 12% yield; RT = 42.900 min), N-(6-amino-5-methyl-3-pyridyl)-2-[(2S,5R)-5-methyl-2-phenyl-1-piperidyl]-2-oxo-acetamide (7.8 mg, 22.13 umol, 35% yield; RT = 49.446 min), and N-(6-amino-5-methylpyridin-3-yl)-2-((2R,5S)-5-methyl-2-phenylpiperidin-1-yl)-2-oxoacetamide **23** (7.8 mg, 22.13 umol, 35% yield; RT = 22.466 min), ^{1}H NMR (500 MHz, $CDCl_3$) δ 1.11 (d, 3H), 1.41 (m, 1H), 1.90 (m, 2H), 2.15 (m, 3H), 2.23 (m, 2H), 3.26 (m, 1H), 4.62 (m, 3H), 5.82 (m, 1H), 7.30 (m, 3H), 7.38 (m, 2H), 7.76 (s, 1H), 8.04 (m, 1H), 9.08 (s, 1H). LCMS (ESI): $[M+H]^+$ m/z: calcd 352.2; found 353.0; Rt = 3.821 min, RT (OJ-H, Hexane-IPA-MeOH, 70-15-15, 0.6 mL/min) = 23.187 min.

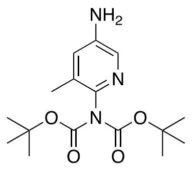

tert-butyl (5-amino-3-methylpyridin-2-yl)(tert-butoxycarbonyl)carbamate (63).
To a solution of 3-methyl-5-nitro-pyridin-2-amine, **49** (10 g, 65.30 mmol, 1 eq) and DMAP (8 g, 65.48 mmol, 1 eq) in DCM (50 mL) was added tert-butoxycarbonyl tert-butyl carbonate (28.53 g, 130.72 mmol, 2 eq). The mixture was stirred at 20 °C for 12 h. The resulting mixture was concentrated under reduced pressure. The residue was diluted with EtOAc (300 mL), washed with saturated NH_4Cl aqueous solution (30 mL x 2), dried over anhydrous Na_2SO_4, filtered and concentrated under reduced pressure to afford tert-butyl N-tert-butoxycarbonyl-N-(3-methyl-5-nitro-2-pyridyl)carbamate (23 g, 65.09 mmol, 100% yield) as yellow solid. LCMS (ESI) $[M+H]^+$ m/z: calcd 353.2; found 198.1($[M+H-156]^+$). To a solution of tert-butyl N-tert-butoxycarbonyl-N-(3-methyl-5-nitro-2-pyridyl)carbamate (23 g, 65.09 mmol, 1 eq) in MeOH (100 mL) was added 10 wt% palladium on carbon (1 g, 50 wt % H_2O). The mixture was degassed and backfilled with H_2 three times and stirred at 20 °C for 24 h under H_2 atmosphere (15 psi). The resulting mixture was filtered and concentrated under reduced pressure to afford **63** (20 g, 61.85 mmol, 95% yield) as yellow solid. LCMS (ESI) $[M+H]^+$ m/z: calcd 324.1; found 324.1.

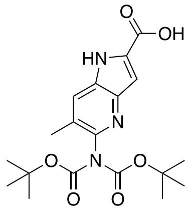

5-(bis(tert-butoxycarbonyl)amino)-6-methyl-1H-pyrrolo[3,2-b]pyridine-2-carboxylic acid (64).

63 (20 g, 61.85 mmol, 1 eq) in acetic acid (100 mL) was added N-iodosuccinimide (15 g, 66.67 mmol, 1.08 eq) . The mixture was stirred at 20 °C for 1 h. The resulting mixture was concentrated under reduced pressure. The residue was quenched by addition of $NaHCO_3$ aqueous solution (500 mL) and extracted with EtOAc (200 mL x 3). The combined organic layer was washed with brine (100 mL), dried over anhydrous Na_2SO_4, filtered and concentrated under reduced pressure. The residue was purified by flash chromatography (ISCO®; 80 g AgelaFlash® Silica Flash Column, petroleum ether/EtOAc with EtOAc from 0-15%, Flow rate: 30 mL/min) to afford tert-butyl N-(5-amino-6-iodo-3-methyl-2-pyridyl)-N-tert-butoxycarbonyl-carbamate (25 g, crude) as yellow oil. LCMS (ESI) $[M+H]^+$ m/z: calcd 450.1; found 350.0$[M+H-100]^+$. To the crude material was added 2-oxopropanoic acid (4.23 g, 47.98 mmol), $Pd(OAc)_2$ (1.25 g, 5.57 mmol), triphenylphosphine (2.92 g, 11.12 mmol) and DMF (80 mL). The mixture was degassed and backfilled with N_2 three times and then stirred for at 100 °C under nitrogen. The resulting mixture was concentrated under reduced pressure. The residue was dissolved in H_2O (300 mL), adjusted to pH=10 with saturated NaOH aqueous solution and washed with EtOAc (300 mL x 3).The aqueous phase was adjusted to pH=5 with saturated citric acid aqueous solution, filtered, and the filter cake was washed with H_2O (5 mL x 3), dried under reduced pressure to afford **64** (3.5 g, 8.94 mmol, 14% yield, 2 steps) as yellow solid. ^1H NMR (400 MHz, DMSO-*d6*) δ 12.02 (1 H, s), 7.72 (1 H, s), 7.09 (1 H, d, J=1.25 Hz), 3.41 - 3.48 (1 H, m), 2.24 (3 H, s), 1.34 (18 H, s). LCMS (ESI) $[M+H]^+$ m/z: calcd 392.2; found 392.1.

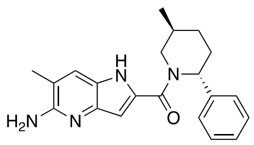

(5-amino-6-methyl-1H-pyrrolo[3,2-b]pyridin-2-yl)((2R,5S)-5-methyl-2-phenylpiperidin-1-yl)methanone (25).

To a solution of **64** (300 mg, 0.766 mmol, 1 eq) and HATU (350 mg, 0.921 mmol, 1.2 eq) in DCM (20 mL) were added DIEA (0.4 mL, 2.30 mmol) and **61** (162 mg, 0.924 mmol, 1.2 eq). The mixture was stirred at 20 °C for 1 h. The mixture was concentrated under reduced pressure to give a crude product, which was purified by flash chromatography (ISCO®; 25 g SepaFlash® Silica Flash Column, petroleum ether/EtOAc with EtOAc from 0-45%, 35 mL/min) to afford tert-butyl N-tert-butoxycarbonyl-N-[6-methyl-2-(5-methyl-2-phenyl-piperidine-1-carbonyl)-1H-pyrrolo[3,2-b]pyridin-5-yl]carbamate (80 mg, 19% yield) as a yellow solid. LCMS (ESI) $[M+H]^+$ m/z: calcd 549.3, found 549.2. To a solution of tert-butyl N-tert-butoxycarbonyl-N-[6-methyl-2-(5-methyl-2-phenyl-piperidine-1-carbonyl)-1H-pyrrolo[3,2-b]pyridin-5-yl]carbamate (170 mg, 0.310 mmol) in DCM (5 mL) was added TFA (2.4 mL). The mixture was stirred at 20 °C for 2 h then adjusted to pH 8 with saturated $NaHCO_3$ aqueous solution, and concentrated under reduced pressure to give a crude product, which was purified by flash chromatography (ISCO®; 25 g SepaFlash® Silica Flash Column, DCM/MeOH with MeOH from 0-5%, 35 mL/min) to give (5-amino-6-methyl-1H-pyrrolo[3,2-b]pyridin-2-yl)-(5-methyl-2-phenyl-1-piperidyl)methanone (105 mg, 97% yield) as a brown solid. LCMS (ESI) $[M+H]^+$ m/z: calcd 349.2, found 349.3. This material was separated by chiral SFC (Instrument: Thar800Q; Column: Daicel Chiralpak IG 250 ×30 mm I.D. 10 μm; Mobile phase: supercritical CO_2/EtOH (0.1% NH_3-H_2O, v%) = 60/40; Flow Rate: 80 mL/min; Column Temperature: 38 °C; Nozzle Pressure: 100 bar; Nozzle Temperature: 60 °C; Evaporator Temperature: 20 °C; Trimmer Temperature: 25 °C; Wavelength: 220 nm) to afford **25** (25.0 mg, retention time = 9.533 min, white dry powder). ^1H NMR (400 MHz, MeOD-d_4) *δ* 7.50 (s, 1H), 7.35 - 7.46 (m, 4H), 7.26 - 7.32 (m, 1H), 6.46 (s, 1H), 5.85 (t, *J* = 3.8 Hz, 1H), 4.27 (d, *J* = 13.6 Hz, 1H), 3.34 (d, *J* = 3.5 Hz, 1H), 2.25 - 2.30 (m, 2H), 2.24 (s, 3H), 1.92 - 2.02 (m, 1H), 1.78 - 1.90 (m, 1H), 1.37 - 1.46 (m, 1H), 1.10 (d, *J* = 7.0 Hz, 3H); LCMS (ESI) $[M+H]^+$ m/z: calcd 349.3, found 349.3; 100%ee, Rt=2.921.

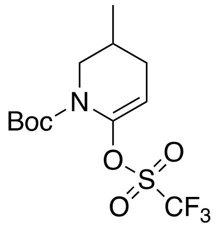

tert-butyl 3-methyl-6-(((trifluoromethyl)sulfonyl)oxy)-3,4-dihydropyridine-1(2H)-carboxylate (65).
Lithium bis(trimethylsilyl)amide (480.56 g, 574.38 mmol, 20% purity, 1.25 eq) was added dropwise under argon to a cooled to -78°C solution of **59** (98 g, 459.51 mmol, 1 eq) in THF (500 mL) . The resulting solution was stirred at -78 °C for 1.5 h, then 1,1,1-trifluoro-N-phenyl-N-(trifluoromethylsulfonyl)methanesulfonamide (188.78 g, 528.43 mmol, 1.15 eq) was added in one portion. The reaction mixture was allowed to warm to 20 °C and stirred for 12 h, then diluted with H_2O (300 mL) and MTBE (700 mL). The organic layer was separated, the aqueous layer was extracted with MTBE (300 mL). The combined organic extracts were washed with 10% aqueous sodium hydroxide solution (3 x 300 mL), dried over potassium carbonate and evaporated in vacuo. The residue was diluted with hexane/MTBE mixture and stirred for 0.5 h. The resulting cloudy solution was decanted from an oily residue, filtered through a short pad of silica gel and evaporated in vacuo to afford **65** (150 g, 434.36 mmol, 95% yield) as light-yellow oil which was uses as is.

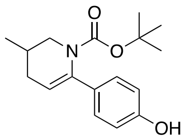

tert-butyl 6-(4-hydroxyphenyl)-3-methyl-3,4-dihydropyridine-1(2H)-carboxylate (66).
65 (32 g, 92.66 mmol, 1 eq), (4-hydroxyphenyl)boronic acid (15.98 g, 115.83 mmol, 1.25 eq) and sodium carbonate (29.46 g, 277.99 mmol, 3 eq) were added to a mixture of 1,4-dioxane (360 mL) and H_2O (120 mL) . The resulting mixture was evacuated and then backfilled with Ar three times then Pd(dppf)Cl_2•DCM (3.02 g, 3.71 mmol, 0.04 eq) was added under Ar. The reaction mixture was stirred under Ar at 90 °C for 18 h, then cooled and filtered. The filter cake was washed with 1,4-dioxane (2 X 20 mL) and discarded. The filtrate was evaporated in vacuo and the residue was purified by column chromatography on silica gel using hexane/EtOAc gradient (0-100% EtOAc) to afford **66** (12 g, 41.47 mmol, 45% yield) as white solid. ^{1}H NMR (500 MHz, $CDCl_3$) δ 0.99 (d, 3H), 1.12 (s, 9H), 1.99 (m, 1H), 2.00 (m, 1H), 2.36 (d, 1H), 2.96 (t, 1H), 4.06 (d, 1H), 5.22 (m, 1H), 6.77 (m, 2H), 7.15 (m, 2H). LCMS (ESI): $[M-Boc]^+$ m/z: calcd 189.2; found 190.2; Rt = 1.395 min.

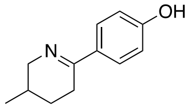

4-(5-methyl-3,4,5,6-tetrahydropyridin-2-yl)phenol (67).
66 (12 g, 41.47 mmol, 1 eq) was dissolved in TFA (95.85 mL). The resulting solution was stirred at 25 °C for 1 h, then evaporated in vacuo. Crushed ice (100 g) was added to the residue and adjusted to pH 9 with 10% aqueous sodium carbonate solution. The resulting mixture was extracted with DCM (2 x 300 mL). The combined organic extracts were dried over sodium sulphate and evaporated in vacuo to afford **67** (8.5 g, crude) as beige solid, which was used directly in the next step. ^{1}H NMR (400 MHz, $CDCl_3$) δ 0.98 (d, 3H), 1.32 (m, 1H), 1.67 (m, 1H), 1.83 (m, 1H), 2.56 (m, 1H), 2.74 (m, 1H), 3.11 (m, 1H), 3.80 (m, 1H), 6.50 (m, 2H), 7.42 (m, 2H), 10.89 (bds, 1H). LCMS (ESI): $[M]^+$ m/z: calcd 189.2; found 190.2; Rt = 0.730 min.

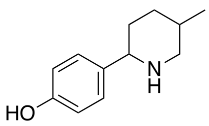

4-((2R,5S)-5-methylpiperidin-2-yl)phenol (68).
Sodium borohydride (2 g, 52.87 mmol, 1.17 eq) was added in one portion to a stirred solution of **67** (8.5 g, 44.91 mmol, 1 eq) in MeOH (100 mL) at 0°C. The resulting mixture was stirred at 0 °C for 1 h then evaporated in vacuo. The residue was dissolved in MeOH (150 mL) and 4.0M hydrogen chloride solution in dioxane (117.81 g, 449.13 mmol, 112.20 mL, 14% purity) was added. The resulting cloudy solution was evaporated in vacuo, the residue was diluted with THF (150 mL) and stirred for 0.5 h. The precipitate was filtered, washed with THF (2 x 50 mL), and dried in vacuo to afford **68** (7.35 g, crude, HCl salt) as light-yellow solid, which was used directly in the next step. ^{1}H NMR (500 MHz, DMSO-d_6) δ 0.92 (d, 3H), 1.28 (m, 1H), 1.95 (m, 3H), 2.11 (m, 1H), 2.65 (m, 1H), 3.12 (m, 1H), 3.99 (m, 1H), 6.81 (m, 2H), 7.40 (m, 2H), 9.30 (m, 1H), 9.70 (m, 2H). LCMS (ESI): $[M]^+$ m/z: calcd 191.2; found 192.2; Rt = 0.732 min.

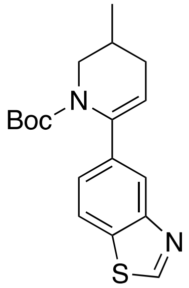

tert-butyl 6-(benzo[d]thiazol-5-yl)-3-methyl-3,4-dihydropyridine-1(2H)-carboxylate (70).
A mixture of **65** (7.72 g, 22.36 mmol, 1 eq), 5-(4,4,5,5-tetramethyl-1,3,2-dioxaborolan-2-yl)-1,3-benzothiazole (7.3 g, 27.95 mmol, 1.25 eq), [1,1′-Bis(diphenylphosphino)ferrocene]dichloropalladium(II) complex with DCM (913 mg, 1.12 mmol, 0.05 eq) and sodium carbonate (7.11 g, 67.09 mmol, 3 eq) in dioxane (120 mL) and H_2O (40 mL) was stirred at 80 °C under Ar atmosphere for 18 h. After cooling to room temperature, the reaction mixture was filtered. The filter cake was washed with dioxane (500 mL) and discarded. The filtrate was concentrated under reduced pressure and the residue was purified by silica gel flash chromatography eluting with a 0 - 100 % MTBE-Hexane gradient to give **70** (5.9 g, 17.85 mmol, 80% yield). ^{1}H NMR (DMSO-d_6, 500 MHz): δ 0.95 – 1.03 (m, 12H), 1.86 (m, 1H), 1.90 (s, 1H), 2.50 (m, 1H), 3.0 (t, 1H), 3.97 (d, 1H), 5.41 (s, 1H). 7.37 (d, 1H), 7.78 (s, 1H), 7.99 (d, 1H), 9.27 (s, 1H). LCMS (ESI): [M+H]+ m/z: calcd 330.2; found 331.2; Rt = 1.435 min.

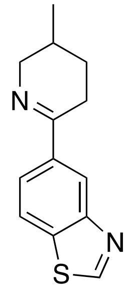

5-(5-methyl-3,4,5,6-tetrahydropyridin-2-yl)benzo[d]thiazole (71).
70 (5.9 g, 17.85 mmol, 1 eq) was stirred in TFA (22 mL) at 20 °C for 1 h, then evaporated in vacuo. Crushed ice (10 g) was added to the residue and adjusted to pH 8 with a 10% aqueous solution of sodium hydroxide. The resulting mixture was extracted with ethyl acetate (2 x 30 mL). The combined organic extracts were dried over sodium sulfate and evaporated in vacuo to afford **71** (4.1 g, 17.80 mmol, 100% yield) as yellow solid, which was used directly in the next step. ^{1}H NMR (DMSO-d_6, 500 MHz): δ 0.95 (m, 3H), 1.35 (m, 1H), 1.65 (m, 1H), 1.89 (m, 1H), 2.67 (m, 1H), 2.87 (d, 1H), 3.19 (t, 1H). 3.95 (d, 1H), 8.02 (d, 1H), 8.14 (d, 1H), 8.43 (s, 1H), 9.41 (s, 1H). LCMS (ESI): [M+H]+ m/z: calcd 230.1; found 231.2; Rt = 0.828 min.

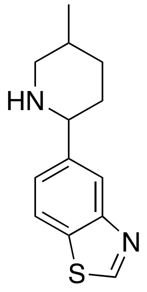

rac-5-((2R,5S)-5-methylpiperidin-2-yl)benzo[d]thiazole (72).
Sodium borohydride (1.01 g, 26.70 mmol, 1.5 eq) was added in one portion to a stirred solution of **71** (4.1 g, 17.80 mmol, 1 eq) in MeOH (90 mL) at 0 °C. The resulting mixture was stirred for 1 h, then evaporated in vacuo. The residue was diluted with H_2O (50 mL) and extracted with DCM (2 x 75 mL). The combined organic extracts were dried over sodium sulfate and evaporated in vacuo to afford **72** (4.1 g, 17.65 mmol, 99% yield) as yellow oil, which was used directly in the next step. ^{1}H NMR(DMSO-d_6, 400 MHz): δ 0.82(d, 3H), 1.05 (m, 1H), 1.34 (m, 1H), 1.52 (m, 1H), 1.75 (m, 2H), 2.26 (t, 1H), 3.00 (d, 1H). 3.61 (d, 1H), 7.46 (d, 1H), 8.03 (m, 2H), 9.31 (s, 1H). LCMS (ESI): [M+H]+ m/z: calcd 232.1; found 233.0; Rt = 0.691 min.

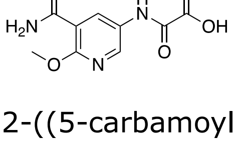

2-((5-carbamoyl-6-methoxypyridin-3-yl)amino)-2-oxoacetic acid (74).
To a solution of 5-amino-2-methoxy-pyridine-3-carboxamide **73** (10.06 g, 60.18 mmol, 1 eq) and TEA (8.39 mL, 60.18 mmol, 1 eq) in dry THF (250 mL) was added 2,2,2-trifluoroethyl 2-chloro-2-oxo-acetate (12.04 g, 63.19 mmol, 1.05 eq) in 50 mL dry THF at 0 °C. After stirring

at room temperature for 12 h the resulting mixture was evaporated to dryness to give 2,2,2-trifluoroethyl 2-[(5-carbamoyl-6-methoxy-3-pyridyl)amino]-2-oxo-acetate (29 g, crude) as a light-pink solid, which was used in the next step without further purification. ^{1}H NMR (DMSO-d_6, 400 MHz): δ 3.97 (s, 3H), 5.00 (q, 2H), 7.75 (brs, 2H), 8.55 (s, 1H), 8.63 (s, 1H), 11.21 (s, 1H). To a solution of this material (15 g, 46.70 mmol, 1 eq) in MeOH (400 mL) was added lithium hydroxide monohydrate (3.92 g, 93.40 mmol, 2 eq) and the resulting mixture was left to stir at room temperature for 1 h, after which the resulting mixture was evaporated to dryness and dissolved in H_2O which was then acidified to pH=1 with aqueous hydrochloric acid. The precipitate was filtered and then suspended in MeOH and TEA (13.02 mL, 93.40 mmol, 2 eq) was added until the solution became clear. The resulting mixture was evaporated to dryness to give **74** (10.35 g, 30.41 mmol, 65% yield, Et3N salt) as a beige solid. ^{1}H NMR (DMSO-d_6, 400 MHz): δ 1.17 (s, 12H), 3.06 (q, 6H), 3.92 (s, 3H), 7.70 (d, 2H), 8.60 (s, 1H), 10.34 (s, 1H), 10.34 (brs, 1H).

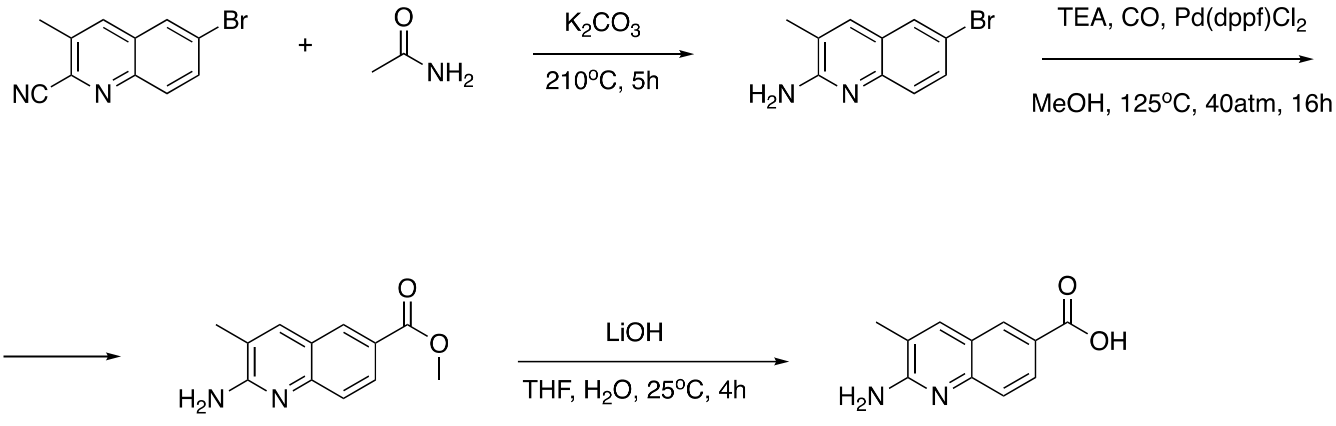

Intermediate 26a

2-amino-3-methylquinoline-6-carboxylic acid, intermediate 26a

6-Bromo-3-methyl-quinolin-2-amine

A mixture of 6-bromo-2-chloro-3-methyl-quinoline (10 g, 38.98 mmol, 1 eq), acetamide (40.00 g, 677.19 mmol. 17.4 eq) and potassium carbonate, anhydrous, 99% (30.00 g, 217.07 mmol, 5.6 eq) was stirred at 210 °C for 5 h. After cooling to room temperature, the reaction mixture was poured into H_2O. The precipitate was collected by filtration, washed with H_2O, and dried at 70 °C overnight to give 6-bromo-3-methyl-quinolin-2-amine (12 g, crude used as is). LCMS (ESI): [M+3H]+ m/z: calcd 239.0; found 239.0; Rt = 0.863 min.

Methyl 2-amino-3-methyl-quinoline-6-carboxylate

Crude 6-bromo-3-methyl-quinolin-2-amine (12 g crude, assume 38.98 mmol, 1 eq), TEA (12.70 mL, 91.10 mmol, 2.4 eq) and [1,1′-Bis(diphenylphosphino)ferrocene]dichloropalladium(II) (2.28 mmol) were dissolved in MeOH (300 mL). The reaction mixture was stirred at 125 °C and 40 atm for 16 h under CO atmosphere. The solvent was evaporated, and the mixture was poured into H_2O (200 mL) and extracted with EtOAc (2 x 200 mL). Combined organic layers were dried over Na_2SO_4 and evaporated to give methyl 2-amino-3-methyl-quinoline-6-carboxylate (5.2 g, 24.05 mmol,

62% yield over 2 steps). ^{1}H NMR (DMSO-d_6, 400 MHz): δ 2.27 (s, 3H), 3.91 (s, 3H), 6.78 (s, 2H), 7.52 (m, 1H), 7.93 – 7.99 (m, 2H), 8.32 (s, 1H). LCMS (ESI): [M+H]+ m/z: calcd 217.1; found 218.2; Rt = 0.812 min.

2-Amino-3-methyl-quinoline-6-carboxylic acid, intermediate 26a
To the stirring solution of methyl 2-amino-3-methyl-quinoline-6-carboxylate (5.2 g, 24.05 mmol, 1 eq) in THF (20 mL) / H_2O (15 mL) Lithium hydroxide monohydrate, 98% (2.32 g, 55.31 mmol, 2.3 eq) was added and the resulting mixture was stirred at 25 °C for 4 h, after which the THF was evaporated under reduced pressure. The remaining aqueous solution was acidified with sodium bisulfate to slightly acidic pH. Product was extracted with EtOAc (2 x 100 mL), dried over Na_2SO_4. EtOAc was evaporated to give 2-amino-3-methyl-quinoline-6-carboxylic acid, **intermediate 26a** (3.7 g, 18.30 mmol, 76% yield). ^{1}H NMR (DMSO-d_6, 400 MHz): δ 2.27 (s, 3H), 6.78 (s, 2H), 7.52 – 7.55 (d, 1H), 7.93 (s, 1H), 7.96 – 7.99 (d, 1H), 8.32 (s, 1H). LCMS (ESI): [M+H]+ m/z: calcd 203.2; found 203.2; Rt = 0.564 min.

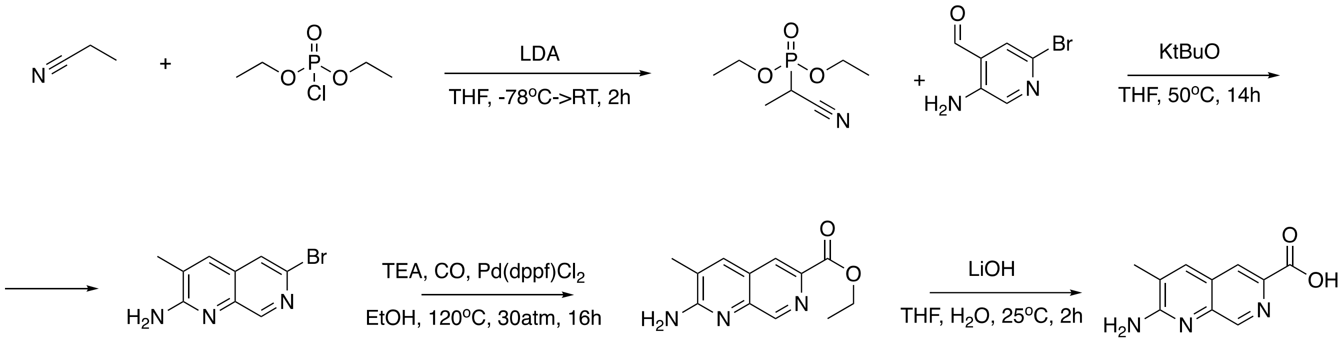

Intermediate 27a

2-amino-3-methyl-1,7-naphthyridine-6-carboxylic acid, intermediate 27a

Diethyl (1-cyanoethyl)phosphonate
To a pre-cooled (−78 °C) lithium diisopropylamide solution (199.70 mL, 417.59 mmol, 28% purity, 2.3 eq) in THF (100 mL) a solution of propanenitrile (12.95 mL, 181.56 mmol, 1 eq) in THF (50 mL) was added dropwise over 5 min. After stirring for a further 10 min at −78 °C, a solution of 1-[chloro(ethoxy)phosphoryl]oxyethane (31.33 g, 181.56 mmol, 1 eq) in THF (50 mL) was added dropwise over 10 min. After a further 45 min of stirring at −78 °C, the mixture was allowed to warm to room temperature. After stirring for 30 additional minutes the reaction was quenched by pouring the reaction mixture onto a stirred mixture of ice (30 g), 2M HCl (150 mL) and MTBE (150 mL). The organic and aqueous phases were partitioned, and the aqueous phase was extracted with CH_2Cl_2 (2 x 50 mL). The combined organic phase was dried over Na_2SO_4, filtered, and concentrated in vacuo to give crude product which was then distilled in vacuo (0.1 Torr / 57-63 °C) to give 2-diethoxyphosphorylpropanenitrile (25 g, 130.78 mmol, 72% yield). ^{1}H NMR ($CDCl_3$, 400 MHz): δ 1.25 – 1.27 (t, 3H), 1.33 – 1.37 (t, 3H), 1.49 – 1.56 (d, 3H), 3.34 – 3.42 (q, 1H), 4.18 – 4.24 (q, 4H).

6-Bromo-3-methyl-1,7-naphthyridin-2-amine
Potassium tert-butoxide (3.91 g, 34.82 mmol, 1.4 eq) was added to the solution of 5-amino-2-bromo-pyridine-4-carbaldehyde (5 g, 24.87 mmol, 1 eq) in THF (120 mL). After 10 minutes, 2-diethoxyphosphorylpropanenitrile (6.18 g, 32.34 mmol, 1.4 eq) was added. The resulting reaction mixture was stirred at 50 °C for 14 h. The volatiles were removed under reduced pressure and the resulting residue was taken up in H_2O (100 mL). A brown precipitate formed and was filtered and dried to give 6-bromo-3-methyl-1,7-naphthyridin-2-amine (4.5 g, 18.90 mmol, 76% yield). ^{1}H NMR (DMSO-d_6, 400 MHz): δ 2.24 (s, 3H), 6.80 (s, 2H), 7.74 (s, 1H), 7.78 (s, 1H), 8.56 (s, 1H). LCMS (ESI): [M+2H]+ m/z: calcd 240.0; found 240.0; Rt = 0.752 min.

Ethyl 2-amino-3-methyl-1,7-naphthyridine-6-carboxylate
6-Bromo-3-methyl-1,7-naphthyridin-2-amine (4.5 g, 18.90 mmol, 1 eq) was dissolved in EtOH (150 mL). TEA (3.16 mL, 22.68 mmol,1.2 eq) and Pd(dppf)Cl2•DCM (15.44 g, 18.90 mmol, 1 eq) were added. The resulting mixture was stirred at 120 °C for 16 h under an atmosphere of CO (30 Bar). The reaction mixture was filtered through a short pad of silica, after which the solvent was evaporated under reduced pressure. The solid residue was washed with EtOAc (2 x 25 mL) and dried in vacuo to give ethyl 2-amino-3-methyl-1,7-naphthyridine-6-carboxylate (1.9 g, 8.22 mmol, 43% yield). ^{1}H NMR (DMSO-d_6, 500 MHz): δ 1.32 (t, 3H), 2.23 (s, 3H), 4.32 (q, 2H), 6.99 (s, 2H), 7.93 (s, 1H), 8.27 (s, 1H), 8.79 (s, 1H). LCMS (ESI): [M+H]+ m/z: calcd 232.2; found 232.1; Rt = 0.792 min.

2-Amino-3-methyl-1,7-naphthyridine-6-carboxylic acid, intermediate 27a
To a stirred solution of ethyl 2-amino-3-methyl-1,7-naphthyridine-6-carboxylate (1.9 g, 8.22 mmol, 1 eq) in THF (40 mL) a solution of lithium hydroxide monohydrate, 98% (517.17 mg, 12.32 mmol, 1.5 eq) in THF (40 mL) was added. The resulting mixture was stirred at 25 °C for 2 h. The reaction mixture was concentrated in vacuo. The residue was acidified with a $NaHSO_4$ solution. The precipitate was filtered, washed with H_2O, then with ACN and MTBE. The resultant solid was dried in vacuo to give 2-amino-3-methyl-1,7-naphthyridine-6-carboxylic acid, **intermediate 27a** (1.4 g, 6.89 mmol, 84% yield). ^{1}H NMR (DMSO-d_6, 400 MHz): δ 2.25 (s, 3H), 6.98 (s, 2H), 7.93 (s, 1H), 8.41 (s, 1H), 8.79 (s, 1H). LCMS (ESI): [M+H]+ m/z: calcd 204.2; found 204.0; Rt = 0.556 min.

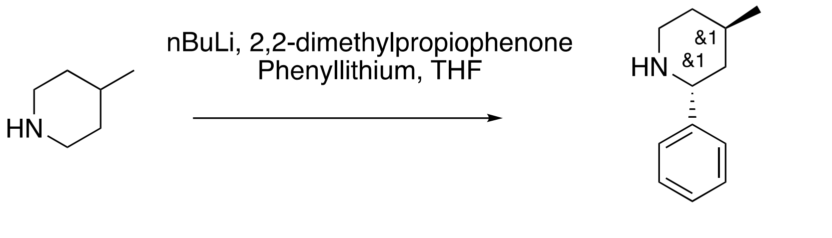

Intermediate 18a

rac-(2R,4R)-4-methyl-2-phenylpiperidine, intermediate 18a
2.5M nBuLi in hexanes (4.36 mL, 10.39 mmol, 22% purity) was added to a cooled (-78 °C) solution of 4-methylpiperidine (1.2 mL, 10.08 mmol) in THF (30 mL). The resultant solution was stirred for 10 minutes. To this was added 2,2-dimethylpropiophenone (1.96 g, 12.10 mmol) in THF (15 mL), and the resultant mixture was stirred at -78 °C for 1 h, then 1.9M phenyllithium in Et_2O (7.6 mL, 15.12 mmol) was slowly added and the resulting mixture was stirred for 5 h, then quenched via the addition of 10 mL MeOH at -78 °C. The solvent was evaporated in vacuo, 10 mL brine was added and this was extracted with MTBE (4 x 25mL). The combined organic extract was dried over sodium sulfate and evaporated to give crude product which was purified by column chromatography ($CHCl_3$-MeOH gradient) to give rac-(2R,4R)-4-methyl-2-phenylpiperidine, **intermediate 18a**, 14% yield.

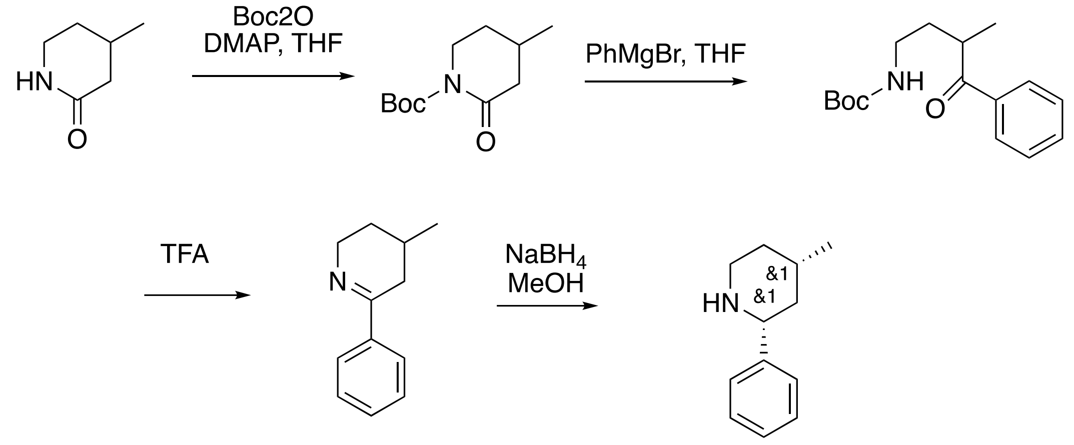

Intermediate 19a

tert-butyl 4-methyl-2-oxopiperidine-1-carboxylate
Di-tert-butyl dicarbonate (7g, 31.8 mmol) was added to a solution of 4-methylpiperidin-2-one (3g, 26.5 mmol) and DMAP (323.9 mg, 2.65 mmol) in THF (100 mL). The solution was stirred at 40 °C for 15 h at which point H_2O (1000 mL) was added and the mixture was stirred for 20 min at which point it was partitioned between 5% aq $NaHSO_4$ and concentrated under reduced pressure affording tert-butyl 4-methyl-2-oxopiperidine-1-carboxylate in 88% yield. LCMS (ESI): [M+H]+ m/z: calcd 213.14; found 214.2; Rt = 1.034 min, column: Agilent Poroshell 120 SB-C18 4.6 X 30mm 2.7 µm, 60 °C, Mobile phase: A-H_2O (0.1% formic acid), B-ACN (0.1% formic acid), flow rate: 3 mL/min, Gradient: 0.01 min - 1% B, 1.5 min - 100% B, 1.73 min - 100% B, MS Ionization mode: ESI, MS Scan range: 83-600 m/z, UV detection: 215 nm, 254 nm, 280 nm.

tert-butyl (3-methyl-4-oxo-4-phenylbutyl)carbamate
tert-butyl 4-methyl-2-oxopiperidine-1-carboxylate (5g, 23.44 mmol) was stirred at -78 °C in THF (100 mL). Phenylmagnesium bromide (42.5 mL, 35.17 mmol, 15% purity) was added over 0.5 h. The solution was then warmed to room temperature and saturated ammonium chloride was added. The aqueous layer was extracted 3 x 50 mL with DCM and the organic layers combined, dried over sodium sulfate, filtered, and concentrated. 7 grams crude of tert-butyl (3-methyl-4-oxo-4-phenylbutyl)carbamate was used without further purification.

4-methyl-6-phenyl-2,3,4,5-tetrahydropyridine
tert-butyl (3-methyl-4-oxo-4-phenylbutyl)carbamate (7g, 24 mmol) was stirred in TFA (23.5 mL) at room temperature for 2 h. The solvent was removed, 50 mL sodium bicarbonate was added and this was extracted with DCM 3 x 50mL. The organic layer was washed with brine (50 mL), dried over sodium sulfate, and evaported to give 4-methyl-6-phenyl-2,3,4,5-tetrahydropyridine (4g, 23.1mmol, 96% yield). LCMS (ESI): [M+H]+ m/z: calcd 173.12; found 174.2; Rt = 0.599 min, column: Agilent Poroshell 120 SB-C18 4.6 X 30 mm 2.7 µm, 60 °C, Mobile phase: A-H_2O (0.1% formic acid), B-ACN (0.1% formic acid), flow rate: 3 mL/min, Gradient: 0.01 min - 1% B, 1.5 min - 100% B, 1.73 min - 100% B, MS Ionization mode: ESI, MS Scan range: 83-600 m/z, UV detection: 215 nm, 254 nm, 280 nm.

rac-(2R,4S)-4-methyl-2-phenylpiperidine, intermediate 19a
4-methyl-6-phenyl-2,3,4,5-tetrahydropyridine (4g, 23.1mmol) was stirred at 0 °C in MeOH (80 mL) and sodium borohydride (873 mg, 23.1 mmol) was added portionwise. The mixture was stirred at 0 °C for 2 h. The solvent was then removed and the residue was partitioned between H_2O (20 mL) and DCM (40 mL). The organic layer was separated, dried over potassium carbonate, and concentrated to give rac-(2R,4S)-4-methyl-2-phenylpiperidine, **intermediate 19a** (3.5 g, 19.97 mmol, 86% yield). LCMS (ESI): [M+H]+ m/z: calcd 175.14;

found 176.2; Rt = 0.726 min, column: Agilent Poroshell 120 SB-C18 4.6 X 30 mm 2.7 um, 60 °C, Mobile phase: A-H_2O (0.1% formic acid), B-ACN (0.1% formic acid), flow rate: 3mL/min, Gradient: 0.01 min - 1% B, 1.5 min - 100% B, 1.73 min - 100% B, MS Ionization mode: ESI, MS Scan range: 83-600 m/z, UV detection: 215 nm, 254 nm, 280 nm.

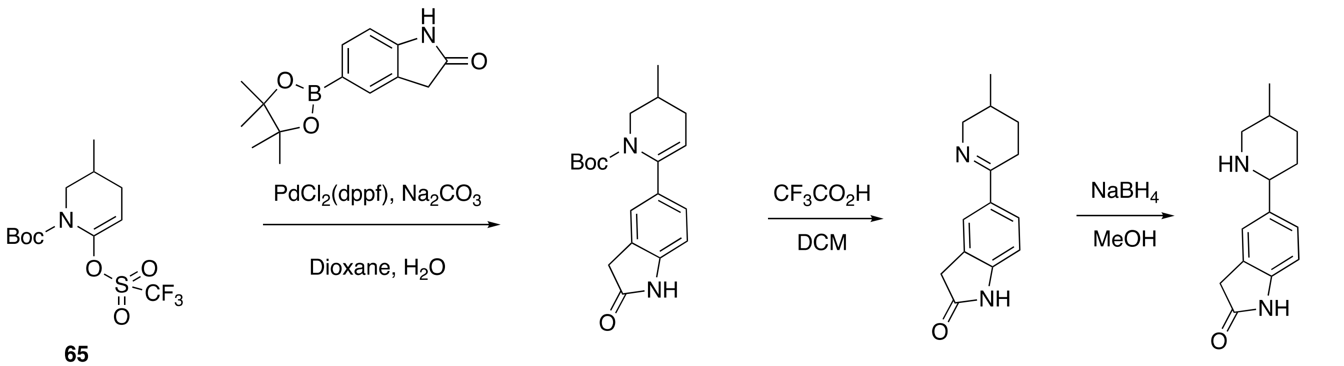

65

Intermediate 36a

rac-5-((2R,5S)-5-methylpiperidin-2-yl)indolin-2-one, intermediate 36a

tert-butyl 3-methyl-6-(2-oxoindolin-5-yl)-3,4-dihydropyridine-1(2H)-carboxylate
tert-butyl 3-methyl-6-(trifluoromethylsulfonyloxy)-3,4-dihydro-2H-pyridine-1-carboxylate (1 eq), 5-(4,4,5,5-tetramethyl-1,3,2-dioxaborolan-2-yl)indolin-2-one (1.1 eq) and Sodium carbonate (2eq) was suspended in 1,4-dioxane: H_2O (9:1, 0.3M) and was purged with argon. Pd(dppf)Cl_2 (0.05 eq) was added under argon. The reaction mixture was stirred under argon at 75 °C for 18h after which it was cooled and filtered. The filtrate was evaporated in vacuo and the residue was diluted with H_2O and MTBE (1:1.5) and the layers were separated. The aqueous layer was further extracted with MTBE. The combined organic layer was dried over Na_2SO_4, filtered and concentrated under reduced pressureto obtain tert-butyl 3-methyl-6-(2-oxoindolin-5-yl)-3,4-dihydro-2H-pyridine-1-carboxylate (83% yield) as brown solid. LCMS (ESI): [M+H]+ m/z: calcd 328.2; found 329.2; Rt = 1.336 min.

5-(5-methyl-3,4,5,6-tetrahydropyridin-2-yl)indolin-2-one
To a stirred solution of tert-butyl 3-methyl-6-(2-oxoindolin-5-yl)-3,4-dihydro-2H-pyridine-1-carboxylate (1 eq) in DCM (0.6M) was added TFA (1.2M) dropwise at 0 °C. The resulting reaction mixture was stirred for 1h at 0 °C. After 1h the reaction mixture was carefully poured into K_2CO_3 solution and extracted with DCM two times. The combined organic phase was dried over Na_2SO_4, filtered and concentrated under reduced pressure to obtain 5-(3-methyl-2,3,4,5-tetrahydropyridin-6-yl)indolin-2-one (100% yield). The crude product was used in the next step reaction without any further purification. LCMS (ESI): [M+H]+ m/z: calcd 228.2; found 229.2; Rt = 0.727 min.

5-(5-methylpiperidin-2-yl)indolin-2-one, intermediate 36a
Sodium Borohydride (3 eq) was added portion wise at 0 °C, to a stirred solution of 5-(3-methyl-2,3,4,5-tetrahydropyridin-6-yl)indolin-2-one (5.54 g, 24.27 mmol, 1 eq) in MeOH (0.2M). The reaction mixture was stirred at 20 °C for 18h. After 18h, the reaction mixture was concentrated under reduced pressure. The obtained residue was diluted with H_2O. The resulting mixture was extracted with DCM 3X. The combined organic layer was dried over Na_2SO_4, filtered, and concentrated under reduced pressure. The obtained residue was dissolved in DCM and washed with aqueous $NaHSO_4$ solution. The aqueous layer was washed

with DCM 3X and then basified with K_2CO_3. The resulting mixture was once again extracted with DCM 3X. The combined organic phase was dried over Na_2SO4, filtered and concentrated under reduced pressure to obtain 5-(5-methyl-2-piperidyl)indolin-2-one, **intermediate 36a** (45% yield). LCMS (ESI): [M+H]+ m/z: calcd 230.1; found 231.2 ; Rt = 0.664 min

Intermediates 30a, 32a, 34a, 38a, 42a were prepared from the corresponding aryl boronates using the same synthetic method as for intermediate 36a.

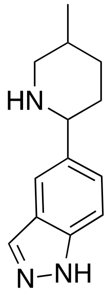

rac-5-((2R,5S)-5-methylpiperidin-2-yl)indolin-2-one, intermediate 30a
Was obtained from 5-(4,4,5,5-tetramethyl-1,3,2-dioxaborolan-2-yl)-1H-indazole following the procedure for **intermediate 36a** in 27% yield over 3 steps. ^{1}H NMR (400 MHz, DMSO-d_6): δ 0.80 (d, 3H), 1.07 (m, 2H), 1.35 (m, 1H), 1.49 (m, 1H), 1.73 (m, 2H), 2.25 (m, 1H), 2.96 (m, 1H), 3.50 (m, 1H), 7.31 (d, 1H), 7.39 (d, 1H), 7.64 (s, 1H), 7.95 (s, 1H), 12.89 (m, 1H). LCMS (ESI): [M+H]+ m/z: calcd 215.1; found 216.2; Rt = 0.645 min.

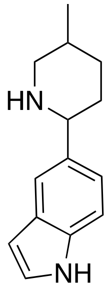

rac-5-((2R,5S)-5-methylpiperidin-2-yl)-1H-indole, intermediate 32a
Was obtained from 5-(4,4,5,5-tetramethyl-1,3,2-dioxaborolan-2-yl)-1H-indole following the procedure for **intermediate 36a** in 14% yield over 3 steps. ^{1}H NMR (400 MHz, DMSO-d_6) δ 0.79 (d, 3H), 1.06 (m, 1H), 1.34 (m, 2H), 1.74 (m, 2H), 2.25 (m, 1H), 2.98 (m, 1H), 3.29 (m, 1H), 5.28 (m, 1H), 6.30 (m, 1H), 7.23 (m, 4H), 10.90 (m, 1H).
LCMS (ESI): [M]+ m/z: calcd 214.2; found 215.2; Rt = 0.950 min.

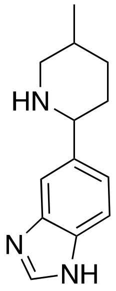

rac-5-((2R,5S)-5-methylpiperidin-2-yl)-1H-benzo[d]imidazole, intermediate 34a

Was obtained from 5-(4,4,5,5-tetramethyl-1,3,2-dioxaborolan-2-yl)-1H-benzo[d]imidazole following the procedure for **intermediate 36a** in 12% yield over 3 steps ^{1}H NMR (500 MHz, DMSO-d_6) δ 0.84 (m, 3H), 1.11 (m, 1H), 1.53 (m, 2H), 1.79 (m, 2H), 2.31 (m, 1H), 3.02 (m, 1H), 3.56 (m, 1H), 7.17 (m, 1H), 7.49 (m, 3H), 8.13 (m, 1H), 12.36 (m, 1H).
LCMS (ESI): [M]+ m/z: calcd 215.2; found 216.2; Rt = 0.627 min.

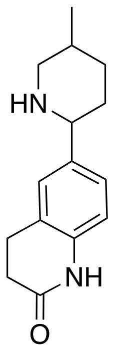

rac-6-((2R,5S)-5-methylpiperidin-2-yl)-3,4-dihydroquinolin-2(1H)-one, intermediate 38a
Was obtained from 6-(4,4,5,5-tetramethyl-1,3,2-dioxaborolan-2-yl)-3,4-dihydroquinolin-2(1H)-one following the procedure for **intermediate 36a** in 13% yield over 3 steps. LCMS (ESI): [M+H]+ m/z: calcd 230.2; found 231.2; Rt = 0.730 min.

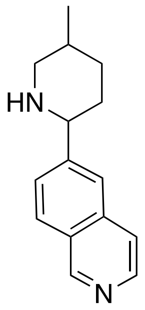

rac-6-((2R,5S)-5-methylpiperidin-2-yl)isoquinoline, intermediate 42a
Was obtained from 6-(4,4,5,5-tetramethyl-1,3,2-dioxaborolan-2-yl)isoquinoline following the procedure for **intermediate 36a** in 29% yield over 3 steps. ^{1}H NMR (400 MHz, $CDCl_3$) δ 0.89 (d, 3H), 1.18 (m, 2H), 1.71 (m, 1H), 1.88 (m, 2H), 2.12 (m, 1H), 2.45 (m, 1H), 3.18 (m, 1H), 3.73 (m, 1H), 7.60 (m, 2H), 7.81 (s, 1H), 7.88 (d, 1H), 8.46 (d, 1H), 8.18 (s, 1H). LCMS (ESI): [M+H]+ m/z: calcd 226.2; found 227.2; Rt = 0.607 min.

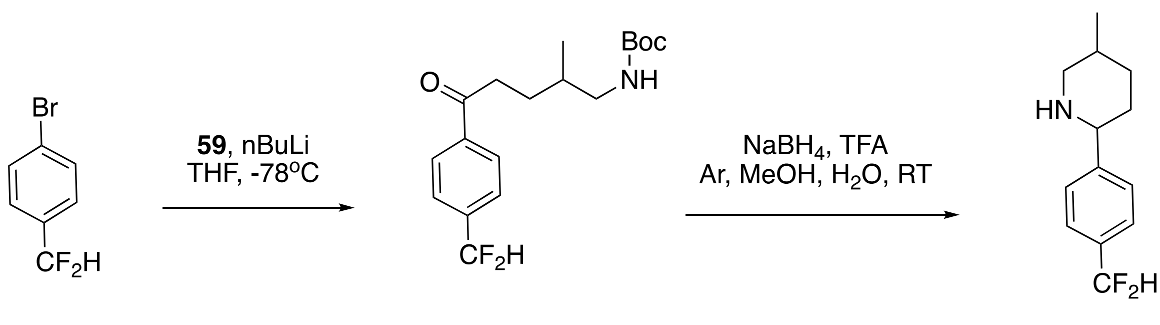

Intermediate 40a

2-(4-(difluoromethyl)phenyl)-5-methylpiperidine, intermediate 40a

tert-butyl (5-(4-(difluoromethyl)phenyl)-2-methyl-5-oxopentyl)carbamate
To a solution of 1-bromo-4-(difluoromethyl)benzene (4.6 g, 22.22 mmol, 1 eq) in THF (25 mL), n-Butyllithium (6.19 g, 22.22 mmol, 8.93 mL, 23% purity, 1 eq) was added dropwise over a period of 30 min at -78° C. The reaction was stirred for 1 h at -78° C and **59** (4.74 g, 22.22 mmol, 1 eq) was added and the reaction was stirred at -78° C. for 1 h, after which the reaction mixture was brought to 0° C and treated with saturated ammonium chloride solution and extracted with ethyl acetate. The organic layer was dried over anhydrous sodium sulfate, filtered and concentrated under reduced pressure to provide tert-butyl N-[5-[4-(difluoromethyl)phenyl]-2-methyl-5-oxo-pentyl]carbamate (7.2 g, 21.09 mmol, 95% yield) which was used in the next step without further purification. ^{1}H NMR (500 MHz, DMSO-d_6) δ 0.94 (m, 3H), 1.43 (m, 9H), 1.69 (m, 2H), 2.35 (m, 2H), 3.04 (m, 2H), 4.71 (m, 2H), 7.50 (m, 2H), 7.59 (m, 2H), 8.04 (m, 1H). LCMS (ESI): [M-Boc]+ m/z: calcd 241.2; found 242.2; Rt = 1.564 min.

2-(4-(difluoromethyl)phenyl)-5-methylpiperidine, intermediate 40a
tert-butyl N-[5-[4-(difluoromethyl)phenyl]-2-methyl-5-oxo-pentyl]carbamate (8.8 g, 25.78 mmol, 1 eq) was stirred in trifluoroacetic acid (9.93 mL, 128.88 mmol, 5 eq) for 1 h. 50% w/v NaOH solution was added to the mixture until the pH was 13-14. The product was extracted 4 x 20mL with DCM and the organic layers combined, dried with $MgSO_4$ and evaporated. The product was dissolved in mixture MeOH (50 mL) / H_2O (50 mL) followed by sodium borohydride (975.20 mg, 25.78 mmol, 1 eq). The mixture was stirred under Ar overnight. The mixture was acidified with 1-2M HCl until the pH was 1-3 and left for 30 minutes. NaOH solution was then added until the pH was 13-14 and the product was extracted with DCM (4 x 100 mL), the organic layers were combined, dried with Na_2SO_4, filtered and evaporated. The residue was flash column to give 2-[4-(difluoromethyl)phenyl]-5-methyl-piperidine, **intermediate 40a** (0.63 g, 2.80 mmol, 11% yield) as an light-yellow oil. ^{1}H NMR (500 MHz, $CDCl_3$) δ 0.92 (d, 3H), 1.17 (m, 1H), 1.84 (m, 5H), 2.43 (m, 1H), 3.16 (m, 1H), 3.62 (m, 1H), 7.47 (s, 4H). LCMS (ESI): [M+H]+ m/z: calcd 225.2; found 226.2; Rt = 0.890 min.

General Procedure C:
HATU (1 eq) was added portion wise at room temperature to a suspension of oxamic acid (1 eq), amine (1 eq) and triethylamine (6 eq) in DMF (0.1 M). The solution was stirred at 20 °C for 18 h and the solvent was evaporated in vacuo to give crude material which was purified by HPLC to give racemic compound. The enantiomers were separated by chiral HPLC or SFC.

Compounds 18, 19, 24, 26, 27, 29, 31, 33, 35, 37, 39, 41, 43, 45, 46, 47.

The following compounds were prepared with General Procedure C.

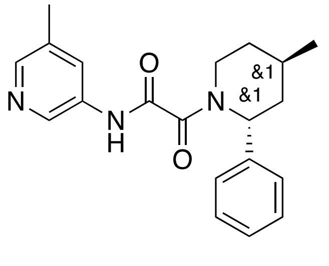

rac-2-((2R,4R)-4-methyl-2-phenylpiperidin-1-yl)-N-(5-methylpyridin-3-yl)-2-oxoacetamide (18).
Amine **intermediate 18a** and acid **56**. White solid, 87% yield. ^{1}H NMR (600 MHz, DMSO-*d*$_6$) δ 0.91 (d, 3H), 1.16 (m, 1H), 1.54 (m, 3H), 2.27 (d, 3H), 2.80 (m, 2H), 3.99 (dd, 1H), 5.49 (dd, 1H), 7.35 (m, 5H), 7.93 (d, 1H), 8.17 (d, 1H), 8.58 (dd, 1H), 11.05 (d, 1H). LCMS (ESI): [M+H]+ m/z: calcd 337.2; found 338.2; Rt = 2.096 min.

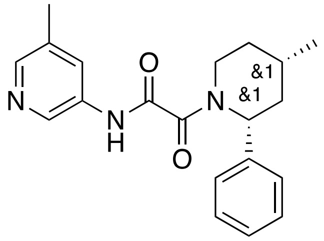

rac-2-((2R,4S)-4-methyl-2-phenylpiperidin-1-yl)-N-(5-methylpyridin-3-yl)-2-oxoacetamide (19).
Amine **intermediate 19a** and acid **56**. White solid, 70% yield. ^{1}H NMR (600 MHz, DMSO-*d*$_6$) δ 0.86 (d, 3H), 1.27 (m, 1H), 1.78 (m, 3H), 2.05 (m, 2H), 2.29 (s, 3H), 5.17 (m, 1H), 7.31 (m, 5H), 7.81 (d, 1H), 8.13 (d, 1H), 8.49 (d, 1H), 10.82 (d, 1H). LCMS (ESI): [M+H]+ m/z: calcd 337.2; found 338.2; Rt = 2.932 min.

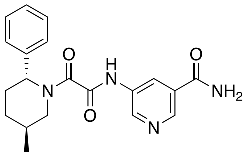

5-(2-((2R,5S)-5-methyl-2-phenylpiperidin-1-yl)-2-oxoacetamido)nicotinamide (24).
Amine **61** and acid **56**. White solid, 35% yield. ^{1}H NMR (600 MHz, DMSO-*d*$_6$) δ 1.03 (m, 3H), 1.34 (m, 1H), 1.66 (m, 1H), 1.85 (m, 1H), 2.05 (m, 1H), 2.21 (m, 1H), 3.26 (m, 1H), 3.75 (m, 1H), 5.61 (m, 1H), 7.27 (m, 1H), 7.37 (m, 4H), 7.59 (m, 1H), 8.15 (m, 1H), 8.48 (m, 1H), 8.77 (m, 1H), 8.89 (m, 1H), 11.23 (m, 1H). LCMS (ESI): $[M+H]^+$ m/z: calcd 366.2; found 367.2; Rt = 4.363 min. RT. HPLC (IA (250 x 25 mm, 5 µm), Hexane-IPA-MeOH, 60-20-20, 0.6 mL/min) = 38.51 min.

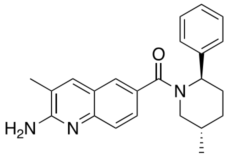

(2-amino-3-methylquinolin-6-yl)((2R,5S)-5-methyl-2-phenylpiperidin-1-yl)methanone (26).
Acid **intermediate 26a** and amine **61**. Light yellow solid, 8% yield. ^{1}H NMR (600 MHz, DMSO-*d*$_6$) δ 0.96 (d, 3H), 1.28 – 1.34 (m, 1H), 1.57 – 1.66 (m, 1H), 1.75 – 1.84 (m, 1H), 2.09 – 2.18 (m, 2H), 2.19 (s, 3H), 2.97 – 3.08 (m, 1H), 3.55 – 3.86 (m, 1H), 5.34 – 5.60 (m, 1H), 6.43 (s, 2H), 7.26 (t, 1H), 7.33 (d, 2H), 7.39 (t, 2H), 7.43 (dd, 1H), 7.45 (d, 1H), 7.66 (d, 1H), 7.77 (s, 1H). LCMS (ESI): [M+H]+ m/z: calcd 360.25; found 360.2; Rt = 1.119 min, column: Agilent Poroshell 120 SB-C18 4.6 x 30mm, 2.7 µm, 60°C, Mobile phase: A – H_2O (0.1% formic acid), B – ACN (0.1% formic acid), flow rate: 3 mL/min, gradient: 0.01 min –

1% B, 1.5 min – 100% B, 1.73 min – 100% B, MS Ionization mode: Electrospray ionization (ESI), MS Scan range: 83 – 600 m/z, UV detection: 215 nm, 254 nm, 280 nm.

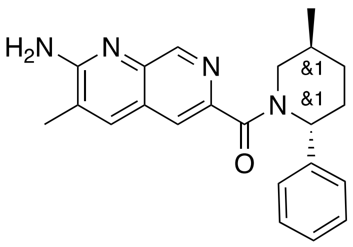

(2-amino-3-methyl-1,7-naphthyridin-6-yl)((2R,5S)-5-methyl-2-phenylpiperidin-1-yl)methanone (27).
Acid **27a intermediate** and amine **61**. White solid, 15% yield. Column: CHIRALPAK AD-H (250 x 20 mm, 5 µm)--ADH0EJ-AT001—VI. Mobile Phase: Hexane:IPA:MeOH, 50:25:25; Flow Rate: 12 mL/min, RT = 17.03 min. ^{1}H NMR (600 MHz, DMSO-d_6) δ 0.93 (m, 3H), 1.33 (m, 2H), 1.68 (m, 2H), 2.17 (m, 5H), 2.98 (m, 2H), 5.75 (m, 2H), 6.80 (s, 1H), 7.24 (m, 1H), 7.38 (m, 3H), 7.79 (s, 1H), 7.86 (s, 1H), 8.76 (s, 1H). LCMS (ESI): [M+H]+ m/z: calcd 361.45; found 361.2; Rt = 2.757 min.

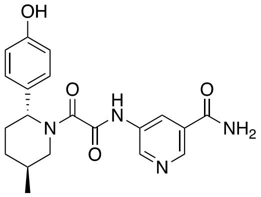

5-(2-((2R,5S)-2-(4-hydroxyphenyl)-5-methylpiperidin-1-yl)-2-oxoacetamido)nicotinamide (29).
Acid **56** and amine **68**. White solid, 4% yield. RT = 29.61 min. ^{1}H NMR (600 MHz, DMSO-d_6) δ 0.98 – 1.04 (m, 3H), 1.27 – 1.38 (m, 1H), 1.65 – 1.77 (m, 1H), 1.80 – 1.91 (m, 1H), 1.96 – 2.11 (m, 1H), 2.12 – 2.20 (m, 1H), 2.74 – 3.21 (m, 1H), 3.37 – 4.02 (m, 1H), 5.01 – 5.56 (m, 1H), 6.70 – 6.78 (m, 2H), 7.04 – 7.15 (m, 2H), 7.53 – 7.68 (m, 1H), 8.10 – 8.22 (m, 1H), 8.41 – 8.51 (m, 1H), 8.69 – 8.81 (m, 1H), 8.82 – 8.93 (m, 1H), 9.36 (s, 1H), 11.11 – 11.28 (m, 1H). LCMS (ESI): $[M]^+$ m/z: calcd 382.5; found 383.2; Rt = 2.56 min.

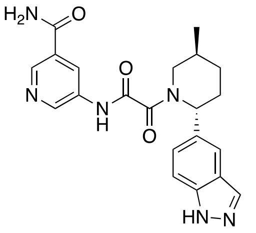

5-(2-((2R,5S)-2-(1H-indazol-5-yl)-5-methylpiperidin-1-yl)-2-oxoacetamido)nicotinamide (31).
Acid **56** and amine **intermediate 30a**. White solid, 16% yield (coupling and separation). ^{1}H NMR (600 MHz, DMSO-d_6) δ 1.00 – 1.06 (m, 3H), 1.30 – 1.43 (m, 1H), 1.73 – 1.83 (m, 1H), 1.83 – 1.93 (m, 1H), 2.07 – 2.23 (m, 1H), 2.25 – 2.35 (m, 1H), 2.78 – 3.26 (m, 1H), 3.44 – 4.06 (m, 1H), 5.20 – 5.74 (m, 1H), 7.27 – 7.39 (m, 1H), 7.49 – 7.64 (m, 2H), 7.69 – 7.75 (m, 1H), 8.00 – 8.07 (m, 1H), 8.08 – 8.21 (m, 1H), 8.42 – 8.55 (m, 1H), 8.71 – 8.80 (m, 1H), 8.80 – 8.96 (m, 1H), 11.16 – 11.33 (m, 1H), 12.96 – 13.07 (m, 1H). LCMS (ESI): [M+H]+ m/z: calcd 406.2; found 407.2; Rt = 2.467 min.

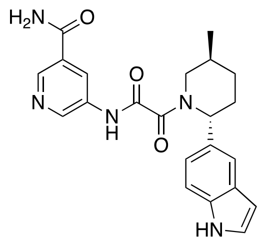

5-(2-((2R,5S)-2-(1H-indol-5-yl)-5-methylpiperidin-1-yl)-2-oxoacetamido)nicotinamide (33). Acid **56** and amine **intermediate 32a**. White solid, 6% yield (coupling and separation). Retention time: 38.68 min. ^{1}H NMR (500 MHz, DMSO-d_6) δ 1.00 – 1.14 (m, 3H), 1.32 – 1.47 (m, 1H), 1.69 – 2.07 (m, 2H), 2.07 – 2.30 (m, 1H), 2.30 – 2.38 (m, 1H), 2.82 – 3.25 (m, 1H), 3.43 – 4.07 (m, 1H), 5.21 – 5.80 (m, 1H), 6.36 – 6.47 (m, 1H), 7.01 – 7.16 (m, 1H), 7.26 – 7.46 (m, 2H), 7.47 – 7.68 (m, 2H), 8.08 – 8.27 (m, 1H), 8.43 – 8.60 (m, 1H), 8.70 – 8.84 (m, 1H), 8.84 – 9.02 (m, 1H), 10.99 – 11.16 (m, 1H), 11.16 – 11.34 (m, 1H). LCMS (ESI): [M]+ m/z: calcd 405.2; found 406.0; Rt = 2.811 min.

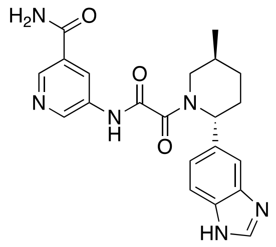

5-(2-((2R,5S)-2-(1H-benzo[d]imidazol-5-yl)-5-methylpiperidin-1-yl)-2-oxoacetamido)nicotinamide (35).
Acid **56** and amine **intermediate 34a**. White solid, 8% yield (coupling and separation). Retention time: 16.21 min. ^{1}H NMR (600 MHz, DMSO-d_6) δ 1.04 (m, 3H), 1.36 (m, 1H), 1.74 (m, 1H), 1.87 (m, 1H), 2.13 (m, 1H), 2.29 (m, 1H), 2.94 (m, 1H), 3.75 (m, 1H), 5.49 (m, 1H), 7.17 (m, 1H), 7.49 (m, 1H), 7.63 (m, 2H), 8.19 (m, 2H), 8.47 (m, 1H), 8.75 (m, 1H), 8.87 (m, 1H), 11.26 (m, 1H), 12.38 (m, 1H). LCMS (ESI): [M]+ m/z: calcd 406.2; found 407.2; Rt = 1.688 min.

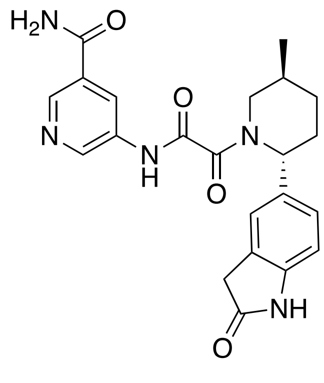

5-(2-((2R,5S)-5-methyl-2-(2-oxoindolin-5-yl)piperidin-1-yl)-2-oxoacetamido)nicotinamide (37).
Acid **56** and amine **intermediate 36a**, pink solid, 4% yield. ^{1}H NMR(DMSO-d_6, 500 MHz): δ 1.03 (m, 3H), 1.33 (m, 1H), 1.72 (m, 1H), 1.89 (m, 1H), 2.02 (m, 1H), 2.18 (m, 1H), 2.83

(m, 1H), 3.64 (m, 3H), 5.33 (m, 1H), 6.81 (m, 1H), 7.16 (m, 2H), 7.60 (m, 1H), 8.15 (m, 1H), 8.47 (m, 1H), 8.75 (m, 1H), 8.90 (m, 1H), 10.35 (m, 1H), 11.26 (m, 1H). LCMS (ESI): [M+H]+ m/z: calcd 421.2; found 422.2 ; Rt = 2.467 min. Chiral HPLC: Rt = 17.33 min (column: IC; Eluent: CO2 - MeOH, 60 – 40; flow rate: 3.0 mL/min).

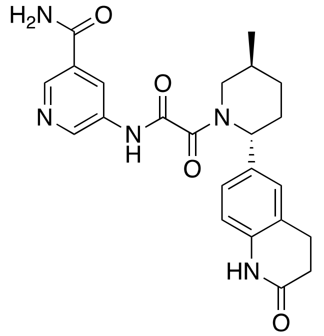

5-(2-((2R,5S)-5-methyl-2-(2-oxo-1,2,3,4-tetrahydroquinolin-6-yl)piperidin-1-yl)-2-oxoacetamido)nicotinamide (39).
Acid **56** and amine **intermediate 38a**. White solid, 7% yield (coupling and separation). RT (IC, MeOH-IPA, 50-50, 0.6 mL/min) = 26.004 min. ^{1}H NMR (600 MHz, DMSO-d_6) δ 0.98 – 1.04 (m, 3H), 1.27 – 1.37 (m, 1H), 1.59 – 1.77 (m, 1H), 1.80 – 1.94 (m, 1H), 1.95 – 2.11 (m, 1H), 2.12 – 2.23 (m, 1H), 2.37 – 2.44 (m, 2H), 2.83 – 3.25 (m, 3H), 3.43 – 4.00 (m, 1H), 5.01 – 5.57 (m, 1H), 6.78 – 6.94 (m, 1H), 7.04 – 7.17 (m, 2H), 7.53 – 7.63 (m, 1H), 8.06 – 8.20 (m, 1H), 8.41 – 8.53 (m, 1H), 8.69 – 8.79 (m, 1H), 8.79 – 8.93 (m, 1H), 10.00 – 10.06 (m, 1H), 11.09 – 11.40 (m, 1H). LCMS (ESI): [M+H]+ m/z: calcd 435.2; found 436.2 ; Rt = 2.215 min.

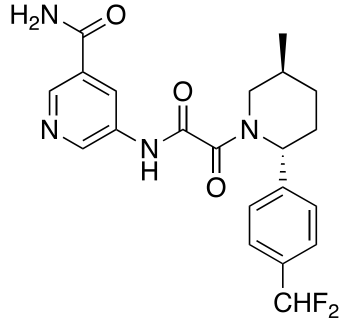

5-(2-((2R,5S)-2-(4-(difluoromethyl)phenyl)-5-methylpiperidin-1-yl)-2-oxoacetamido)nicotinamide (41).
Acid **56** and amine **intermediate 40a**. White solid, 10% yield (coupling and separation). ^{1}H NMR (600 MHz, DMSO-d_6) δ 1.00 – 1.04 (m, 3H), 1.28 – 1.38 (m, 1H), 1.60 – 1.68 (m, 1H), 1.82 – 1.95 (m, 1H), 2.05 – 2.18 (m, 1H), 2.19 – 2.30 (m, 1H), 2.75 – 3.24 (m, 1H), 3.38 – 4.11 (m, 1H), 5.19 – 5.67 (m, 1H), 6.90 – 7.12 (m, 1H), 7.43 – 7.51 (m, 2H), 7.55 – 7.65 (m, 3H), 8.09 – 8.20 (m, 1H), 8.41 – 8.52 (m, 1H), 8.71 – 8.80 (m, 1H), 8.81 – 8.93 (m, 1H), 11.13 – 11.39 (m, 1H). LCMS (ESI): [M+H]+ m/z: calcd 416.2; found 417.2; Rt = 3.135 min.

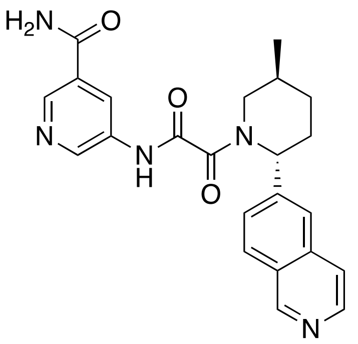

5-(2-((2R,5S)-2-(isoquinolin-6-yl)-5-methylpiperidin-1-yl)-2-oxoacetamido)nicotinamide (43). Acid **56** and amine **intermediate 38a**. White solid, 9% yield (coupling and separation). RT (AD-H, Hex-IPA-MeOH, 50-25-25, 0.6 mL/min) = 23.254 min. ^{1}H NMR (DMSO-d_6, 500 MHz): δ 1.06 (t, 3H), 1.39 (m, 1H), 1.73 (m, 1H), 1.89 (m, 1H), 2.18 (m, 1H), 2.39 (m, 2H), 3.83 (dd, 1H), 5.58 (m, 1H), 7.66 (m, 2H), 7.83 (m, 1H), 7.92 (m, 1H), 8.15 (m, 2H), 8.49 (m, 2H), 8.75 (m, 1H), 8.87 (m, 1H), 9.28 (m, 1H), 11.29 (m, 1H). LCMS (ESI): [M+H]+ m/z: calcd 417.2; found 418.2; Rt = 1.425 min.

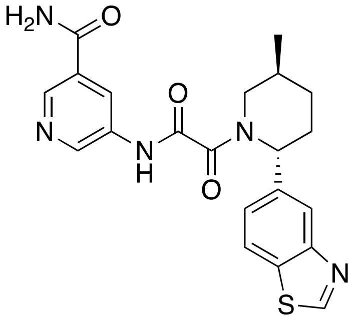

5-(2-((2R,5S)-2-(benzo[d]thiazol-5-yl)-5-methylpiperidin-1-yl)-2-oxoacetamido)nicotinamide (44).
Acid **56** and amine **72**. White solid, 24% yield (coupling and separation). ^{1}H NMR (600 MHz, DMSO-d_6) δ 1.00 – 1.09 (m, 3H), 1.32 – 1.45 (m, 1H), 1.68 – 1.76 (m, 1H), 1.83 – 1.98 (m, 1H), 2.05 – 2.32 (m, 1H), 2.32 – 2.37 (m, 1H), 2.80 – 3.27 (m, 1H), 3.48 – 4.08 (m, 1H), 5.30 – 5.76 (m, 1H), 7.42 – 7.53 (m, 1H), 7.53 – 7.66 (m, 1H), 8.00 – 8.05 (m, 1H), 8.08 – 8.23 (m, 2H), 8.39 – 8.52 (m, 1H), 8.69 – 8.79 (m, 1H), 8.80 – 8.93 (m, 1H), 9.36 – 9.41 (m, 1H), 11.15 – 11.40 (m, 1H). LCMS (ESI): [M]+ m/z: calcd 423.2; found 424.2; Rt = 2.315 min. [α]21D = +169.7° (c = 0.1 g/100 mL EtOH

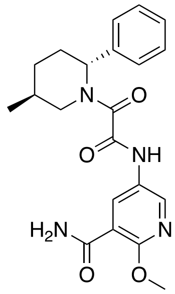

2-methoxy-5-(2-((2R,5S)-5-methyl-2-phenylpiperidin-1-yl)-2-oxoacetamido)nicotinamide (45).

Acid **74** and amine **61**. White solid, 38% yield. RT = 22.71 min. ^{1}H NMR (500 MHz, DMSO-d_6) δ 1.04 (m, 3H), 1.35 (m, 1H), 1.67 (m, 1H), 1.89 (m, 1H), 2.06 (m, 1H), 2.23 (m, 1H), 3.47 (m, 1H), 3.96 (m, 3H), 4.22 (m, 1H), 5.40 (m, 1H), 7.32 (m, 2H), 7.40 (m, 3H), 7.75 (m, 2H), 8.47 (m, 1H), 8.55 (m, 1H), 11.04 (m, 1H).
LCMS (ESI): [M+1] m/z: calcd 396.4; found 397.2; Rt = 3.361 min.

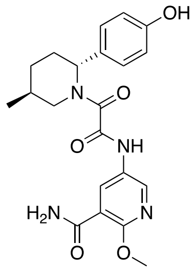

5-(2-((2R,5S)-2-(4-hydroxyphenyl)-5-methylpiperidin-1-yl)-2-oxoacetamido)-2-methoxynicotinamide (46).
Acid **74** and amine **68**. White solid, 16% yield. Retention time: 44.81 min. ^{1}H NMR (500 MHz, DMSO-d_6) δ 0.98 – 1.06 (m, 3H), 1.27 – 1.40 (m, 1H), 1.63 – 1.77 (m, 1H), 1.80 – 1.93 (m, 1H), 1.96 – 2.12 (m, 1H), 2.12 – 2.25 (m, 1H), 2.72 – 3.20 (m, 1H), 3.39 – 3.47 (m, 1H), 3.93 – 4.01 (m, 3H), 4.95 – 5.66 (m, 1H), 6.68 – 6.81 (m, 2H), 7.06 – 7.21 (m, 2H), 7.68 – 7.82 (m, 2H), 8.41 – 8.49 (m, 1H), 8.50 – 8.60 (m, 1H), 9.37 (s, 1H), 10.86 – 11.11 (m, 1H). LCMS (ESI): [M]+ m/z: calcd 412.4; found 413.4; Rt = 2.861 min.

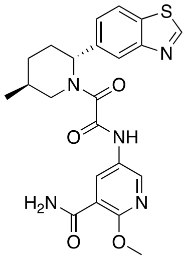

5-(2-((2R,5S)-2-(benzo[d]thiazol-5-yl)-5-methylpiperidin-1-yl)-2-oxoacetamido)-2-methoxynicotinamide (47).
Acid **74** and amine **72**. White solid, 58% yield. RT (IC, Hexane-IPA-MeOH, 50-25-25, 0.6mL/min) = 67.435 min. ^{1}H NMR (600 MHz, DMSO-d_6) δ 1.01 – 1.06 (m, 3H), 1.32 – 1.40 (m, 1H), 1.69 – 1.75 (m, 1H), 1.83 – 1.93 (m, 1H), 2.08 – 2.23 (m, 1H), 2.29 – 2.35 (m, 1H), 2.77 – 3.28 (m, 1H), 3.50 – 4.09 (m, 4H), 5.26 – 5.75 (m, 1H), 7.41 – 7.51 (m, 1H), 7.65 – 7.78 (m, 2H), 8.00 – 8.04 (m, 1H), 8.14 – 8.20 (m, 1H), 8.38 – 8.57 (m, 2H), 9.35 – 9.44 (m, 1H), 10.98 – 11.21 (m, 1H). LCMS (ESI): $[M+H]^+$ m/z: calcd 453.1; found 454.2; Rt = 2.672 min.

General Procedure D:

A mixture of amine (1 eq), **69** (1 eq) and triethylamine (10 eq) in DMF (0.2 M) was stirred at 25 °C for 15 minutes, then HATU (1 eq) was added. The reaction mixture was stirred at 25 °C for 2 h, then concentrated in vacuo to purified by reverse phase HPLC to afforded the racemic

amide, which was then separated by preparative chiral HPLC (Column: Chiralpak IC (250 x 20 mm, 5 µm); CO_2-MeOH, 55-45. Flow Rate: 35 mL
/min; Column Temperature: 40 °C; Wavelength: 215 nm) to afford separate isomers which were used directly in the next step by dissolving in a mixture of 1:1 H_2O:1,4-dioxane and stirred at 95 °C for 18 h then cooled and purified by reverse phase HPLC to afford desired oxamide product.

Compounds 28, 30, 32, 34, 36, 38, 40, 42.

The following compounds were prepared through General Procedure D:

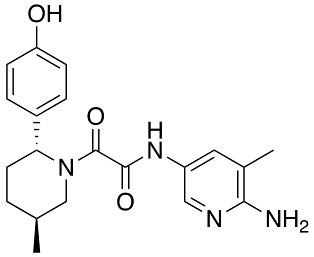

N-(6-amino-5-methylpyridin-3-yl)-2-((2R,5S)-2-(4-hydroxyphenyl)-5-methylpiperidin-1-yl)-2-oxoacetamide (28).
Amine **69.** White solid, 2% yield over 2 steps and separation of isomers. ^{1}H NMR (600 MHz, DMSO-d_6) δ 0.96 – 1.01 (m, 3H), 1.26 – 1.36 (m, 1H), 1.63 – 1.75 (m, 1H), 1.78 – 1.89 (m, 1H), 1.93 – 2.04 (m, 4H), 2.11 – 2.19 (m, 1H), 2.68 – 3.18 (m, 1H), 3.37 – 3.96 (m, 1H), 4.96 – 5.52 (m, 1H), 5.53 – 5.66 (m, 2H), 6.69 – 6.79 (m, 2H), 7.04 – 7.15 (m, 2H), 7.39 – 7.53 (m, 1H), 7.91 – 8.06 (m, 1H), 9.26 – 9.38 (m, 1H), 10.40 – 10.51 (m, 1H). LCMS (ESI): $[M]^+$ m/z: calcd 368.4; found 369.2; Rt = 2.013 min.

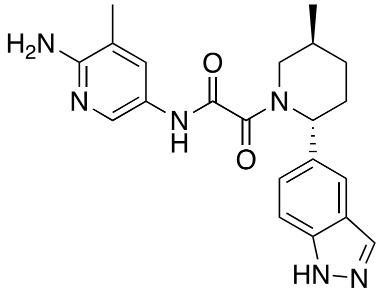

2-((2*R*,5*S*)-2-(1*H*-indazol-5-yl)-5-methylpiperidin-1-yl)-*N*-(6-amino-5-methylpyridin-3-yl)-2-oxoacetamide (30).
Amine **intermediate 30a**. White solid, 20% yield (coupling, deprotection, separation) RT (IA-3, Hexane-IPA-MeOH, 60-20-20, 0.15 mL/min) = 16.782 min. ^{1}H NMR (600 MHz, dmso) δ 0.98 – 1.03 (m, 3H), 1.29 – 1.40 (m, 1H), 1.71 – 1.79 (m, 1H), 1.82 – 1.94 (m, 1H), 1.96 – 2.04 (m, 3H), 2.05 – 2.20 (m, 1H), 2.22 – 2.30 (m, 1H), 2.74 – 3.23 (m, 1H), 3.41 – 4.03 (m, 1H), 5.20 – 5.58 (m, 1H), 5.59 – 5.70 (m, 2H), 7.24 – 7.38 (m, 1H), 7.40 – 7.57 (m, 2H), 7.67 – 7.73 (m, 1H), 7.93 – 8.07 (m, 2H), 10.43 – 10.55 (m, 1H), 12.94 – 13.08 (m, 1H).
LCMS (ESI): [M+H]+ m/z: calcd 392.2; found 393.2; Rt = 2.227 min.

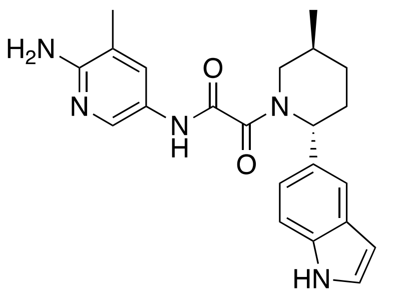

2-((2R,5S)-2-(1H-indol-5-yl)-5-methylpiperidin-1-yl)-N-(6-amino-5-methylpyridin-3-yl)-2-oxoacetamide (32).
Amine **intermediate 32a**. White solid, yield 18% (coupling, deprotection, separation). (column: IC, Hexane-IPA-MeOH, 60-20-20, 0.6 mL/min as mobile phase Retention time: 19.53 min
^{1}H NMR (600 MHz, DMSO-*d6*) δ 1.00 – 1.03 (m, 3H), 1.26 – 1.39 (m, 1H), 1.74 – 1.90 (m, 2H), 1.96 – 2.03 (m, 3H), 2.04 – 2.21 (m, 1H), 2.23 – 2.32 (m, 1H), 2.75 – 3.24 (m, 1H), 3.37 – 4.01 (m, 1H), 5.14 – 5.58 (m, 1H), 5.58 – 5.72 (m, 2H), 6.34 – 6.42 (m, 1H), 6.96 – 7.12 (m, 1H), 7.29 – 7.32 (m, 1H), 7.33 – 7.39 (m, 1H), 7.41 – 7.47 (m, 1H), 7.48 – 7.52 (m, 1H), 7.91 – 8.09 (m, 1H), 10.41 – 10.58 (m, 1H), 11.03 (s, 1H).
LCMS (ESI): [M]+ m/z: calcd 391.2; found 392.2; Rt = 2.214 min.

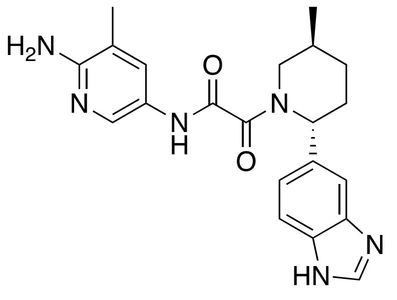

2-((2R,5S)-2-(1H-benzo[d]imidazol-5-yl)-5-methylpiperidin-1-yl)-N-(6-amino-5-methylpyridin-3-yl)-2-oxoacetamide (34).
Amine **intermediate 34a**. White solid, 5% yield (coupling, deprotection, separation). ^{1}H NMR (500 MHz, DMSO-*d6*) δ 1.03 – 1.06 (m, 3H), 1.29 – 1.45 (m, 1H), 1.71 – 1.83 (m, 1H), 1.82 – 1.94 (m, 1H), 1.94 – 2.19 (m, 4H), 2.20 – 2.35 (m, 1H), 2.77 – 3.28 (m, 1H), 3.47 – 4.08 (m, 1H), 5.23 – 5.62 (m, 1H), 5.61 – 5.78 (m, 2H), 7.12 – 7.27 (m, 1H), 7.40 – 7.56 (m, 2H), 7.59 – 7.68 (m, 1H), 7.94 – 8.10 (m, 1H), 8.18 – 8.24 (m, 1H), 10.48 – 10.59 (m, 1H), 12.33 – 12.47 (m, 1H). LCMS (ESI): [M]+ m/z: calcd 392.4; found 393.2; Rt = 1.857 min.

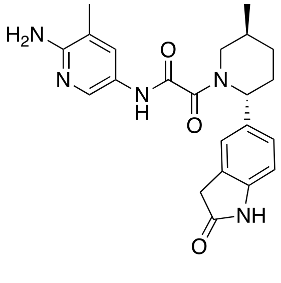

N-(6-amino-5-methylpyridin-3-yl)-2-((2R,5S)-5-methyl-2-(2-oxoindolin-5-yl)piperidin-1-yl)-2-oxoacetamide (36).

Amine **intermediate 36a**. White solid, 2% yield over 2 steps and separation of isomers. ^{1}H NMR (DMSO-d_6, 600 MHz): δ 1.00 (m, 3H), 1.30 (m, 1H), 1.70 (m, 1H), 1.85 (m, 1H), 1.99 (m, 4H), 2.15 (m, 1H), 3.04 (m, 1H), 3.43 (m, 3H), 5.56 (m, 3H), 6.80 (m, 1H), 7.10 (m, 2H), 7.44 (m, 1H), 7.98 (m, 1H), 10.40 (m, 2H).
LCMS (ESI): [M+H]+ m/z: calcd 407.2; found 408.0 ; Rt = 1.634 min.

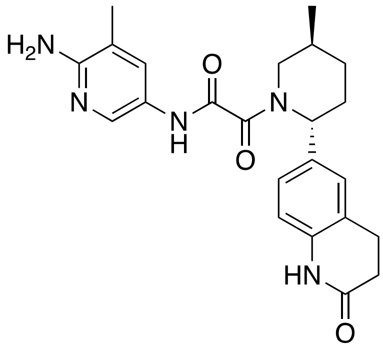

N-(6-amino-5-methylpyridin-3-yl)-2-((2R,5S)-5-methyl-2-(2-oxo-1,2,3,4-tetrahydroquinolin-6-yl)piperidin-1-yl)-2-oxoacetamide (38).
Amine: **intermediate 38a**. White solid, 1% yield (coupling, deprotection, purification). ^{1}H NMR (600 MHz, DMSO-d_6) δ 0.98 – 1.20 (m, 3H), 1.26 – 1.37 (m, 1H), 1.63 – 1.75 (m, 1H), 1.80 – 1.92 (m, 1H), 1.95 – 2.07 (m, 4H), 2.11 – 2.21 (m, 1H), 2.40 – 2.43 (m, 2H), 2.72 – 2.75 (m, 0.4H), 2.83 – 2.89 (m, 2H), 3.17 – 3.22 (m, 0.6H), 3.42 – 3.99 (m, 1H), 5.01 – 5.53 (m, 1H), 5.57 – 5.66 (m, 2H), 6.79 – 6.86 (m, 1H), 7.04 – 7.13 (m, 2H), 7.39 – 7.52 (m, 1H), 7.91 – 8.07 (m, 1H), 10.02 (s, 1H), 10.41 – 10.52 (m, 1H). LCMS (ESI): [M+H]+ m/z: calcd 421.2; found 422.4; Rt = 1.868 min.

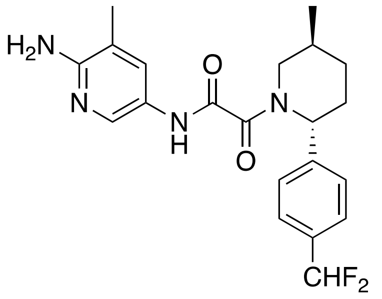

N-(6-amino-5-methylpyridin-3-yl)-2-((2R,5S)-2-(4-(difluoromethyl)phenyl)-5-methylpiperidin-1-yl)-2-oxoacetamide (40).
Amine: **intermediate 40a**. White solid, 1% yield (coupling, deprotection, separation). ^{1}H NMR (500 MHz, DMSO-d_6) δ 0.99 – 1.05 (m, 3H), 1.27 – 1.44 (m, 1H), 1.57 – 1.70 (m, 1H), 1.81 – 1.93 (m, 1H), 1.97 – 2.10 (m, 4H), 2.17 – 2.28 (m, 1H), 2.71 – 3.17 (m, 1H), 3.49 – 4.14 (m, 1H), 5.22 – 5.67 (m, 3H), 6.92 – 7.17 (m, 1H), 7.44 – 7.54 (m, 3H), 7.57 – 7.66 (m, 2H), 7.94 – 8.11 (m, 1H), 10.45 – 10.65 (m, 1H). LCMS (ESI): [M+H]+ m/z: calcd 402.2; found 403.0; Rt = 2.740 min.

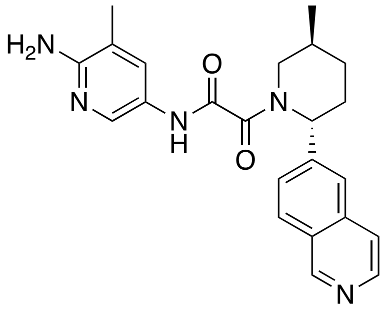

N-(6-amino-5-methylpyridin-3-yl)-2-((2R,5S)-2-(isoquinolin-6-yl)-5-methylpiperidin-1-yl)-2-oxoacetamide (42).
Amine: **intermediate 42a**. White solid, 1% yield (coupling, deprotection, and separation). LCMS (ESI): [M+2H]+ m/z: calcd 403.2; found 404.2; Rt = 1.259 min.

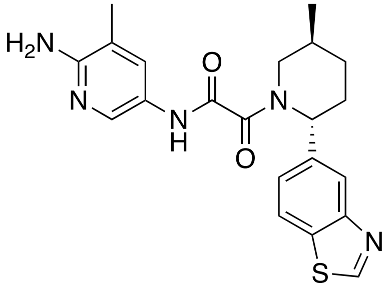

N-(6-amino-5-methylpyridin-3-yl)-2-((2R,5S)-2-(benzo[d]thiazol-5-yl)-5-methylpiperidin-1-yl)-2-oxoacetamide (TNG908).
HATU (491 mg, 1.29 mmol, 1 eq) was added portion wise at room temperature. to a suspension of **69** (381 mg, 1.29 mmol, 1 eq), **72** (300 mg, 1.29 mmol, 1 eq) and triethylamine (1.08 mL, 7.75 mmol, 6 eq) in DMF (10 mL). The clear solution was stirred at 25 °C for 18 h and the solvents were evaporated in vacuo. The residue was dissolved in ethyl acetate (100 mL), washed with H_2O (3x50 mL) and evaporated in vacuo to give tert-butyl N-[5-[[2-[2-(1,3-benzothiazol-5-yl)-5-methyl-1-piperidyl]-2-oxo-acetyl]amino]-3-methyl-2-pyridyl]carbamate (700 mg, crude). ^{1}H NMR (DMSO-d_6, 400 MHz): δ 1.01 (d, 3H), 1.39 (m, 13H), 2.09 (m, 8H), 5.71 (m, 1H), 7.43 (m, 2H), 8.12 (m, 1H), 8.43 (s, 1H), 9.03 (s, 1H), 9.38 (m, 1H), 11.00 (s, 1H). LCMS (ESI): [M+H]+ m/z: calcd 509.2; found 510.2; Rt = 1.319 min. 4.0 M hydrogen chloride solution in dioxane (3.41 mL, 13.74 mmol, 10 eq) was carefully added at room temperature to a solution of this crude material (700 mg, 1.37 mmol, 1 eq) in DCM (10 mL). The reaction mixture was then stirred for 12 h at room temperature, then solvents were evaporated in vacuo. The residue was purified by RP-HPLC (column: YMC Triart C18 100 x 20mm, 5 µm; 40-40-90% 0-1-5min 0.1% NH_3-MeOH as mobile phase) to give racemic N-(6-amino-5-methyl-3-pyridyl)-2-[2-(1,3-benzothiazol-5-yl)-5-methyl-1-piperidyl]-2-oxo-acetamide (331 mg, 0.808 mmol, 59% yield). LCMS (ESI): [M+H]+ m/z: calcd 409.2; found 410.2; Rt = 2.176 min. The racemic mixture was separated by chiral HPLC (column: IC II, Hexane-IPA-MeOH, 50-25-25, 12 mL/min as mobile phase) to give two individual enantiomers N-(6-amino-5-methyl-3-pyridyl)-2-[(2S,5R)-2-(1,3-benzothiazol-5-yl)-5-methyl-1-piperidyl]-2-oxo-acetamide (161 mg, 0.393 mmol, 49% yield) [α]21D = -176.7° (c = 0.1g/100mL, EtOH) and TNG908 (160 mg, 0.390 mmol, 48% yield) [α]21D = +191.5° (c = 0.1g/100mL, EtOH). RT (IC, Hexane-IPA-MeOH, 50-25-25, 0.6 mL/min) = 47.098 min. ^{1}H NMR (600 MHz, DMSO-d_6) δ 0.98 – 1.06 (m, 3H), 1.30 – 1.42 (m, 1H), 1.66 – 1.75 (m, 1H), 1.82 – 1.91 (m, 1H), 1.95 – 2.04 (m, 3H), 2.06 – 2.23 (m, 1H), 2.26 – 2.35 (m, 1H), 2.76 – 3.27 (m, 1H), 3.38 – 4.06 (m, 1H), 5.26 – 5.60 (m, 1H), 5.60 –

5.76 (m, 2H), 7.39 – 7.46 (m, 1H), 7.50 (s, 1H), 7.92 – 8.01 (m, 1H), 8.01 – 8.06 (m, 1H), 8.13 – 8.20 (m, 1H), 9.37 – 9.43 (m, 1H), 10.50 – 10.70 (m, 1H). LCMS (ESI): [M+H]+ m/z: calcd 409.2; found 410.2; Rt = 3.805 min. melting point (instrument: CAS-TJ-MPA-02) 167.3 °C. HRMS (ESI, +vw ion) m/z calcd for C21H24N5O2S+ [M+H+] 410.1645, found 410.1652.

Analytical data for final compounds

Compound 1R

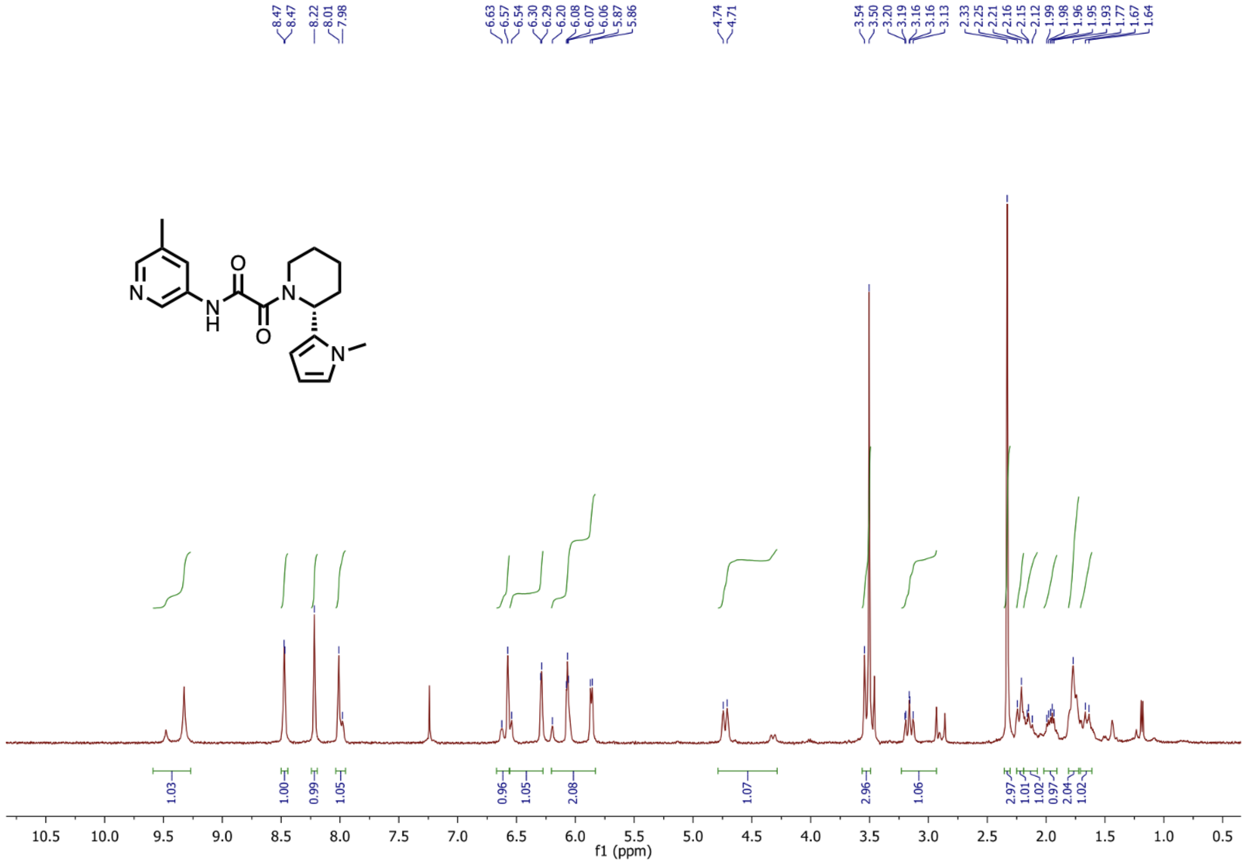

#	Time	Area%
1	4.022	99.05
2	5.566	0.95

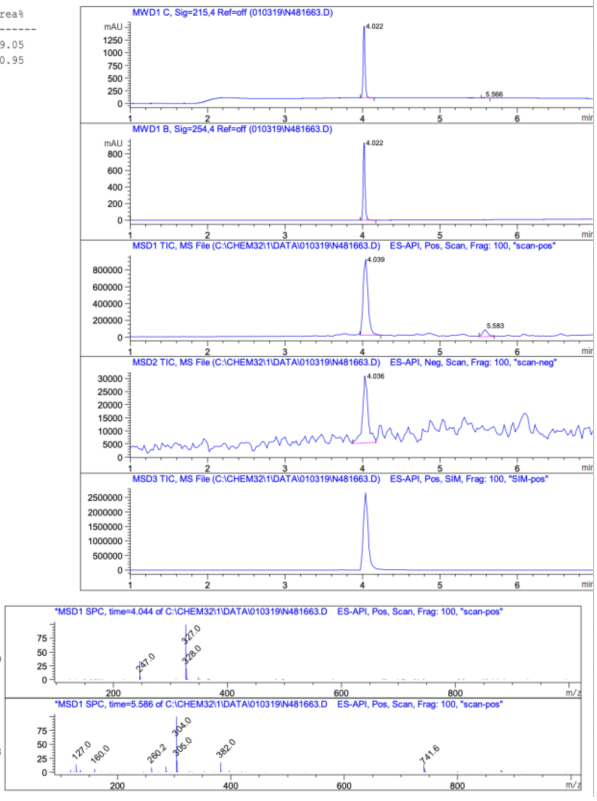

RT 4.039

RT 5.583

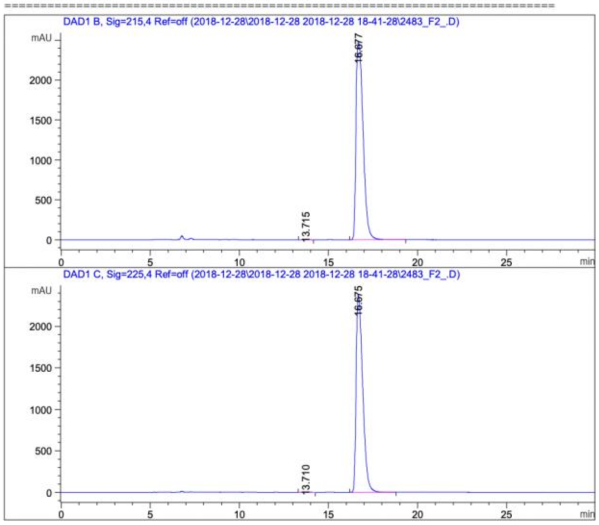

Signal: DAD1 B, Sig=215,4 Ref=off

RetTime (min)	Area, %	Symm.	Resolution	Selectivity
13.7152	0.12	0.860		
16.6772	99.88	0.468	4.86	1.22

Signal: DAD1 C, Sig=225,4 Ref=off

RetTime (min)	Area, %	Symm.	Resolution	Selectivity
13.7102	0.12	0.803		
16.6752	99.88	0.473	5.06	1.22

Compound 1S

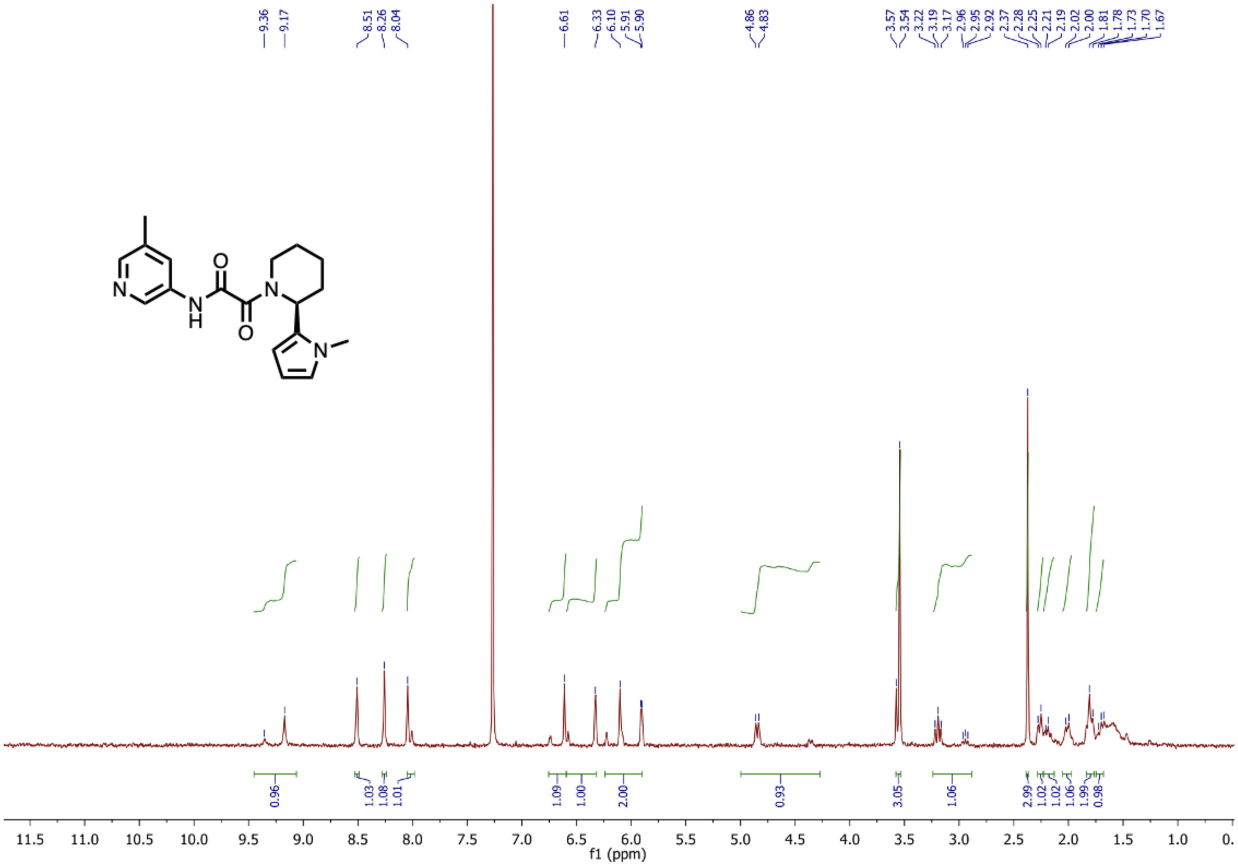

#	Time	Area%
1	4.022	99.48
2	5.569	0.52

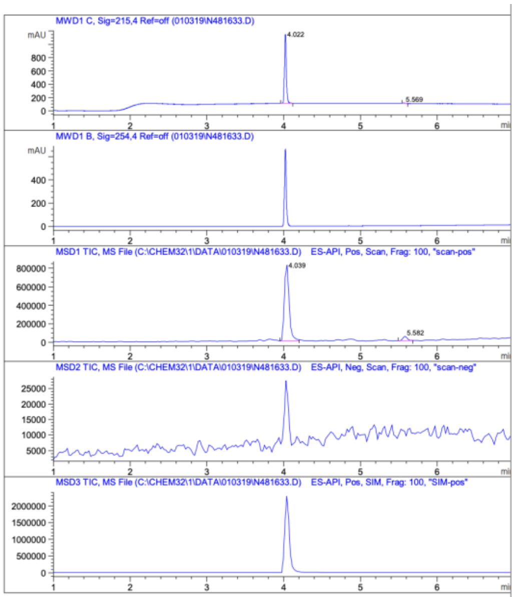

RT 4.039

RT 5.582

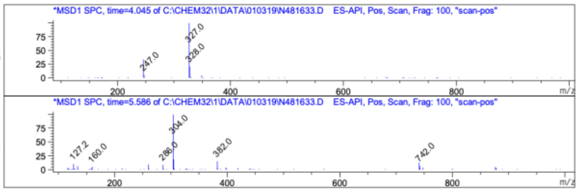

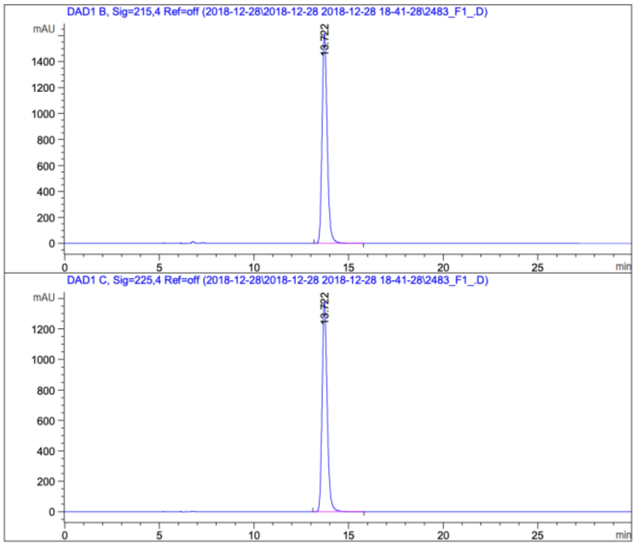

Signal: DAD1 B, Sig=215,4 Ref=off

RetTime (min)	Area, %	Symm.	Resolution	Selectivity
13.7222	100.00	0.782		

Signal: DAD1 C, Sig=225,4 Ref=off

RetTime (min)	Area, %	Symm.	Resolution	Selectivity
13.7222	100.00	0.788		

Compound 1

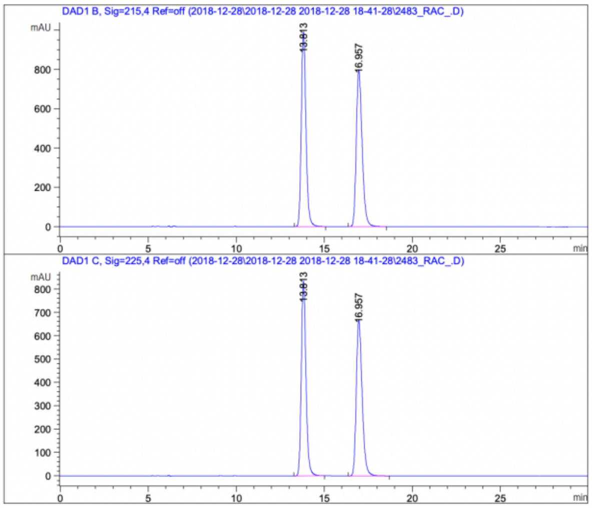

Signal: DAD1 B, Sig=215,4 Ref=off

RetTime (min)	Area, %	Symm.	Resolution	Selectivity
13.8132	49.88	0.805		
16.9572	50.12	0.714	5.89	1.23

Signal: DAD1 C, Sig=225,4 Ref=off

RetTime (min)	Area, %	Symm.	Resolution	Selectivity
13.8132	49.89	0.803		
16.9572	50.11	0.716	5.90	1.23

Compound 2R

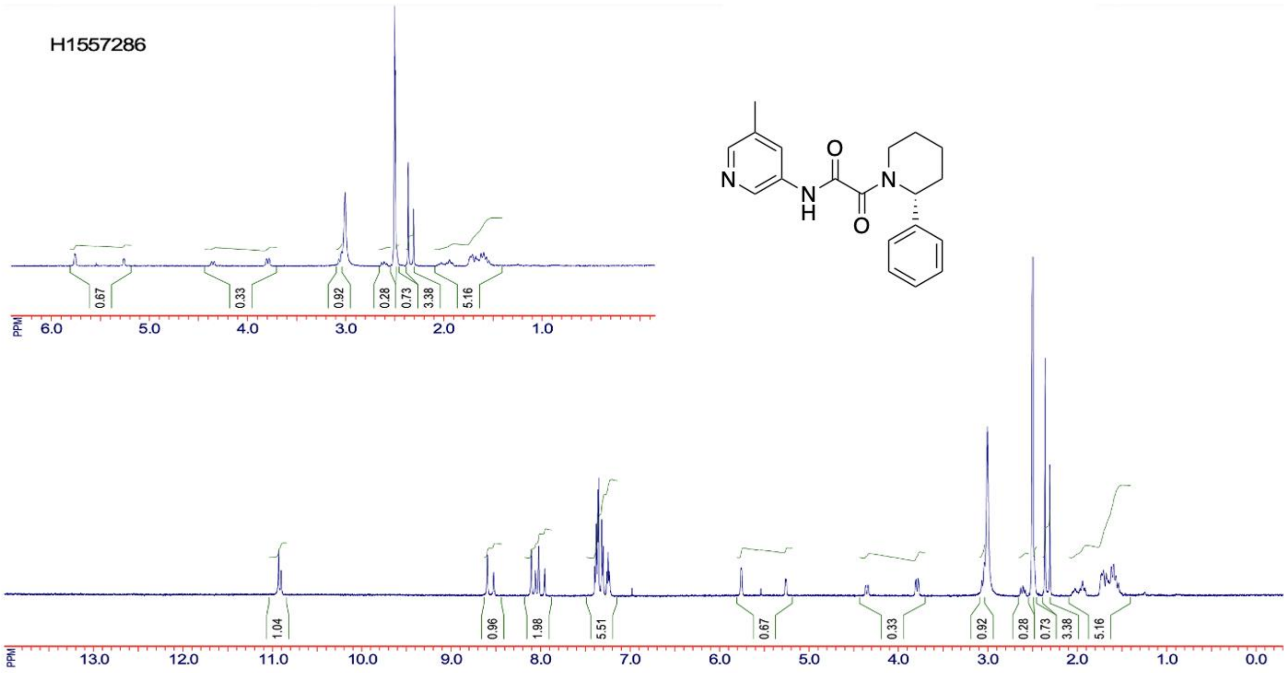

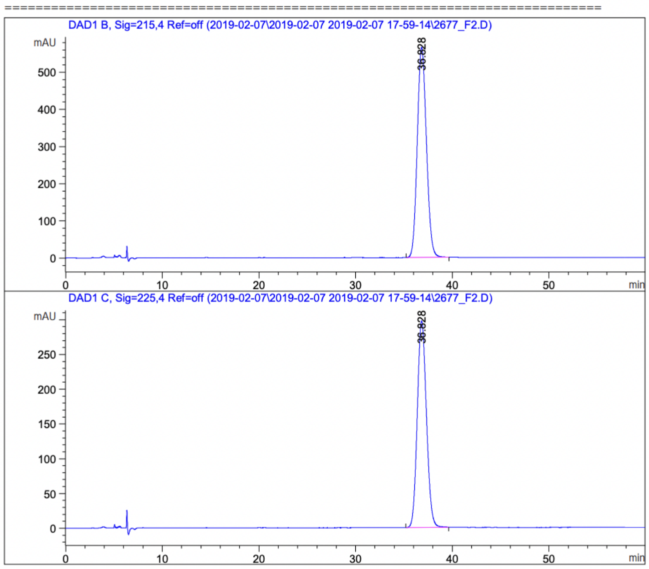

#	Time	Area%
1	4.293	100.00

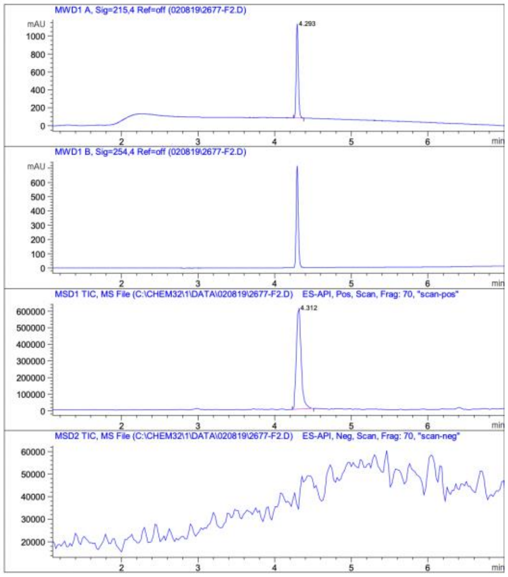

RT 4.312

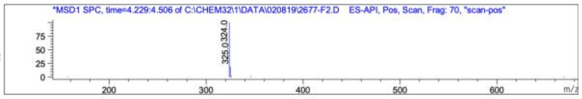

Signal: DAD1 B, Sig=215,4 Ref=off

RetTime(min)	Area,%	Symm.	Resolution	Selectivity
36.8282	100.00	0.860		

Signal: DAD1 C, Sig=225,4 Ref=off

RetTime(min)	Area,%	Symm.	Resolution	Selectivity
36.8282	100.00	0.855		

Compound 2S

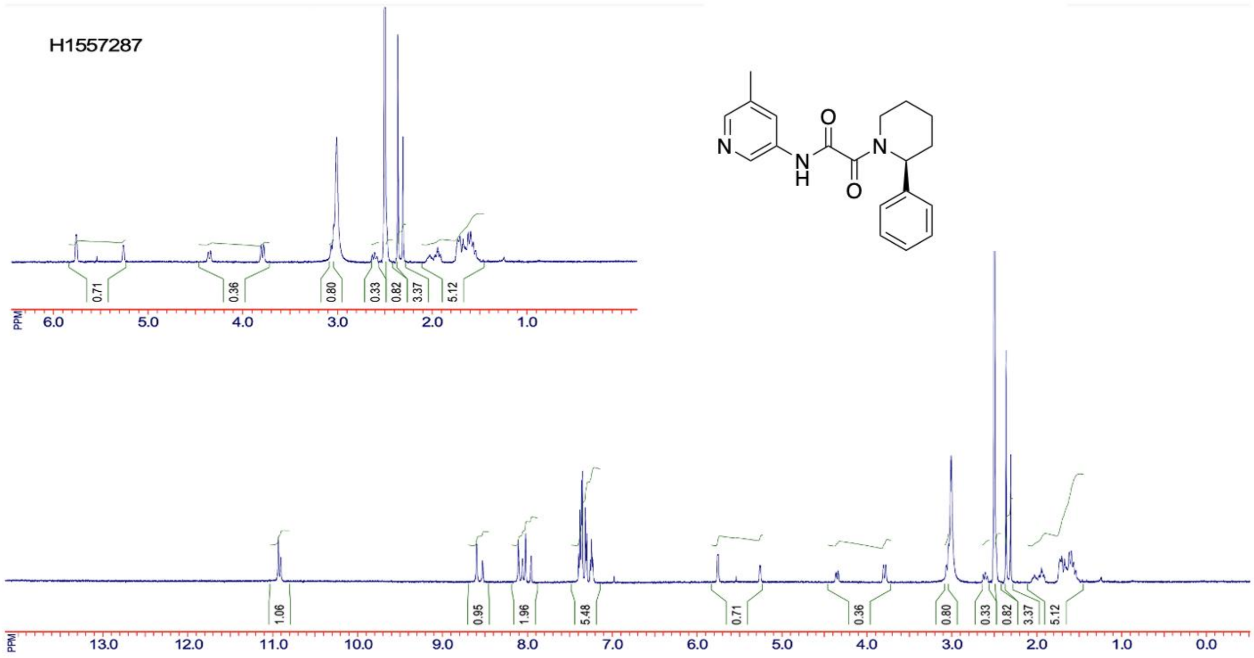


```
#  Time   Area%
----------------
1  4.293  100.00
```

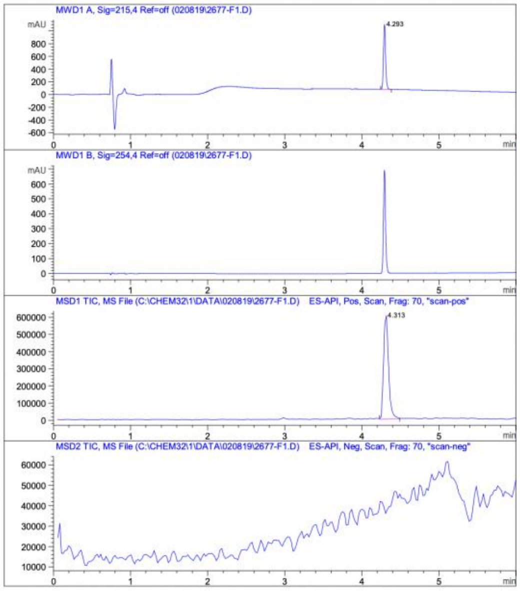

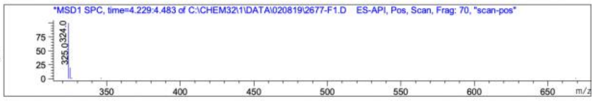

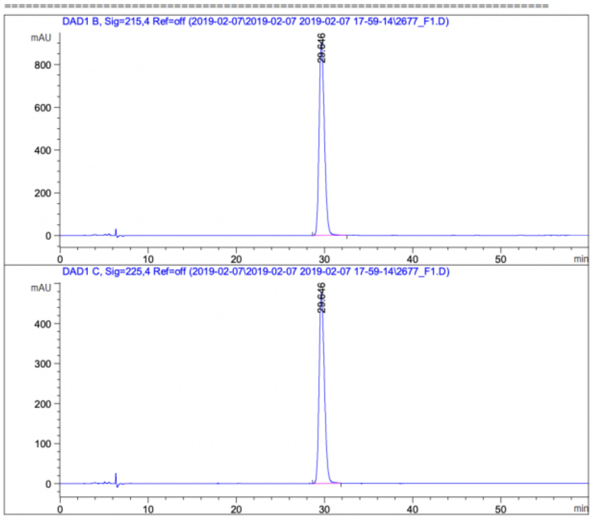

```
Signal:  DAD1 B, Sig=215,4 Ref=off
=====================================================================
RetTime(min)  Area,%    Symm.    Resolution Selectivity
|------------|--------|----------|-----------|-----------|
29.6462        100.00     0.739
=====================================================================

Signal:  DAD1 C, Sig=225,4 Ref=off
=====================================================================
RetTime(min)  Area,%    Symm.    Resolution Selectivity
|------------|--------|----------|-----------|-----------|
29.6462        100.00     0.742
=====================================================================
```

Compound 3

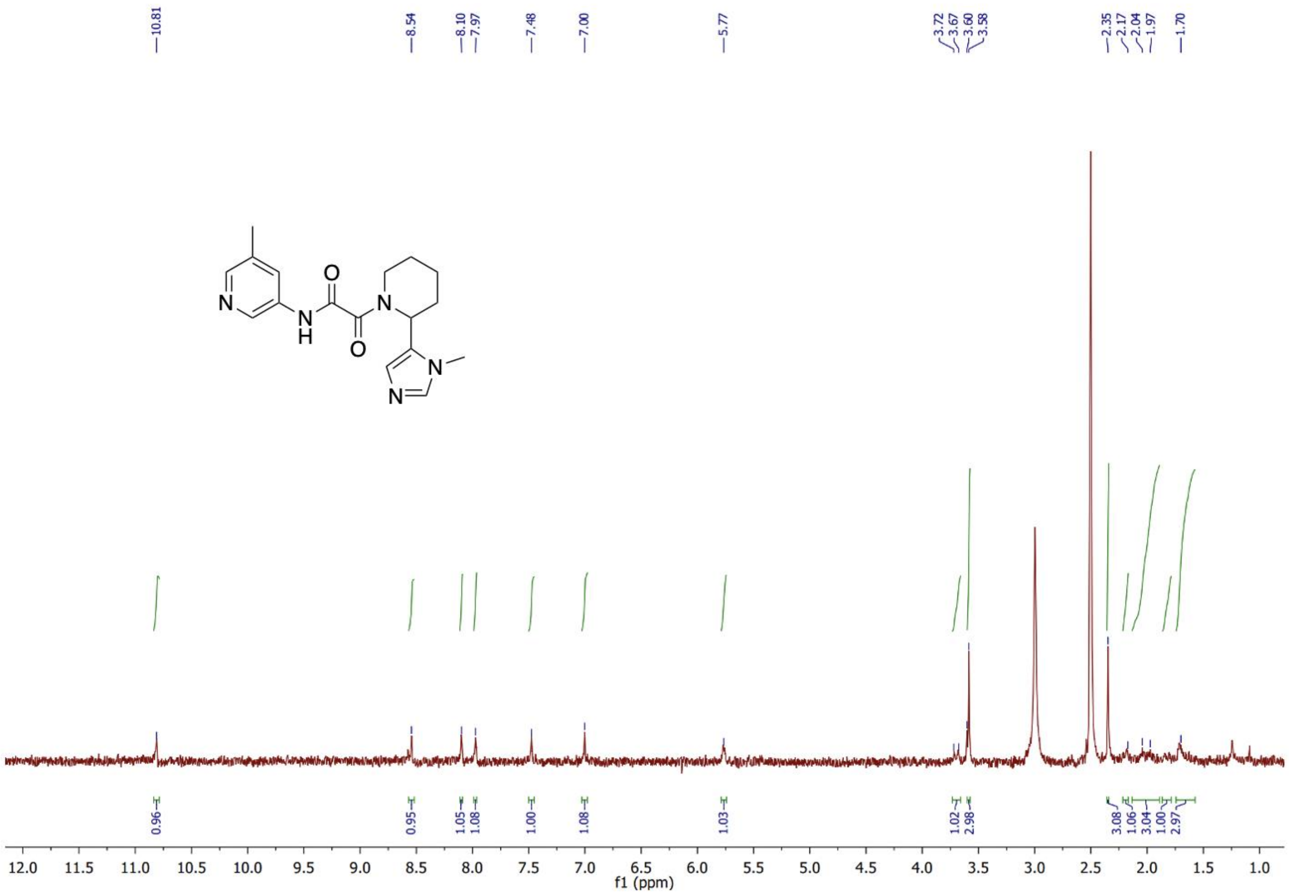
10.81
8.54
8.10
7.97
7.48
7.00
5.77
3.72
3.67
3.60
3.58
2.35
2.17
2.04
1.97
1.70
0.96
0.95
1.05
1.08
1.00
1.08
1.03
1.02
2.98
3.08
1.06
3.04
1.00
2.97
12.0 11.5 11.0 10.5 10.0 9.5 9.0 8.5 8.0 7.5 7.0 6.5 6.0 5.5 5.0 4.5 4.0 3.5 3.0 2.5 2.0 1.5 1.0
f1 (ppm)

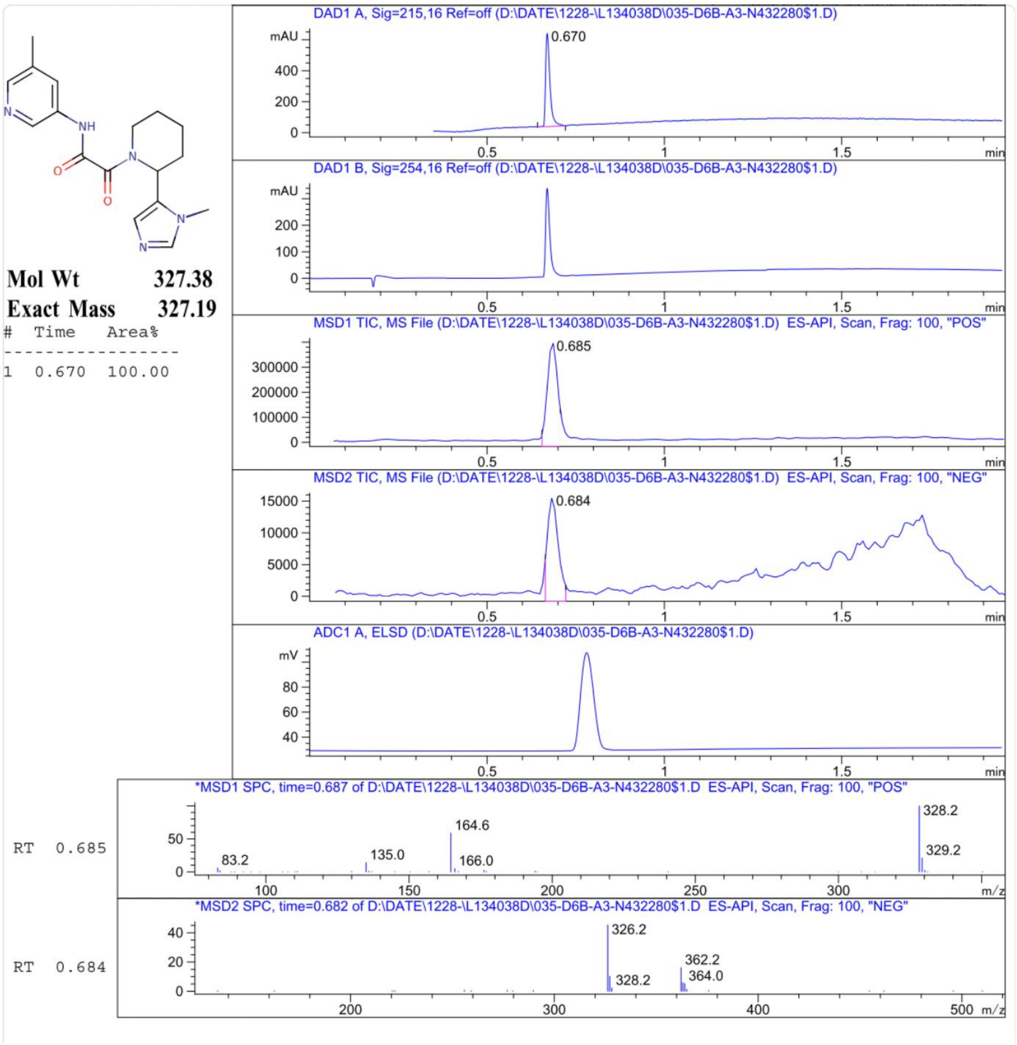
Mol Wt 327.38
Exact Mass 327.19
Time Area%
1 0.670 100.00
DAD1 A, Sig=215,16 Ref=off (D:\DATE\1228-\L134038D\035-D6B-A3-N432280$1.D)
0.670
DAD1 B, Sig=254,16 Ref=off (D:\DATE\1228-\L134038D\035-D6B-A3-N432280$1.D)
MSD1 TIC, MS File (D:\DATE\1228-\L134038D\035-D6B-A3-N432280$1.D) ES-API, Scan, Frag: 100, "POS"
0.685
MSD2 TIC, MS File (D:\DATE\1228-\L134038D\035-D6B-A3-N432280$1.D) ES-API, Scan, Frag: 100, "NEG"
0.684
ADC1 A, ELSD (D:\DATE\1228-\L134038D\035-D6B-A3-N432280$1.D)
*MSD1 SPC, time=0.687 of D:\DATE\1228-\L134038D\035-D6B-A3-N432280$1.D ES-API, Scan, Frag: 100, "POS"
RT 0.685
83.2 135.0 164.6 166.0 328.2 329.2
*MSD2 SPC, time=0.682 of D:\DATE\1228-\L134038D\035-D6B-A3-N432280$1.D ES-API, Scan, Frag: 100, "NEG"
RT 0.684
326.2 328.2 362.2 364.0

Compound 4

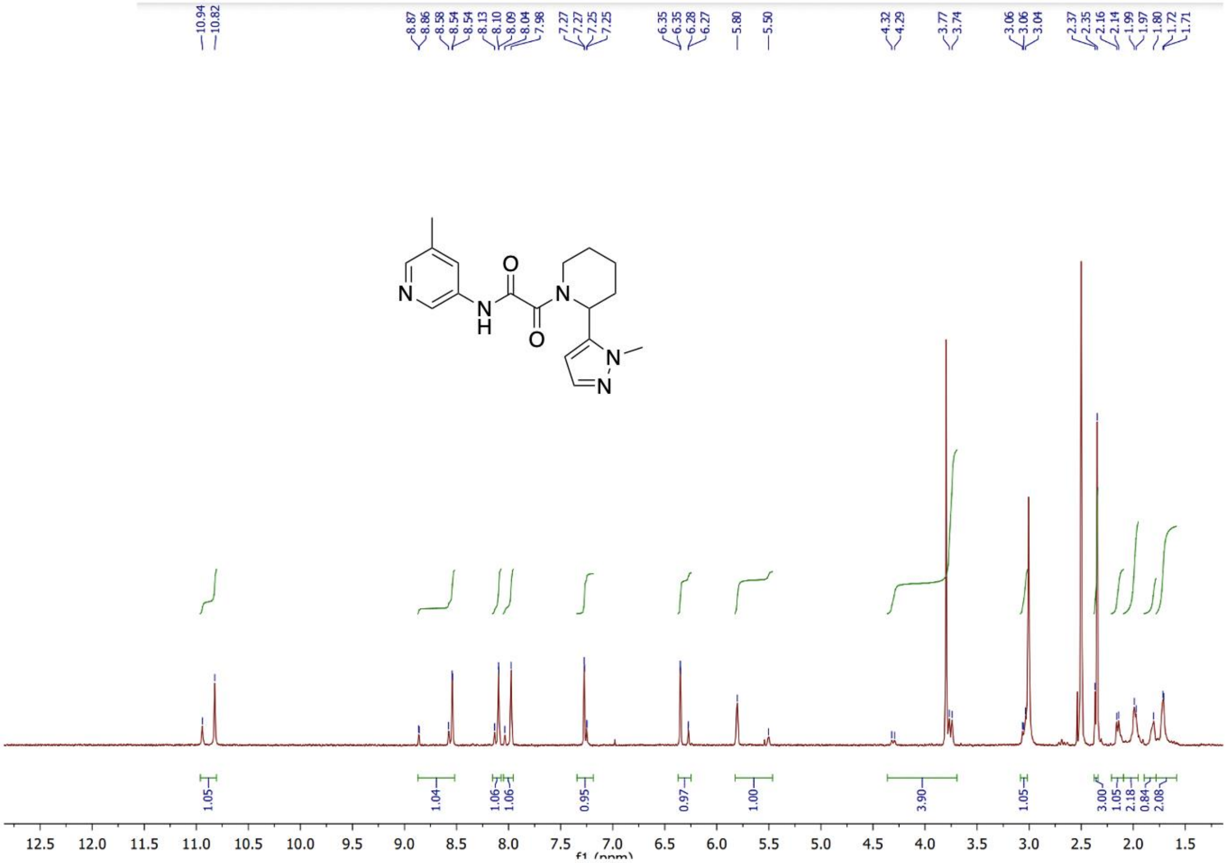
10.94
10.82
8.87
8.86
8.58
8.54
8.54
8.13
8.10
8.09
8.04
7.98
7.27
7.27
7.25
7.25
6.35
6.35
6.28
6.27
5.80
5.50
4.32
4.29
3.77
3.74
3.06
3.06
3.04
2.37
2.35
2.16
2.14
1.99
1.97
1.80
1.72
1.71
1.05
1.04
1.06
1.06
0.95
0.97
1.00
3.90
1.05
3.00
1.05
2.18
0.84
2.08
f1 (ppm)

MaxPeak: 98.09%
Ret_Time: 0.904 min

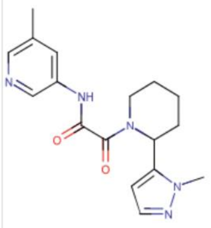

Mol Wt 327.38
Exact Mass 327.19

#	Time	Area%
1	0.692	1.91
2	0.904	98.09

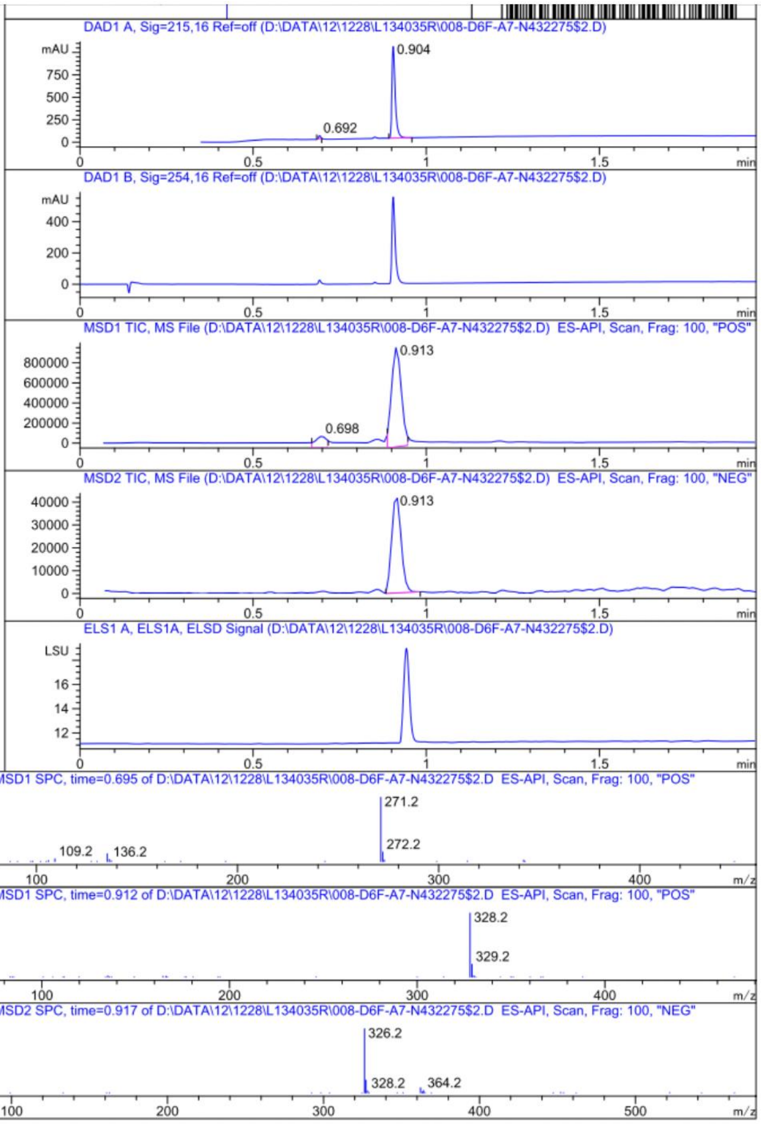

Compound 5

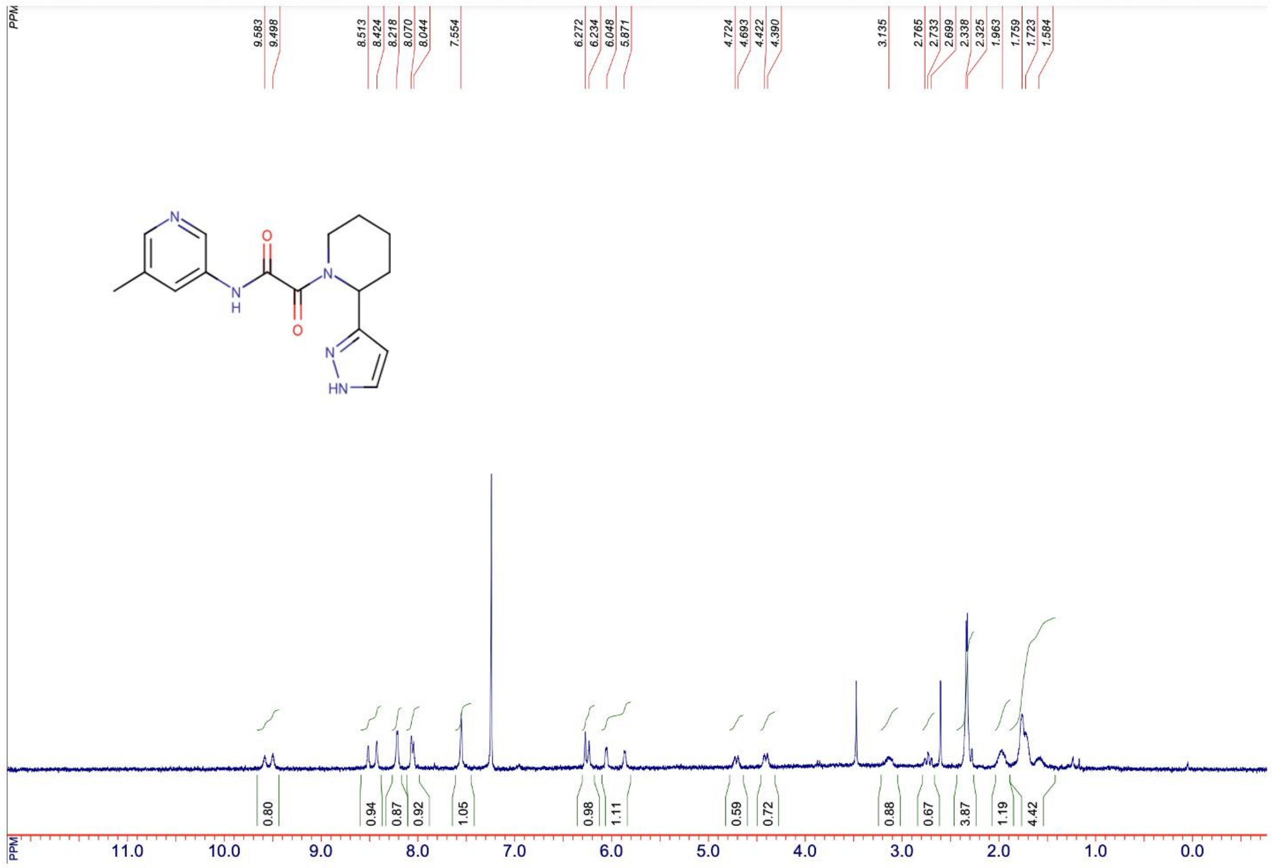

PPM
9.583
9.498
8.513
8.424
8.218
8.070
8.044
7.554
6.272
6.234
6.048
5.871
4.724
4.693
4.422
4.390
3.135
2.765
2.733
2.699
2.338
2.325
1.963
1.759
1.723
1.584
N
O
N
H
O
N
N
HN
0.80
0.94
0.87
0.92
1.05
0.98
1.11
0.59
0.72
0.88
0.67
3.87
1.19
4.42
PPM
11.0
10.0
9.0
8.0
7.0
6.0
5.0
4.0
3.0
2.0
1.0
0.0

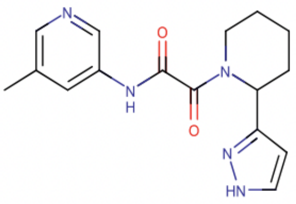

Mol Wt 313.35

Exact Mass 313.17

#	Time	Area%
1	2.096	96.09
2	2.561	3.91

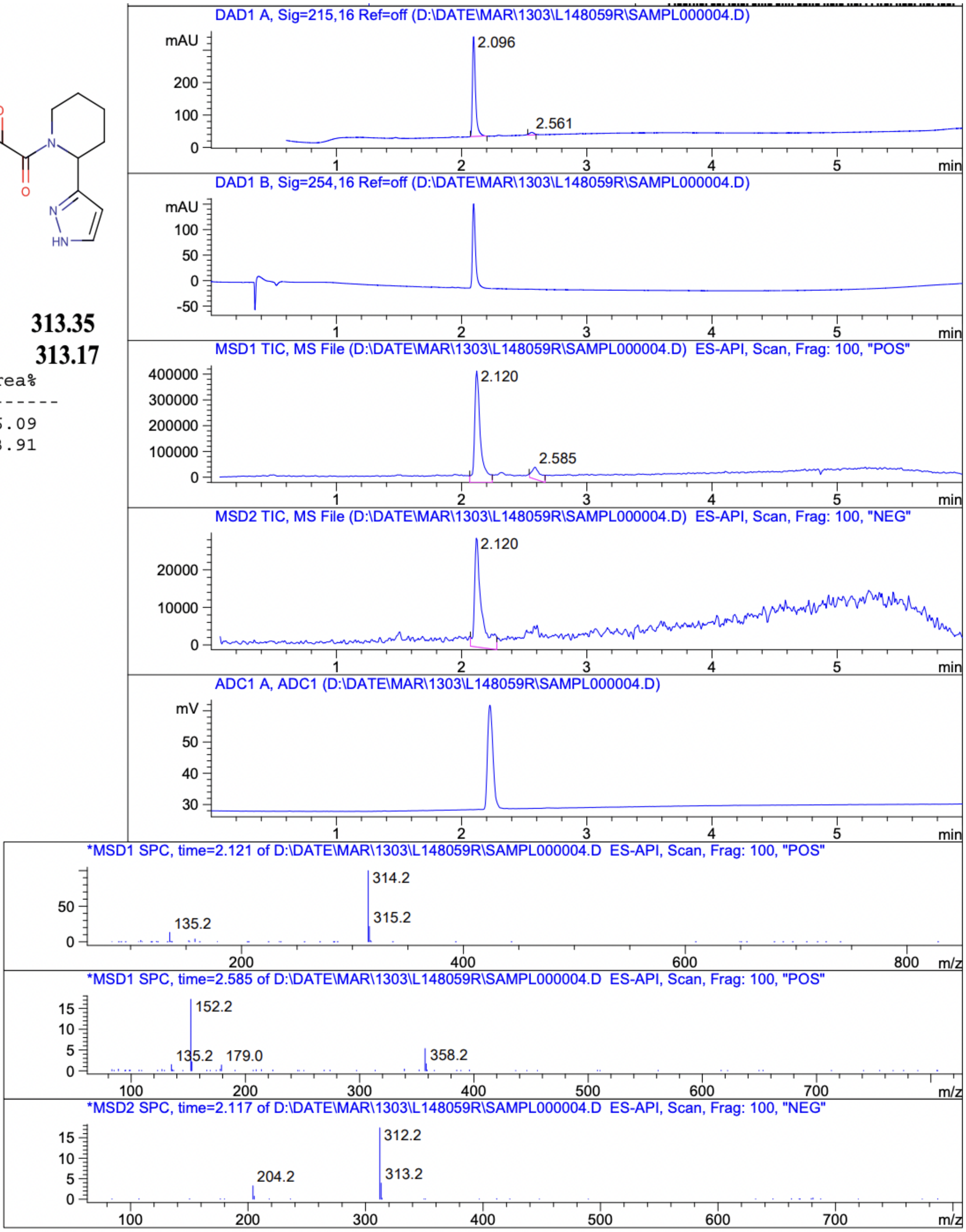

RT 2.120

RT 2.585

RT 2.120

Compound 6

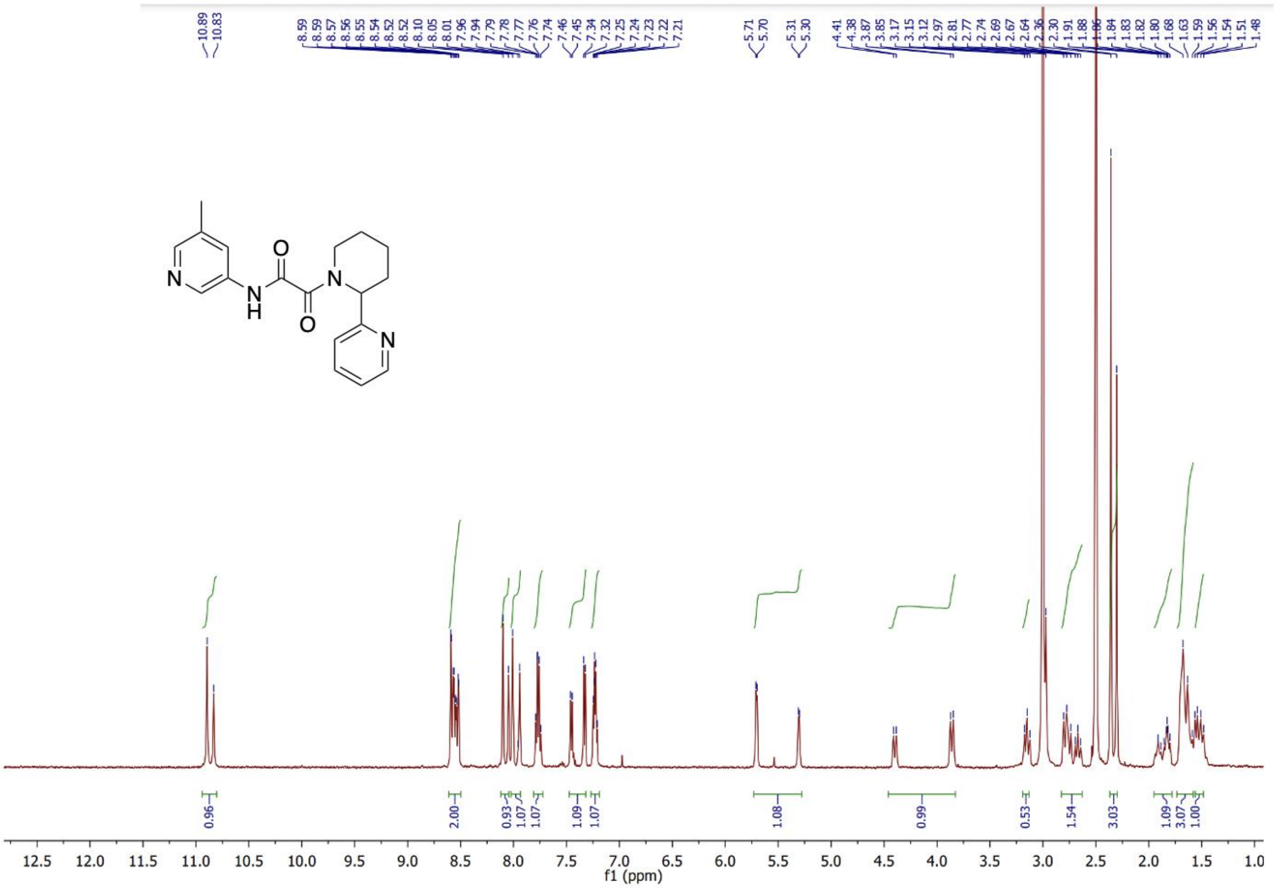

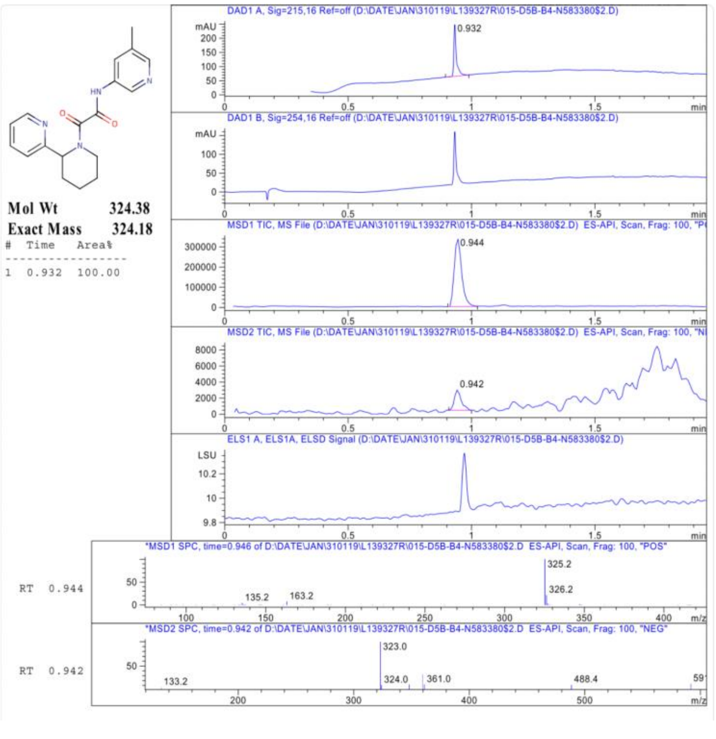

Compound 7

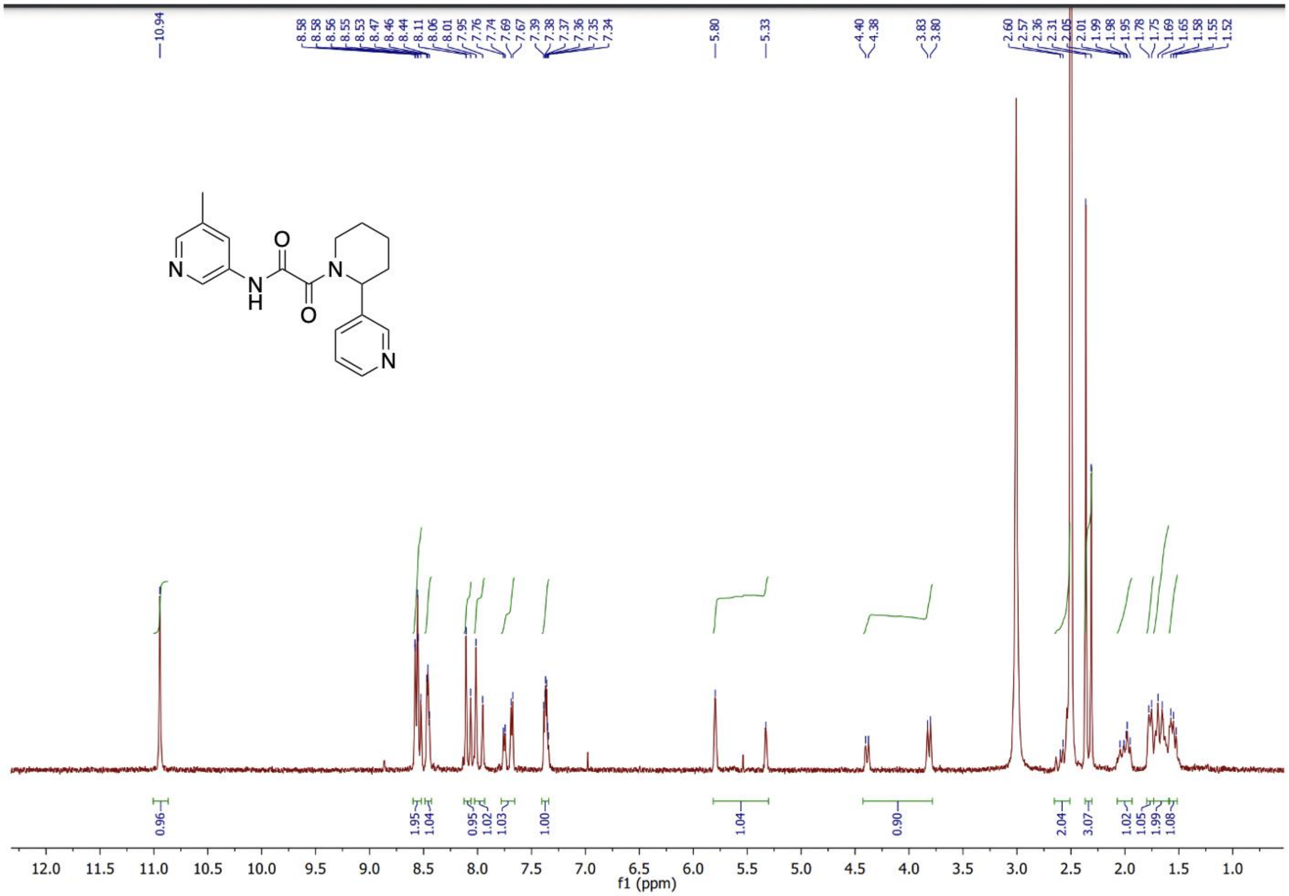

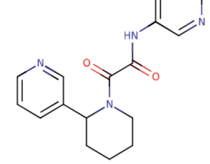

Mol Wt 324.38
Exact Mass 324.18

#	Time	Area%
1	0.717	3.18
2	0.757	96.82

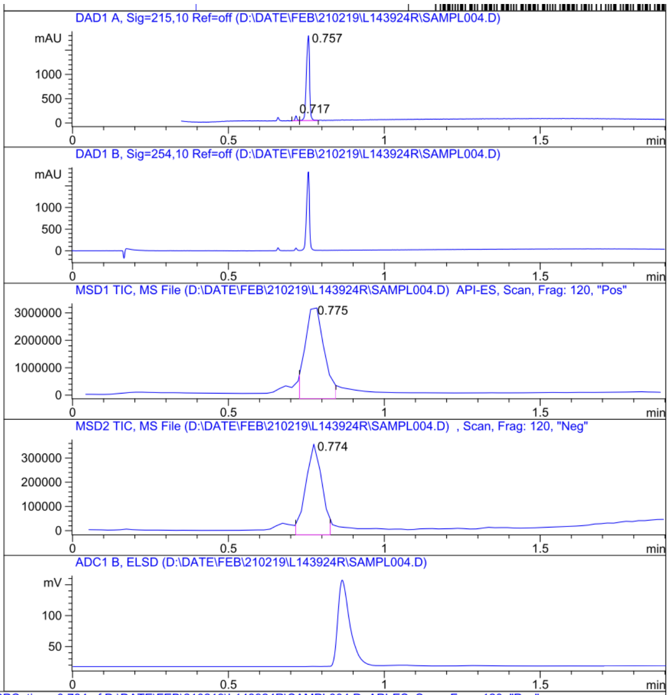

RT 0.775

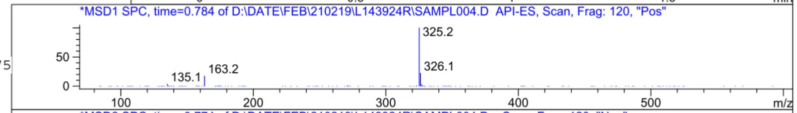

RT 0.774

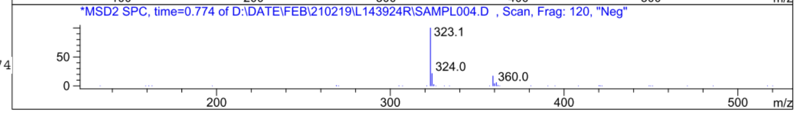

Compound 8

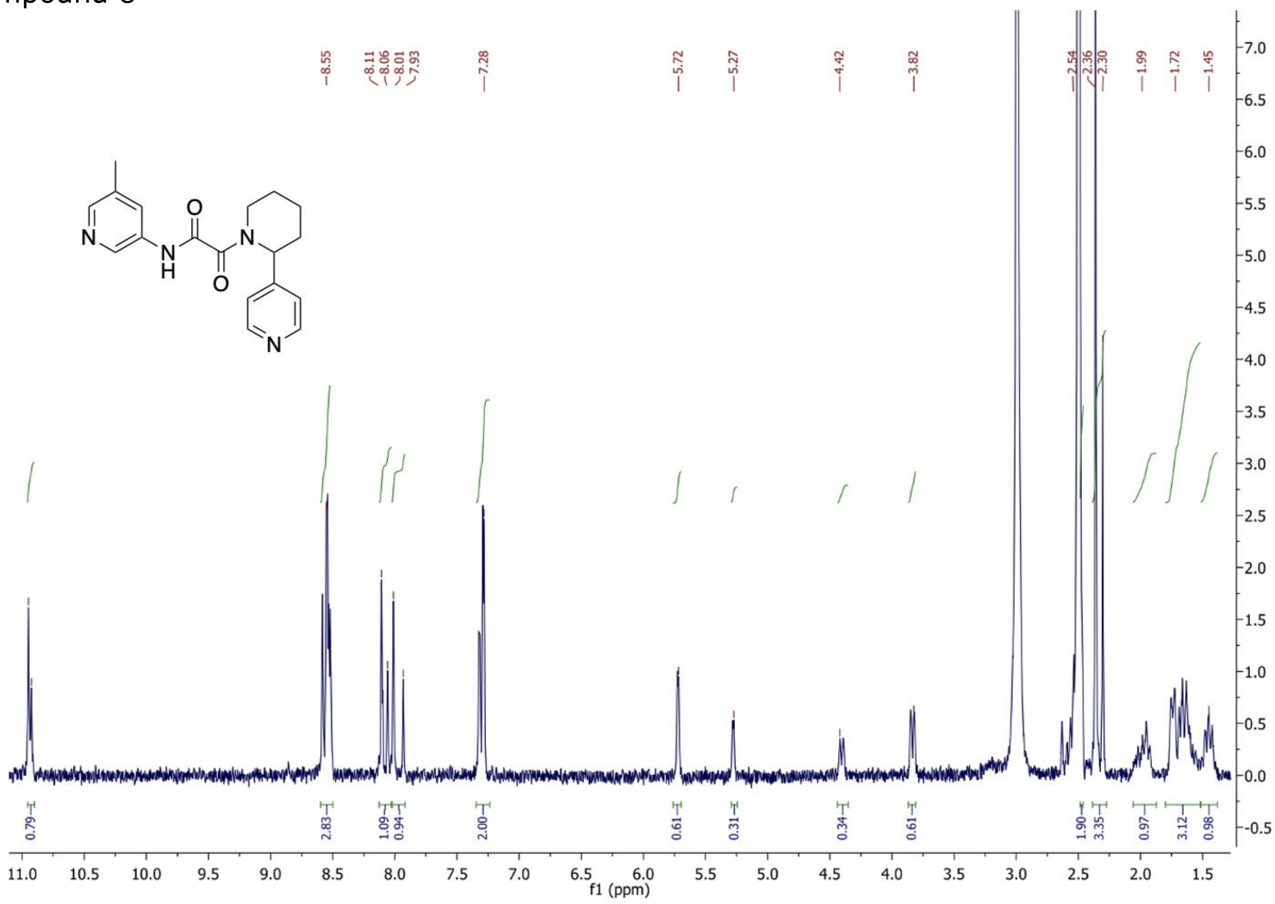

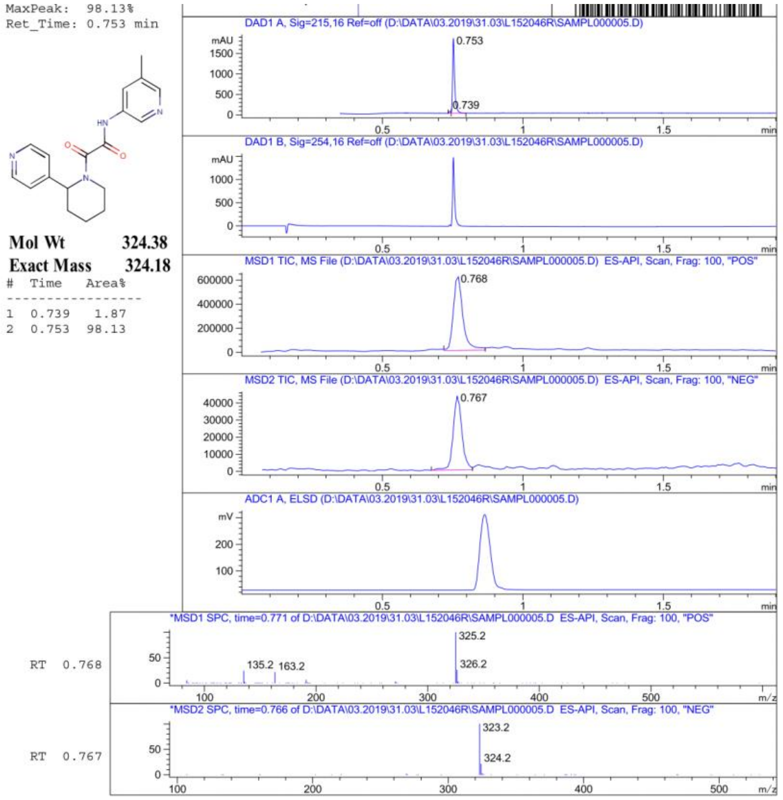

Compound 9

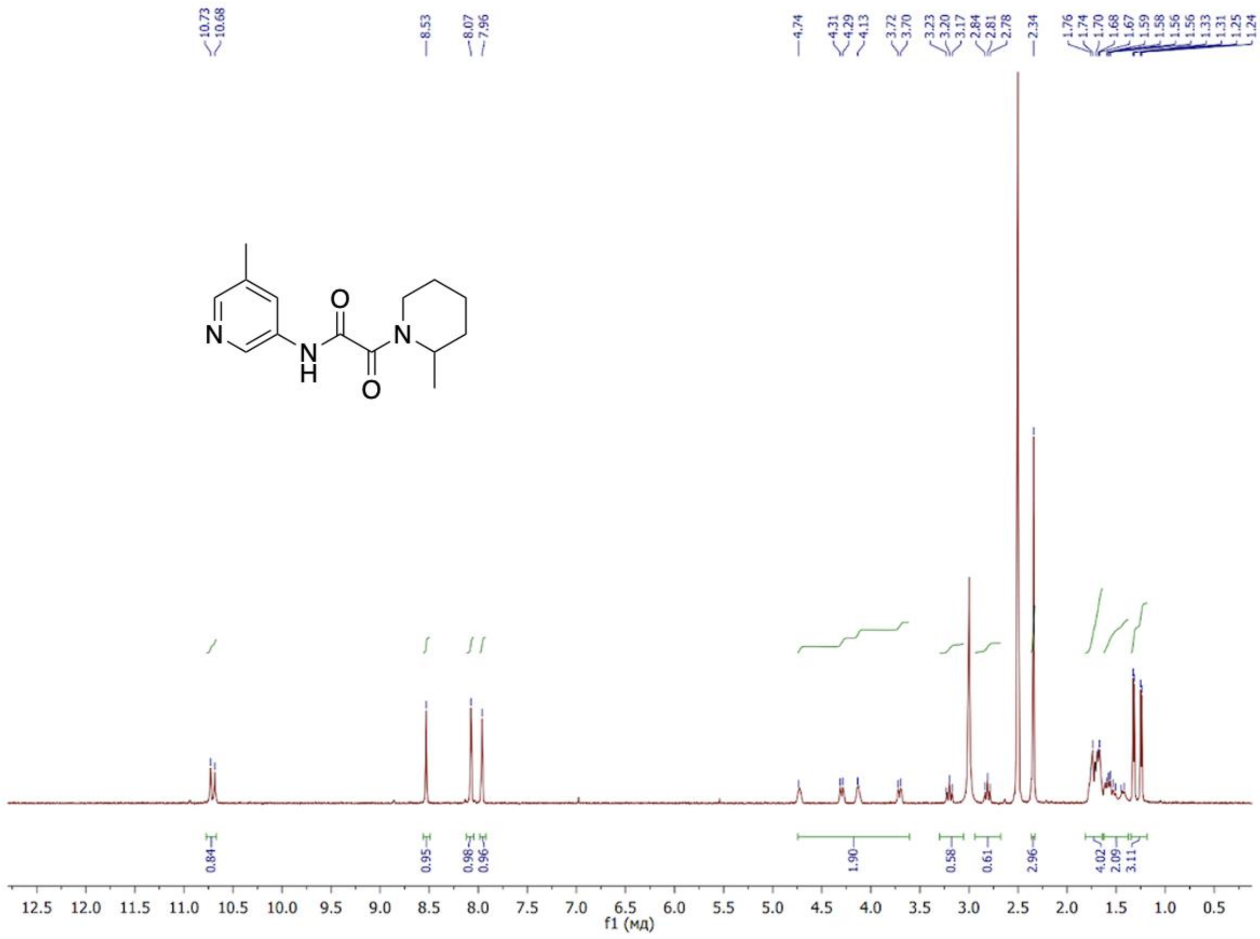
10.73
10.68
8.53
8.07
7.96
4.74
4.31
4.29
4.13
3.72
3.70
3.23
3.20
3.17
2.84
2.81
2.78
2.34
1.76
1.74
1.70
1.68
1.67
1.59
1.58
1.56
1.56
1.33
1.31
1.25
1.24
0.84
0.95
0.98
0.96
1.90
0.58
0.61
2.96
4.02
2.09
3.11
12.5 12.0 11.5 11.0 10.5 10.0 9.5 9.0 8.5 8.0 7.5 7.0 6.5 6.0 5.5 5.0 4.5 4.0 3.5 3.0 2.5 2.0 1.5 1.0 0.5
f1 (мд)

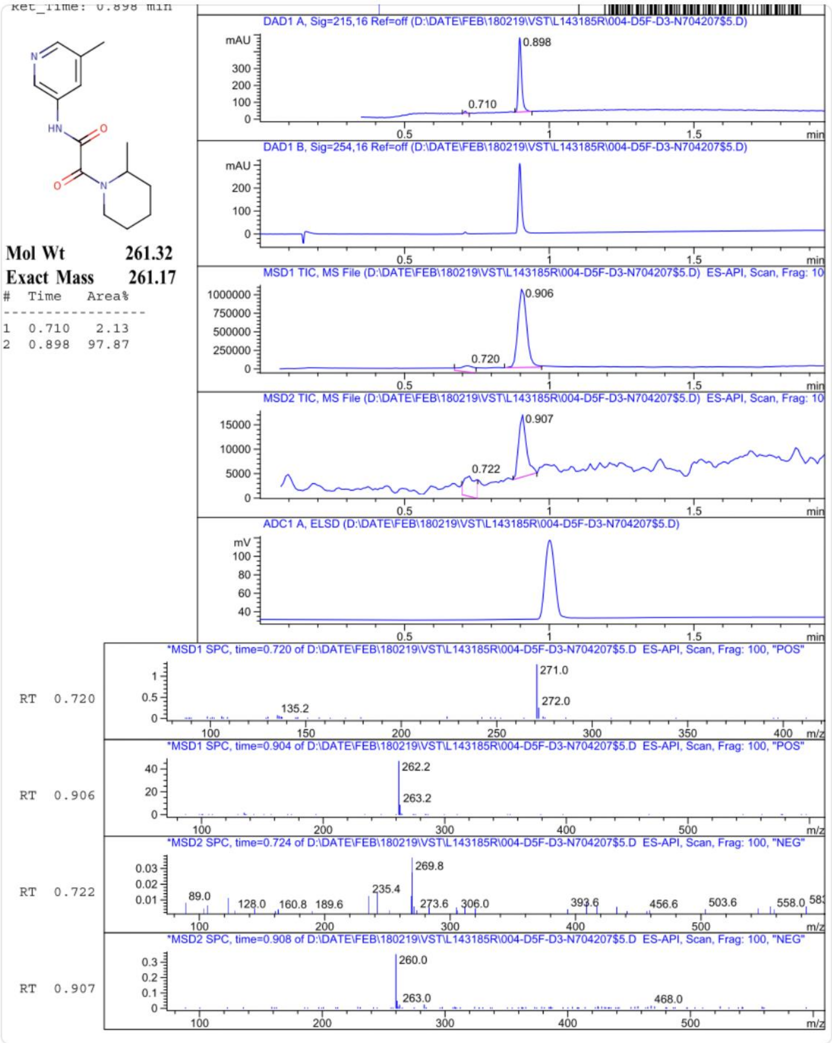
Mol Wt 261.32
Exact Mass 261.17
Time Area%
1 0.710 2.13
2 0.898 97.87
DAD1 A, Sig=215,16 Ref=off (D:\DATE\FEB\180219\VST\L143185R\004-D5F-D3-N704207$5.D)
DAD1 B, Sig=254,16 Ref=off (D:\DATE\FEB\180219\VST\L143185R\004-D5F-D3-N704207$5.D)
MSD1 TIC, MS File (D:\DATE\FEB\180219\VST\L143185R\004-D5F-D3-N704207$5.D) ES-API, Scan, Frag: 10
MSD2 TIC, MS File (D:\DATE\FEB\180219\VST\L143185R\004-D5F-D3-N704207$5.D) ES-API, Scan, Frag: 10
ADC1 A, ELSD (D:\DATE\FEB\180219\VST\L143185R\004-D5F-D3-N704207$5.D)
RT 0.720
RT 0.906
RT 0.722
RT 0.907

Compound 10

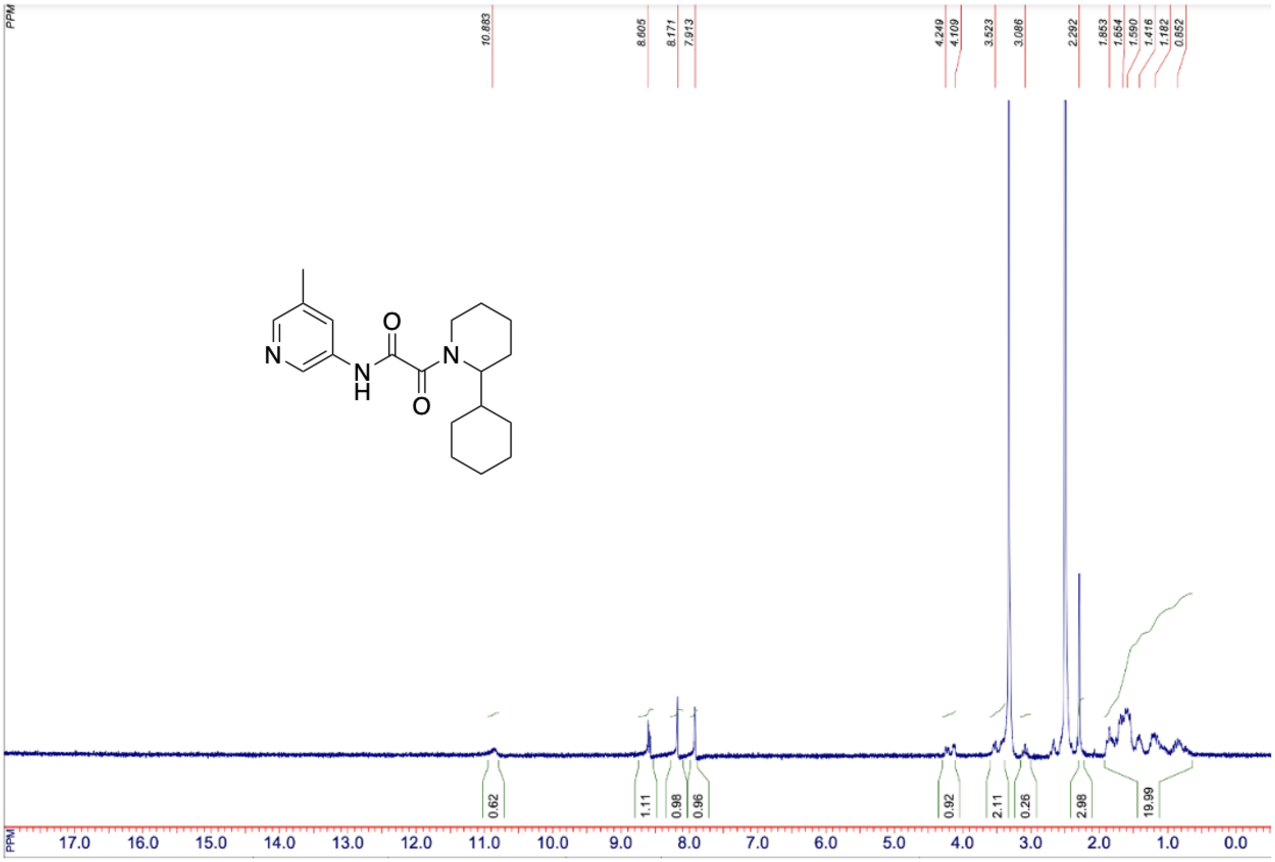

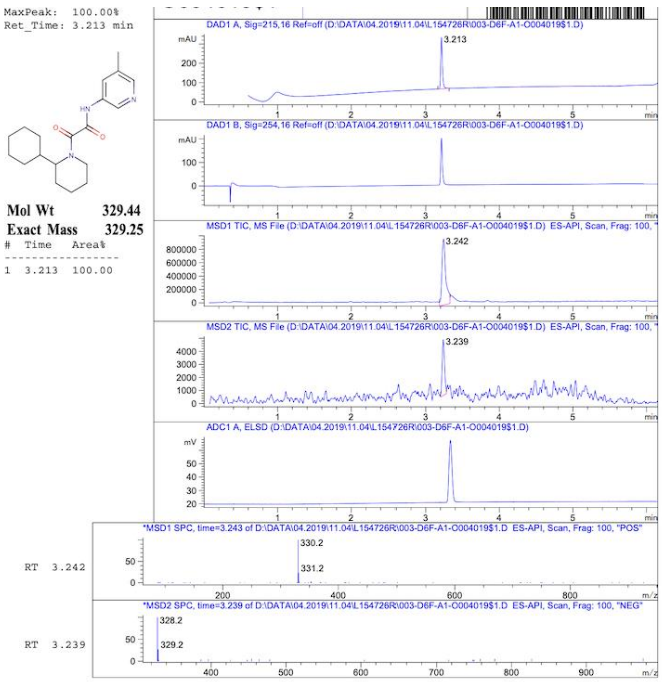

Compound 11

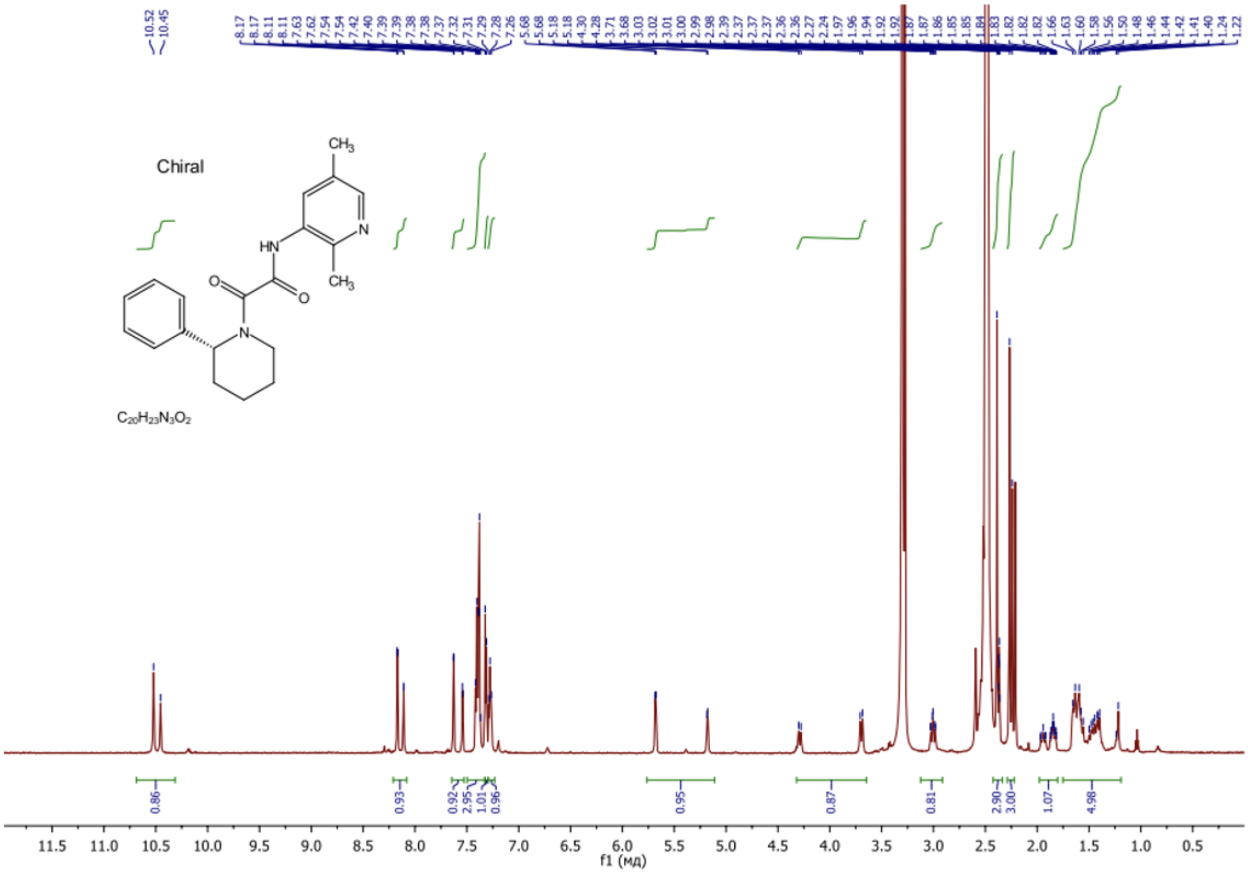
Chiral
C20H23N3O2
f1 (мд)

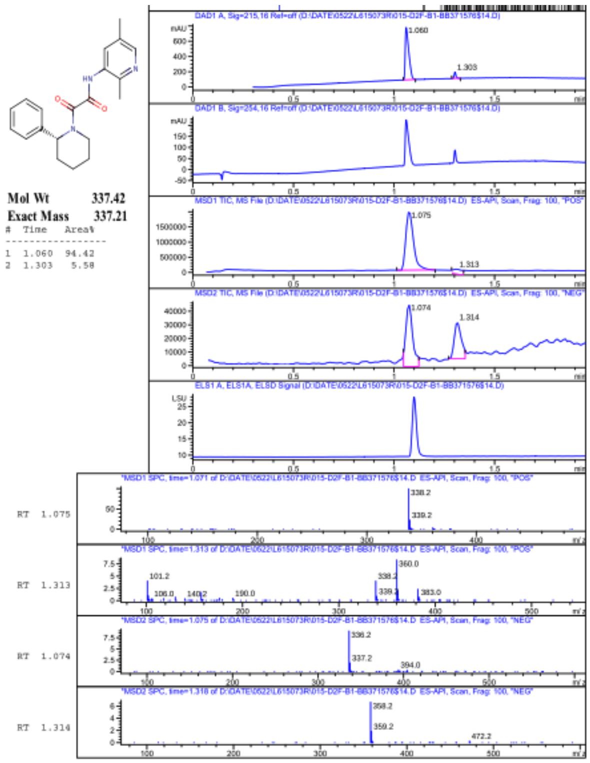
Mol Wt 337.42
Exact Mass 337.21
Time Area%
1 1.060 94.42
2 1.303 5.58
RT 1.075
RT 1.313
RT 1.074
RT 1.314

Compound 12

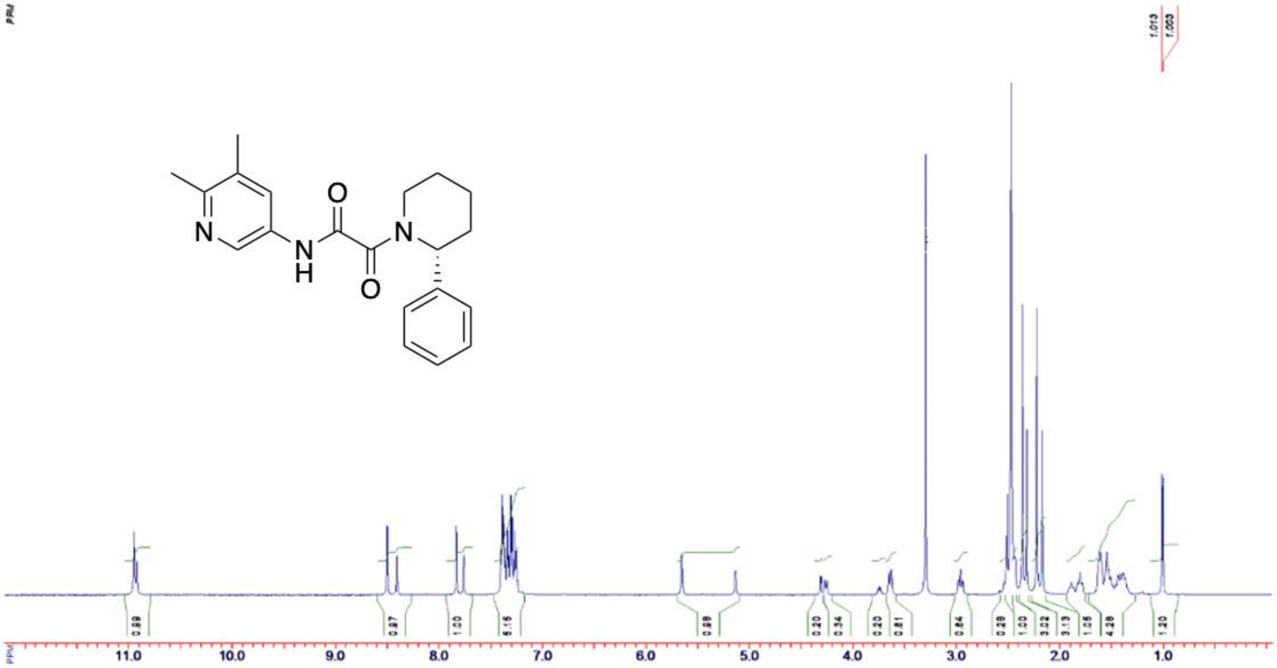

#	Time	Area%
1	3.778	100.00

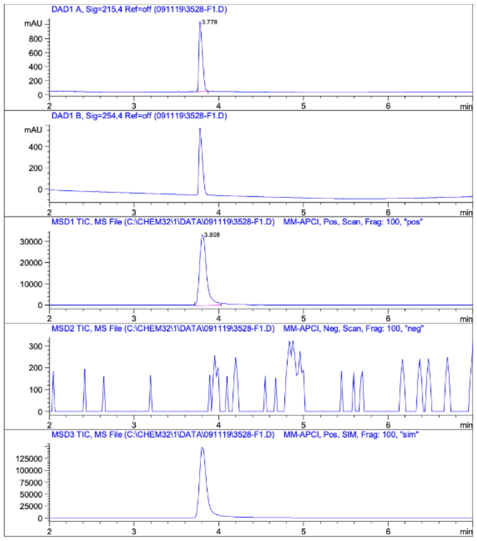

RT 3.808

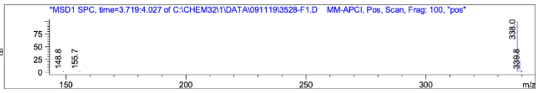

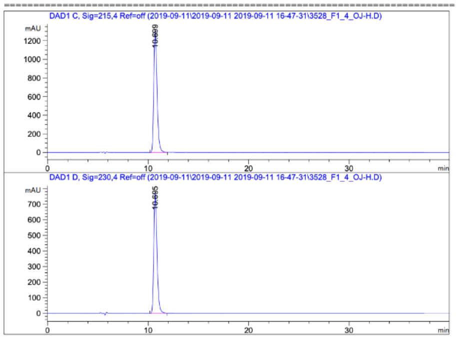

Signal: DAD1 C, Sig=215,4 Ref=off

RetTime(min)	Area,%	Symm.	Resolution	Selectivity
10.6992	100.00	0.726		

Signal: DAD1 D, Sig=230,4 Ref=off

RetTime(min)	Area,%	Symm.	Resolution	Selectivity
10.6952	100.00	0.707		

Compound 13

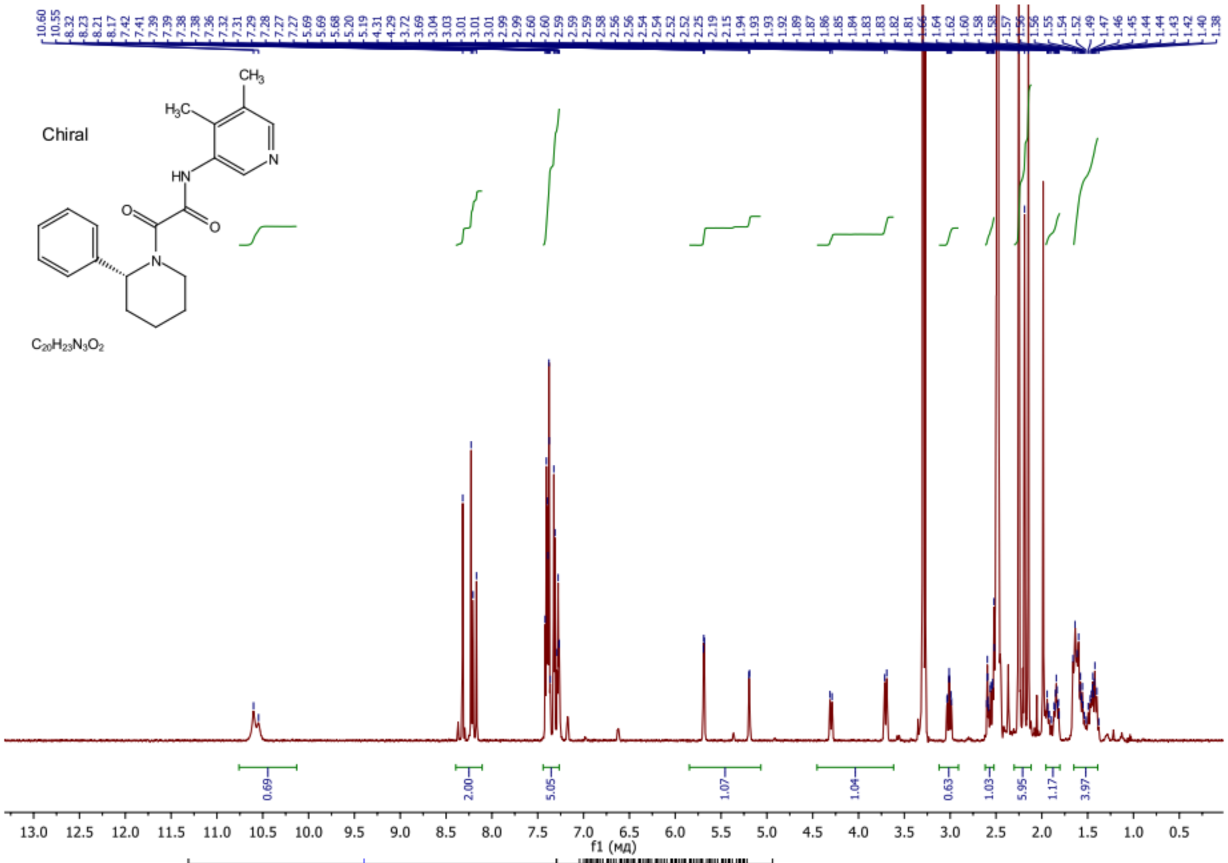
Chiral
$C_{20}H_{23}N_3O_2$
f1 (мд)

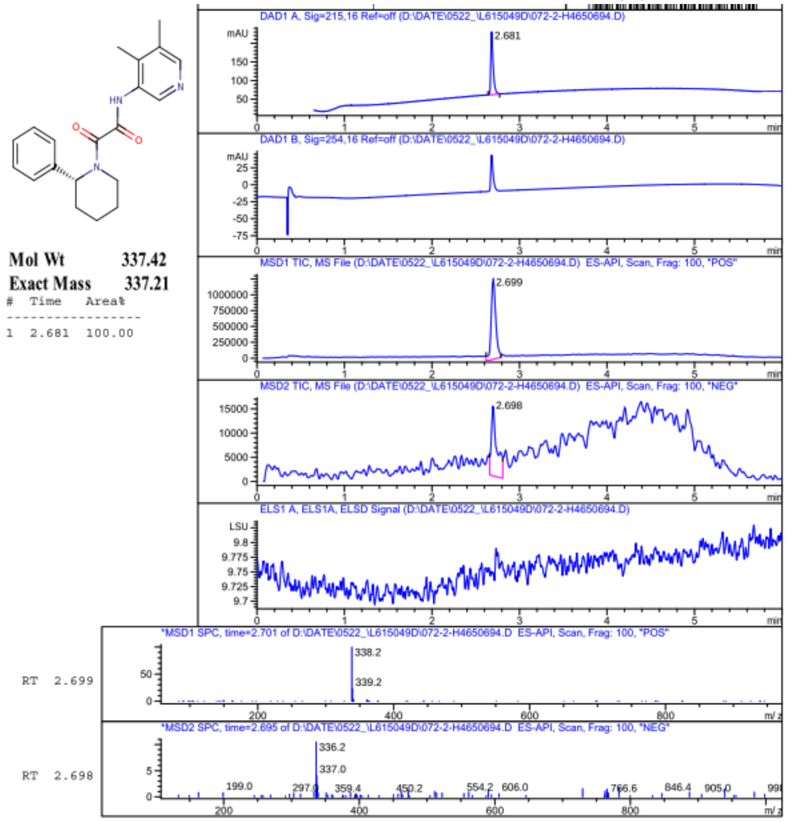
Mol Wt 337.42
Exact Mass 337.21
Time Area%
1 2.681 100.00
RT 2.699
RT 2.698

Compound 14

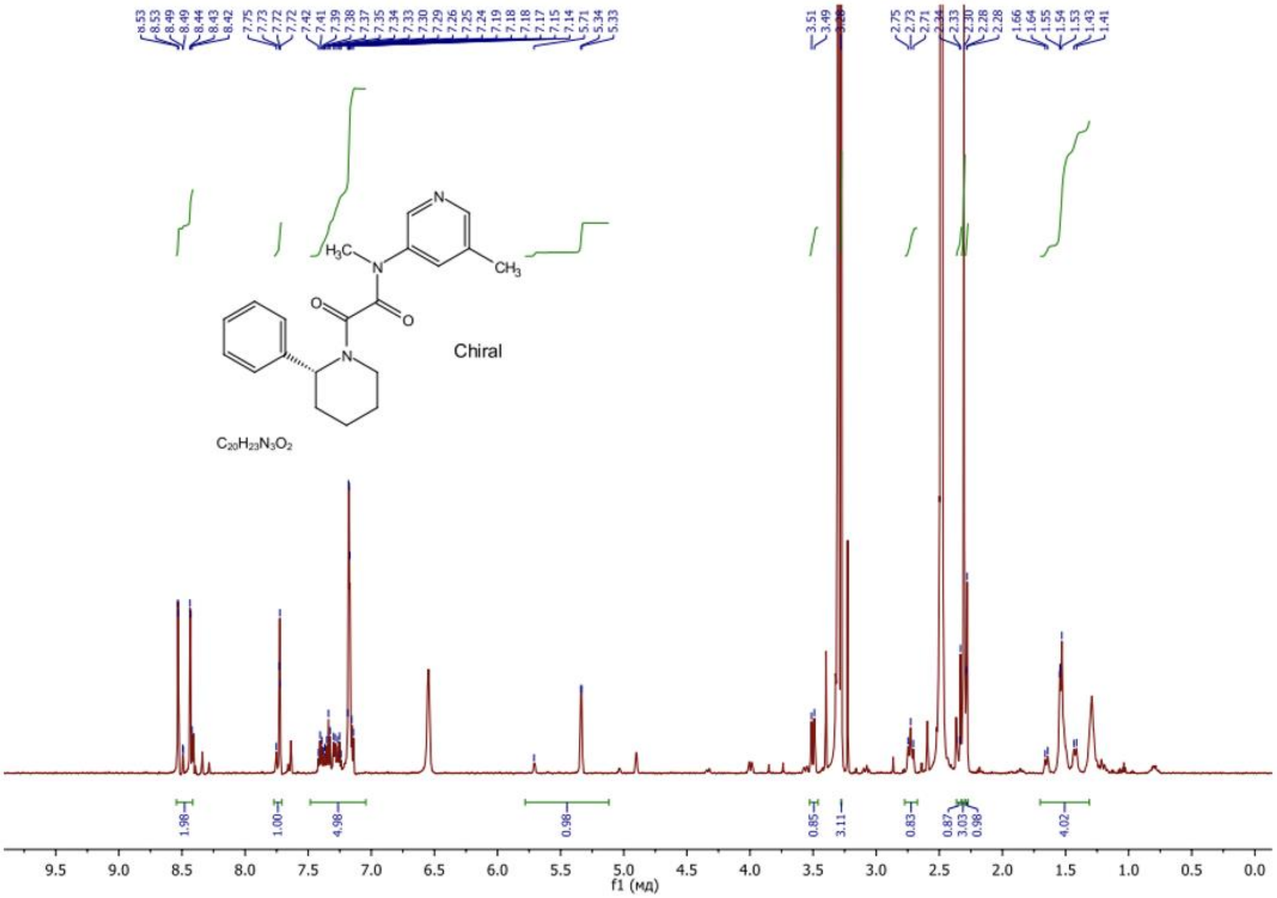
Chiral
$C_{20}H_{23}N_3O_2$
f1 (мд)

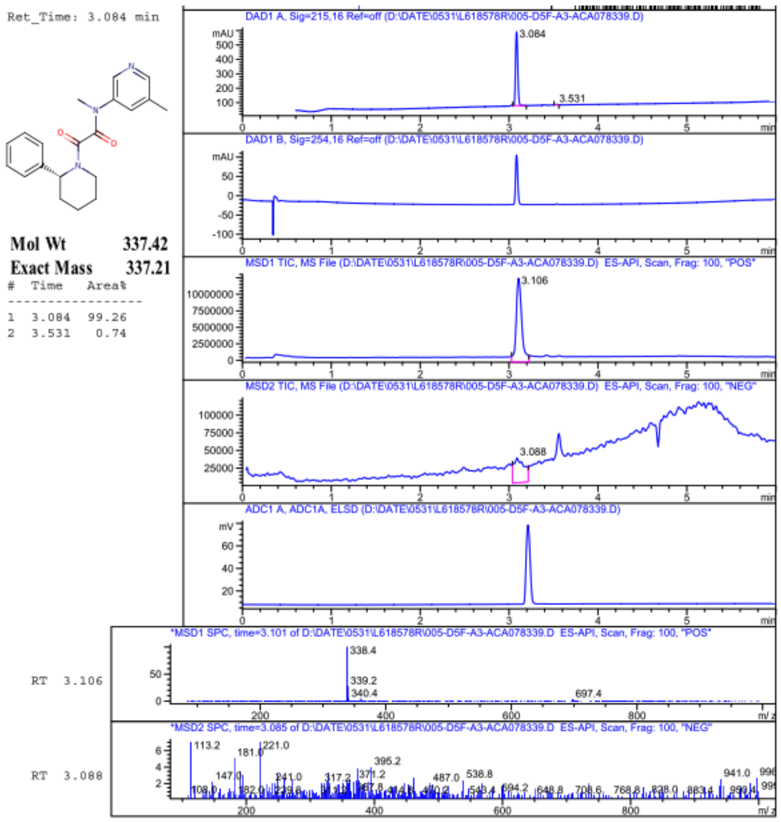
Ret_Time: 3.084 min
Mol Wt 337.42
Exact Mass 337.21
Time Area%
1 3.084 99.26
2 3.531 0.74
DAD1 A, Sig=215,16 Ref=off (D:\DATE\0531\L618578R\005-D5F-A3-ACA078339.D)
DAD1 B, Sig=254,16 Ref=off (D:\DATE\0531\L618578R\005-D5F-A3-ACA078339.D)
MSD1 TIC, MS File (D:\DATE\0531\L618578R\005-D5F-A3-ACA078339.D) ES-API, Scan, Frag: 100, "POS"
MSD2 TIC, MS File (D:\DATE\0531\L618578R\005-D5F-A3-ACA078339.D) ES-API, Scan, Frag: 100, "NEG"
ADC1 A, ADC1A, ELSD (D:\DATE\0531\L618578R\005-D5F-A3-ACA078339.D)
*MSD1 SPC, time=3.101 of D:\DATE\0531\L618578R\005-D5F-A3-ACA078339.D ES-API, Scan, Frag: 100, "POS"
RT 3.106
*MSD2 SPC, time=3.085 of D:\DATE\0531\L618578R\005-D5F-A3-ACA078339.D ES-API, Scan, Frag: 100, "NEG"
RT 3.088

Compound 15

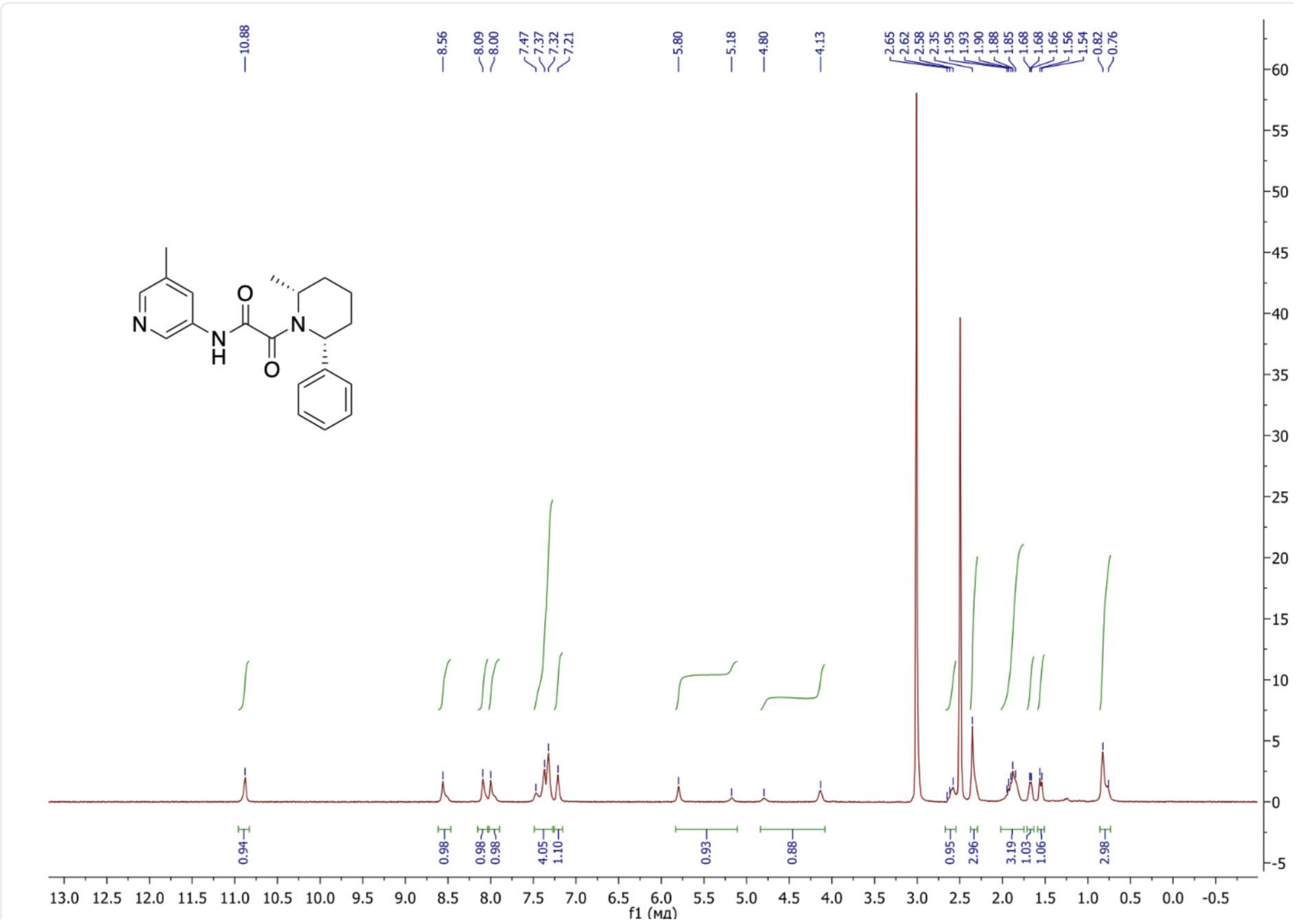

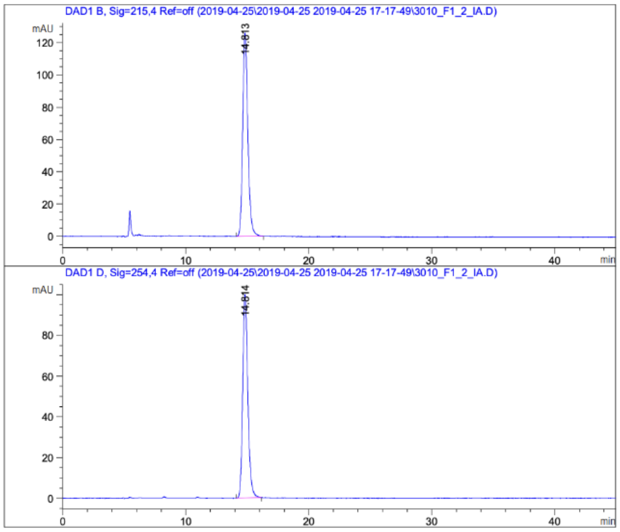

#	Time	Area%
1	4.299	100.00

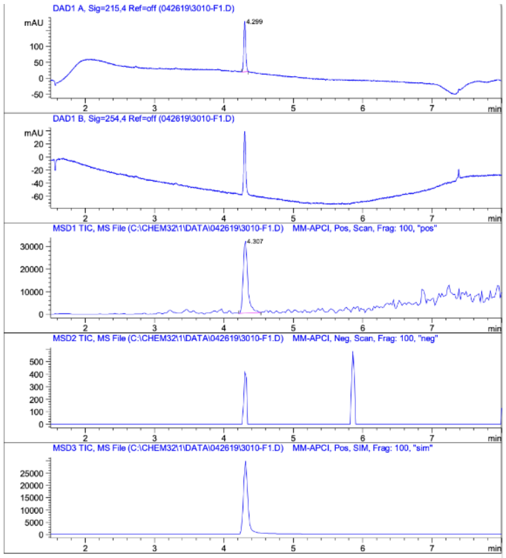

Signal: DAD1 B, Sig=215,4 Ref=off

RetTime (min)	Area,%	Symm.	Resolution	Selectivity
14.8132	100.00	0.768		

RT 4.307

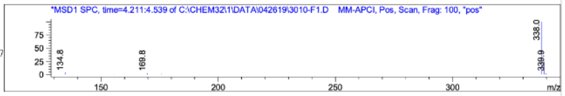

Compound 16

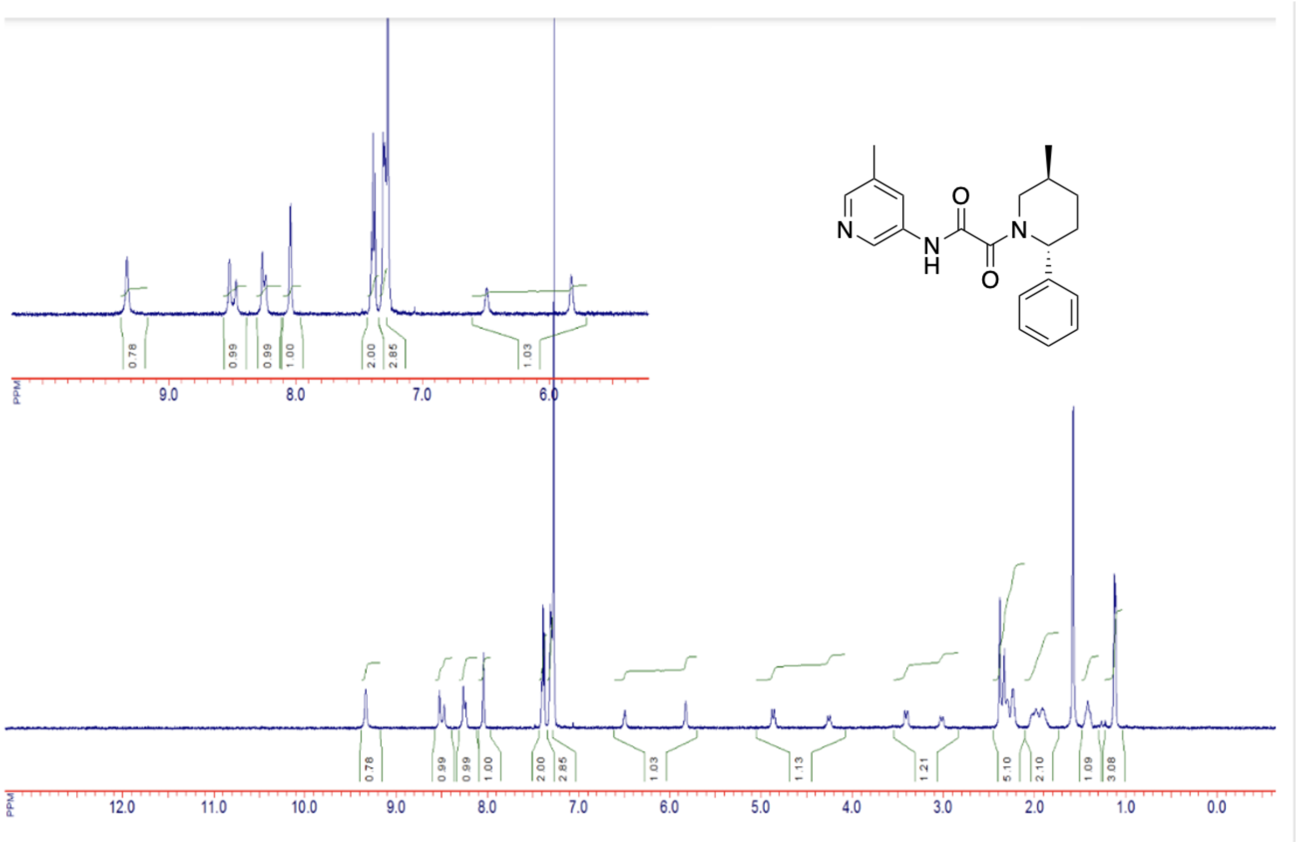


```
#  Time   Area%
-----------------
1  4.740  100.00
```

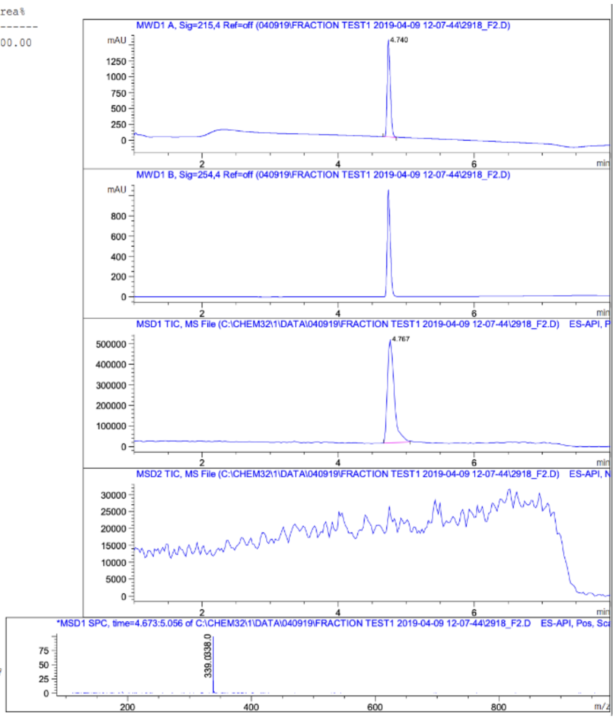

RT 4.767

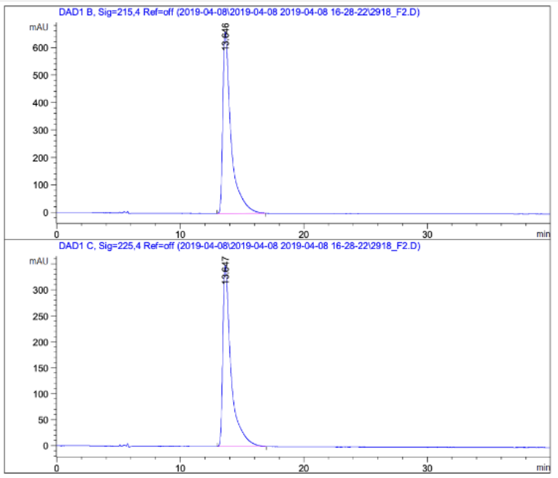

Signal: DAD1 B, Sig=215,4 Ref=off

RetTime (min)	Area,%	Symm.	Resolution	Selectivity
13.6462	100.00	0.391		

Signal: DAD1 C, Sig=225,4 Ref=off

RetTime (min)	Area,%	Symm.	Resolution	Selectivity
13.6472	100.00	0.393		

Compound 17

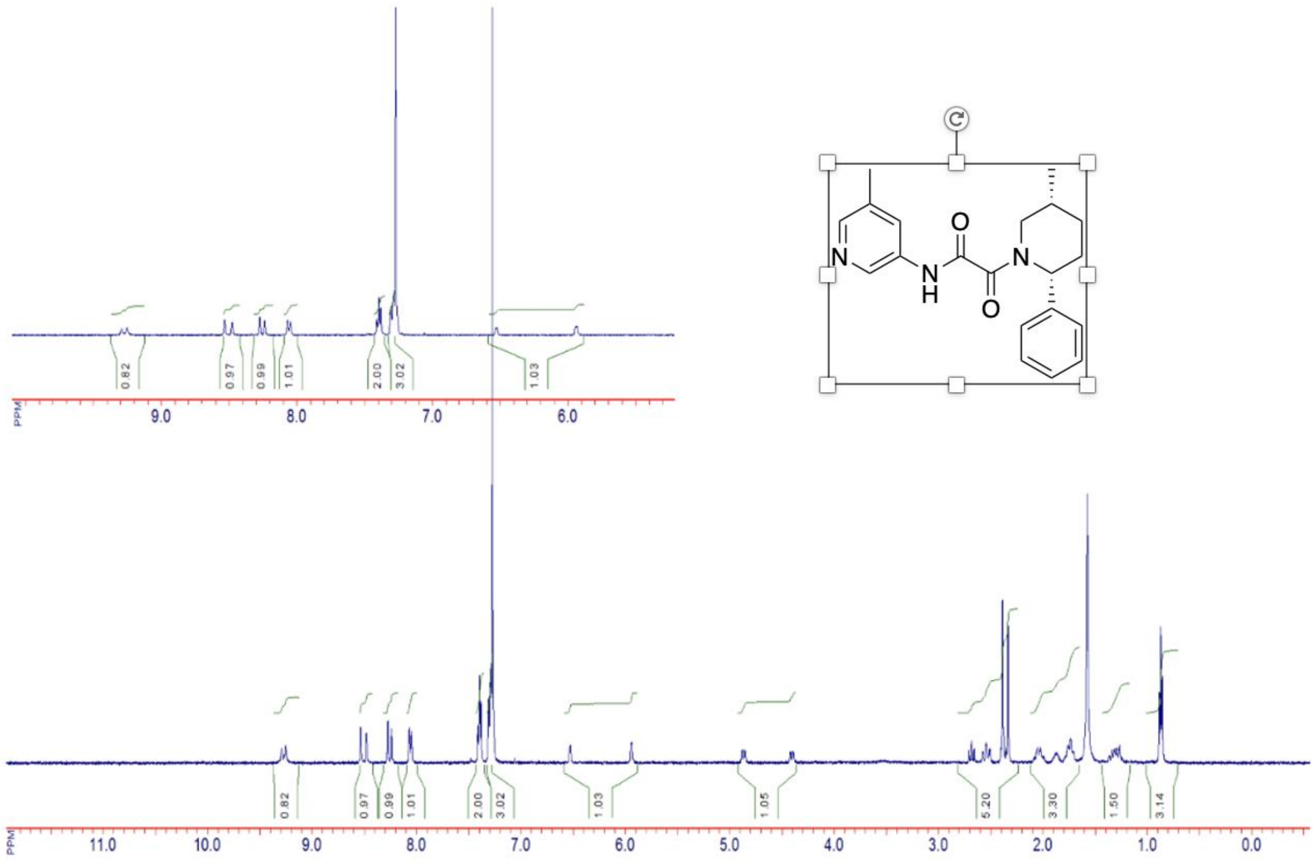

```
#  Time   Area%
-----------------
1  4.808  100.00
```

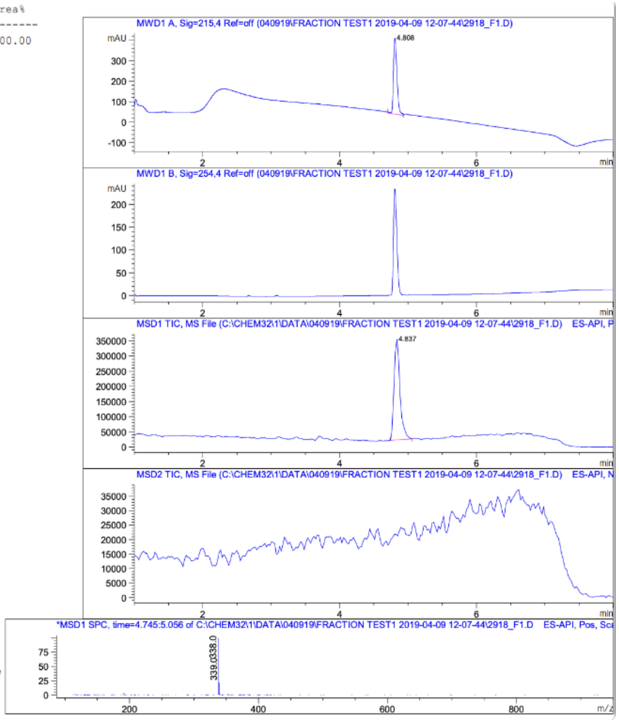

RT 4.837

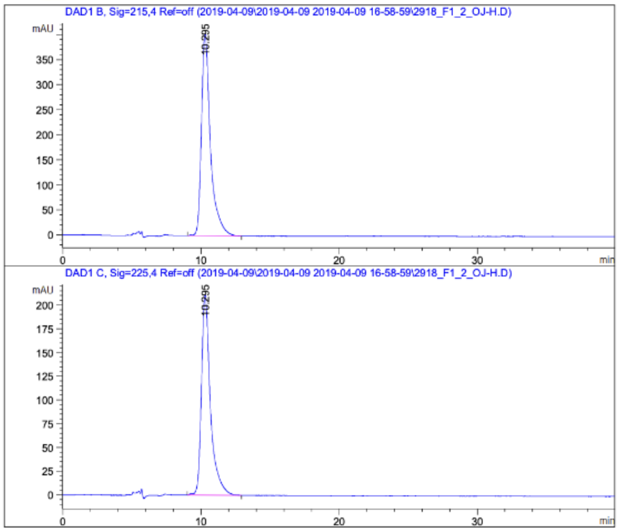


```
Signal:  DAD1 B, Sig=215,4 Ref=off
=====================================================================
RetTime(min)  Area,%    Symm.   Resolution Selectivity
|------------|--------|----------|-----------|-----------|
10.2952       100.00    0.621
=====================================================================

Signal:  DAD1 C, Sig=225,4 Ref=off
=====================================================================
RetTime(min)  Area,%    Symm.   Resolution Selectivity
|------------|--------|----------|-----------|-----------|
10.2952       100.00    0.625
=====================================================================
```

Compound 18

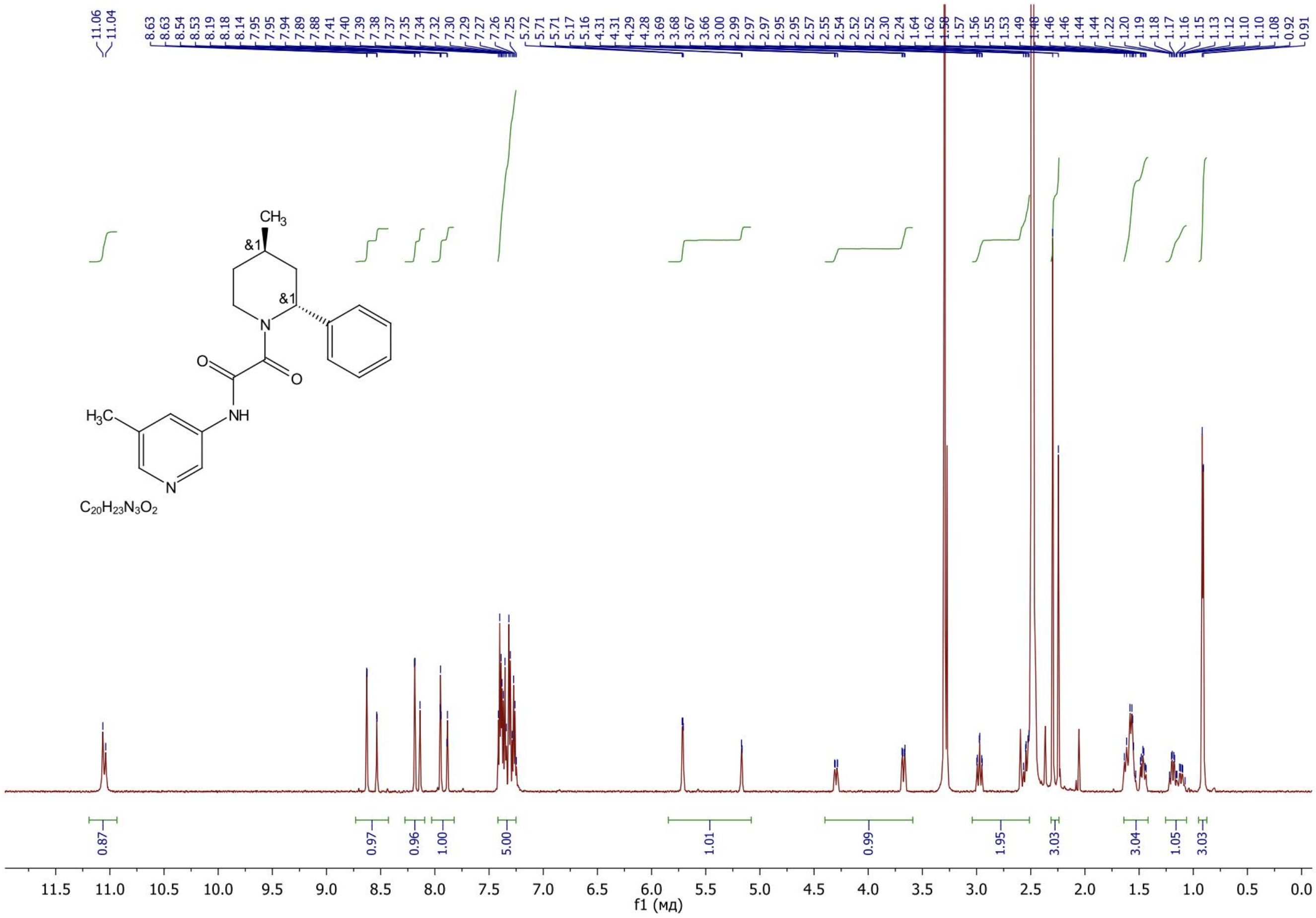

CH3
&1
&1
N
O
O
H3C
NH
N
C20H23N3O2
11.5 11.0 10.5 10.0 9.5 9.0 8.5 8.0 7.5 7.0 6.5 6.0 5.5 5.0 4.5 4.0 3.5 3.0 2.5 2.0 1.5 1.0 0.5 0.0
f1 (мд)
0.87
0.97
0.96
1.00
5.00
1.01
0.99
1.95
3.03
3.04
1.05
3.03

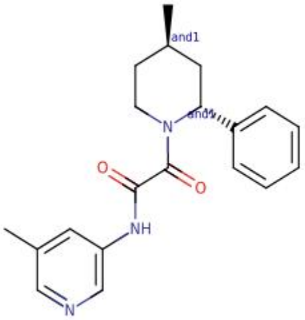

Mol Wt 337.42
Exact Mass 337.21

#	Time	Area%
1	1.684	1.54
2	2.091	98.46

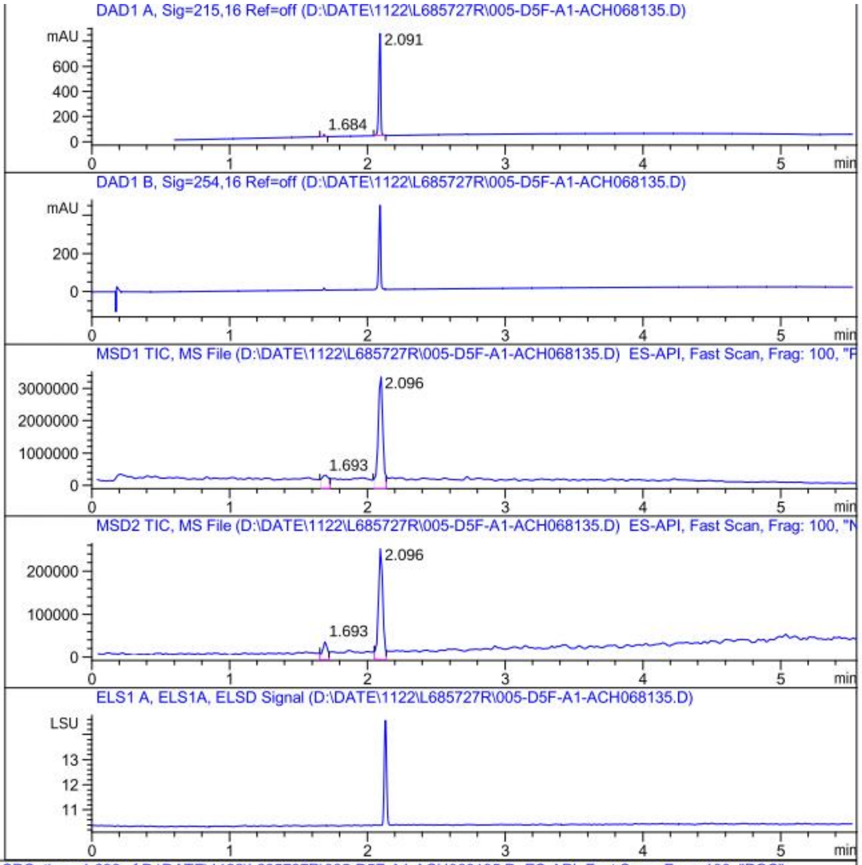

RT 1.693
RT 2.096
RT 1.693
RT 2.096

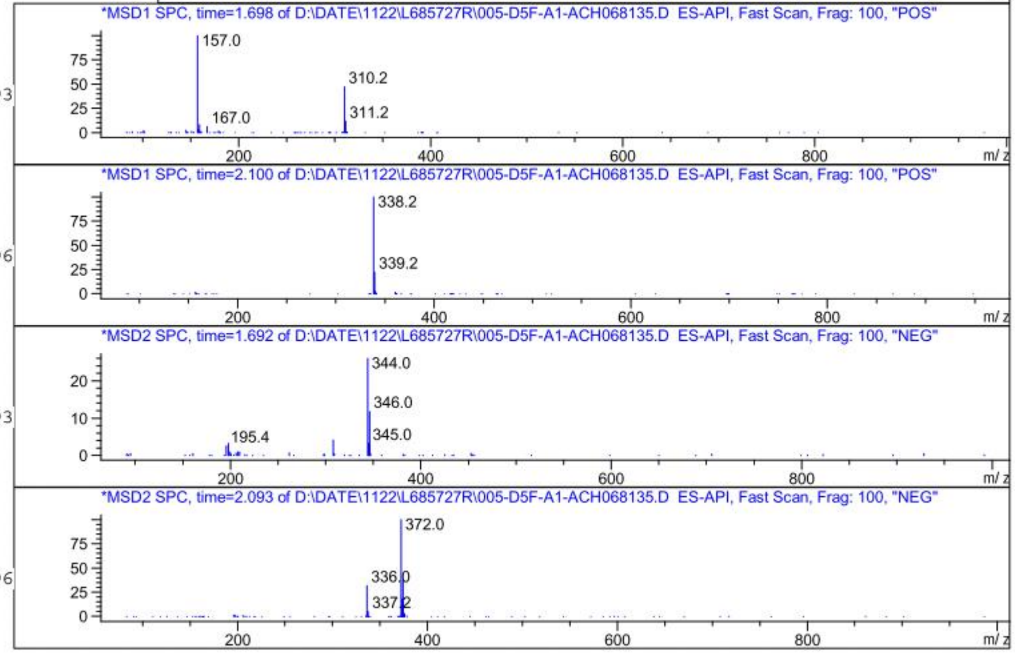

Intermediate 18a

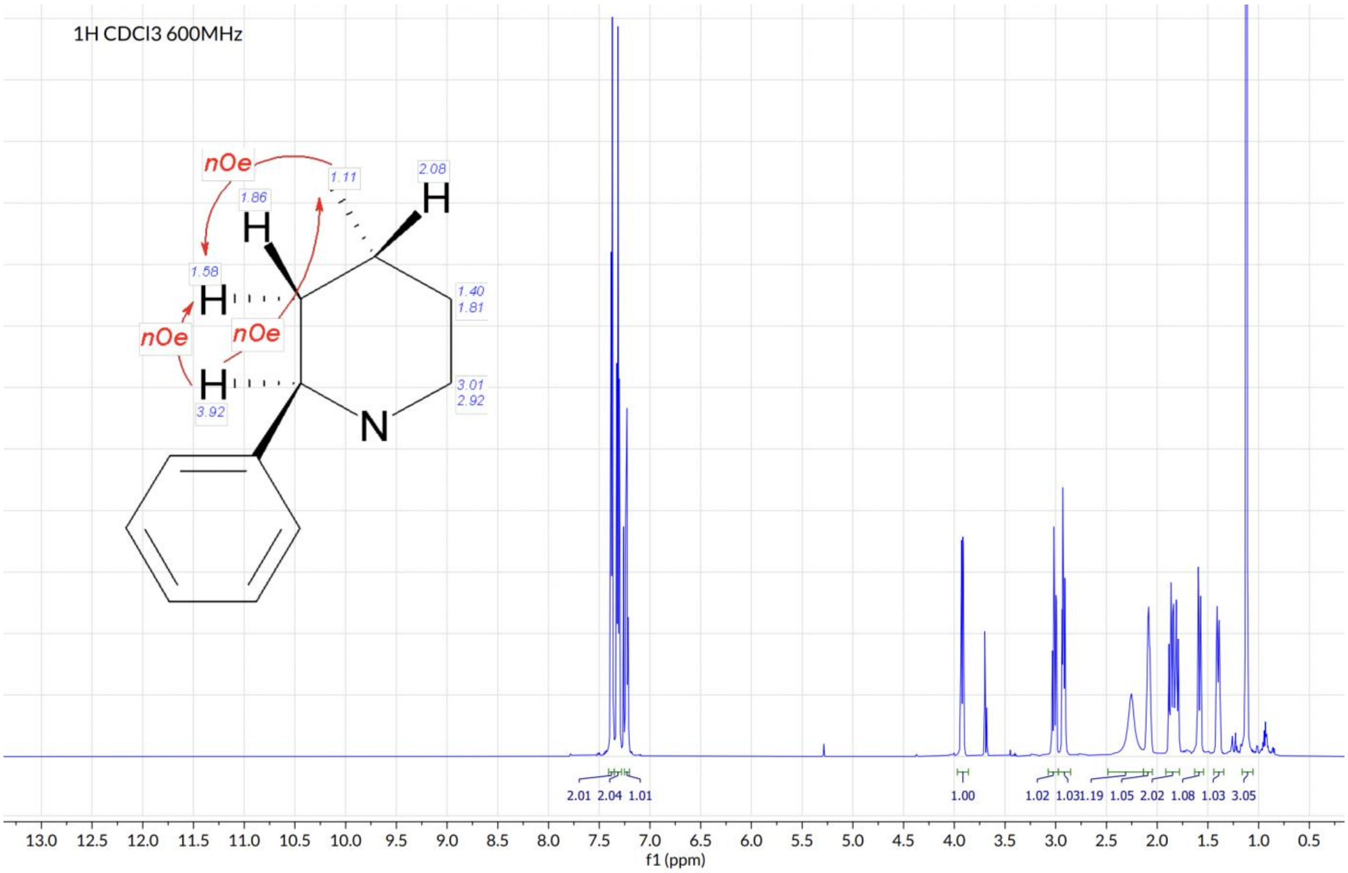
1H CDCl3 600MHz
nOe
nOe
nOe
1.11
2.08
1.86
1.58
1.40
1.81
3.01
2.92
3.92
N
2.01 2.04 1.01
1.00
1.02 1.03 1.19 1.05 2.02 1.08 1.03 3.05
13.0 12.5 12.0 11.5 11.0 10.5 10.0 9.5 9.0 8.5 8.0 7.5 7.0 6.5 6.0 5.5 5.0 4.5 4.0 3.5 3.0 2.5 2.0 1.5 1.0 0.5
f1 (ppm)

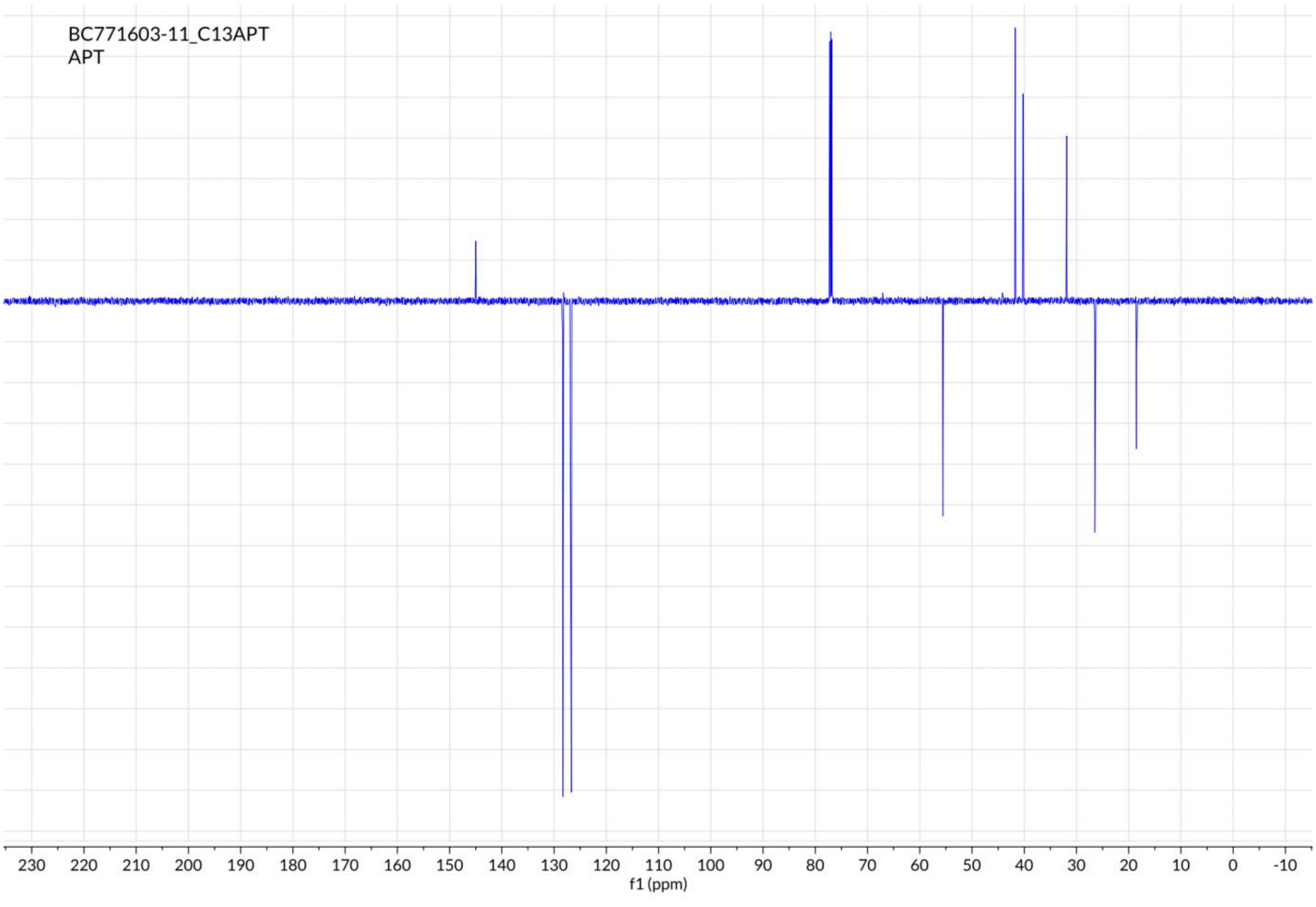
BC771603-11_C13APT
APT
230 220 210 200 190 180 170 160 150 140 130 120 110 100 90 80 70 60 50 40 30 20 10 0 -10
f1 (ppm)

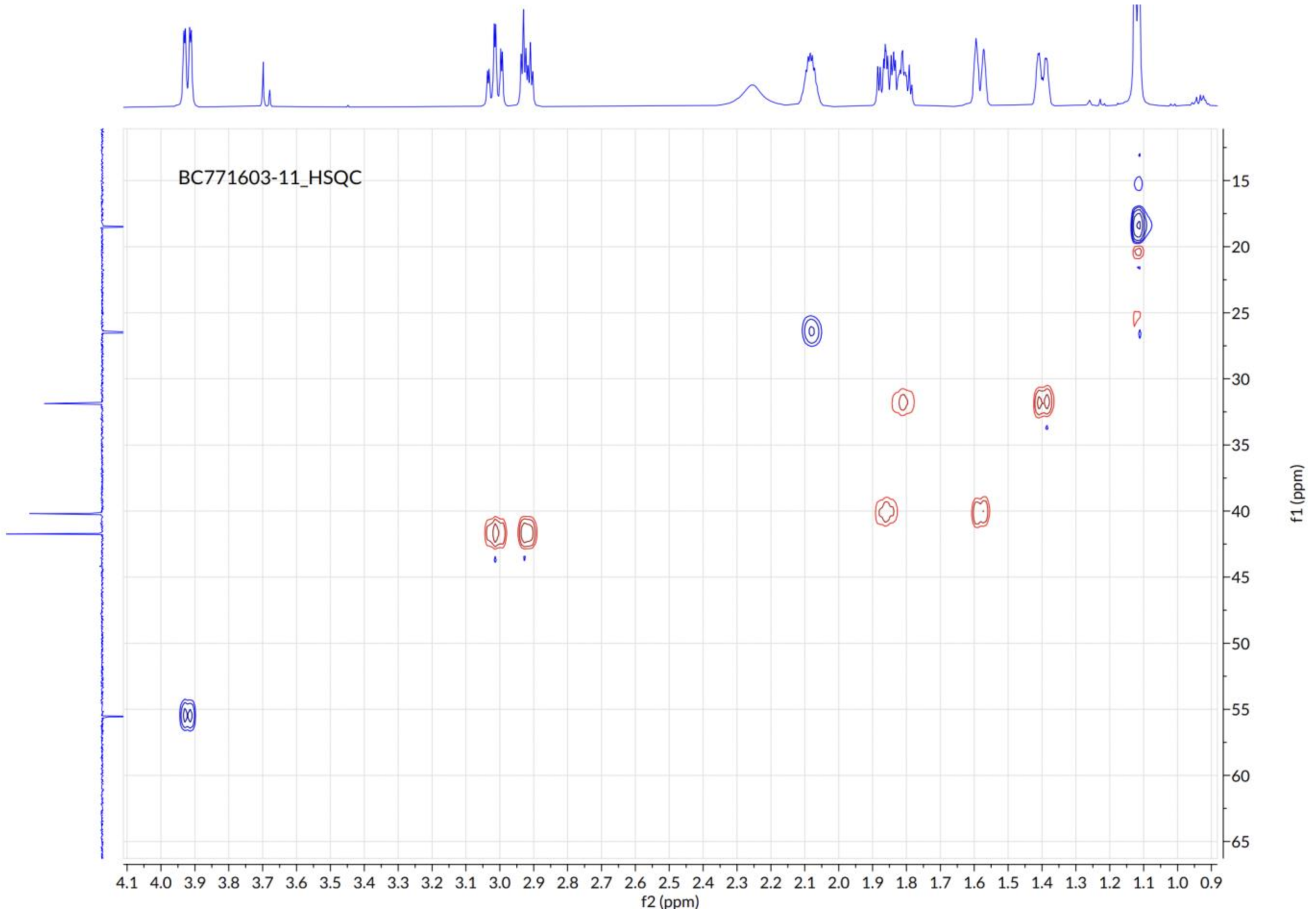
BC771603-11_HSQC
f1 (ppm)
f2 (ppm)

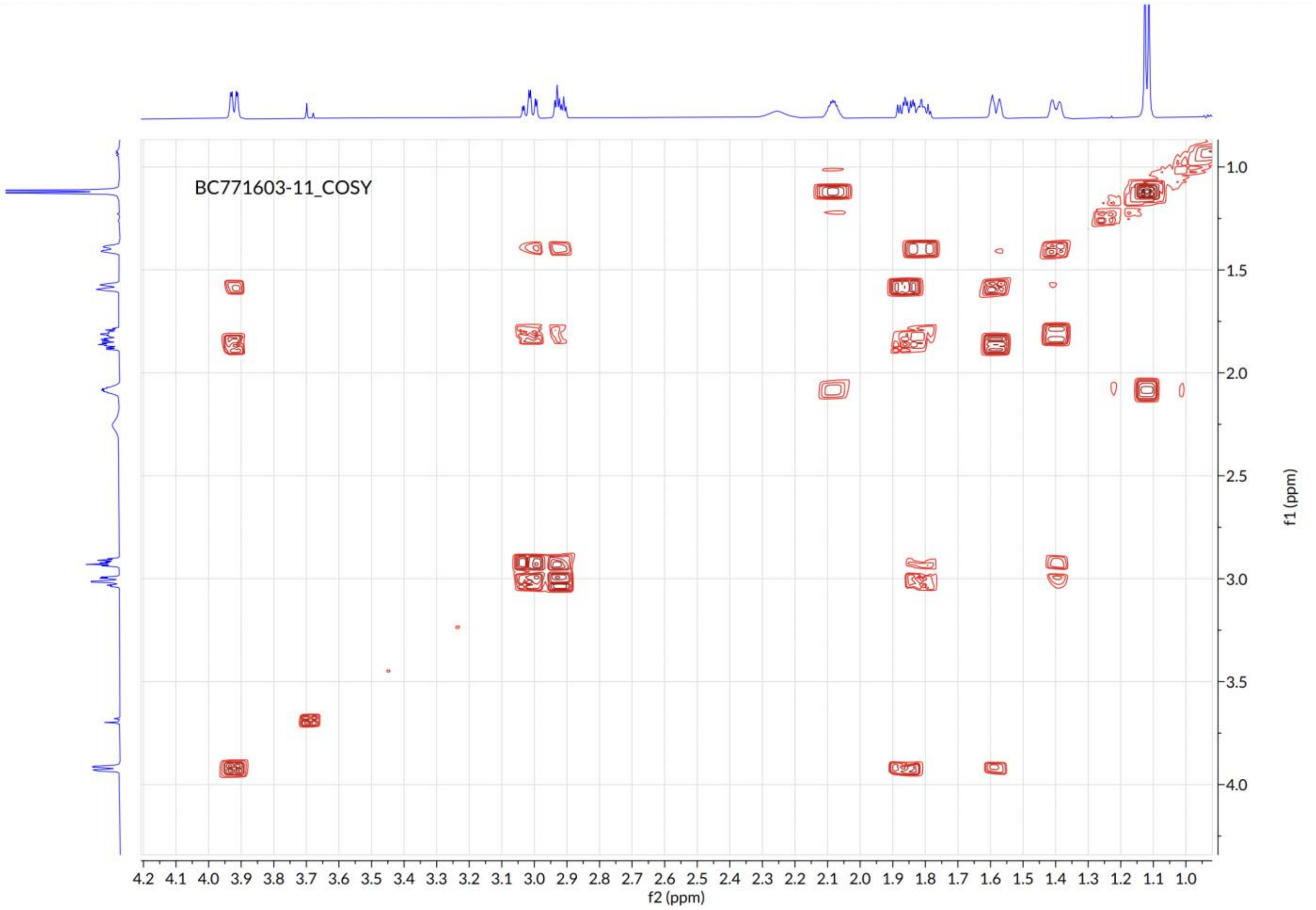
BC771603-11_COSY
f1 (ppm)
f2 (ppm)

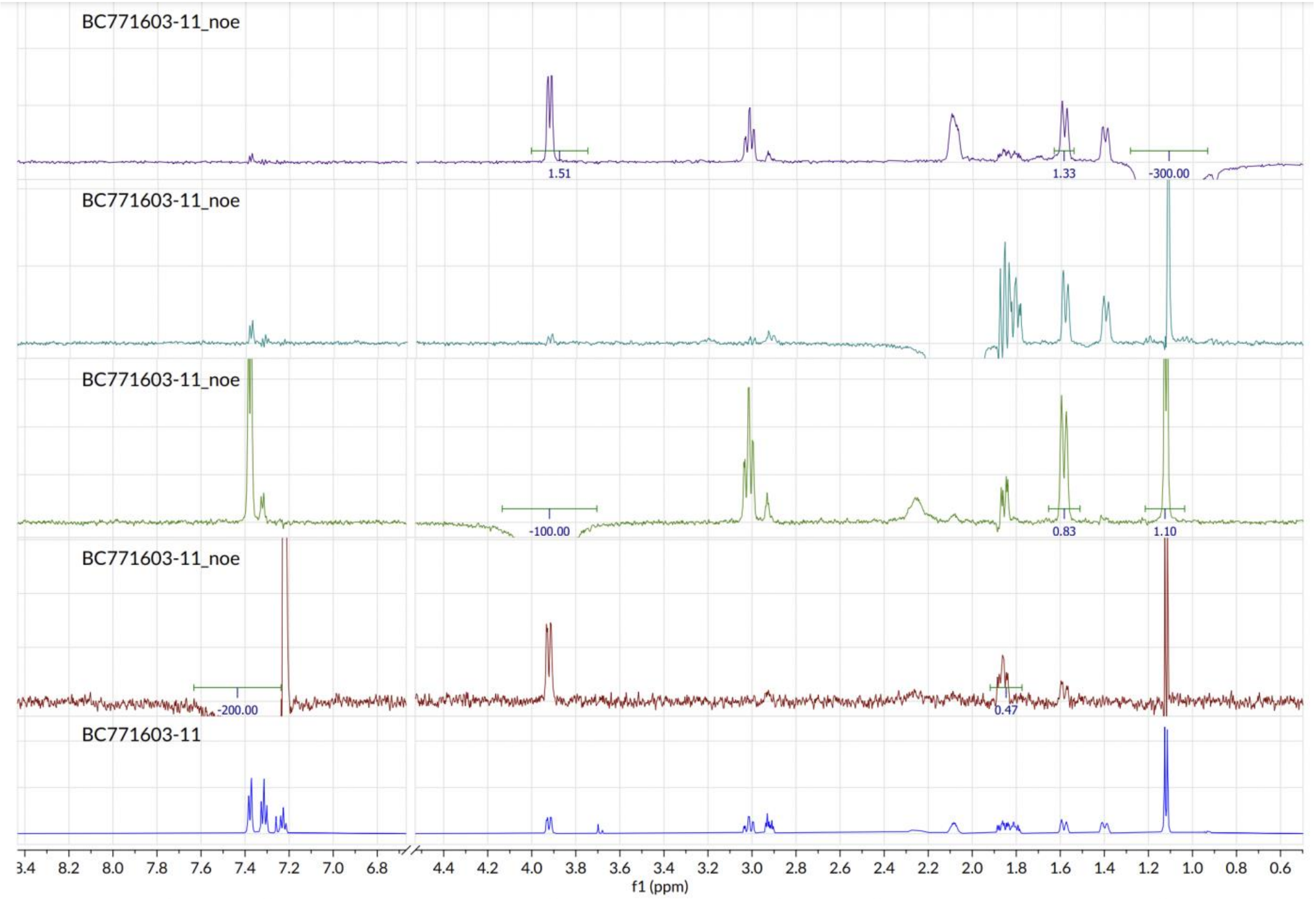
BC771603-11_noe
1.51
1.33
-300.00
BC771603-11_noe
BC771603-11_noe
-100.00
0.83
1.10
BC771603-11_noe
-200.00
0.47
BC771603-11
f1 (ppm)

Compound 19

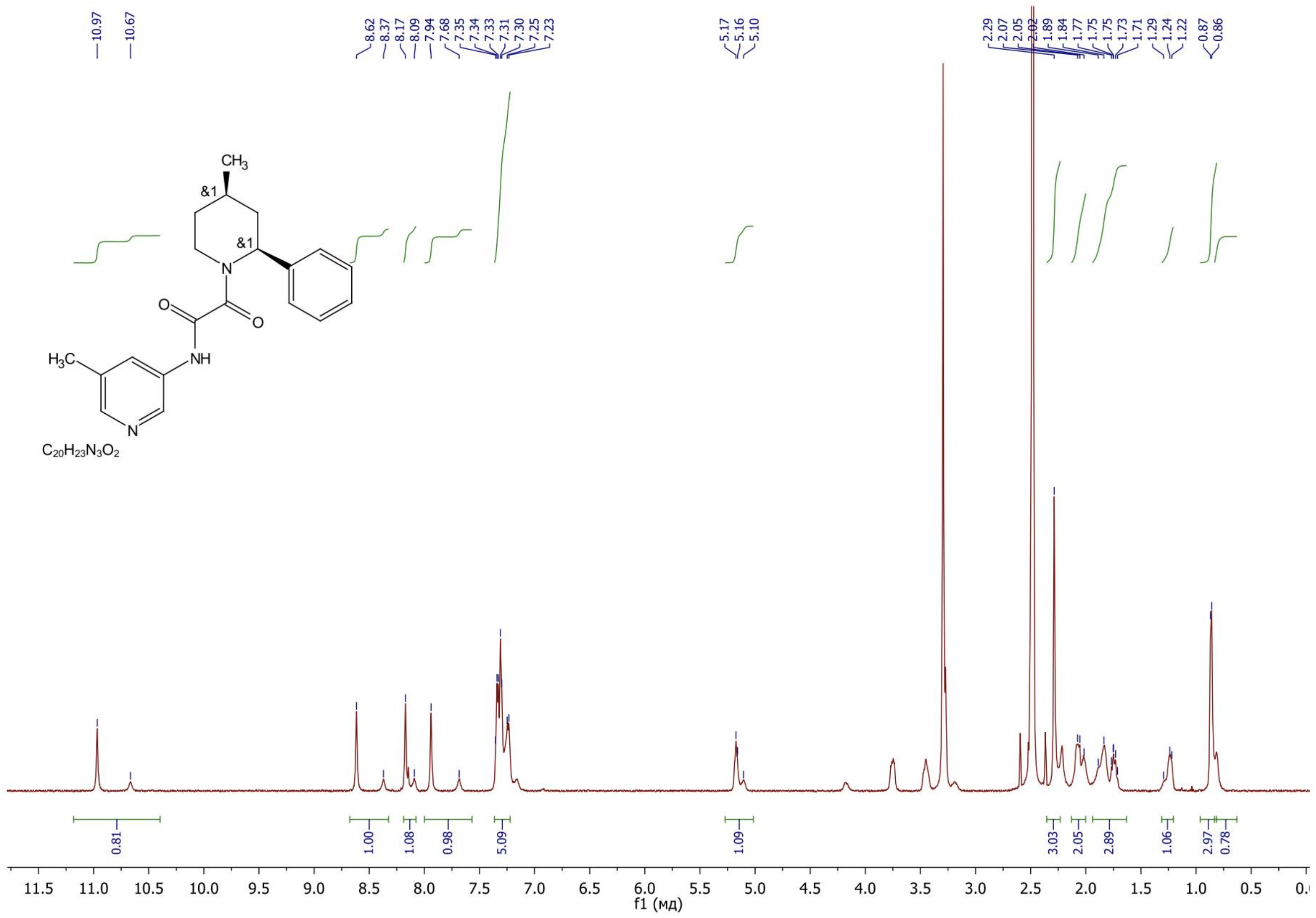
CH3
&1
&1
N
O
O
NH
H3C
N
C20H23N3O2
f1 (мд)

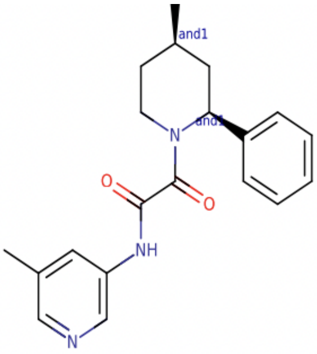

Mol Wt 337.42

Exact Mass 337.21

#	Time	Area%
1	2.914	100.00

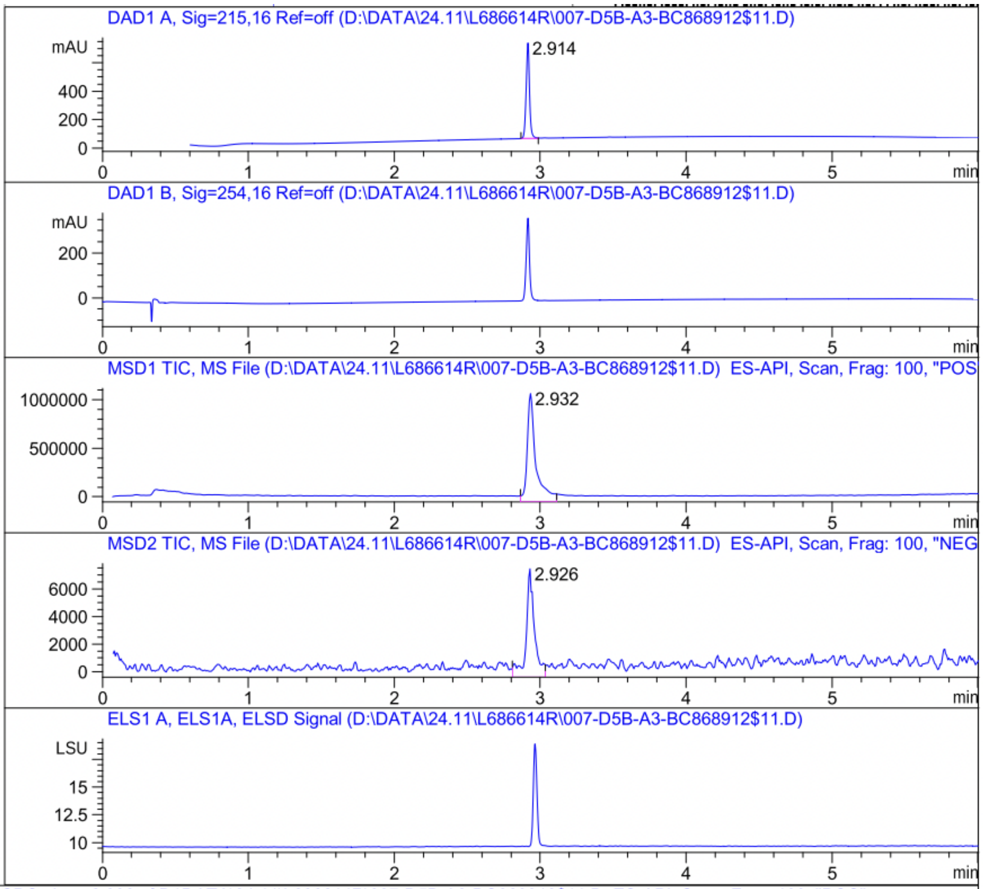

RT 2.932

RT 2.926

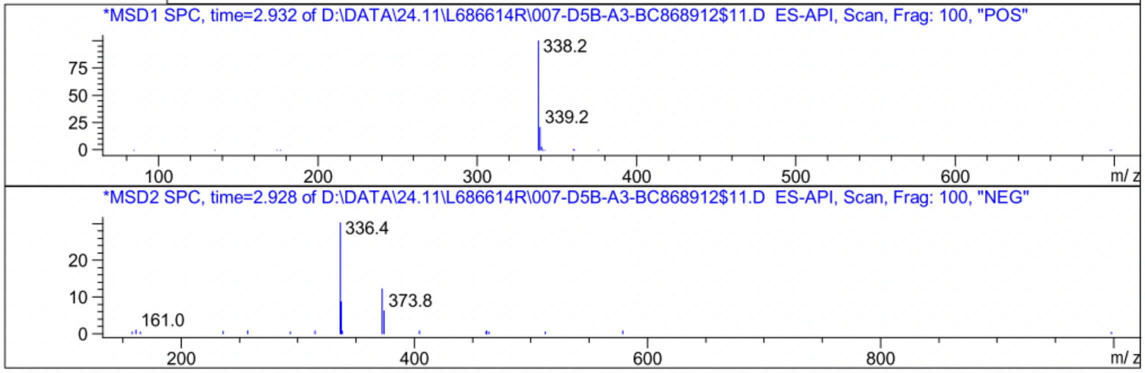

Intermediate 19a

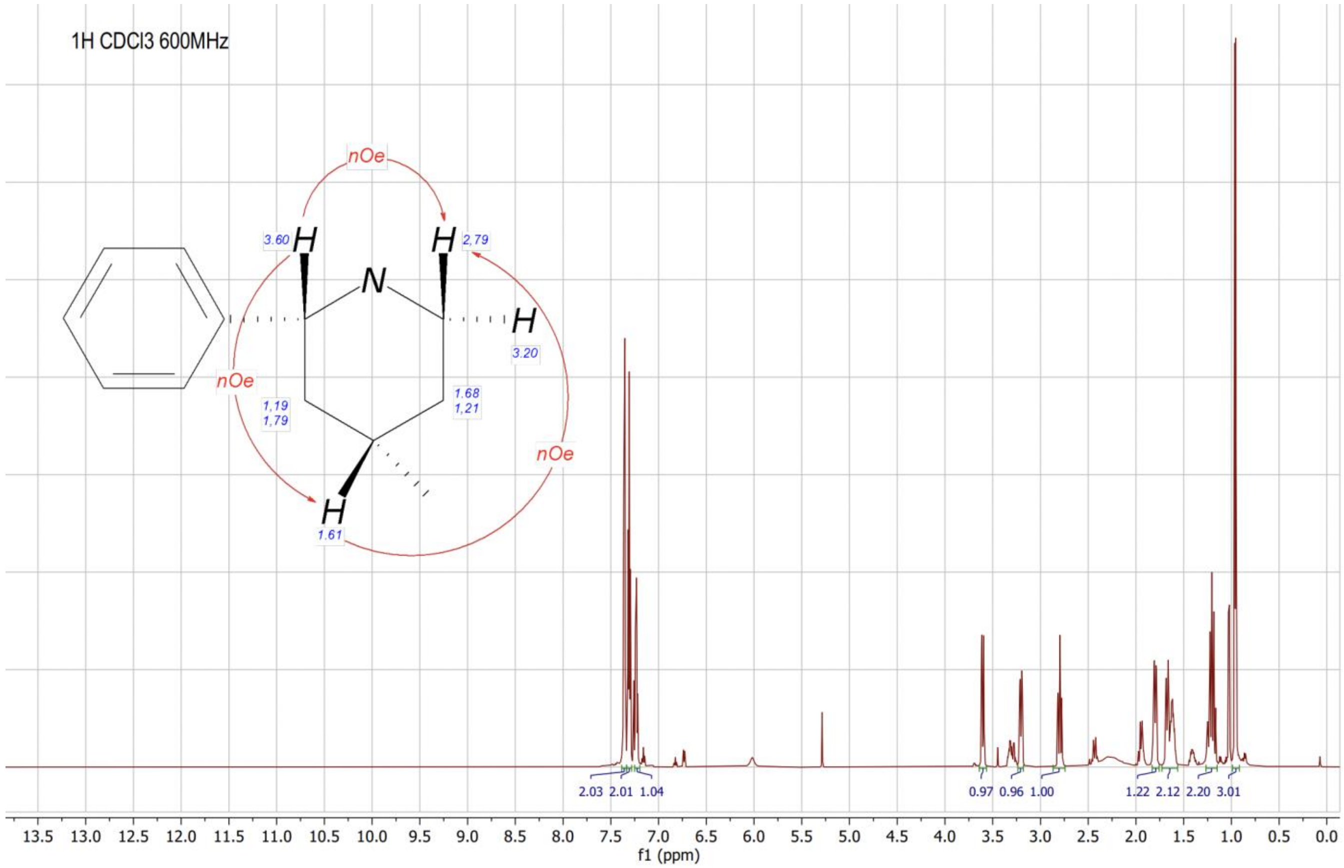
1H CDCl3 600MHz
nOe
3.60
2,79
3.20
nOe
1,19
1,79
1.68
1,21
nOe
1.61
2.03 2.01 1.04
0.97 0.96 1.00
1.22 2.12 2.20 3.01
13.5 13.0 12.5 12.0 11.5 11.0 10.5 10.0 9.5 9.0 8.5 8.0 7.5 7.0 6.5 6.0 5.5 5.0 4.5 4.0 3.5 3.0 2.5 2.0 1.5 1.0 0.5 0.0
f1 (ppm)

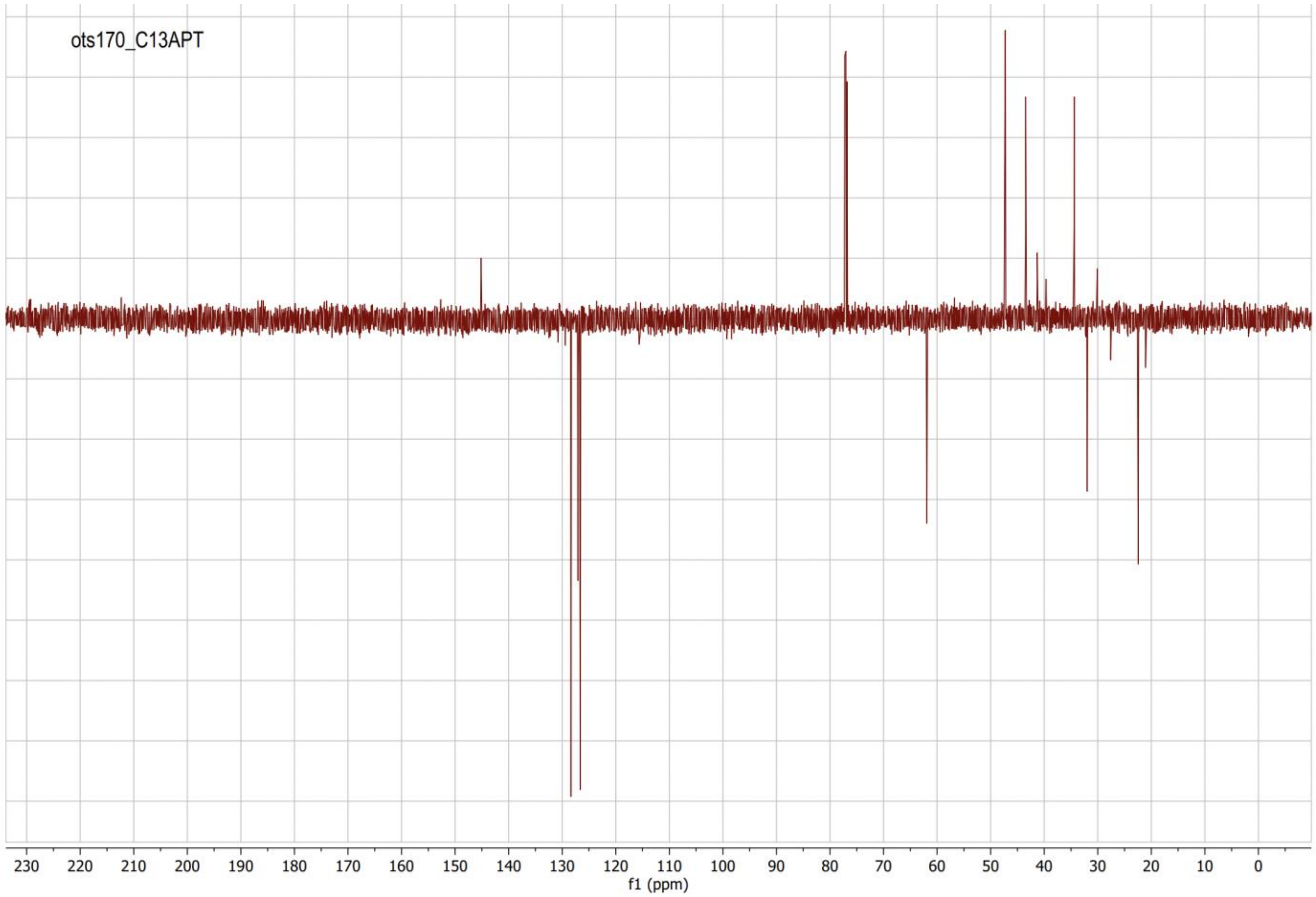
ots170_C13APT
230 220 210 200 190 180 170 160 150 140 130 120 110 100 90 80 70 60 50 40 30 20 10 0
f1 (ppm)

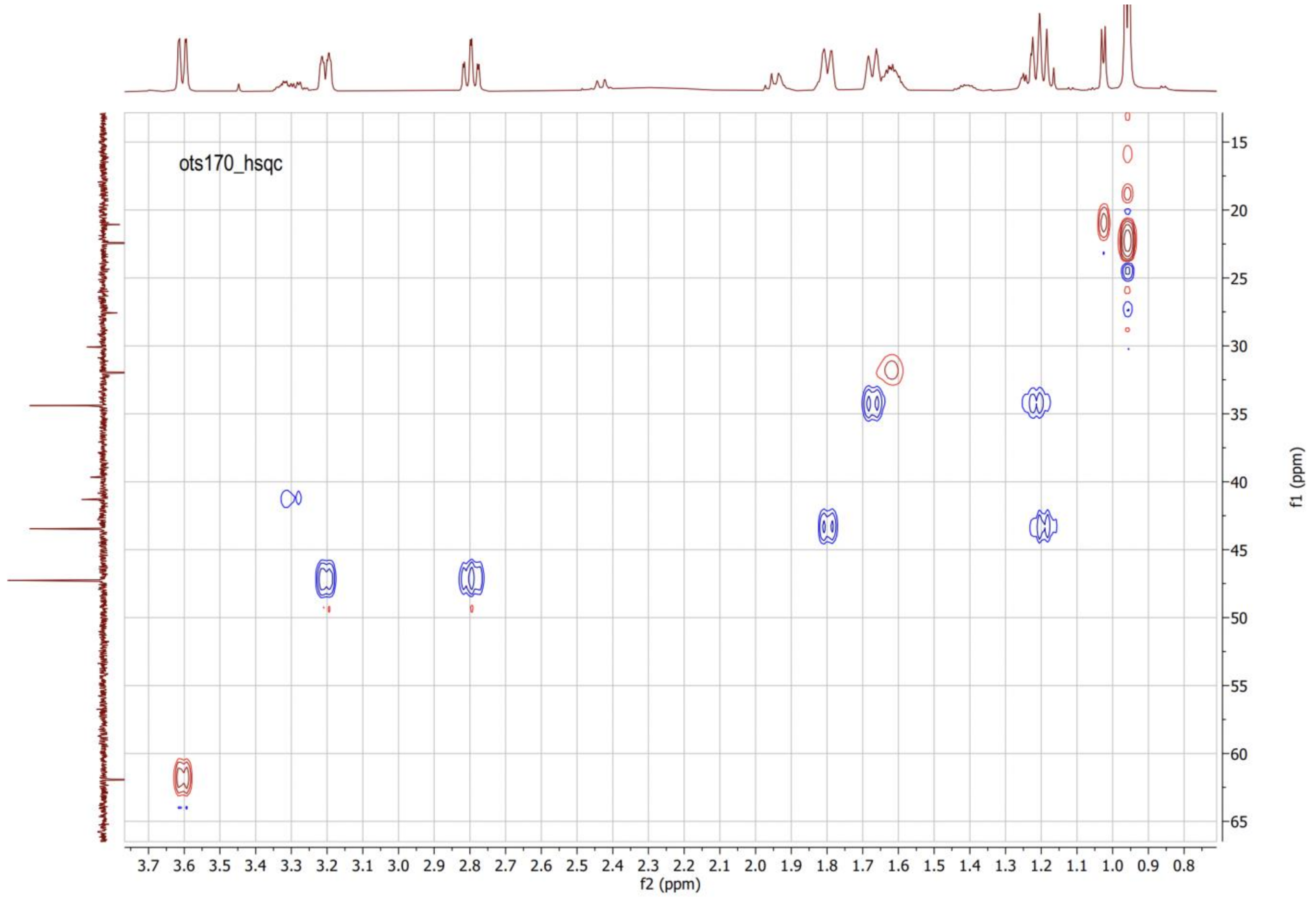
ots170_hsqc
f2 (ppm)
f1 (ppm)

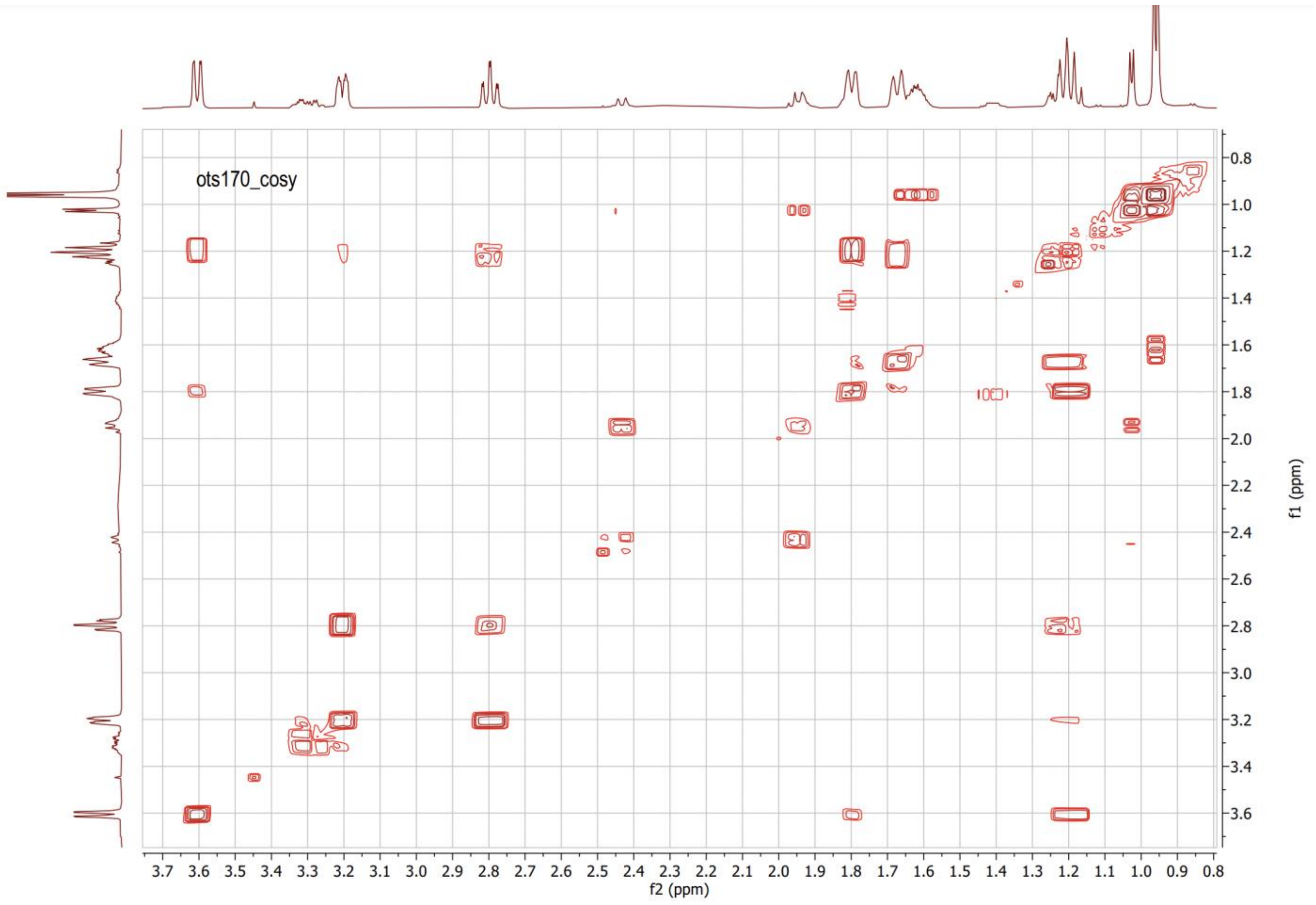
ots170_cosy
f2 (ppm)
f1 (ppm)

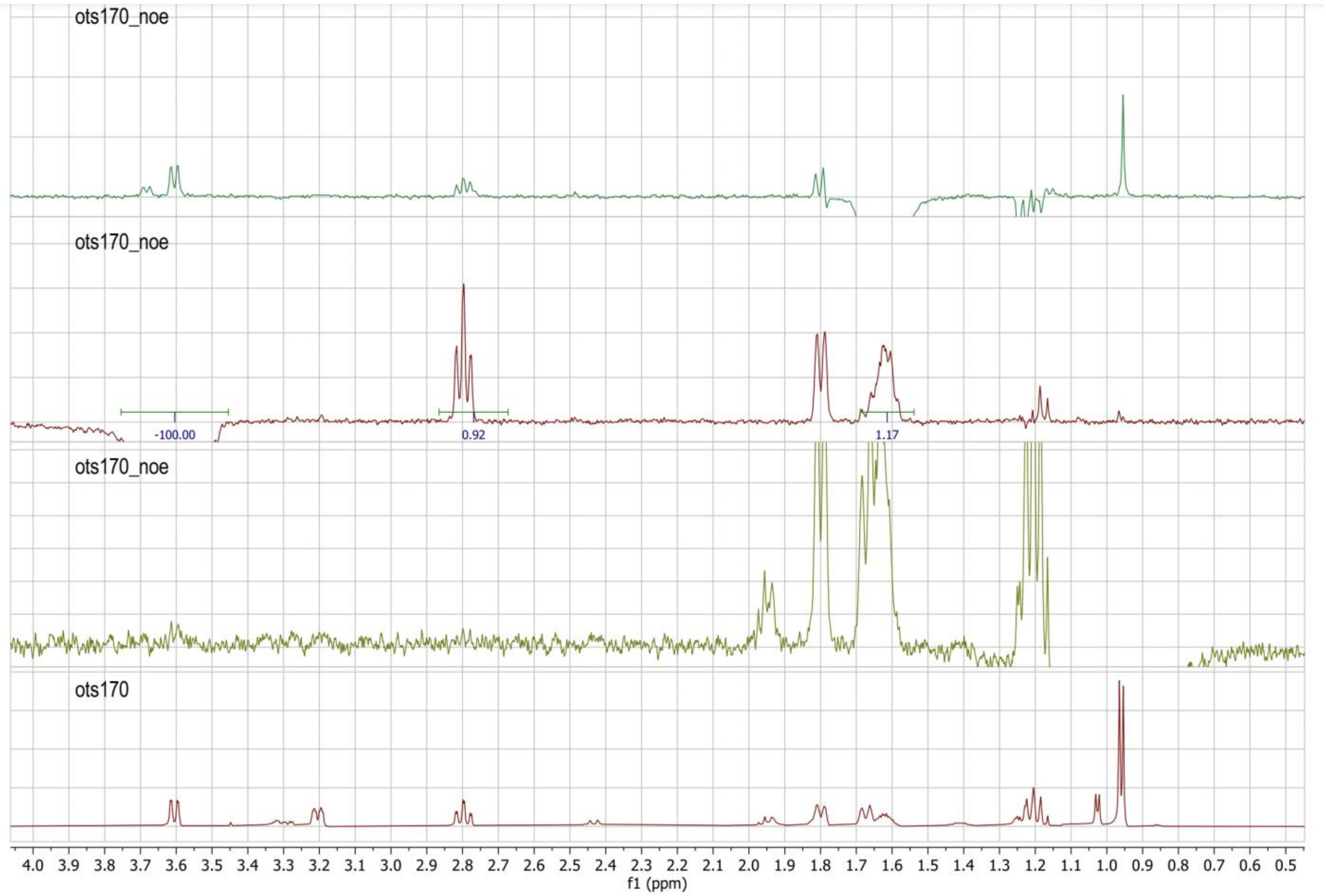
ots170_noe
ots170_noe
-100.00
0.92
1.17
ots170_noe
ots170
4.0 3.9 3.8 3.7 3.6 3.5 3.4 3.3 3.2 3.1 3.0 2.9 2.8 2.7 2.6 2.5 2.4 2.3 2.2 2.1 2.0 1.9 1.8 1.7 1.6 1.5 1.4 1.3 1.2 1.1 1.0 0.9 0.8 0.7 0.6 0.5
f1 (ppm)

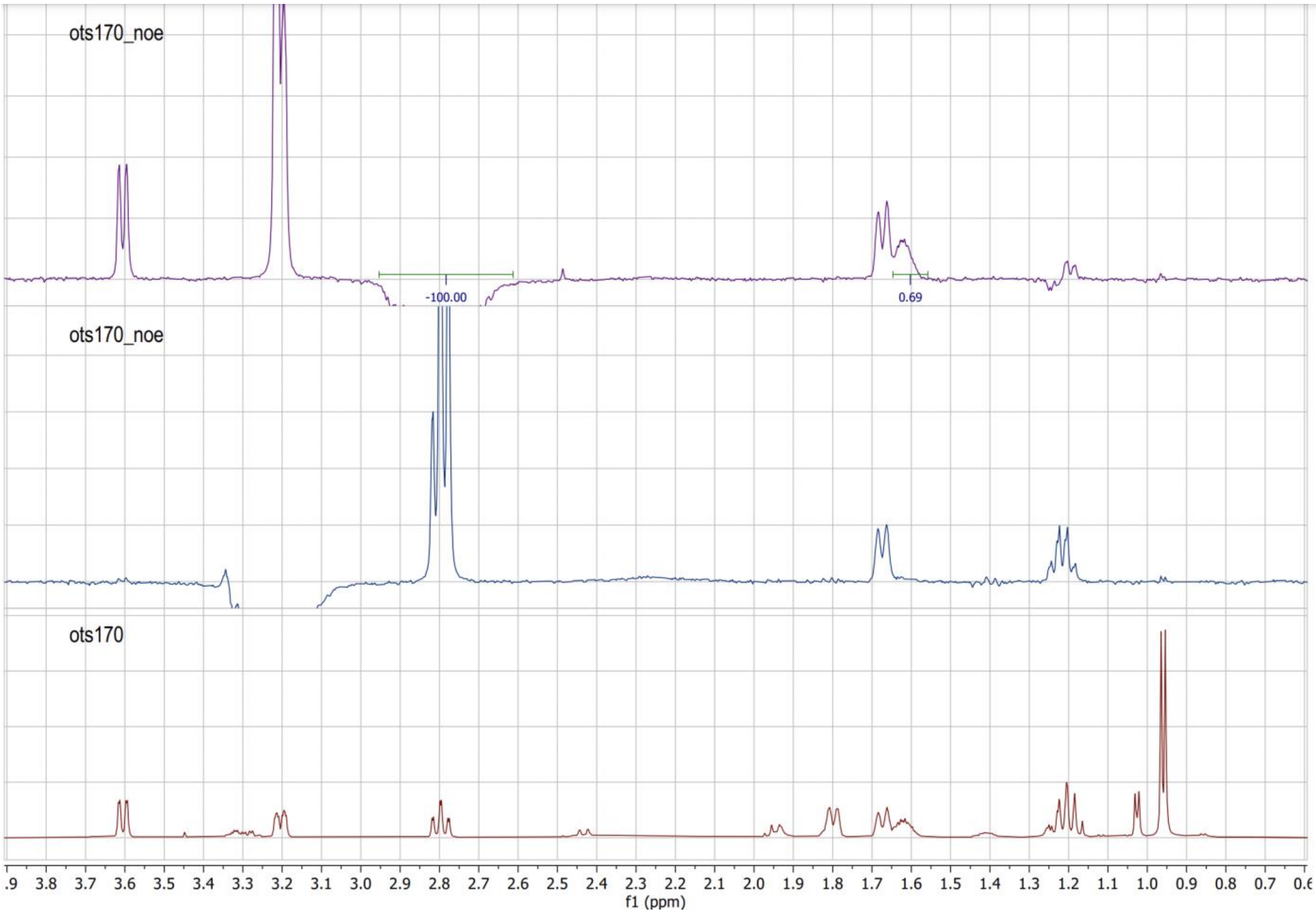

ots170_noe
-100.00
0.69
ots170_noe
ots170
.9 3.8 3.7 3.6 3.5 3.4 3.3 3.2 3.1 3.0 2.9 2.8 2.7 2.6 2.5 2.4 2.3 2.2 2.1 2.0 1.9 1.8 1.7 1.6 1.5 1.4 1.3 1.2 1.1 1.0 0.9 0.8 0.7 0.6
f1 (ppm)

Compound 20

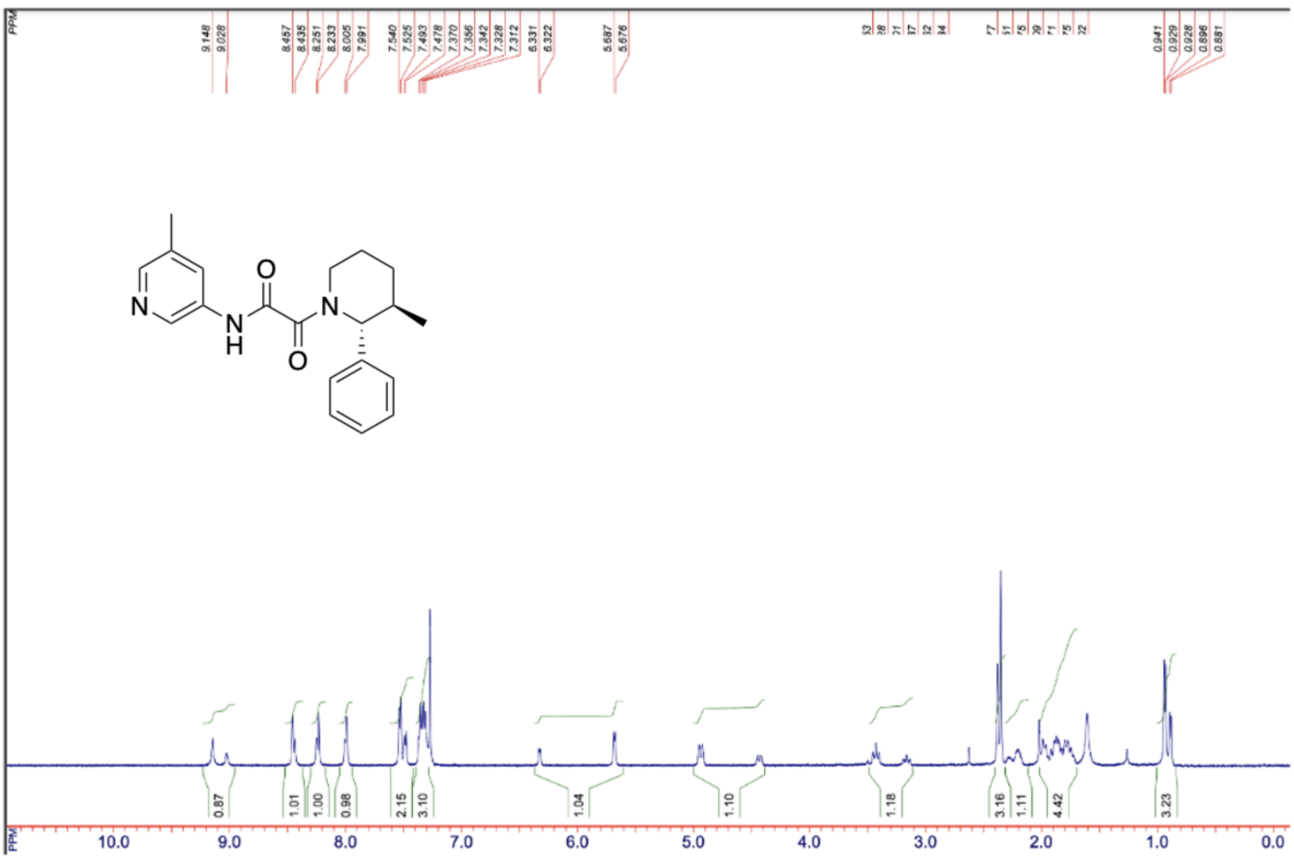

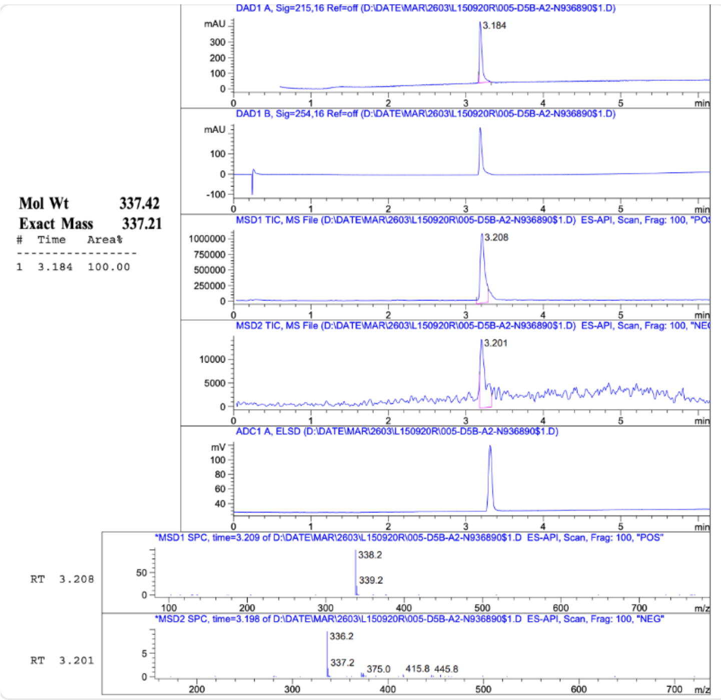
Mol Wt 337.42
Exact Mass 337.21
Time Area%
1 3.184 100.00
RT 3.208
RT 3.201

Compound 21

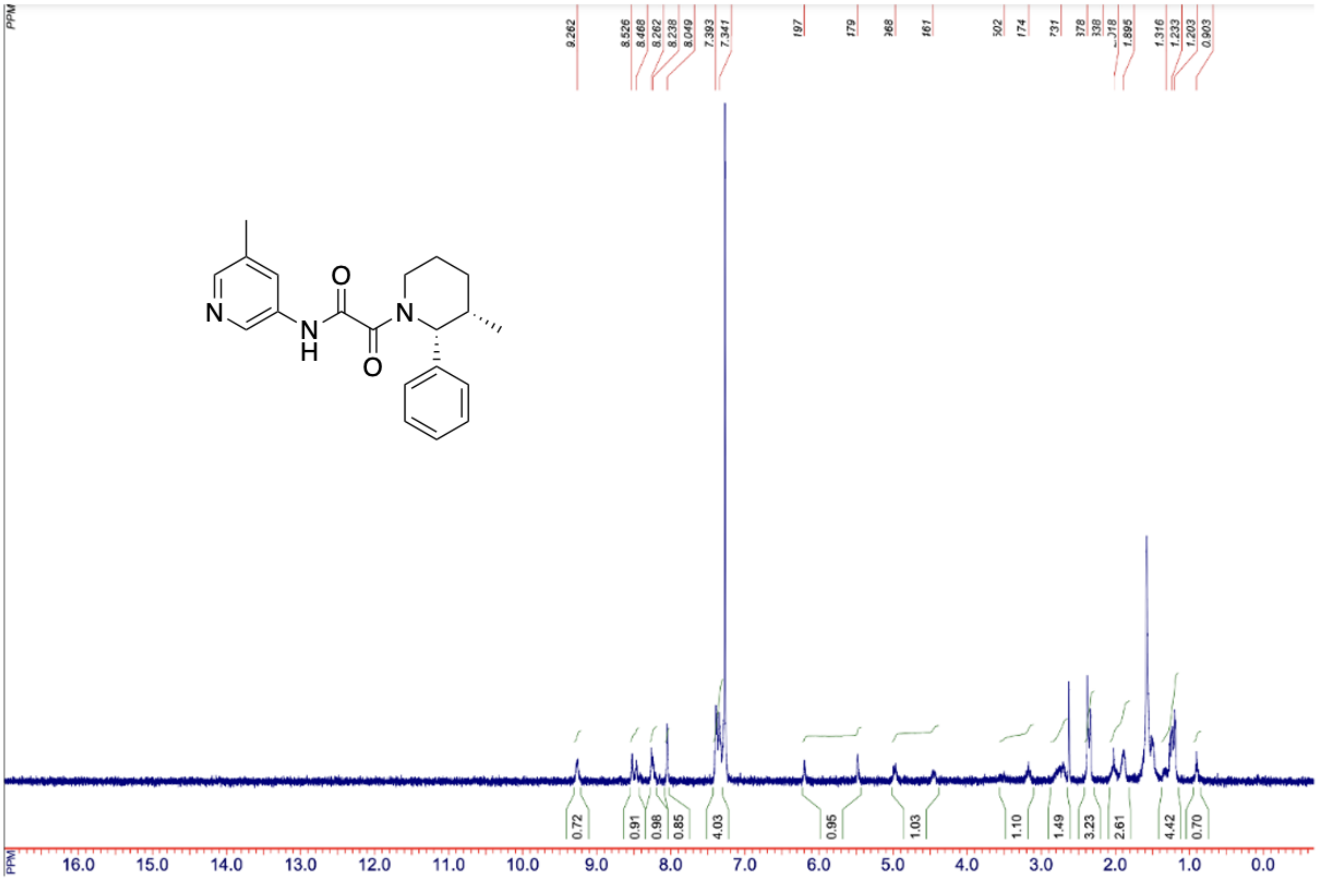

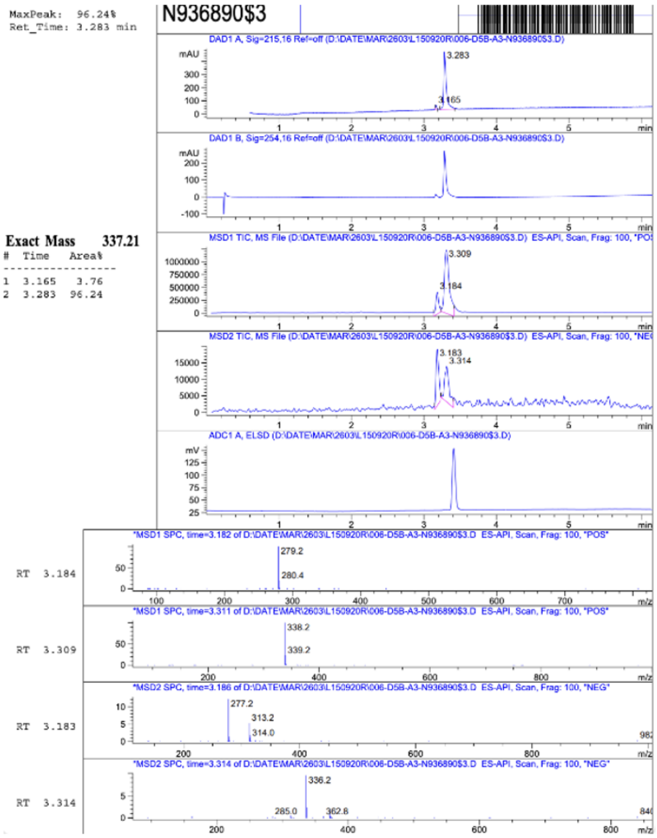

Compound 22

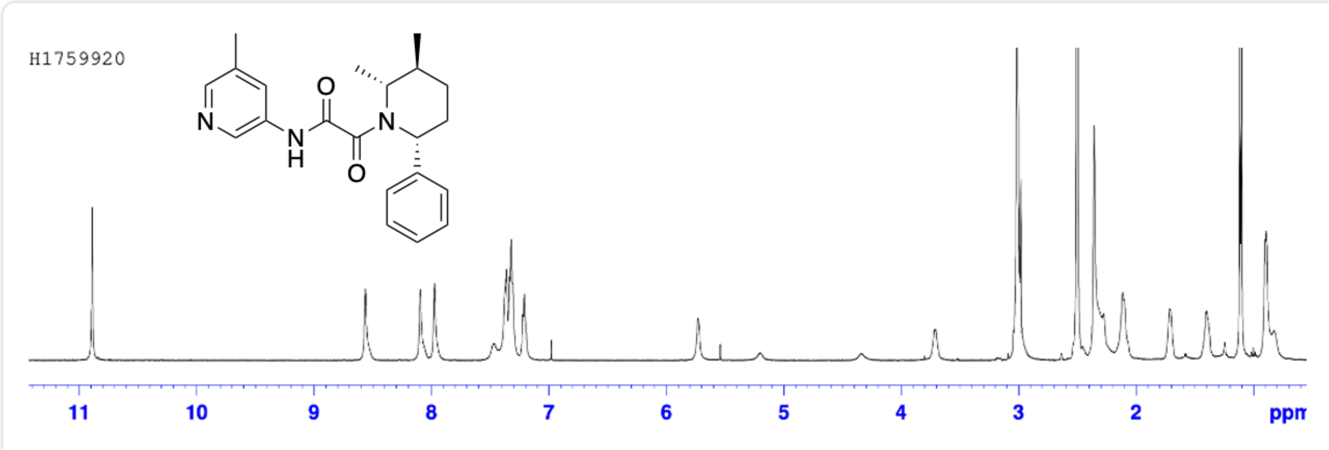

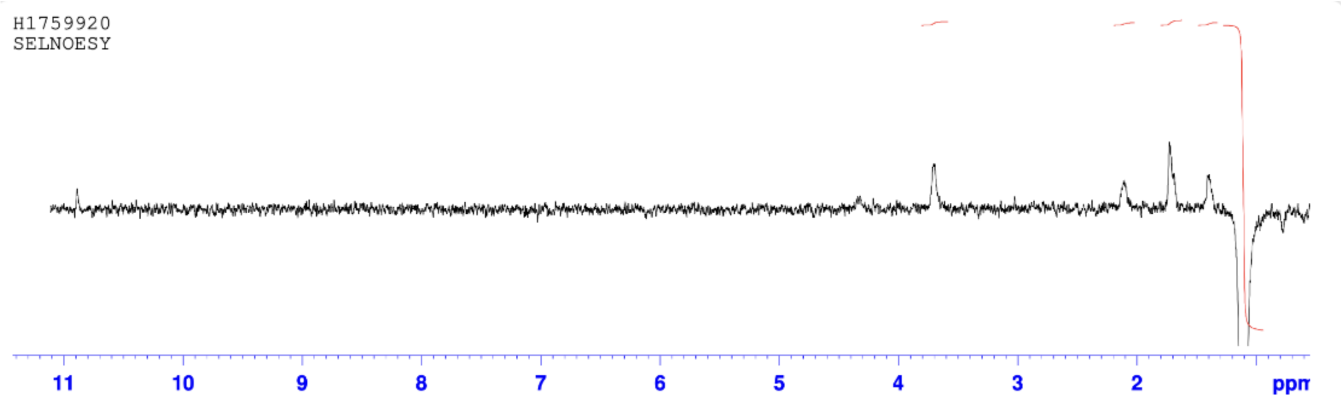

#	Time	Area%
1	4.788	100.00

RT 4.807

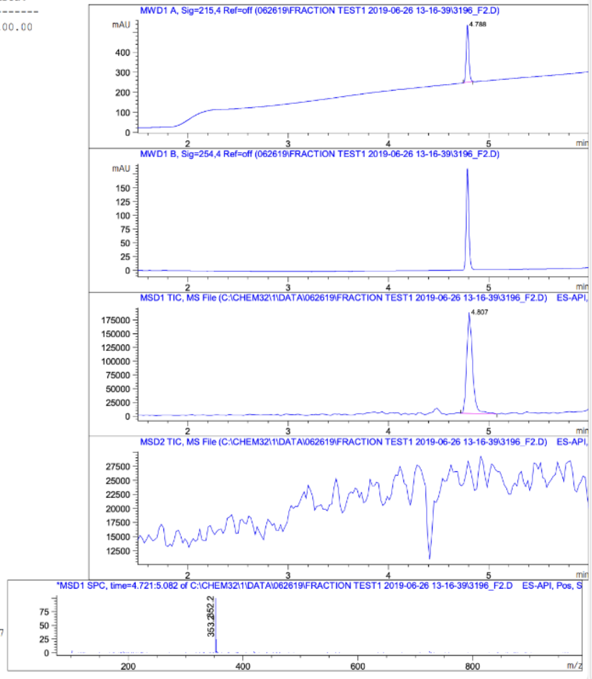

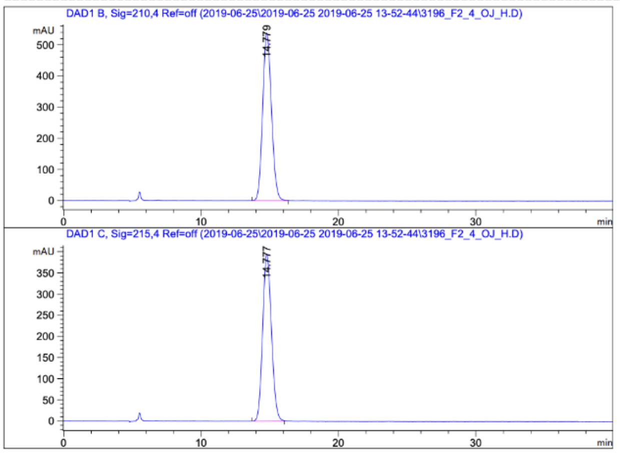

Signal: DAD1 B, Sig=210,4 Ref=off

RetTime (min)	Area,%	Symm.	Resolution	Selectivity
14.7792	100.00	0.865		

Signal: DAD1 C, Sig=215,4 Ref=off

RetTime (min)	Area,%	Symm.	Resolution	Selectivity
14.7772	100.00	0.863		

Compound 23

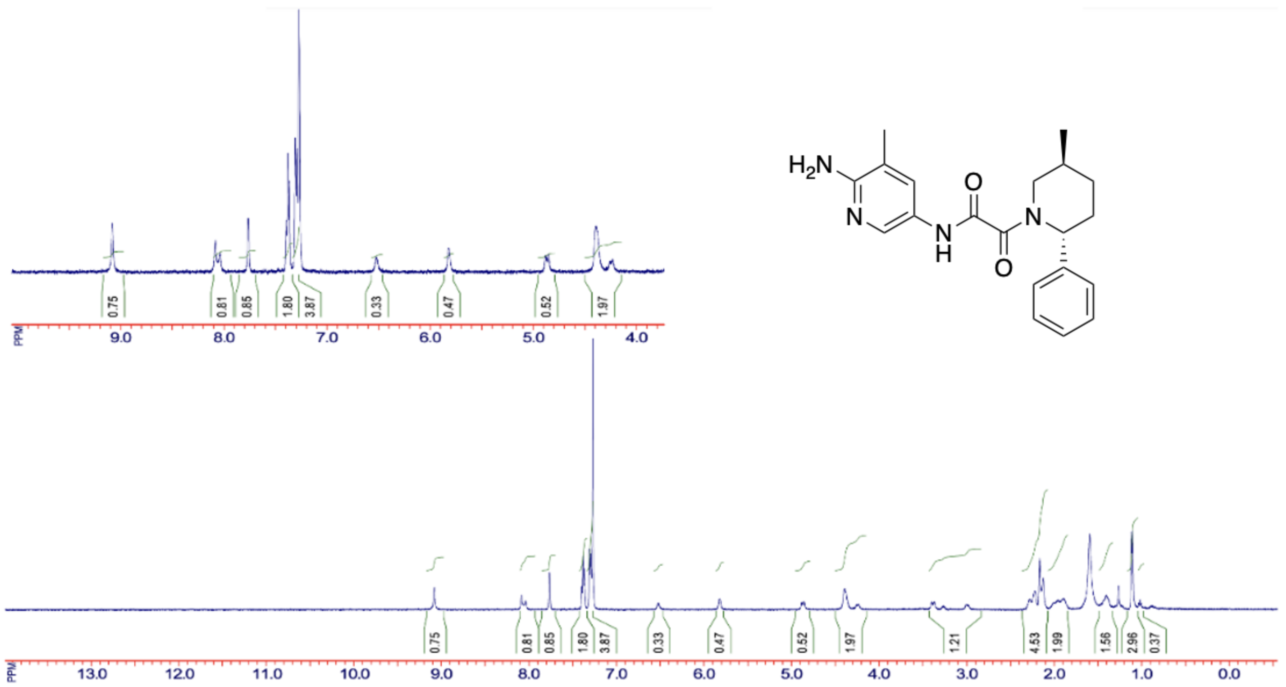

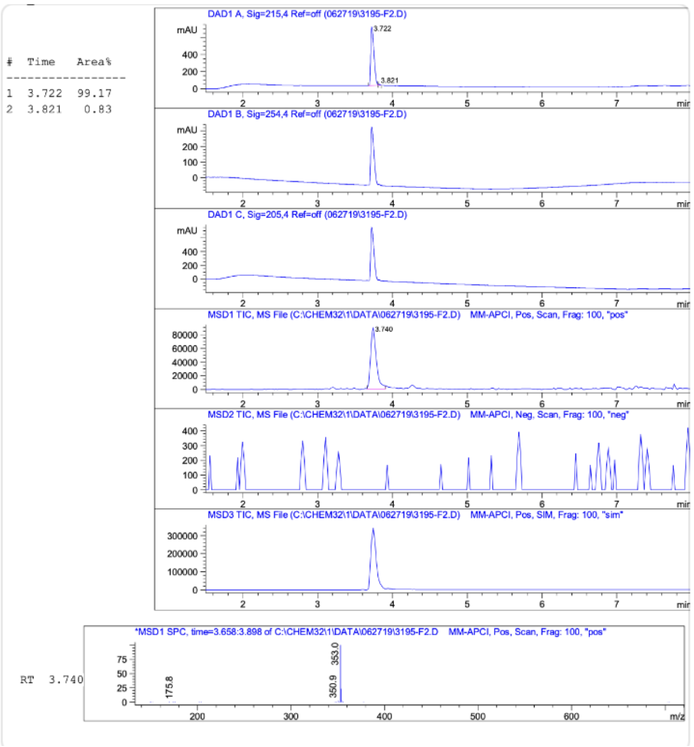

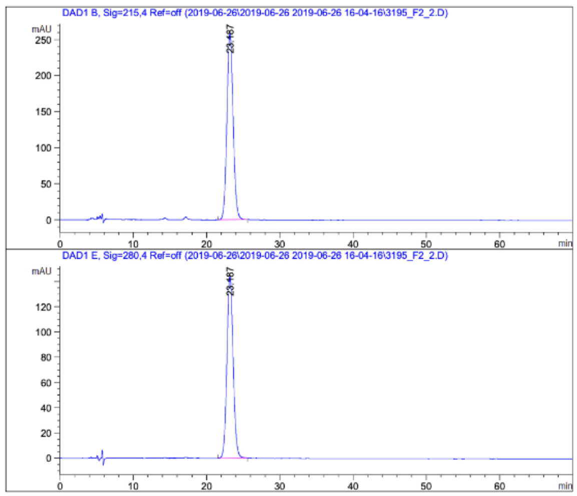

Signal: DAD1 B, Sig=215,4 Ref=off

RetTime (min)	Area,%	Symm.	Resolution	Selectivity
23.1872	100.00	0.896		

Signal: DAD1 E, Sig=280,4 Ref=off

RetTime (min)	Area,%	Symm.	Resolution	Selectivity
23.1872	100.00	0.899		

Compound 24

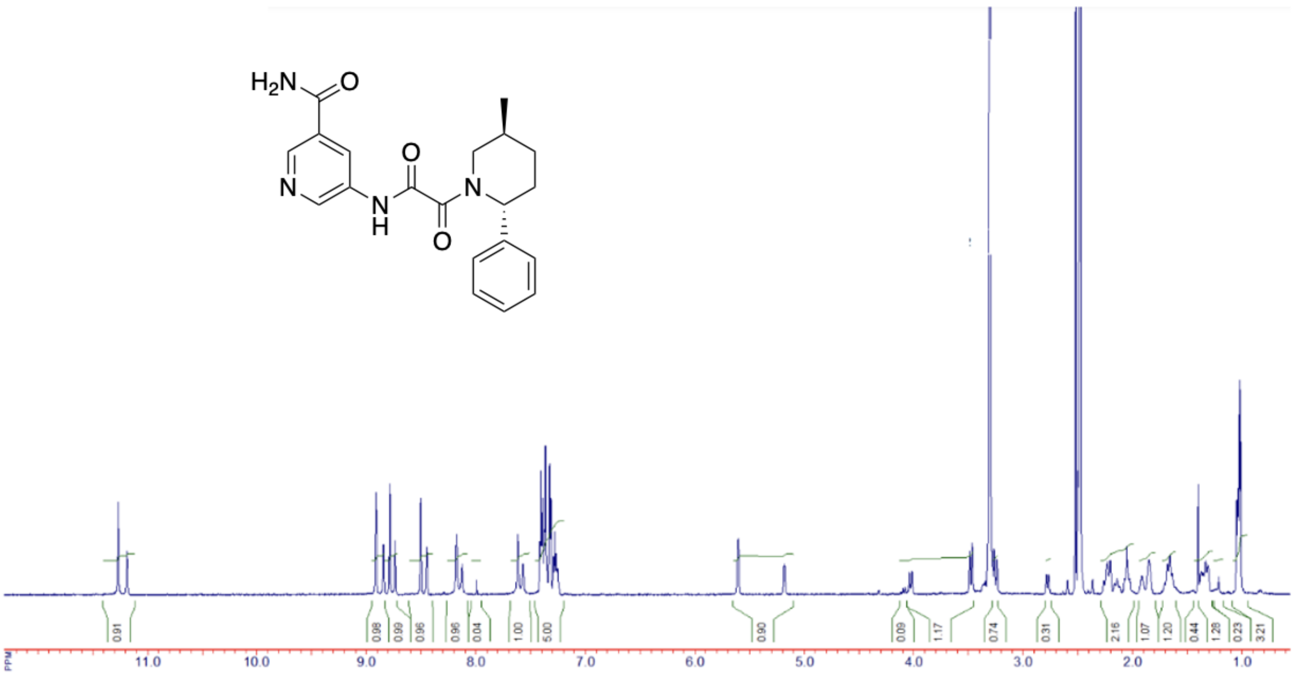
H2N
O
N
N
H
O
O
N
11.0
10.0
9.0
8.0
7.0
6.0
5.0
4.0
3.0
2.0
1.0

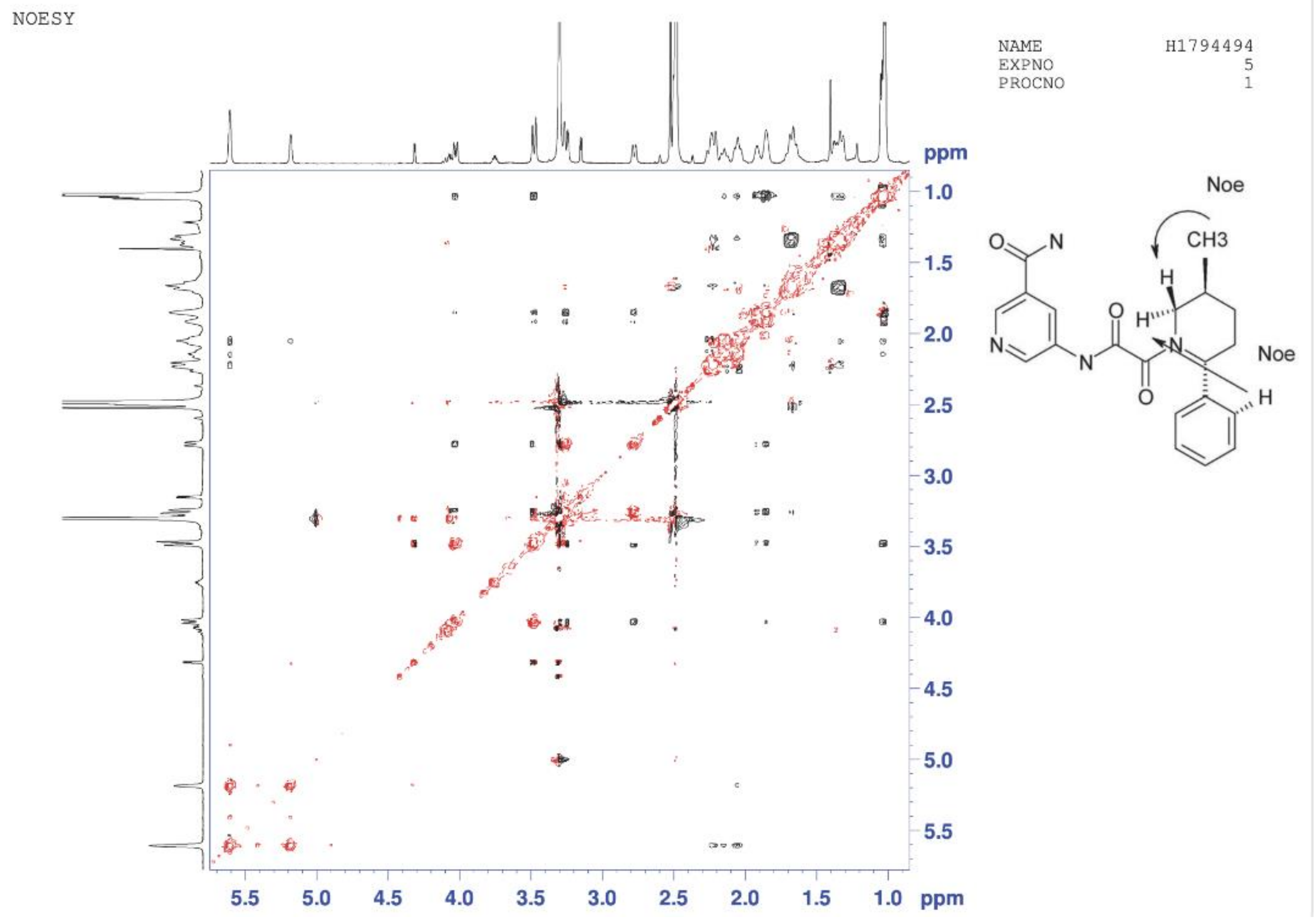
NOESY
NAME H1794494
EXPNO 5
PROCNO 1
ppm
Noe
CH3
Noe
H
5.5
5.0
4.5
4.0
3.5
3.0
2.5
2.0
1.5
1.0
ppm

HSQC

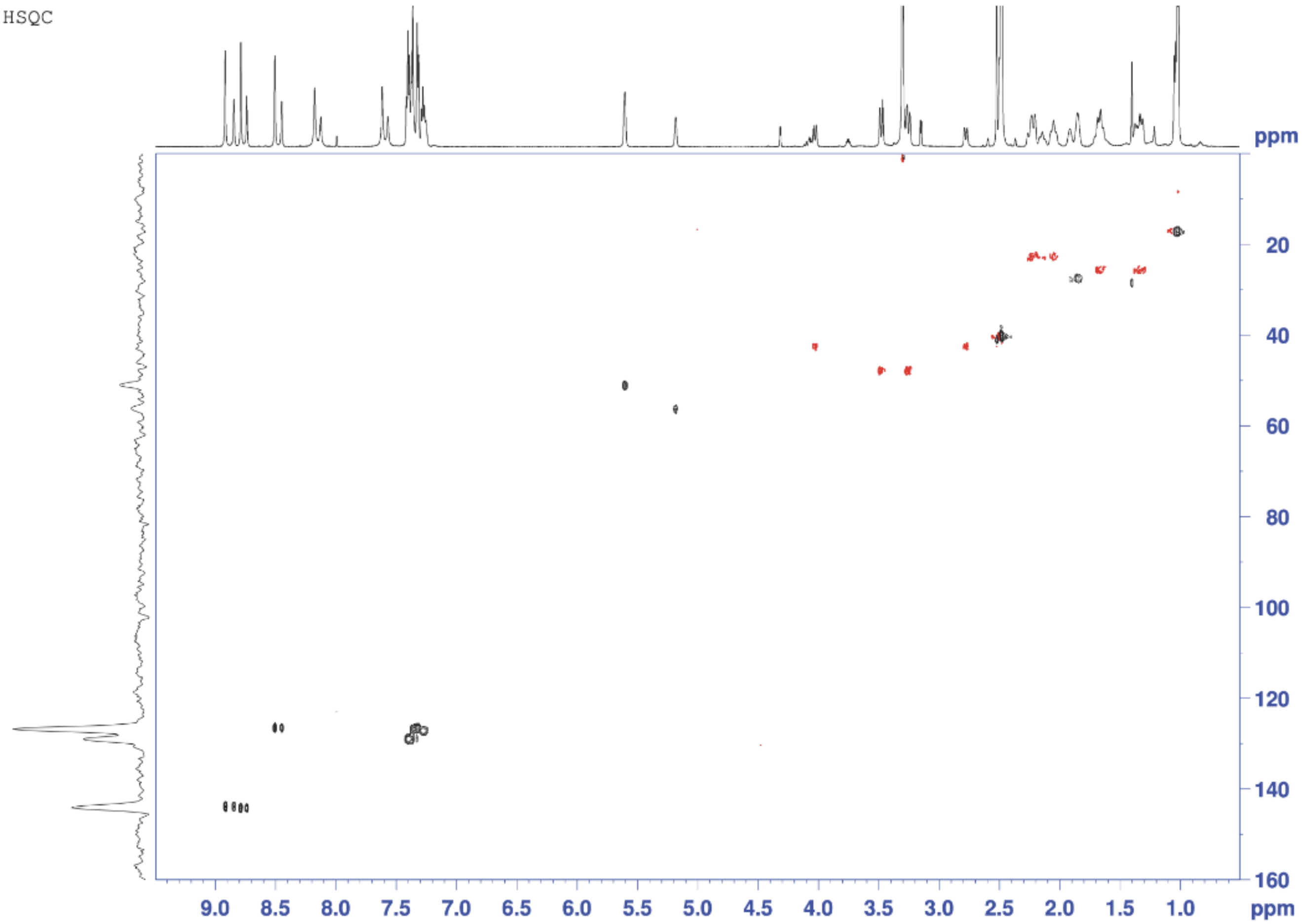

ppm
20
40
60
80
100
120
140
160
9.0
8.5
8.0
7.5
7.0
6.5
6.0
5.5
5.0
4.5
4.0
3.5
3.0
2.5
2.0
1.5
1.0
ppm

COSY

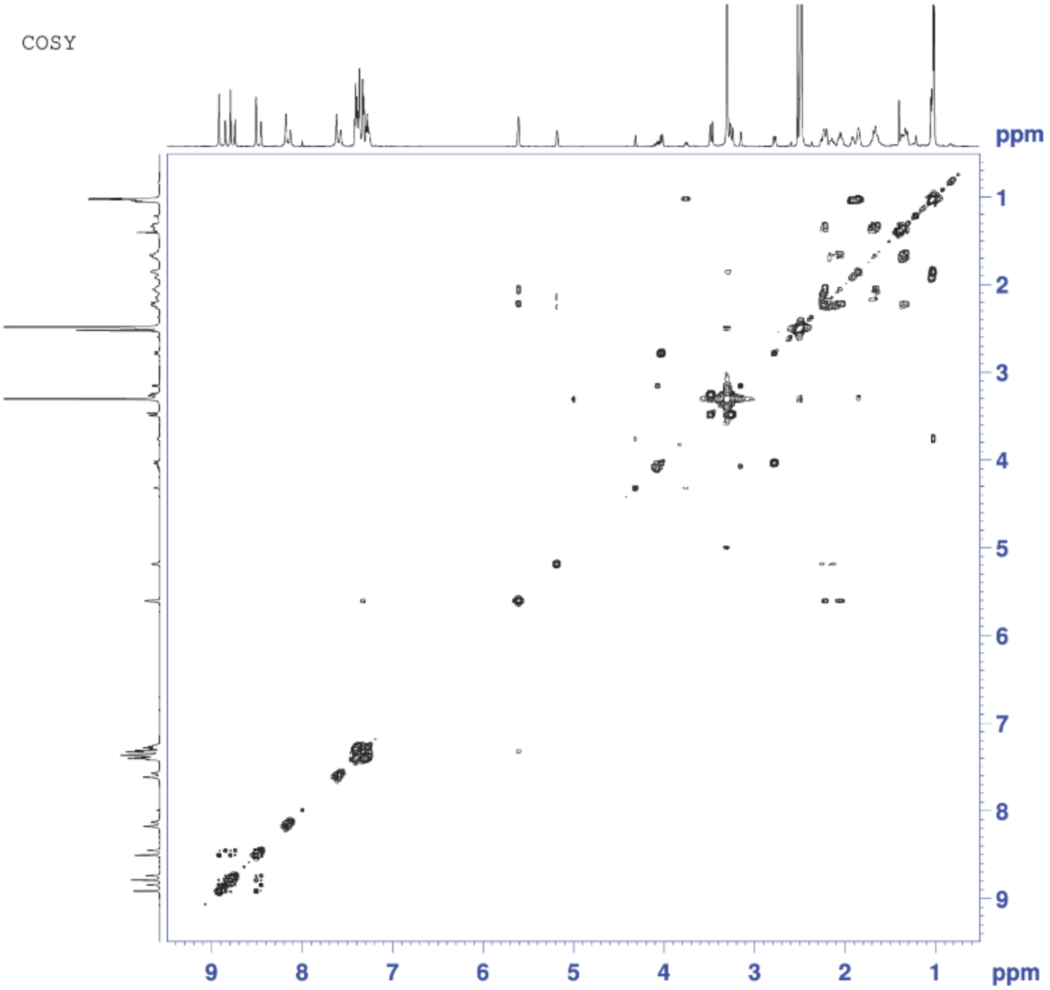

#	Time	Area%
1	4.363	98.82
2	6.870	1.18

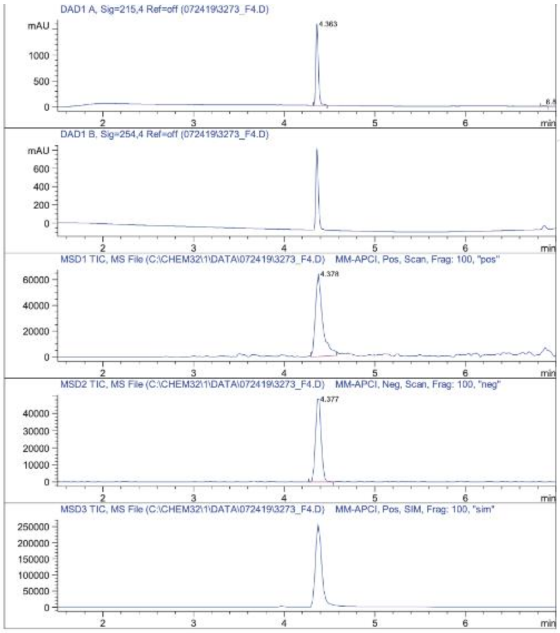

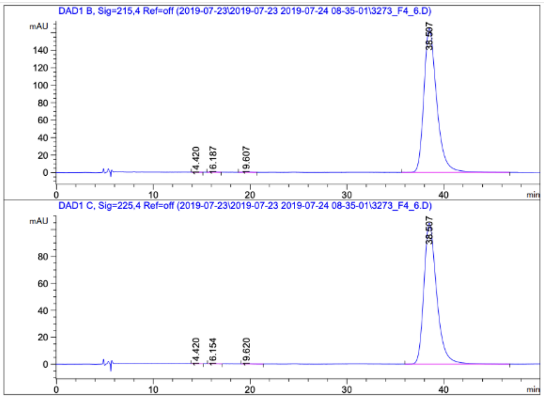

RT 4.378

RT 4.377

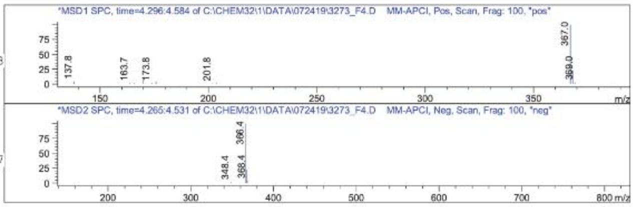

Signal: DAD1 B, Sig=215,4 Ref=off

RetTime(min)	Area,%	Symm.	Resolution	Selectivity
14.4202	0.09	0.001		
16.1872	0.19	0.002	1.72	1.12
19.6072	0.22	0.011	3.26	1.21
38.5072	99.50	0.002	11.30	1.96

Signal: DAD1 C, Sig=225,4 Ref=off

RetTime(min)	Area,%	Symm.	Resolution	Selectivity
14.4202	0.10	0.000		
16.1542	0.18	0.064	1.66	1.12
19.6202	0.22	0.001	3.35	1.21
38.5072	99.49	0.003	11.26	1.96

Compound 25

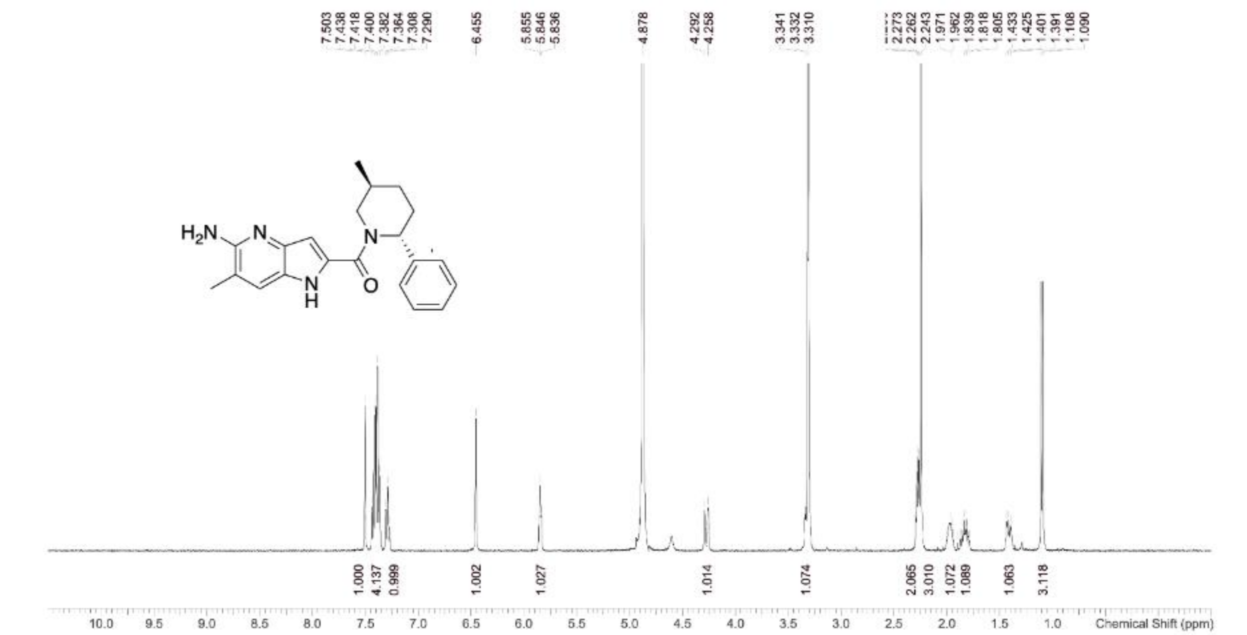

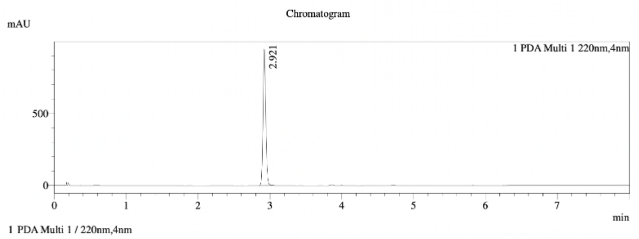

Integration Result

Peak Table

PDA Ch1 220nm

Peak#	Ret. Time	Height	Height%	USP Width	Area	Area%
1	2.921	945014	100.000	0.073	2535800	100.000

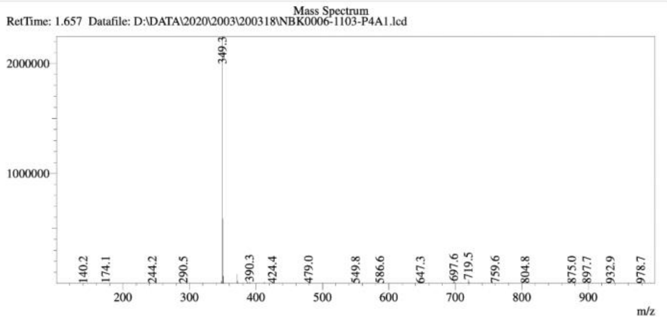

Compound 26

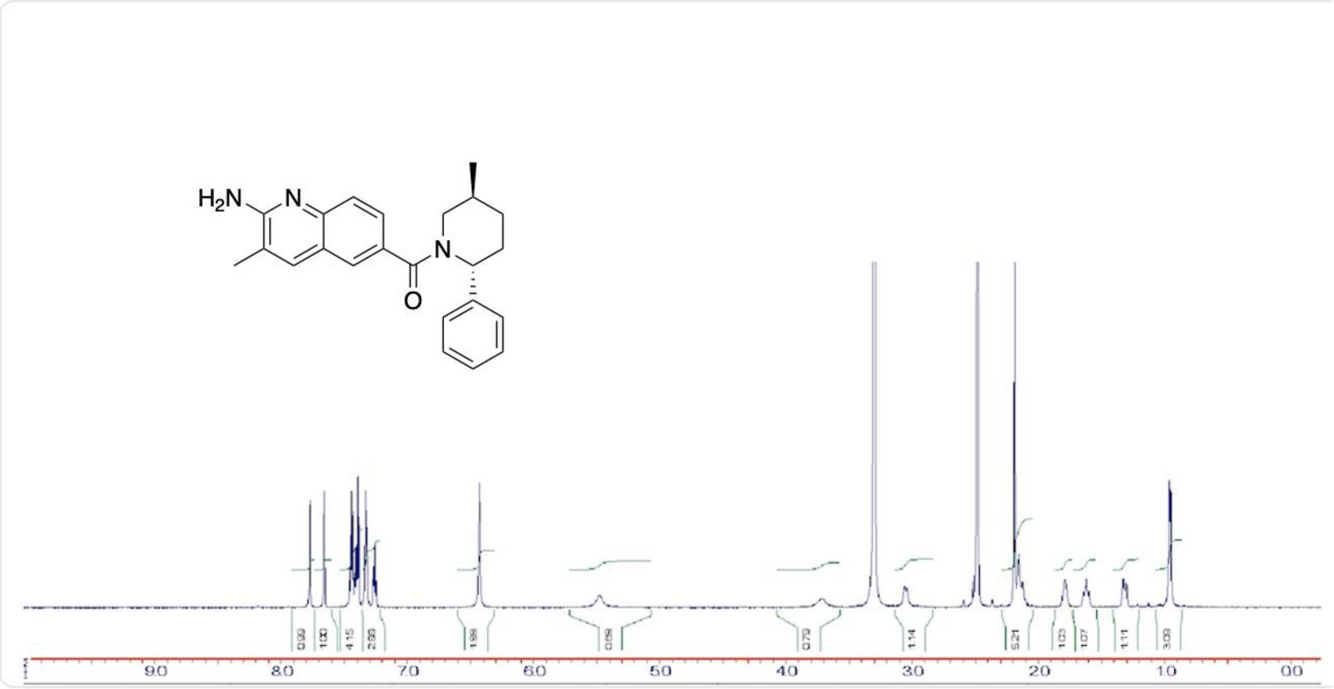

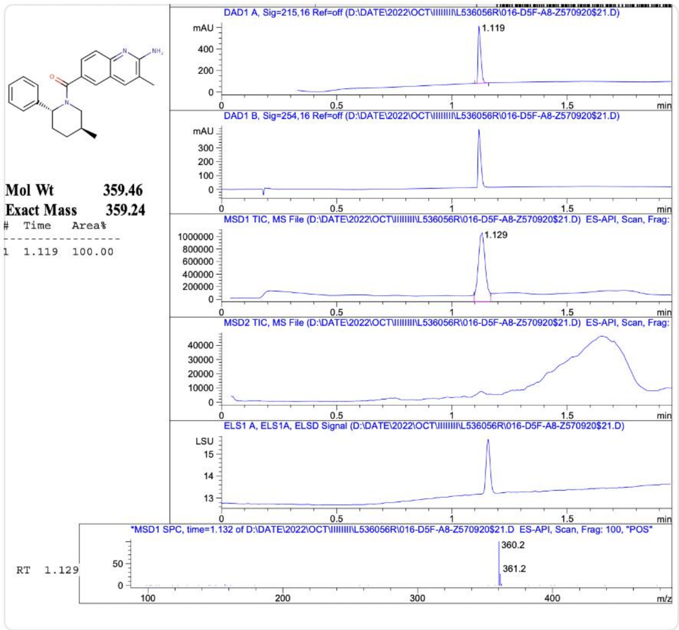

Mol Wt 359.46
Exact Mass 359.24
Time Area%
1 1.119 100.00
RT 1.129
DAD1 A, Sig=215,16 Ref=off (D:\DATE\2022\OCT\IIIIIIII\L536056R\016-D5F-A8-Z570920$21.D)
DAD1 B, Sig=254,16 Ref=off (D:\DATE\2022\OCT\IIIIIIII\L536056R\016-D5F-A8-Z570920$21.D)
MSD1 TIC, MS File (D:\DATE\2022\OCT\IIIIIIII\L536056R\016-D5F-A8-Z570920$21.D) ES-API, Scan, Frag:
MSD2 TIC, MS File (D:\DATE\2022\OCT\IIIIIIII\L536056R\016-D5F-A8-Z570920$21.D) ES-API, Scan, Frag:
ELS1 A, ELS1A, ELSD Signal (D:\DATE\2022\OCT\IIIIIIII\L536056R\016-D5F-A8-Z570920$21.D)
*MSD1 SPC, time=1.132 of D:\DATE\2022\OCT\IIIIIIII\L536056R\016-D5F-A8-Z570920$21.D ES-API, Scan, Frag: 100, "POS"
1.119
1.129
360.2
361.2

Compound 27

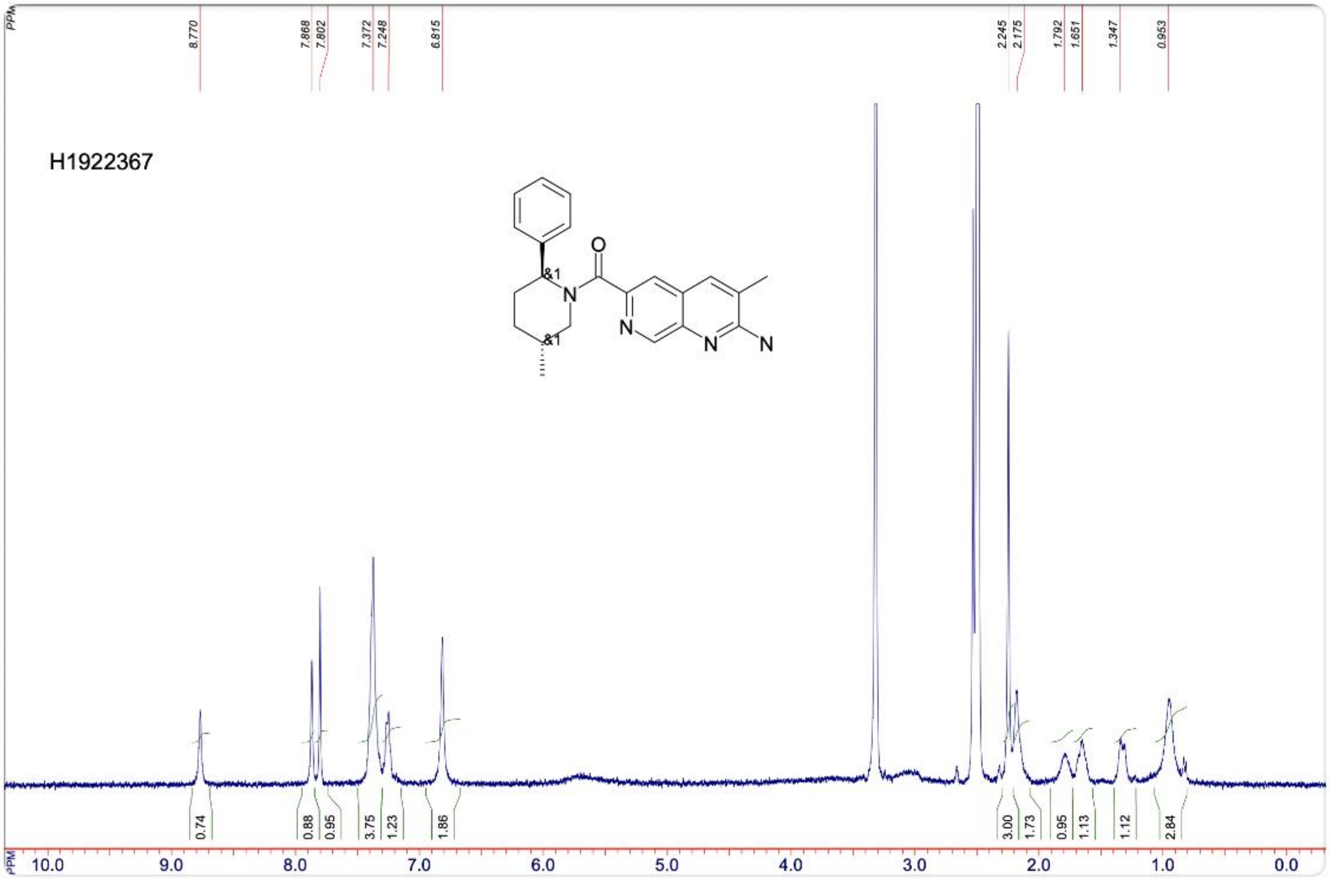

H1922367
8.770
7.868
7.802
7.372
7.248
6.815
2.245
2.175
1.792
1.651
1.347
0.953
0.74
0.88
0.95
3.75
1.23
1.86
3.00
1.73
0.95
1.13
1.12
2.84
PPM

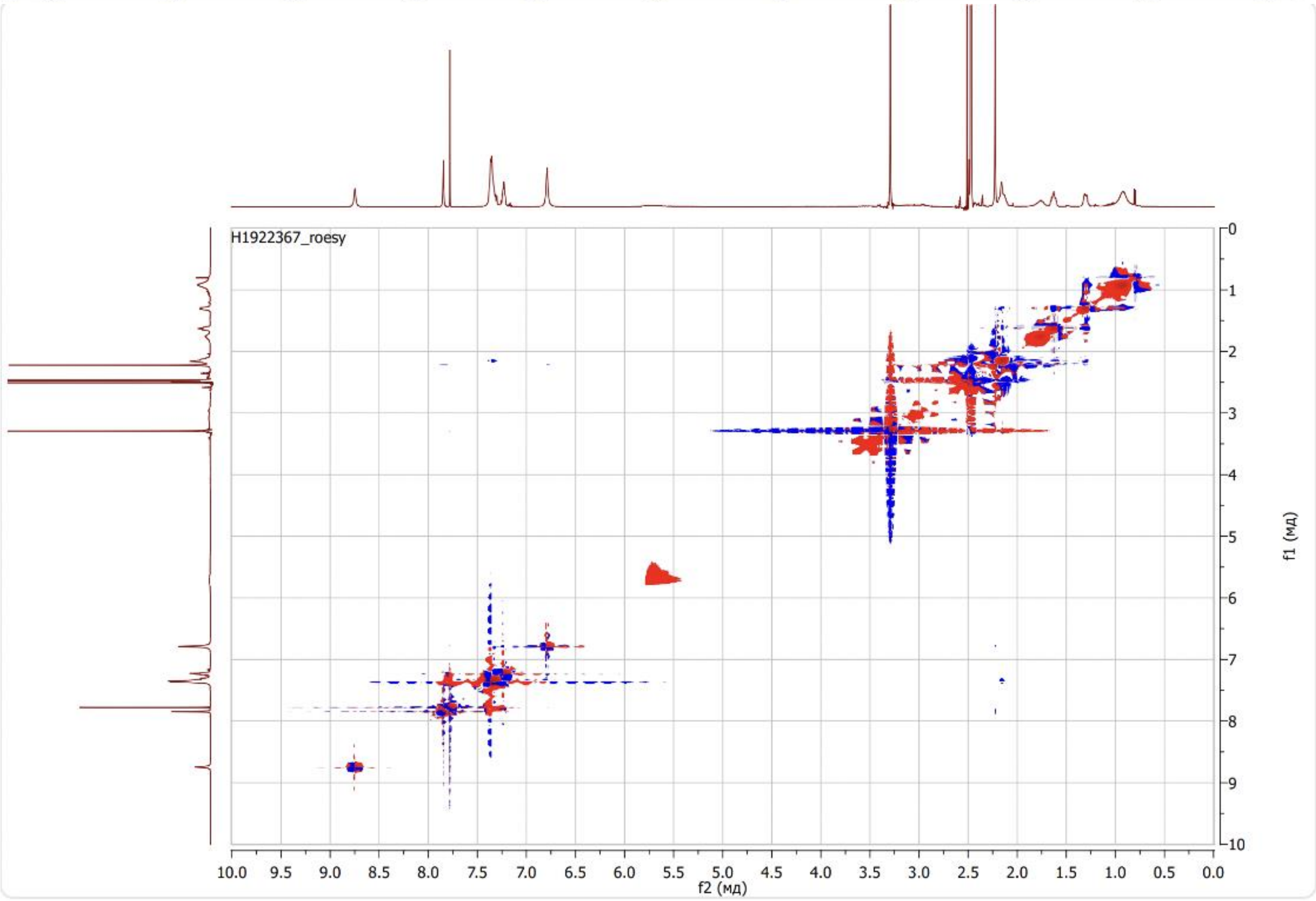

H1922367_roesy
f2 (мд)
f1 (мд)

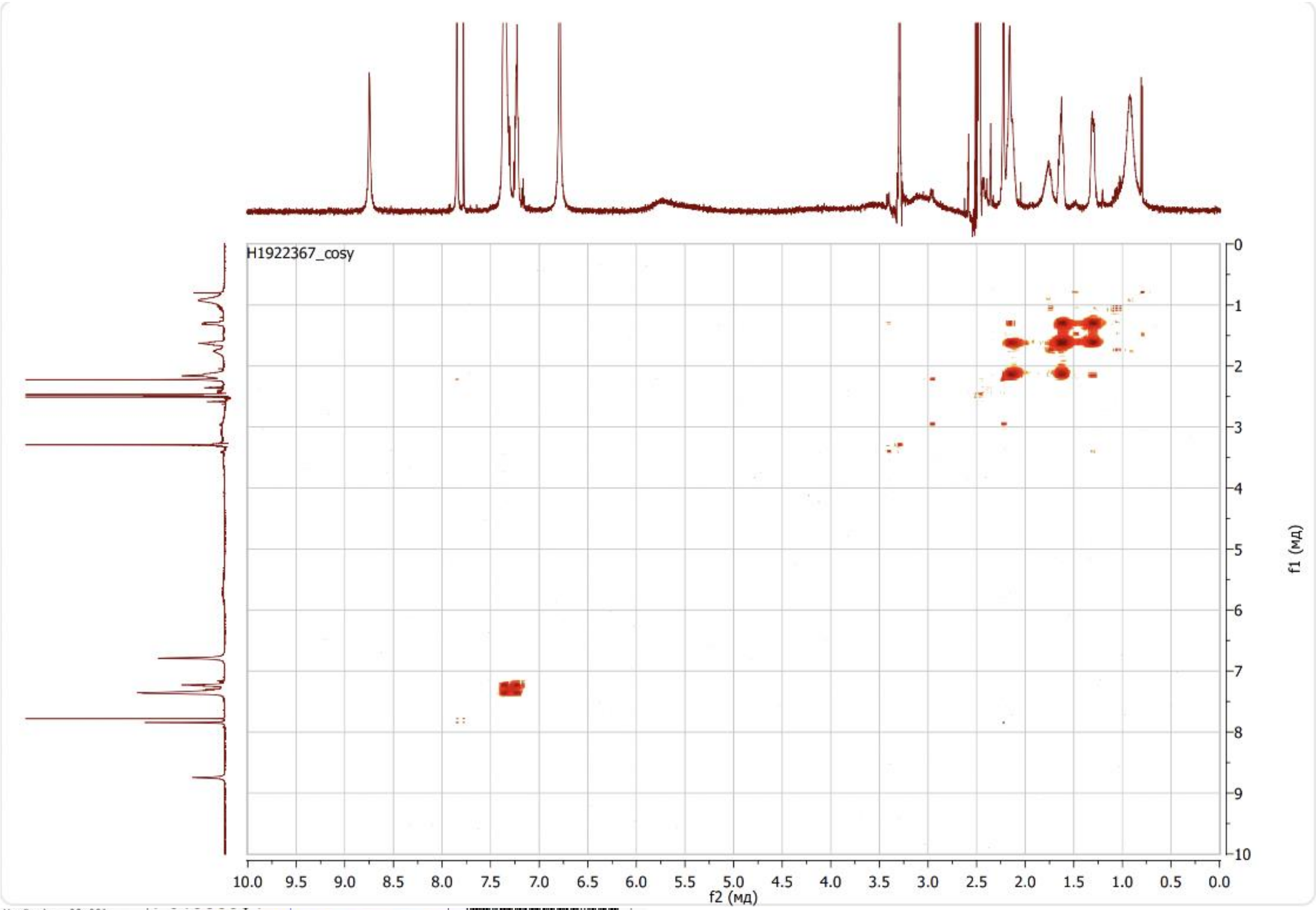

MaxPeak: 98.90%
Ret_Time: 2.511 min

Mol Wt 360.45
Exact Mass 360.23

#	Time	Area%
1	1.484	1.10
2	2.511	98.90

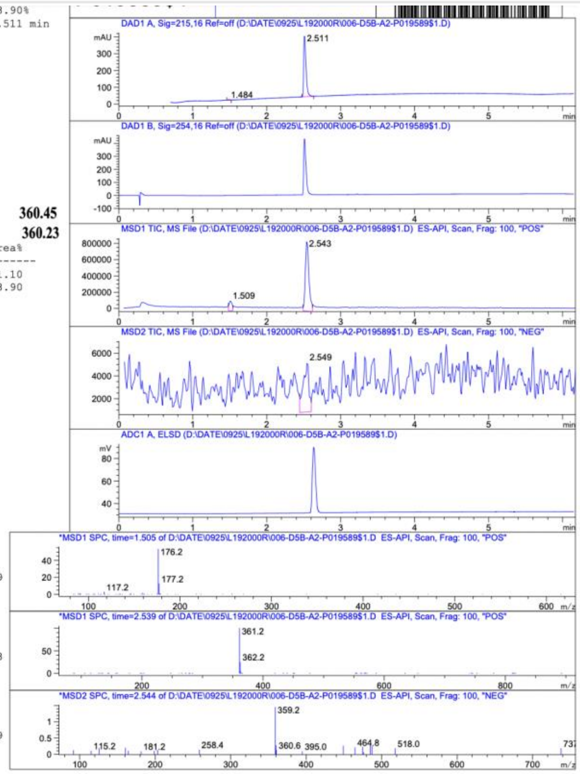

Compound 28

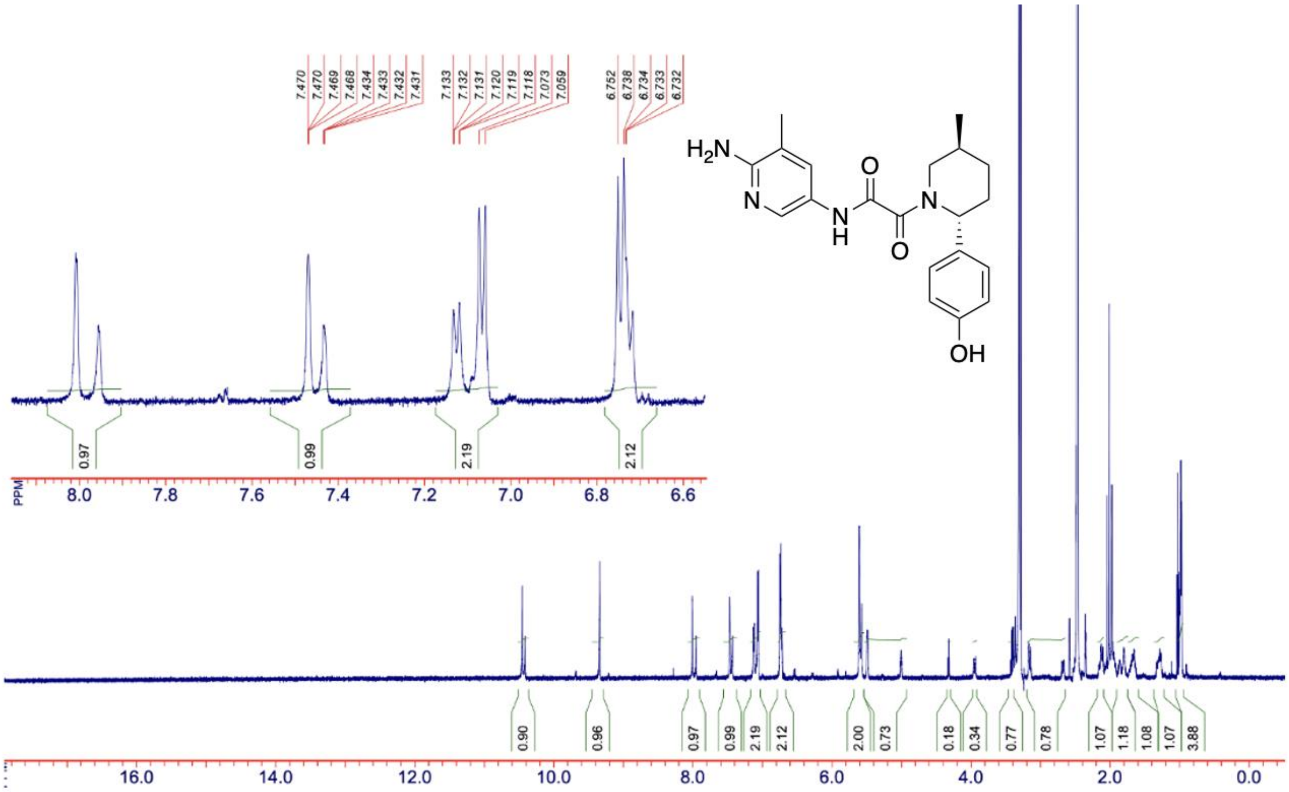

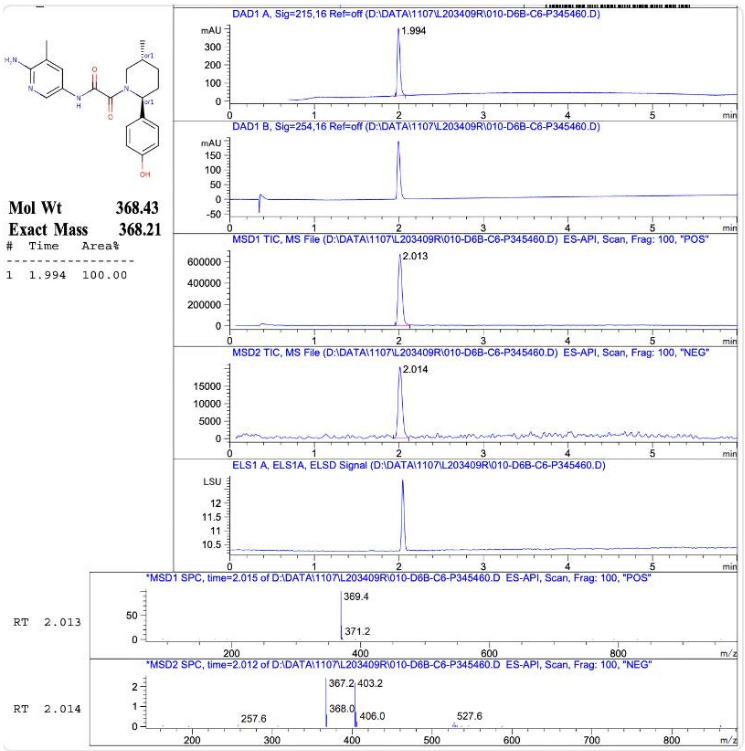
Mol Wt 368.43
Exact Mass 368.21
Time Area%
1 1.994 100.00
RT 2.013
RT 2.014

Compound 29

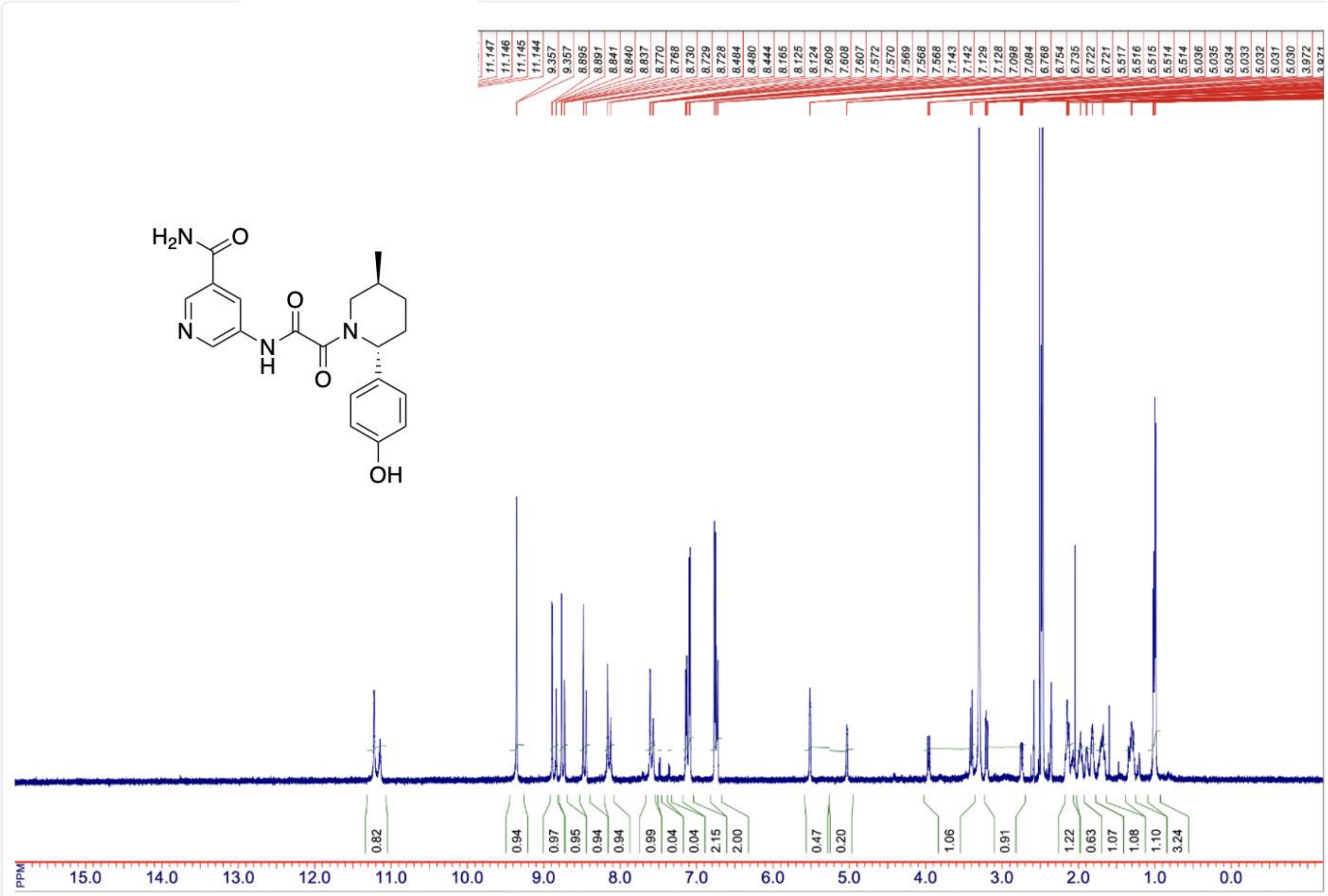

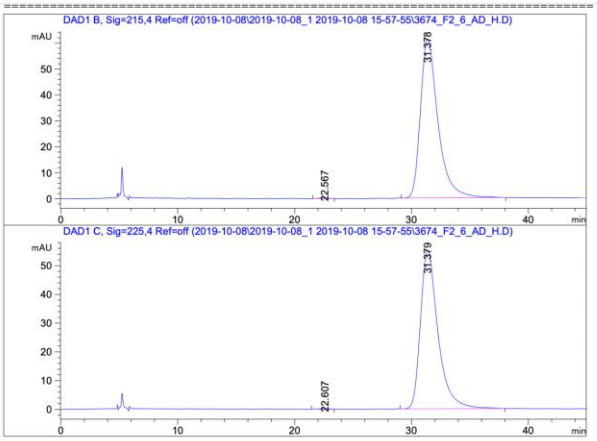

Signal: DAD1 B, Sig=215,4 Ref=off

RetTime(min)	Area,%	Symm.	Resolution	Selectivity
22.5672	0.22	1.326		
31.3782	99.78	0.655	4.03	1.48

Signal: DAD1 C, Sig=225,4 Ref=off

RetTime(min)	Area,%	Symm.	Resolution	Selectivity
22.6072	0.25	1.695		
31.3792	99.75	0.659	4.12	1.48

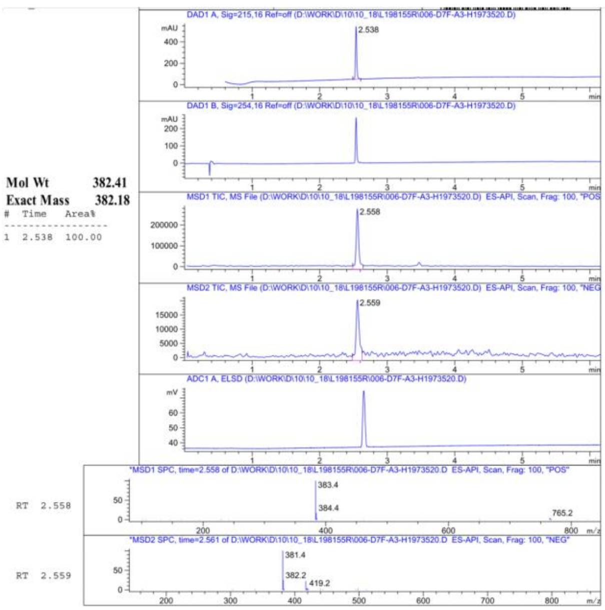

Compound 30

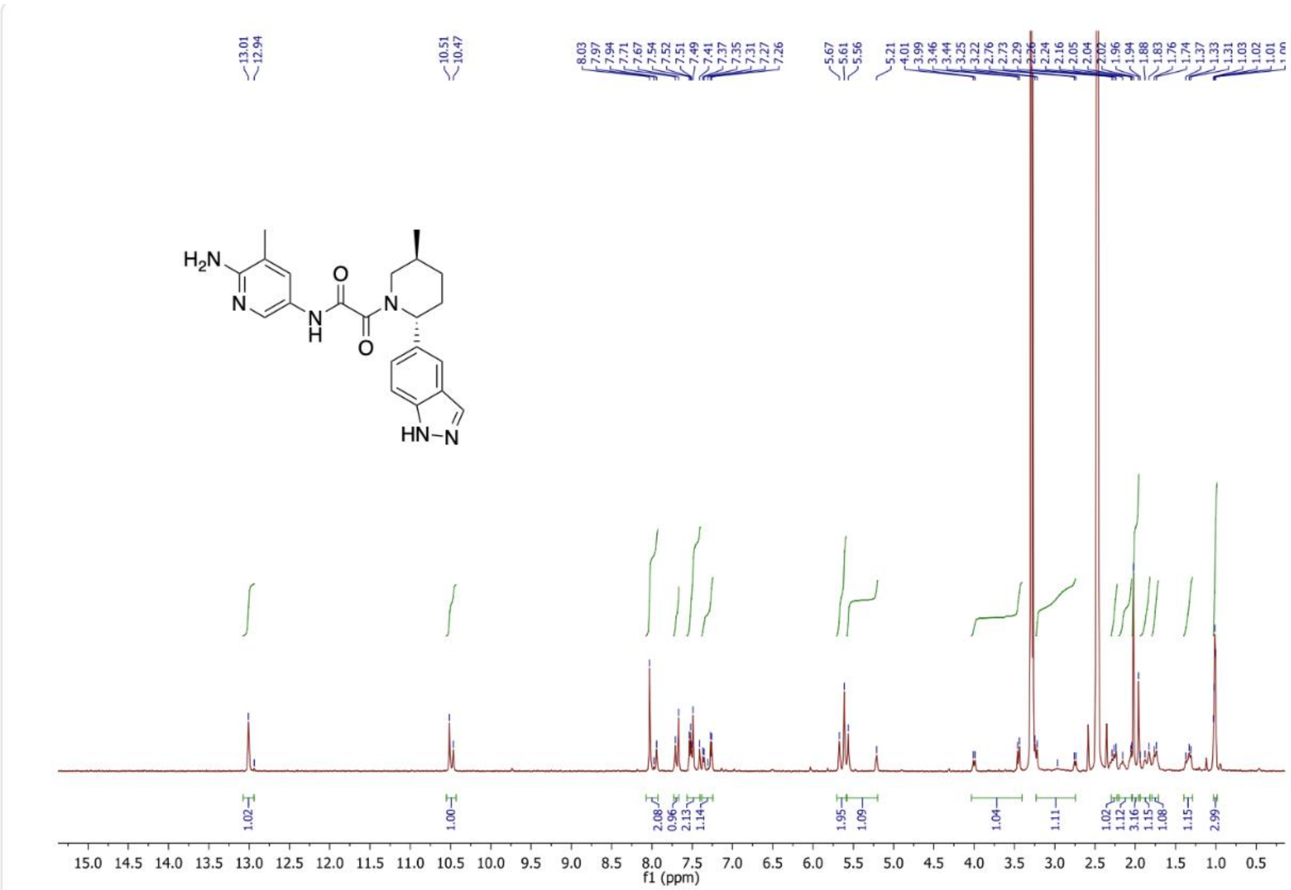
13.01
12.94
10.51
10.47
8.03
7.97
7.94
7.71
7.67
7.54
7.52
7.51
7.49
7.41
7.37
7.35
7.31
7.27
7.26
5.67
5.61
5.56
5.21
4.01
3.99
3.46
3.44
3.25
3.22
2.76
2.73
2.29
2.26
2.24
2.16
2.05
2.04
2.02
1.96
1.94
1.88
1.83
1.76
1.74
1.37
1.33
1.31
1.03
1.02
1.01
H_2N
N
N
H
O
O
N
HN–N
1.02
1.00
2.08
0.96
2.13
1.14
1.95
1.09
1.04
1.11
1.02
1.12
3.16
1.15
1.08
1.15
2.99
15.0 14.5 14.0 13.5 13.0 12.5 12.0 11.5 11.0 10.5 10.0 9.5 9.0 8.5 8.0 7.5 7.0 6.5 6.0 5.5 5.0 4.5 4.0 3.5 3.0 2.5 2.0 1.5 1.0 0.5
f1 (ppm)

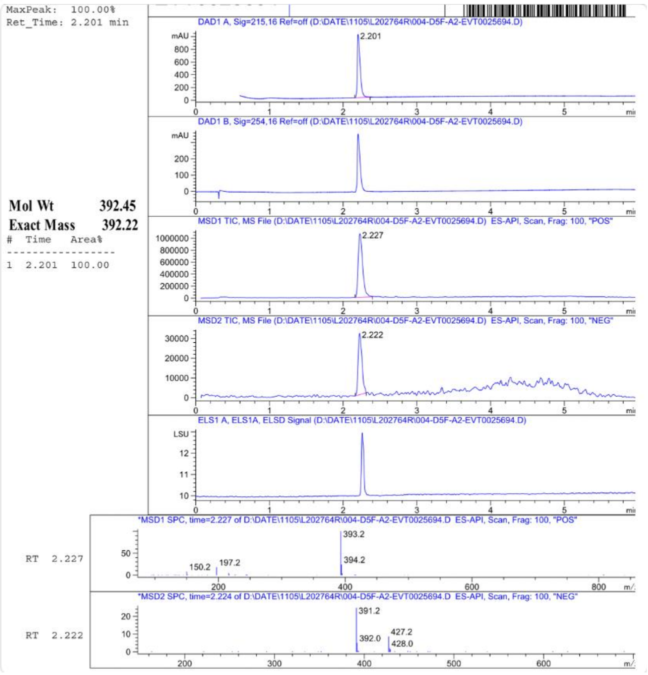
MaxPeak: 100.00%
Ret_Time: 2.201 min
Mol Wt 392.45
Exact Mass 392.22
Time Area%
1 2.201 100.00
DAD1 A, Sig=215,16 Ref=off (D:\DATE\1105\L202764R\004-D5F-A2-EVT0025694.D)
2.201
DAD1 B, Sig=254,16 Ref=off (D:\DATE\1105\L202764R\004-D5F-A2-EVT0025694.D)
MSD1 TIC, MS File (D:\DATE\1105\L202764R\004-D5F-A2-EVT0025694.D) ES-API, Scan, Frag: 100, "POS"
2.227
MSD2 TIC, MS File (D:\DATE\1105\L202764R\004-D5F-A2-EVT0025694.D) ES-API, Scan, Frag: 100, "NEG"
2.222
ELS1 A, ELS1A, ELSD Signal (D:\DATE\1105\L202764R\004-D5F-A2-EVT0025694.D)
RT 2.227
*MSD1 SPC, time=2.227 of D:\DATE\1105\L202764R\004-D5F-A2-EVT0025694.D ES-API, Scan, Frag: 100, "POS"
393.2
394.2
150.2
197.2
RT 2.222
*MSD2 SPC, time=2.224 of D:\DATE\1105\L202764R\004-D5F-A2-EVT0025694.D ES-API, Scan, Frag: 100, "NEG"
391.2
392.0
427.2
428.0

Compound 31

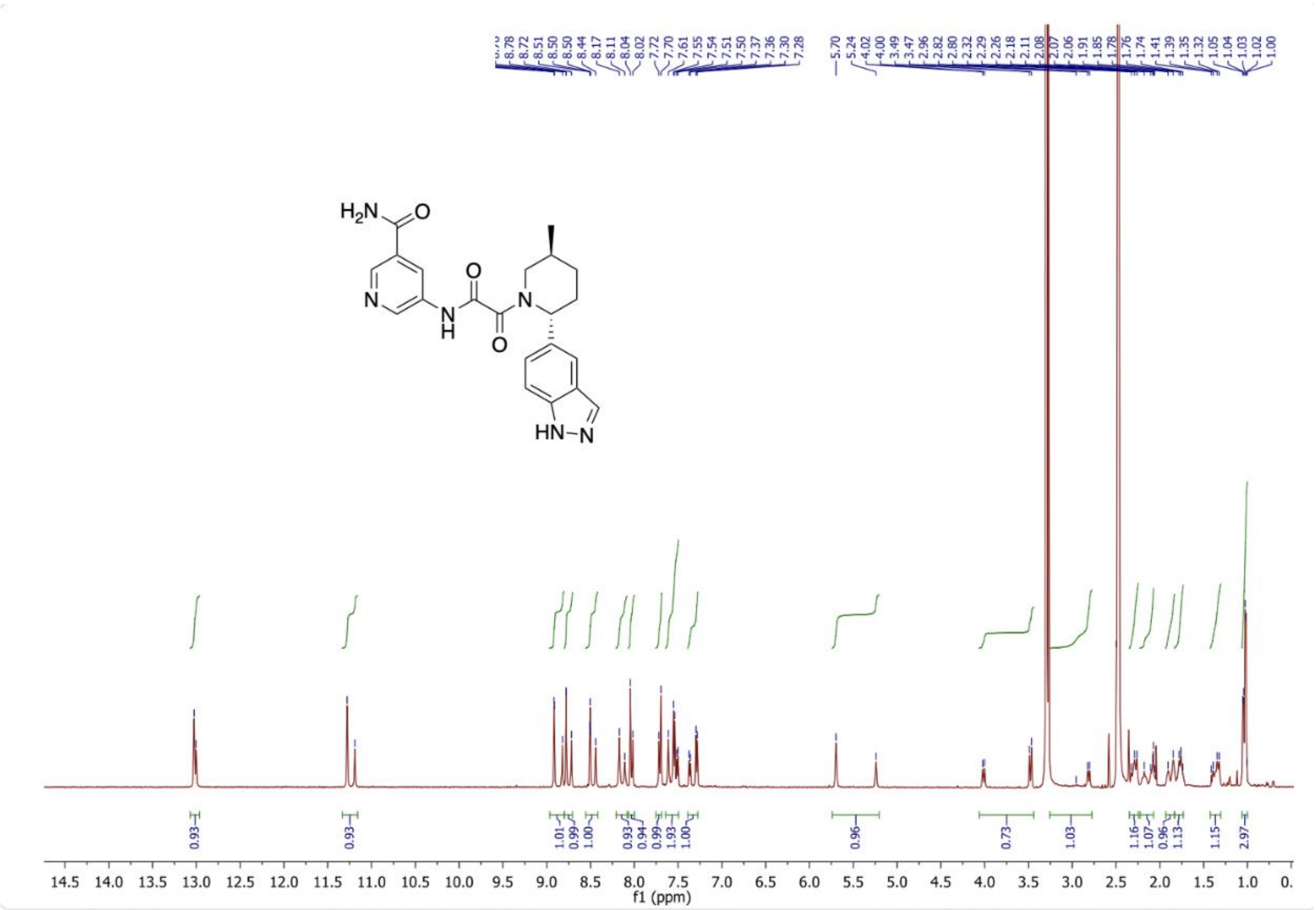

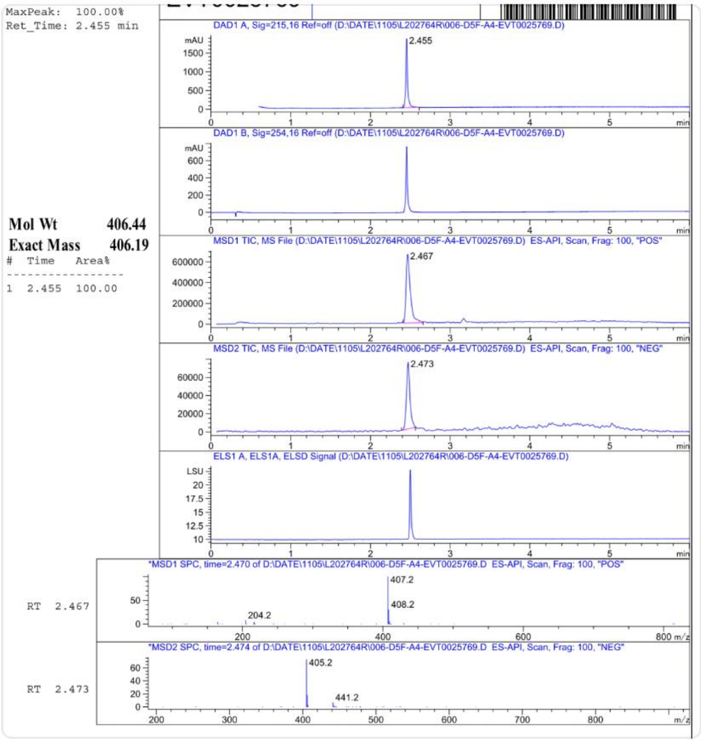

MaxPeak: 100.00%
Ret_Time: 2.455 min
Mol Wt 406.44
Exact Mass 406.19
Time Area%
1 2.455 100.00
RT 2.467
RT 2.473

Compound 32

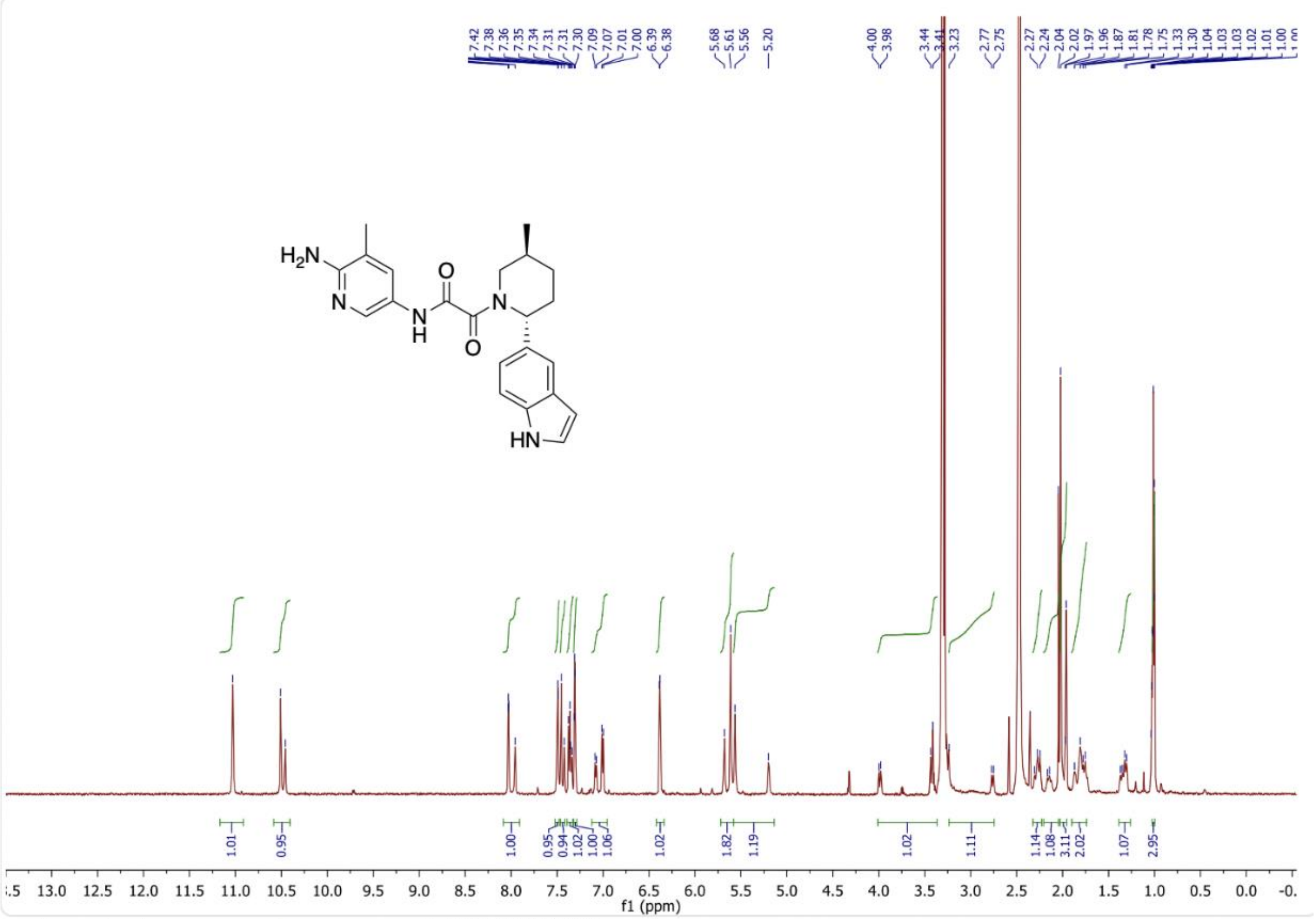
7.42
7.38
7.36
7.35
7.34
7.31
7.31
7.30
7.09
7.07
7.01
7.00
6.39
6.38
5.68
5.61
5.56
5.20
4.00
3.98
3.44
3.41
3.23
2.77
2.75
2.27
2.24
2.04
2.02
1.97
1.96
1.87
1.81
1.78
1.75
1.33
1.30
1.04
1.03
1.03
1.02
1.01
1.00
H_2N
N
N
H
O
O
N
HN
1.01
0.95
1.00
0.95
0.94
1.02
1.00
1.06
1.02
1.82
1.19
1.02
1.11
1.14
1.08
3.11
2.02
1.07
2.95
f1 (ppm)

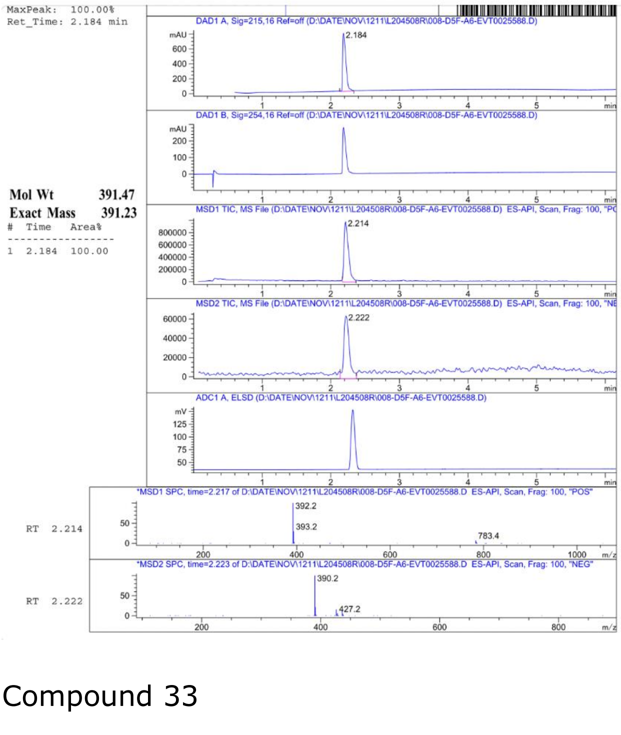
MaxPeak: 100.00%
Ret_Time: 2.184 min
DAD1 A, Sig=215,16 Ref=off (D:\DATE\NOV\1211\L204508R\008-D5F-A6-EVT0025588.D)
2.184
DAD1 B, Sig=254,16 Ref=off (D:\DATE\NOV\1211\L204508R\008-D5F-A6-EVT0025588.D)
Mol Wt 391.47
Exact Mass 391.23
Time Area%
1 2.184 100.00
MSD1 TIC, MS File (D:\DATE\NOV\1211\L204508R\008-D5F-A6-EVT0025588.D) ES-API, Scan, Frag: 100, "PO
2.214
MSD2 TIC, MS File (D:\DATE\NOV\1211\L204508R\008-D5F-A6-EVT0025588.D) ES-API, Scan, Frag: 100, "NE
2.222
ADC1 A, ELSD (D:\DATE\NOV\1211\L204508R\008-D5F-A6-EVT0025588.D)
*MSD1 SPC, time=2.217 of D:\DATE\NOV\1211\L204508R\008-D5F-A6-EVT0025588.D ES-API, Scan, Frag: 100, "POS"
RT 2.214
392.2
393.2
783.4
*MSD2 SPC, time=2.223 of D:\DATE\NOV\1211\L204508R\008-D5F-A6-EVT0025588.D ES-API, Scan, Frag: 100, "NEG"
RT 2.222
390.2
427.2

Compound 33

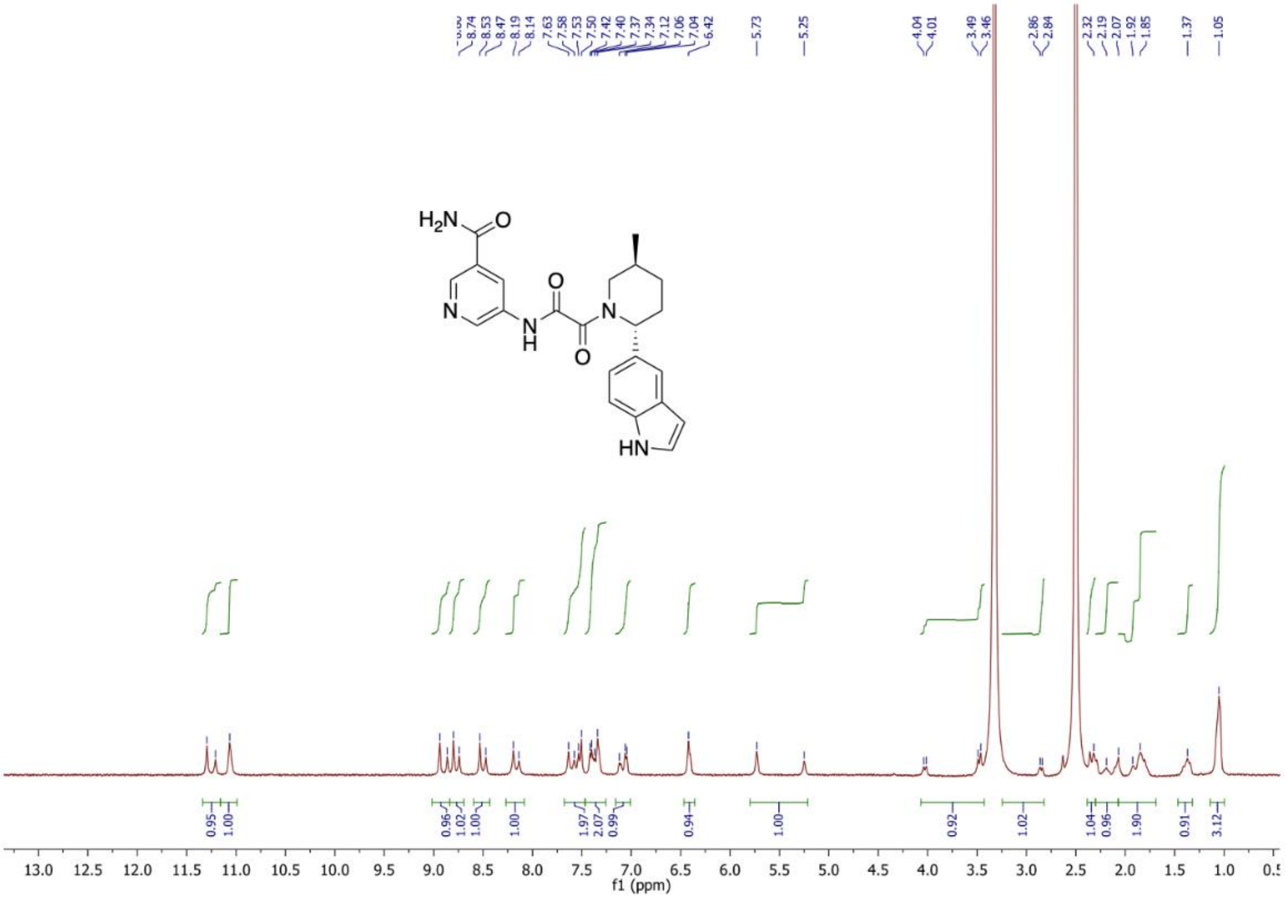

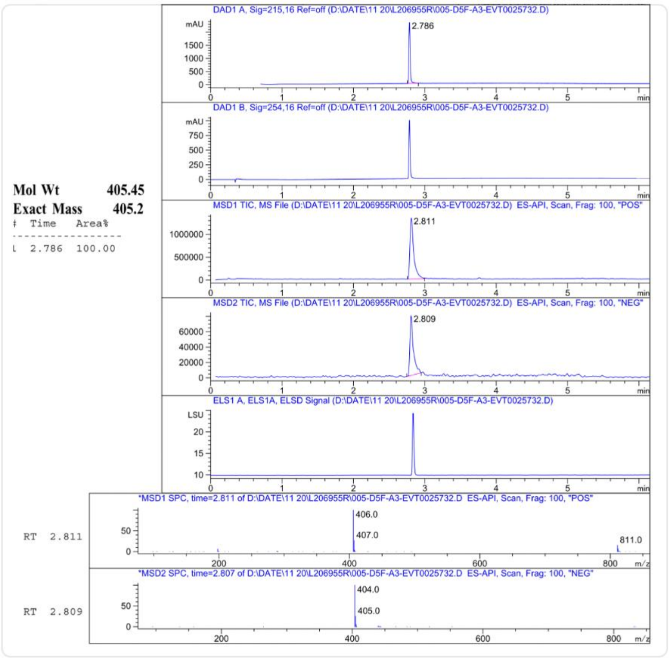

Mol Wt 405.45
Exact Mass 405.2
Time Area%
1 2.786 100.00
RT 2.811
RT 2.809

Compound 34

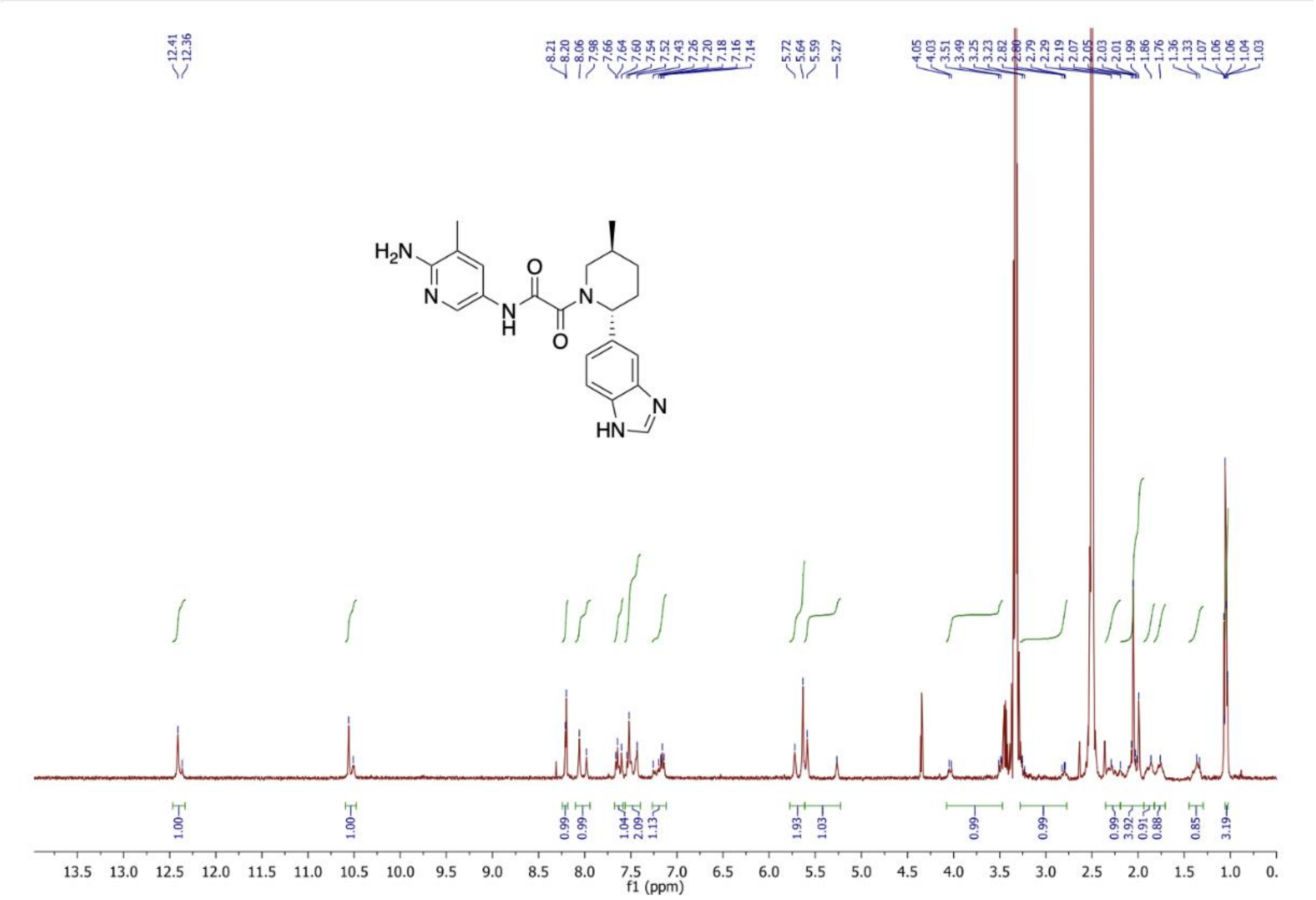
12.41
12.36
8.21 8.20 8.06 7.98 7.66 7.64 7.60 7.54 7.52 7.43 7.26 7.20 7.18 7.16 7.14
5.72 5.64 5.59 5.27
4.05 4.03 3.51 3.49 3.25 3.23 2.82 2.80 2.79 2.29 2.19 2.07 2.05 2.03 2.01 1.99 1.86 1.76 1.36 1.33 1.07 1.06 1.06 1.04 1.03
H2N
N
N
H
O
O
N
HN
N
1.00 1.00 0.99 0.99 1.04 2.09 1.13 1.93 1.03 0.99 0.99 0.99 3.92 0.91 0.88 0.85 3.19
f1 (ppm)

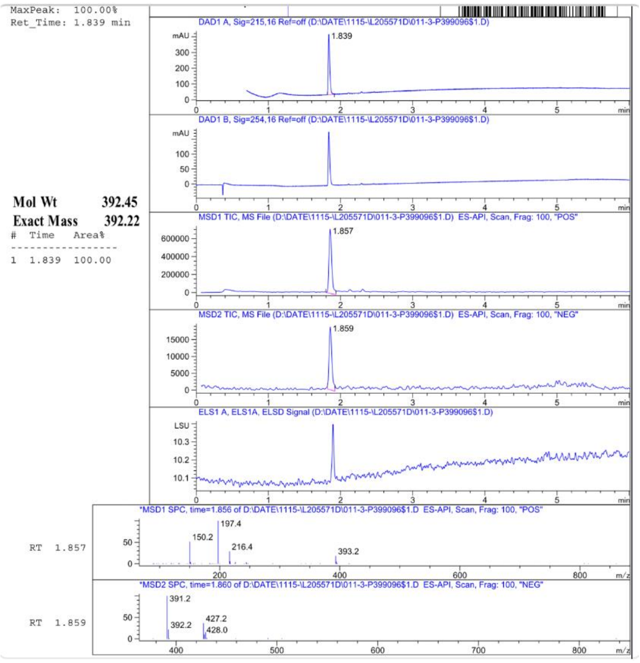
MaxPeak: 100.00%
Ret_Time: 1.839 min
Mol Wt 392.45
Exact Mass 392.22
Time Area%
1 1.839 100.00
DAD1 A, Sig=215,16 Ref=off (D:\DATE\1115-\L205571D\011-3-P399096$1.D)
DAD1 B, Sig=254,16 Ref=off (D:\DATE\1115-\L205571D\011-3-P399096$1.D)
MSD1 TIC, MS File (D:\DATE\1115-\L205571D\011-3-P399096$1.D) ES-API, Scan, Frag: 100, "POS"
MSD2 TIC, MS File (D:\DATE\1115-\L205571D\011-3-P399096$1.D) ES-API, Scan, Frag: 100, "NEG"
ELS1 A, ELS1A, ELSD Signal (D:\DATE\1115-\L205571D\011-3-P399096$1.D)
*MSD1 SPC, time=1.856 of D:\DATE\1115-\L205571D\011-3-P399096$1.D ES-API, Scan, Frag: 100, "POS"
RT 1.857
150.2 197.4 216.4 393.2
*MSD2 SPC, time=1.860 of D:\DATE\1115-\L205571D\011-3-P399096$1.D ES-API, Scan, Frag: 100, "NEG"
RT 1.859
391.2 392.2 427.2 428.0

Compound 35

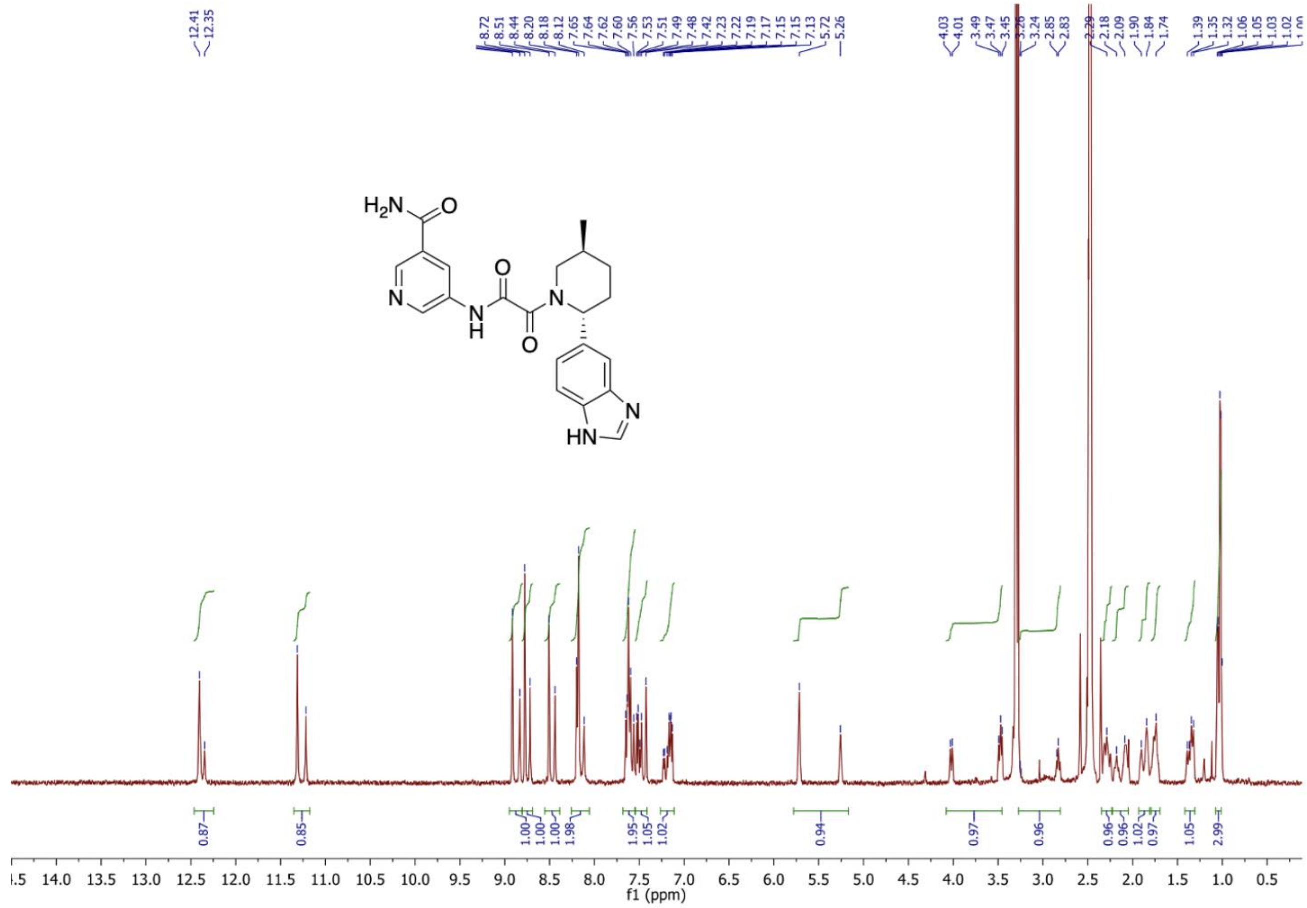

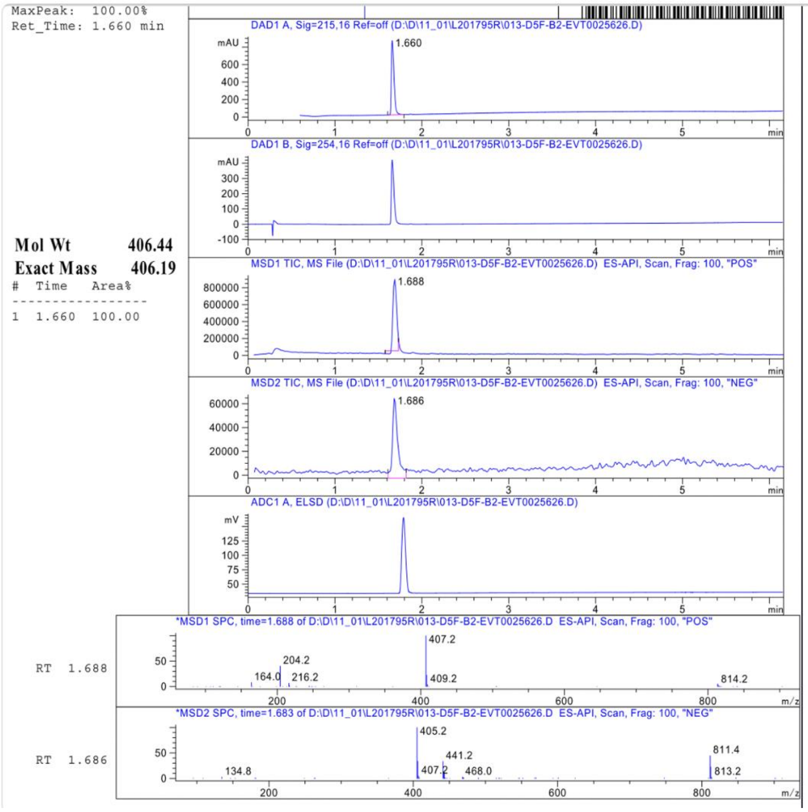
MaxPeak: 100.00%
Ret_Time: 1.660 min
Mol Wt 406.44
Exact Mass 406.19
Time Area%
1 1.660 100.00
RT 1.688
RT 1.686

Compound 36

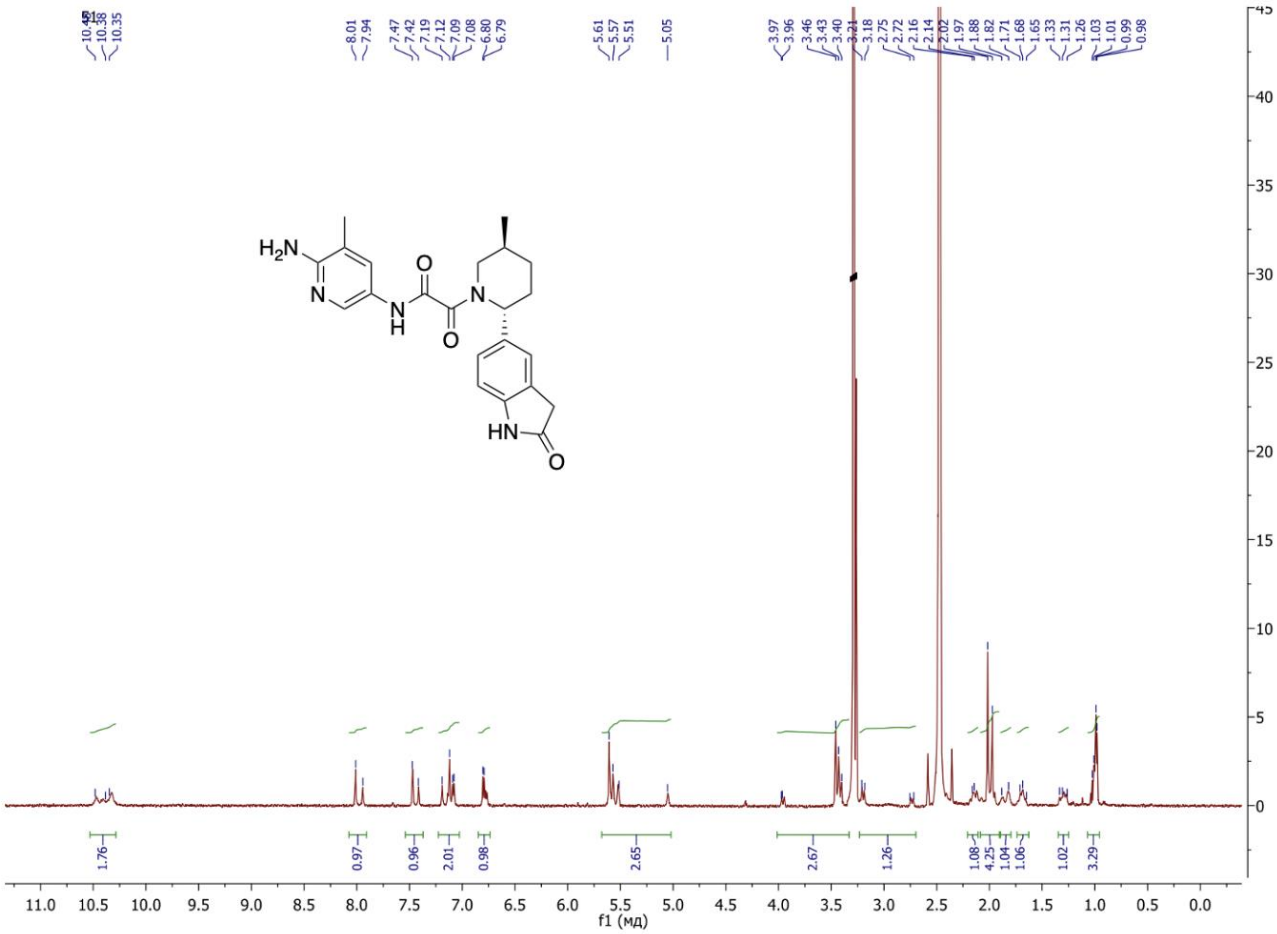

H2N
N
NH
O
O
HN
O
f1 (мд)

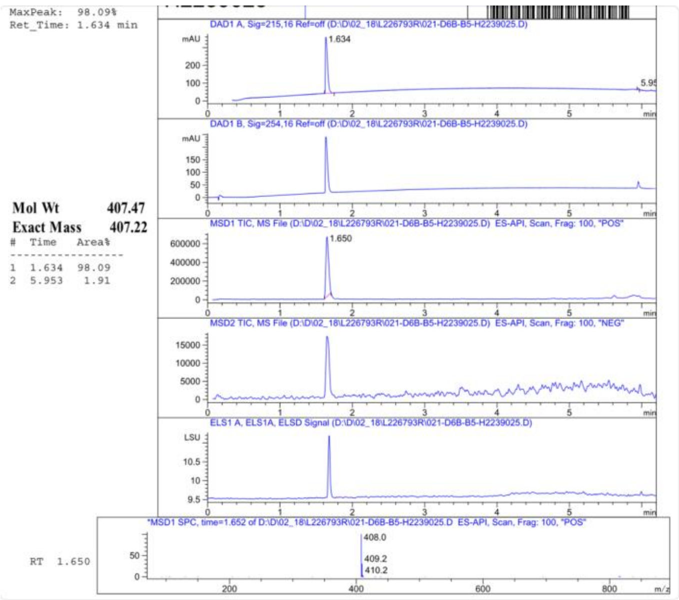

MaxPeak: 98.09%
Ret_Time: 1.634 min
Mol Wt 407.47
Exact Mass 407.22
Time Area%
1 1.634 98.09
2 5.953 1.91
RT 1.650
DAD1 A, Sig=215,16 Ref=off (D:\D\02_18\L226793R\021-D6B-B5-H2239025.D)
DAD1 B, Sig=254,16 Ref=off (D:\D\02_18\L226793R\021-D6B-B5-H2239025.D)
MSD1 TIC, MS File (D:\D\02_18\L226793R\021-D6B-B5-H2239025.D) ES-API, Scan, Frag: 100, "POS"
MSD2 TIC, MS File (D:\D\02_18\L226793R\021-D6B-B5-H2239025.D) ES-API, Scan, Frag: 100, "NEG"
ELS1 A, ELS1A, ELSD Signal (D:\D\02_18\L226793R\021-D6B-B5-H2239025.D)
*MSD1 SPC, time=1.652 of D:\D\02_18\L226793R\021-D6B-B5-H2239025.D ES-API, Scan, Frag: 100, "POS"
408.0
409.2
410.2

Compound 37

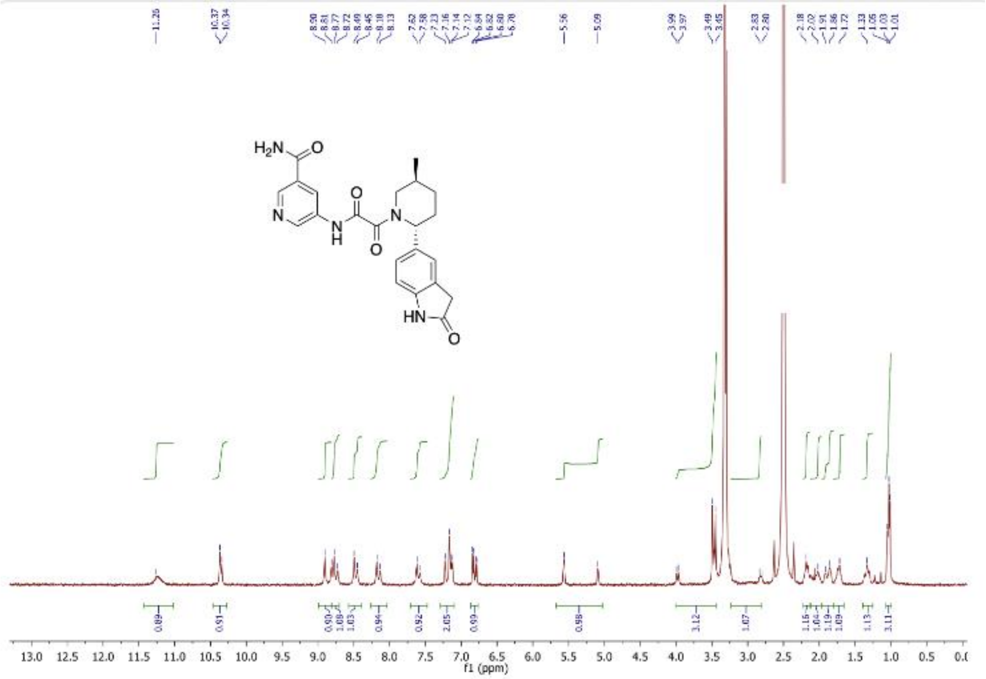

H_2N
O
N
H
O
O
N
HN
O
f1 (ppm)

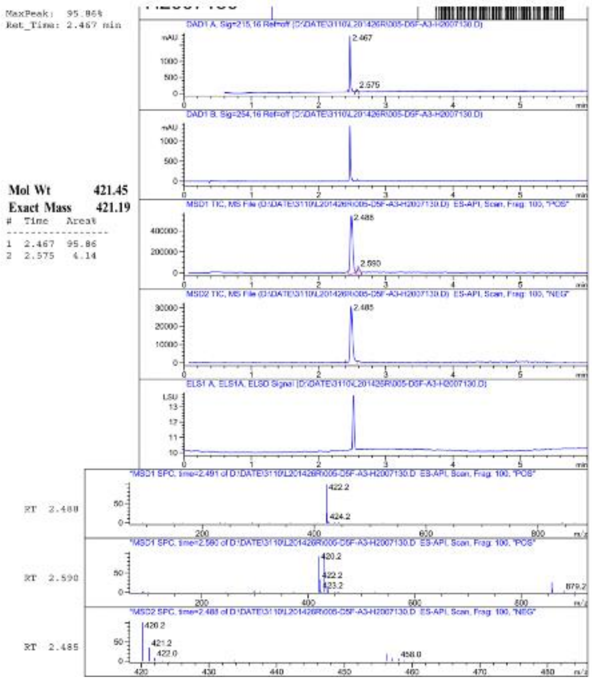

MaxPeak: 95.86%
Ret_Time: 2.467 min
Mol Wt 421.45
Exact Mass 421.19
Time Area%
1 2.467 95.86
2 2.575 4.14
RT 2.488
RT 2.590
RT 2.485

Compound 38

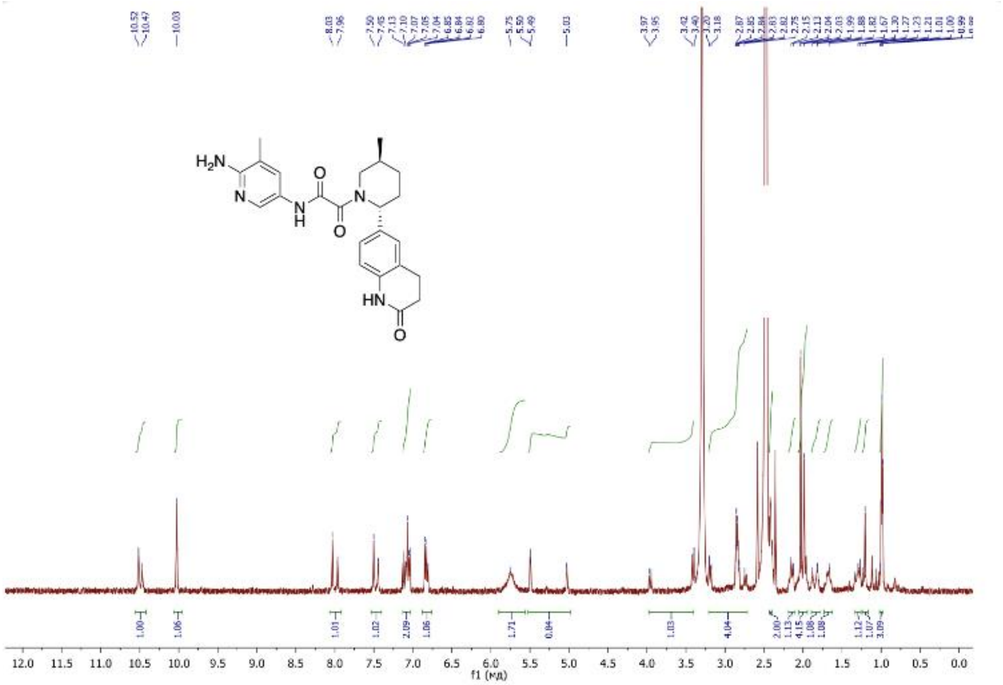

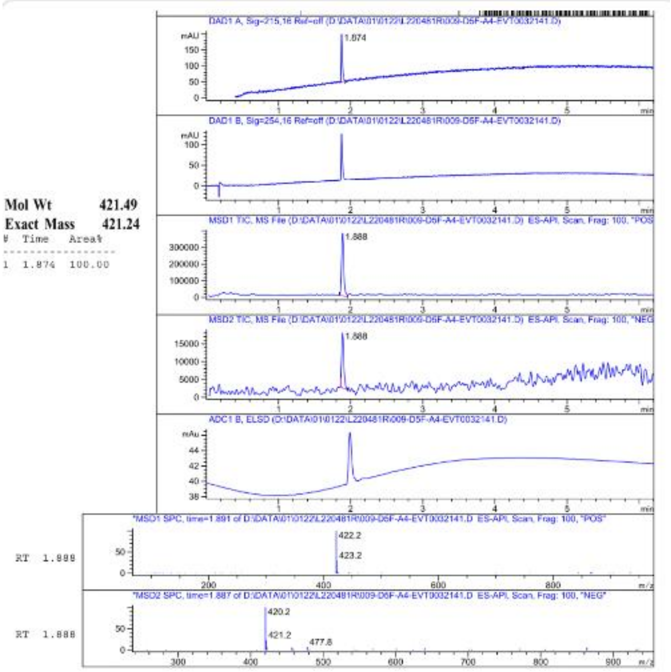

Mol Wt 421.49
Exact Mass 421.24
Time Area%
1 1.874 100.00
RT 1.888
RT 1.888

Compound 39

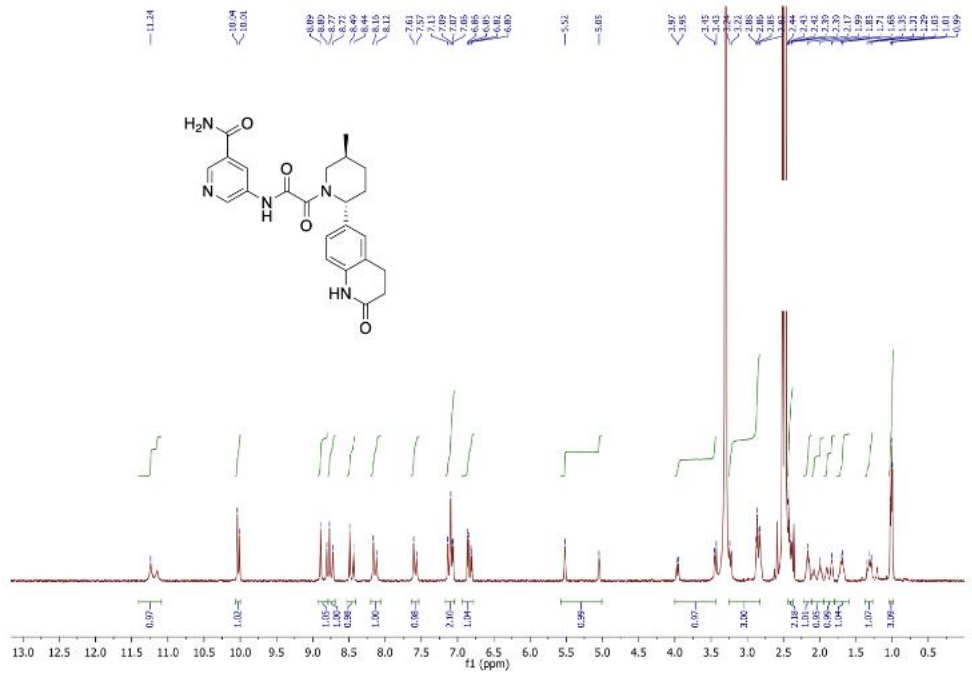

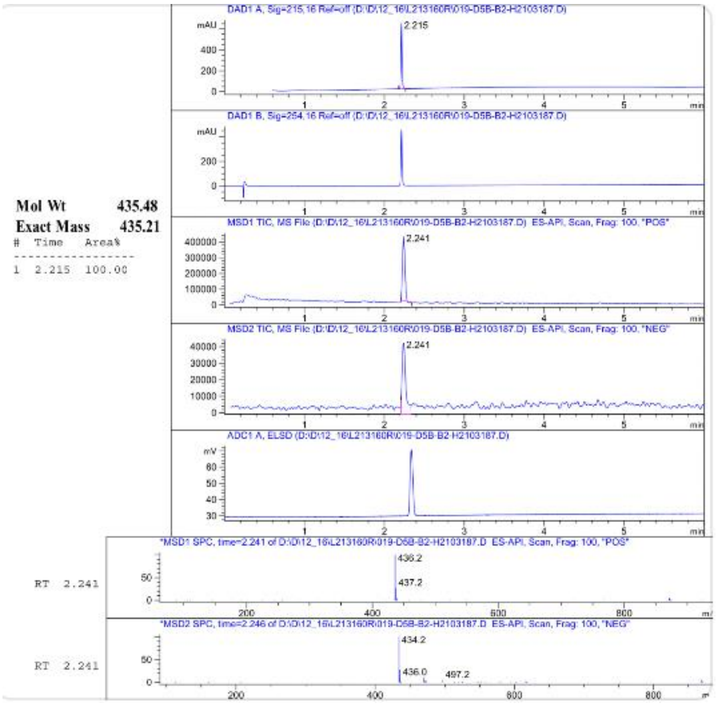
Mol Wt 435.48
Exact Mass 435.21
Time Area%
1 2.215 100.00
RT 2.241
RT 2.241

Compound 40

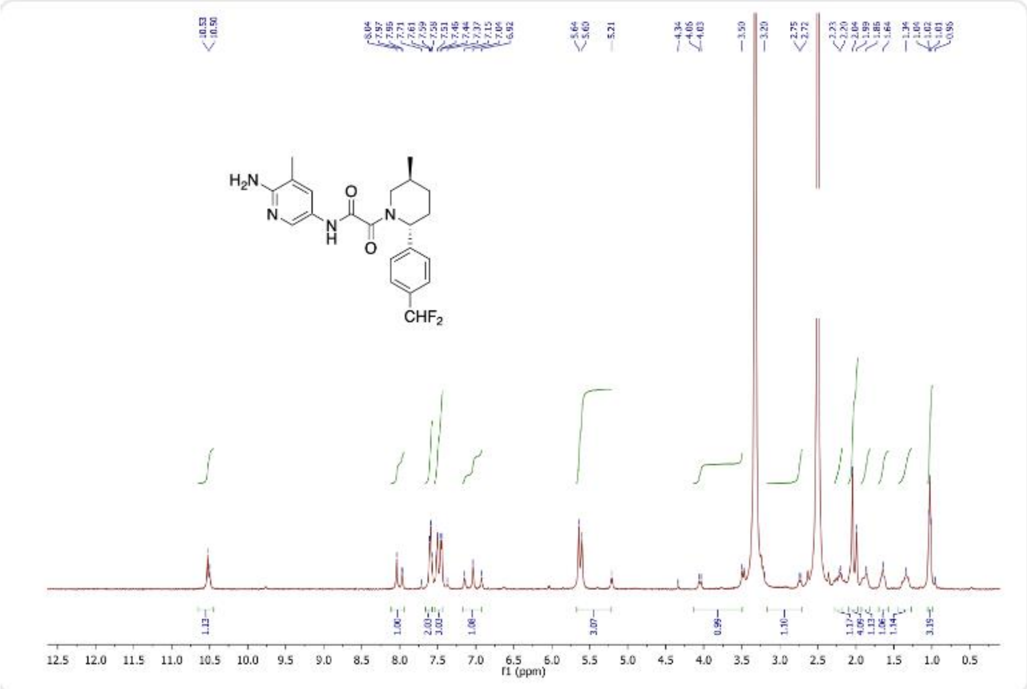

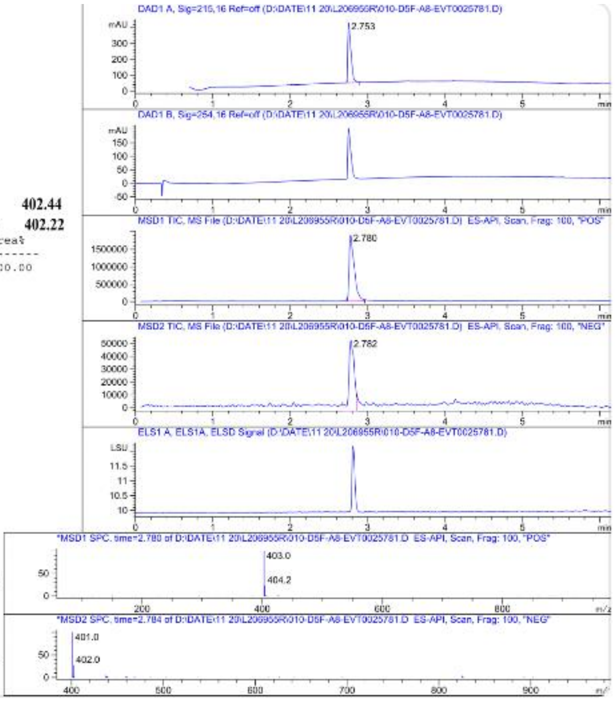

Mol Wt 402.44
Exact Mass 402.22

#	Time	Area%
1	2.753	100.00

RT 2.780

RT 2.782

Compound 41

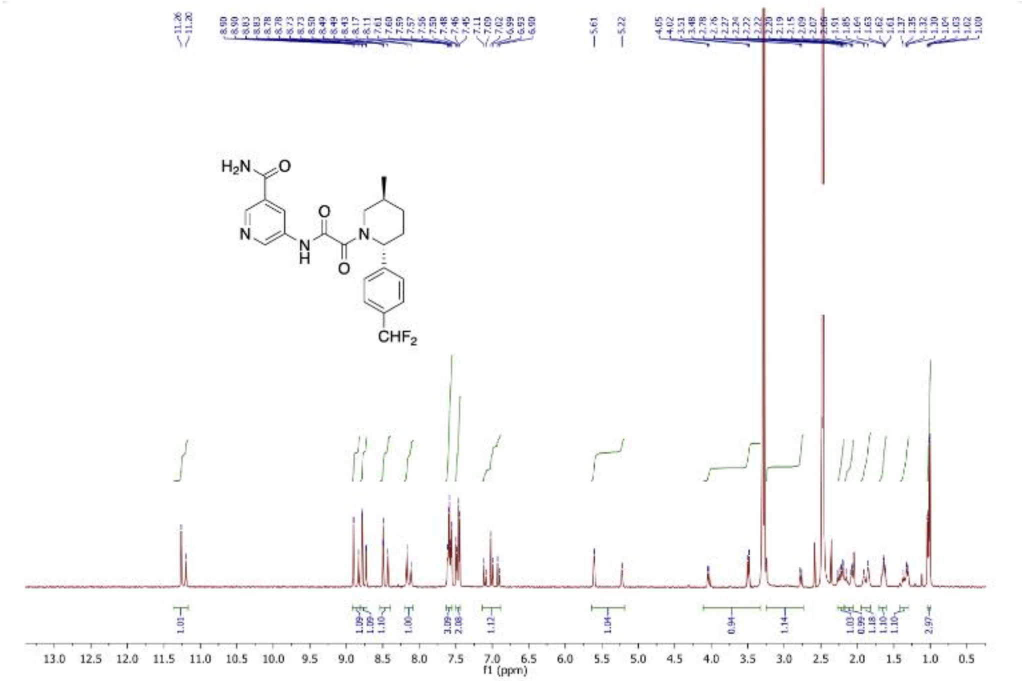

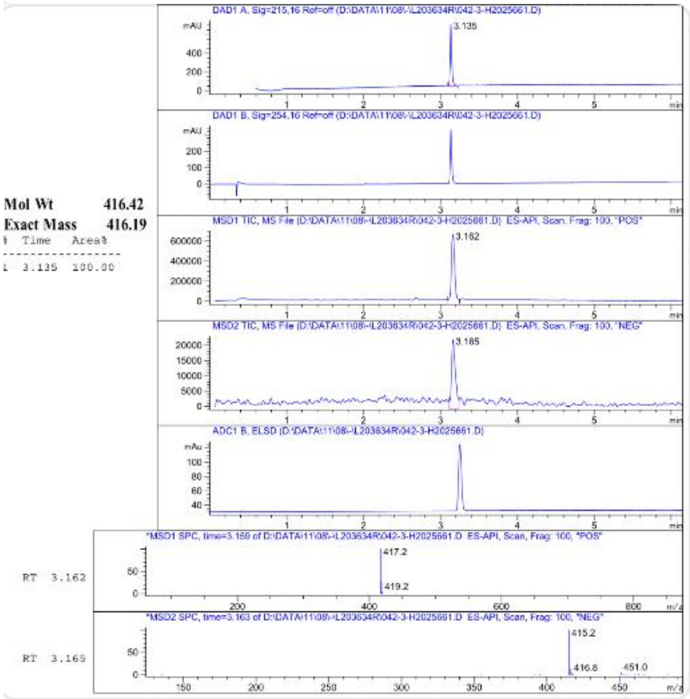

Compound 42

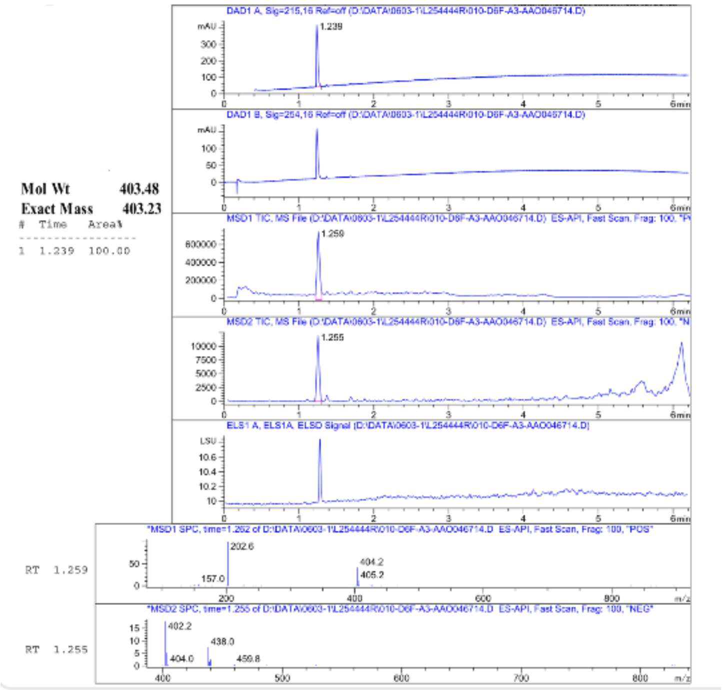

Mol Wt 403.48
Exact Mass 403.23
Time Area%
1 1.239 100.00
RT 1.259
RT 1.255

Compound 43

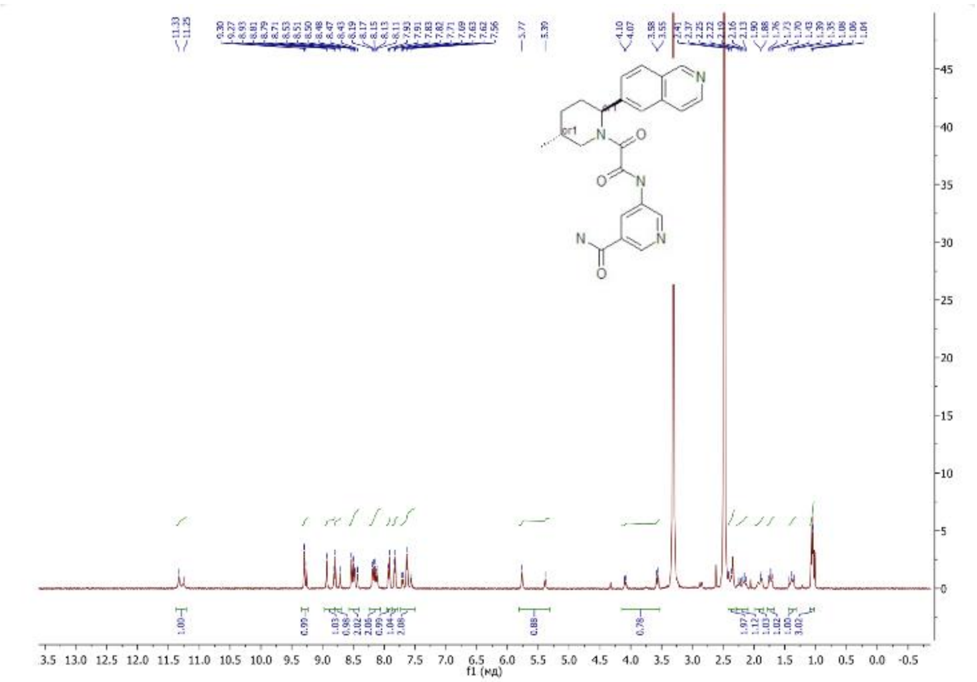

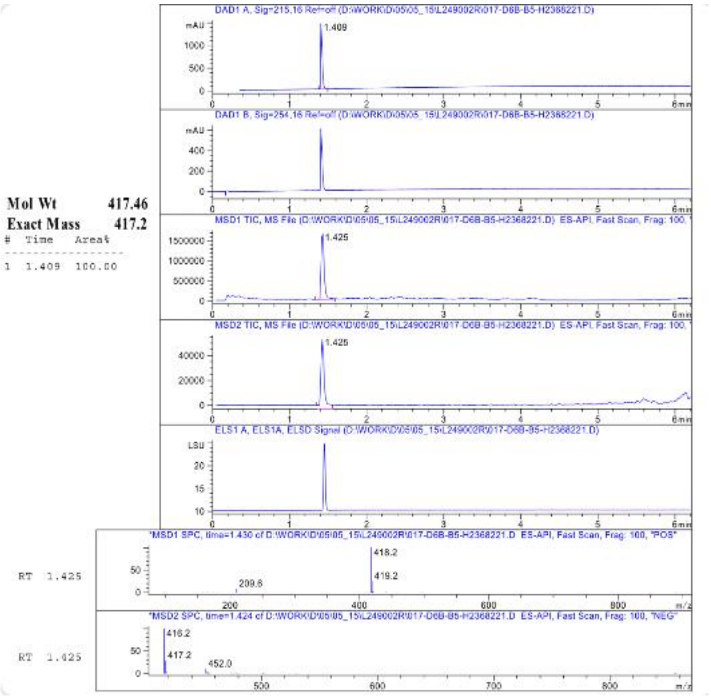

Mol Wt 417.46
Exact Mass 417.2
Time Area%
1 1.409 100.00
RT 1.425
RT 1.425

Compound 44

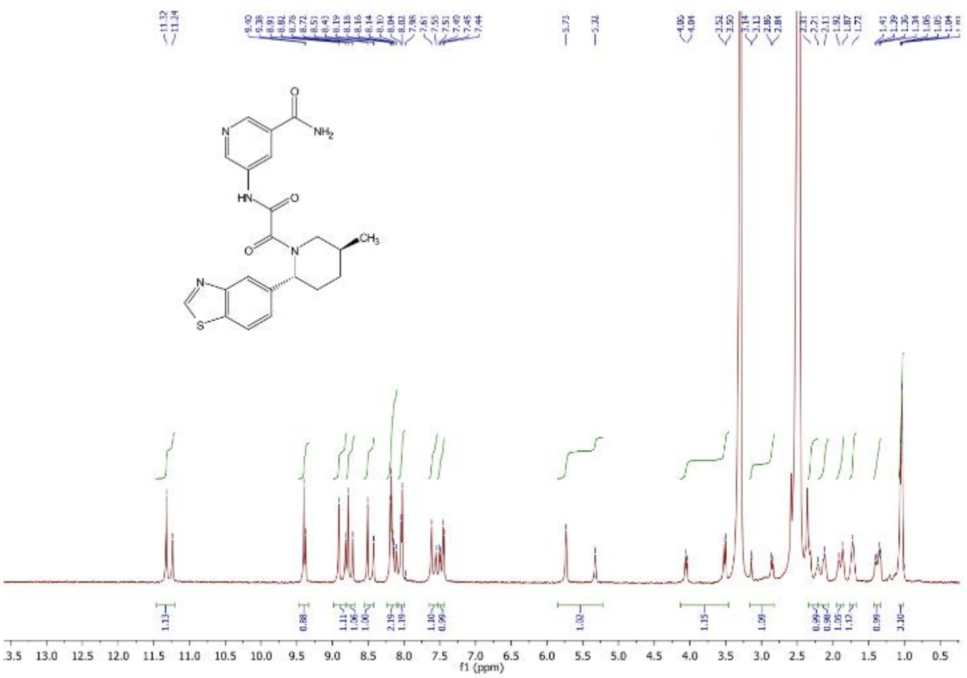

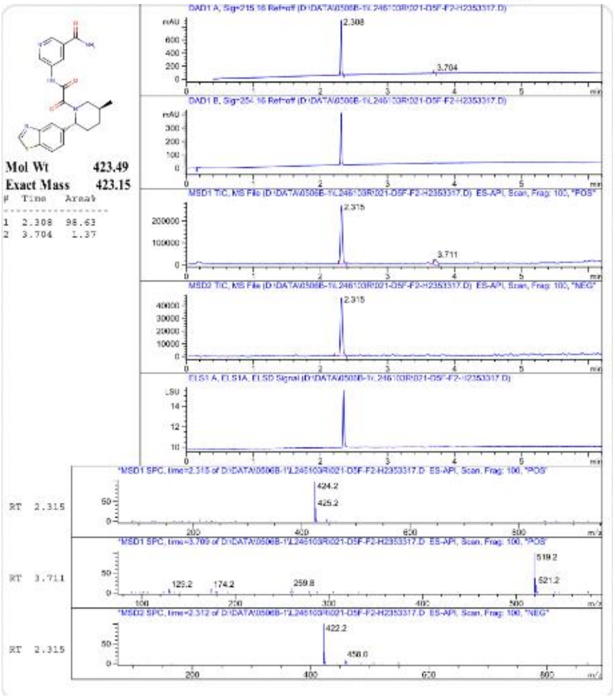

Compound 45

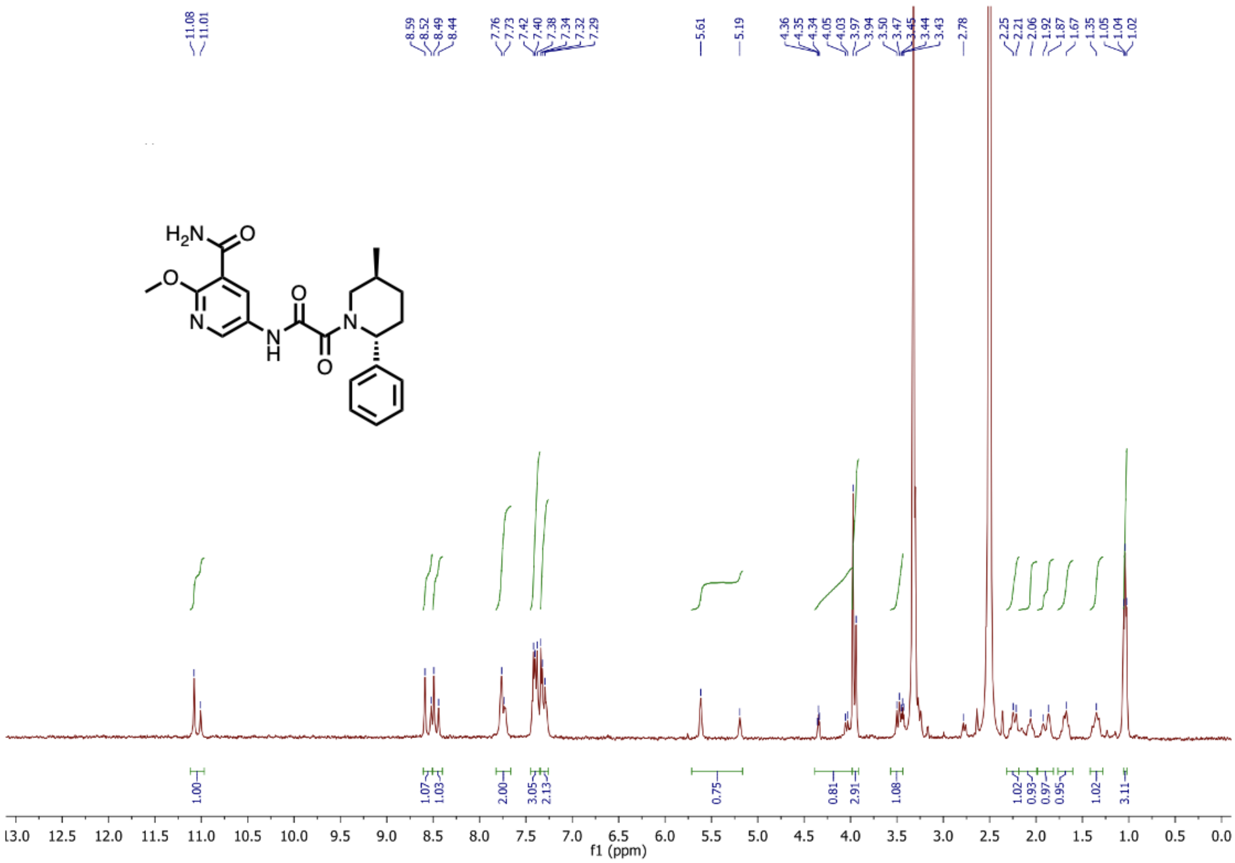

Mol Wt 396.44
Exact Mass 396.2

#	Time	Area%
1	3.345	100.00

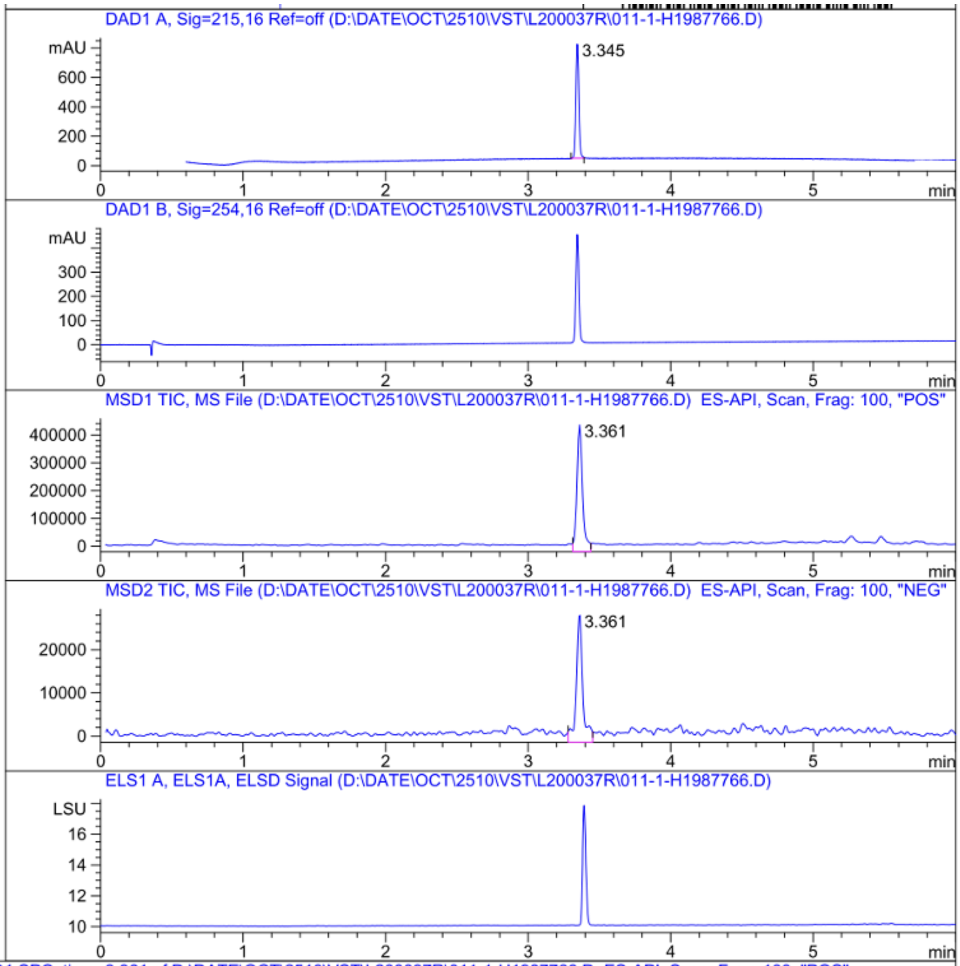

RT 3.361

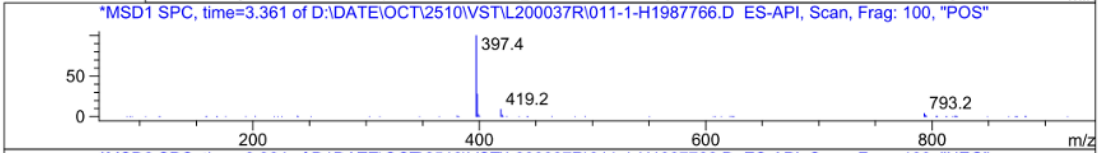

RT 3.361

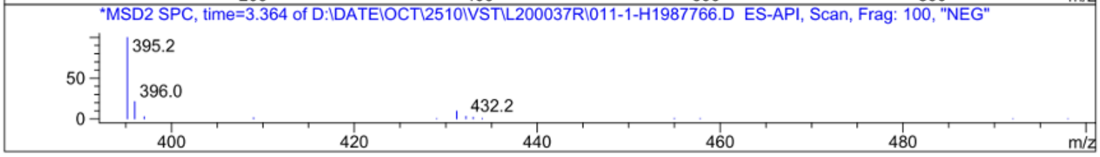

Compound 46

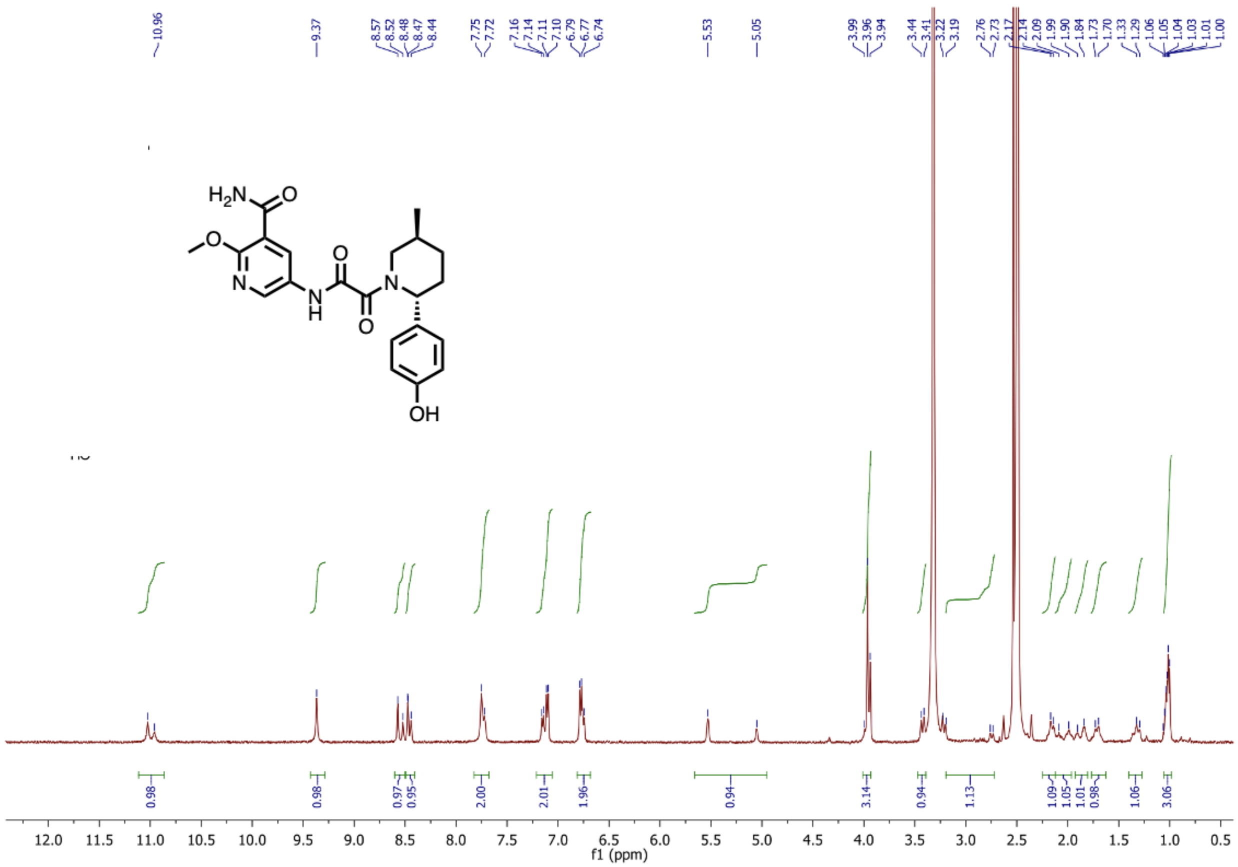

H2N
O
O
N
N
H
O
O
N
OH
f1 (ppm)

Mol Wt **412.44**
Exact Mass **412.19**

#	Time	Area%
1	2.840	100.00

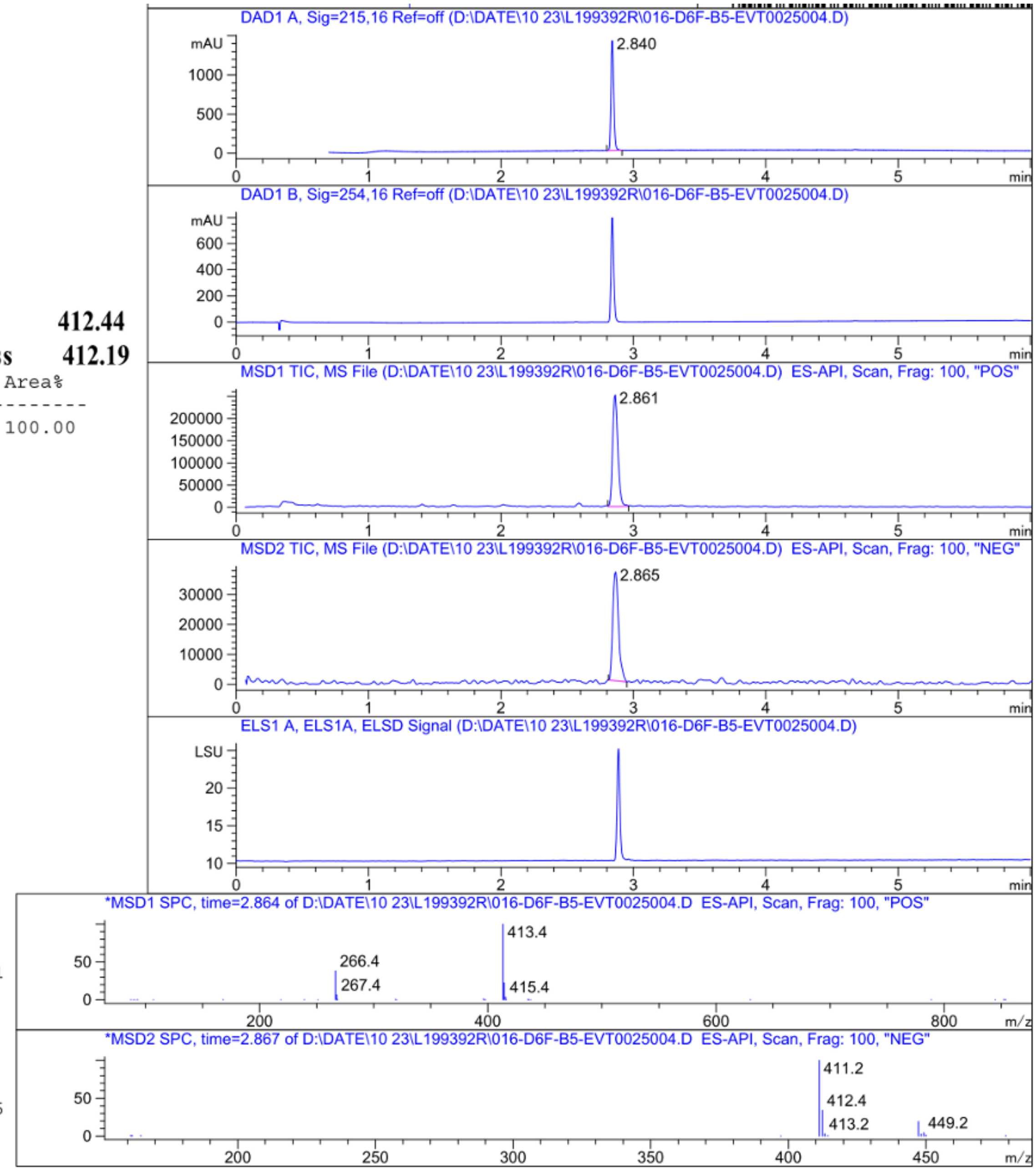

RT 2.861

RT 2.865

Compound 47

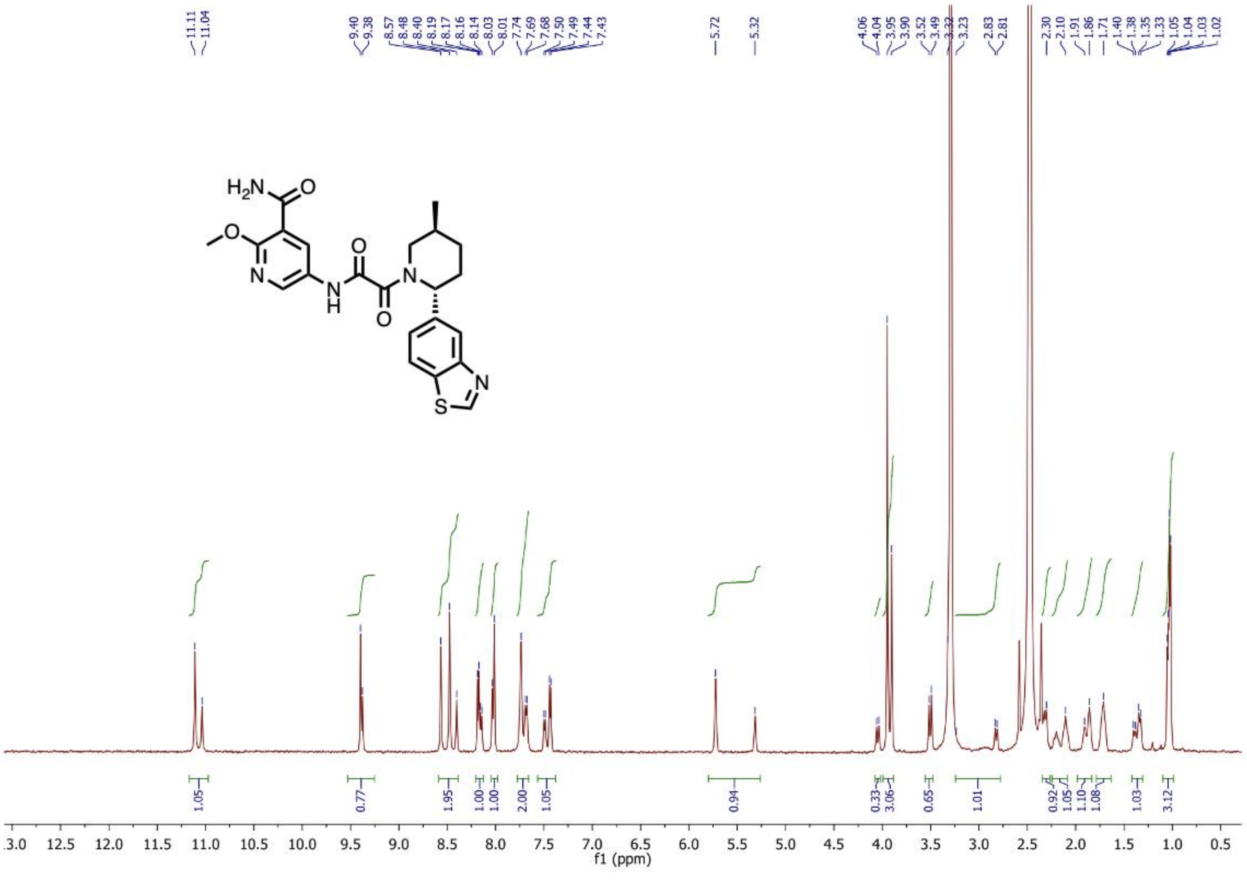

11.11
11.04
9.40
9.38
8.57
8.48
8.40
8.19
8.17
8.16
8.14
8.03
8.01
7.74
7.69
7.68
7.50
7.49
7.44
7.43
5.72
5.32
4.06
4.04
3.95
3.90
3.52
3.49
3.32
3.23
2.83
2.81
2.30
2.10
1.91
1.86
1.71
1.40
1.38
1.35
1.33
1.05
1.04
1.03
1.02
1.05
0.77
1.95
1.00
1.00
2.00
1.05
0.94
0.33
3.06
0.65
1.01
0.92
1.05
1.10
1.08
1.03
3.12
f1 (ppm)

Mol Wt 453.51
Exact Mass 453.16

#	Time	Area%
1	2.665	100.00

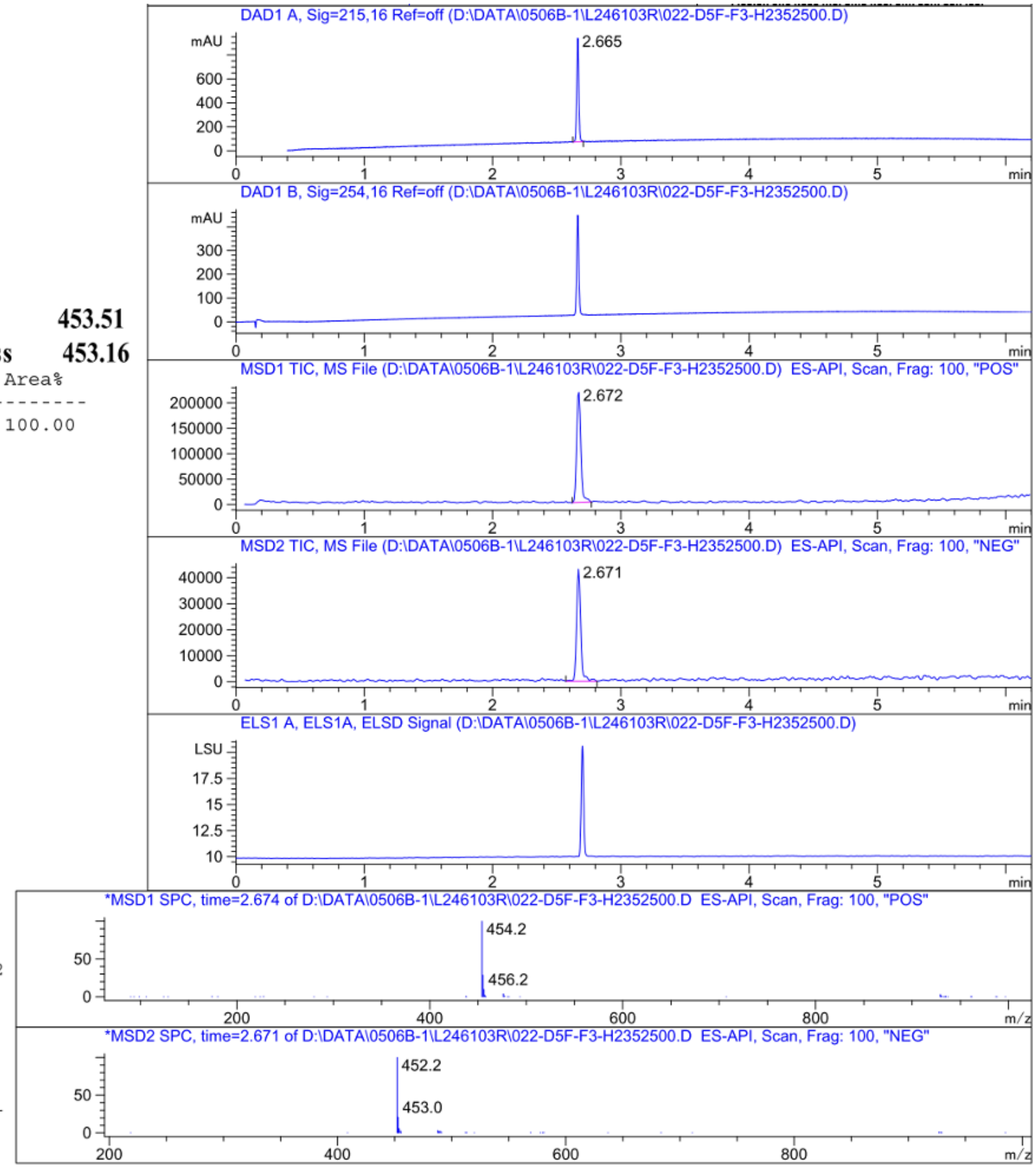

RT 2.672

RT 2.671

TNG908

Chiral HPLC – TNG908

Injection Volume: 5 mkl
Sample Info: IC, Hexane-IPA-MeOH, 50-25-25, 0.6ml/min

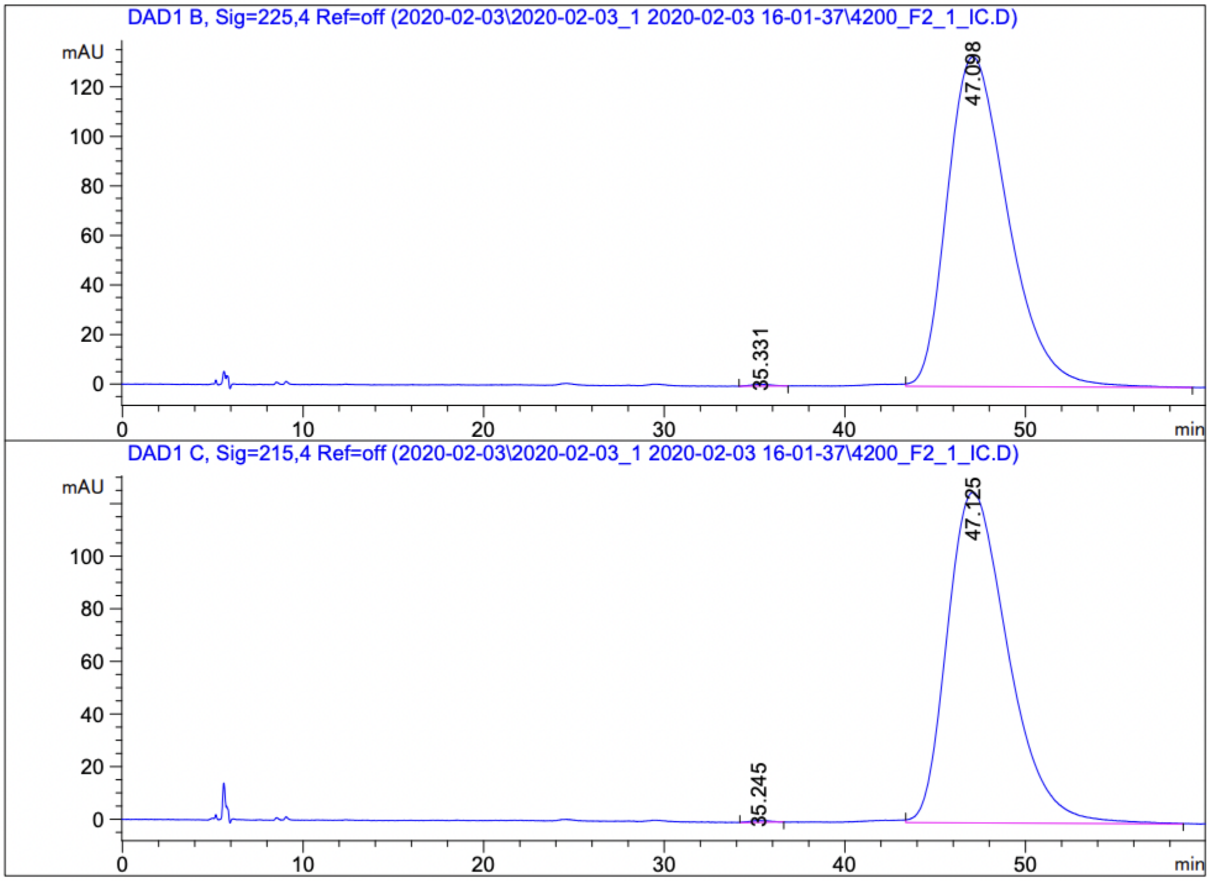

Signal: DAD1 B, Sig=225,4 Ref=off

RetTime(min)	Area,%	Symm.	Resolution	Selectivity
35.3312	0.19	0.000		
47.0982	99.81	0.000	2.86	1.33

Signal: DAD1 C, Sig=215,4 Ref=off

RetTime(min)	Area,%	Symm.	Resolution	Selectivity
35.2452	0.17	0.018		
47.1252	99.83	0.000	2.94	1.34

LCMS- TNG908

Sample 4200-F2 0.1% FA-ACN 5-95%ACN 6 min. Zorbax Eclipse-plus C18 4.6*100mm, 3.5 mkm.

#	Time	Area%
1	3.805	99.71
2	4.685	0.29

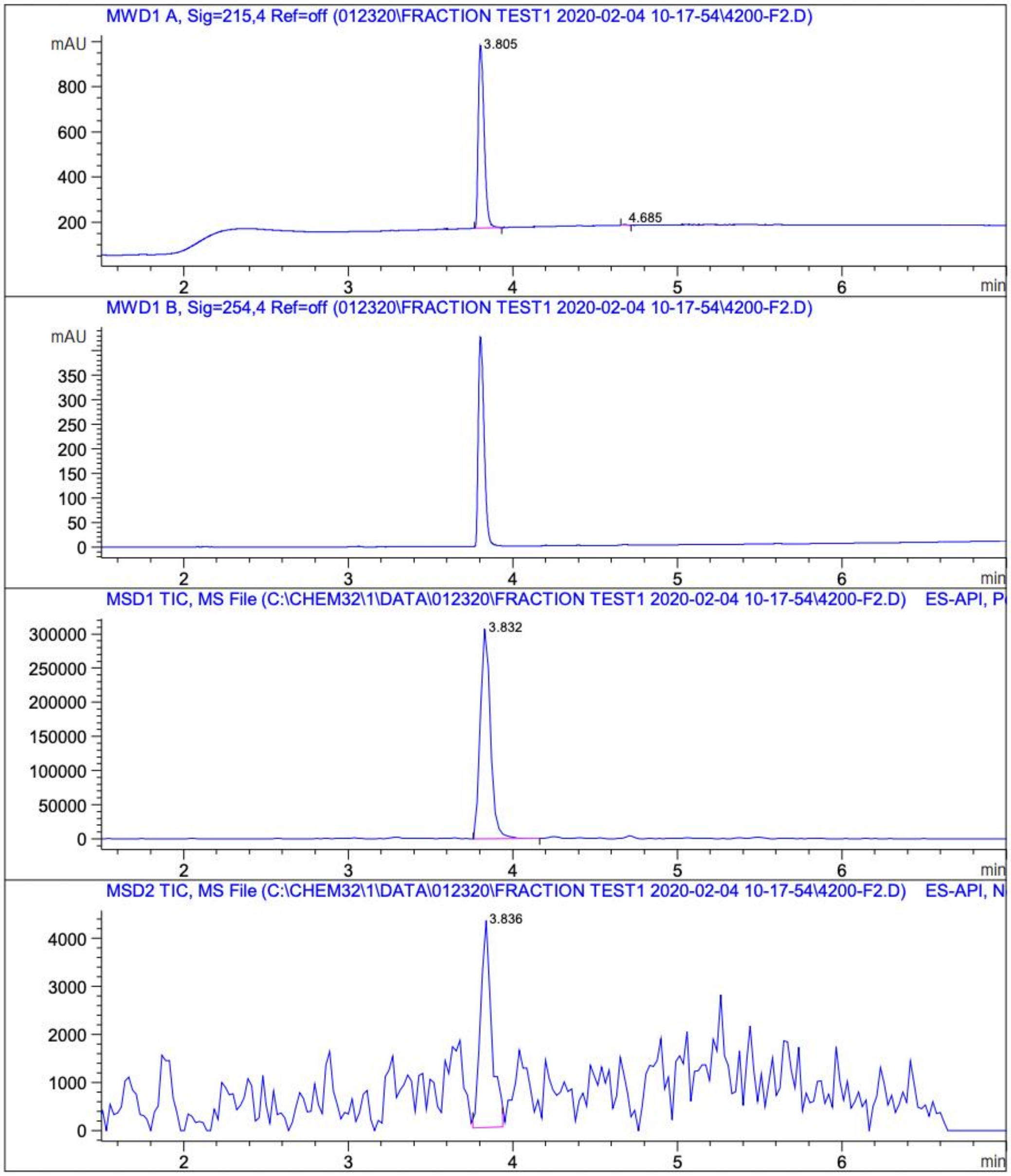

RT 3.832

RT 3.836

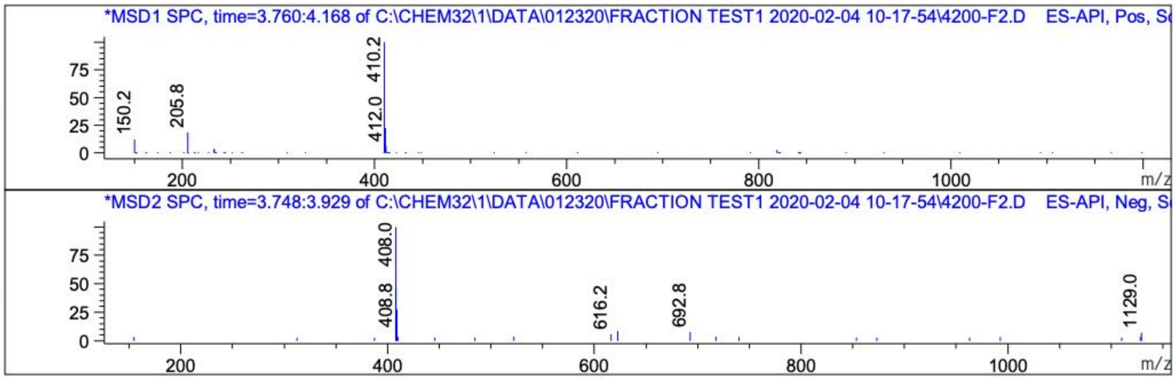

^{1}H NMR TNG908

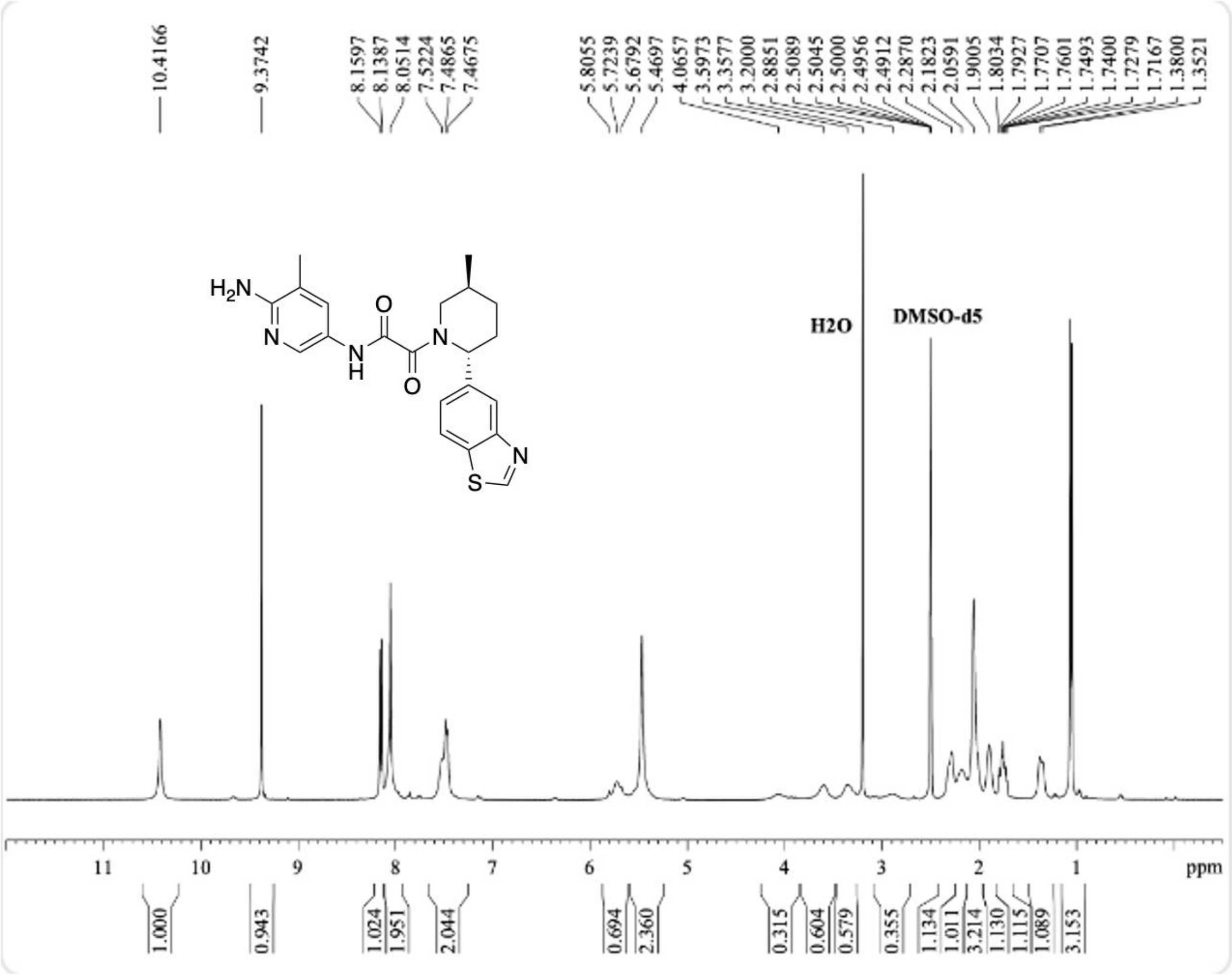

1H-1H COSY – TNG908

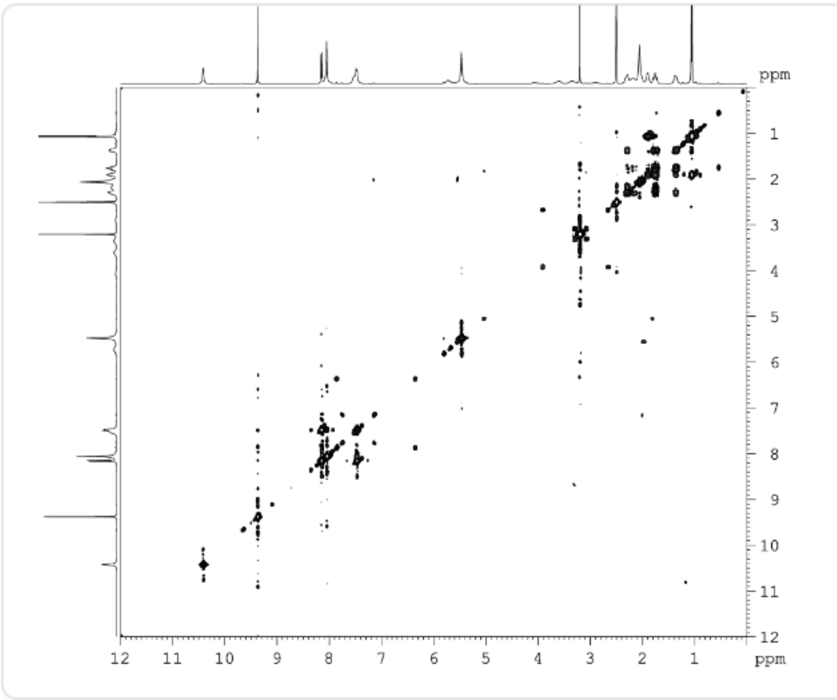

1H-1H ROESY-TNG908

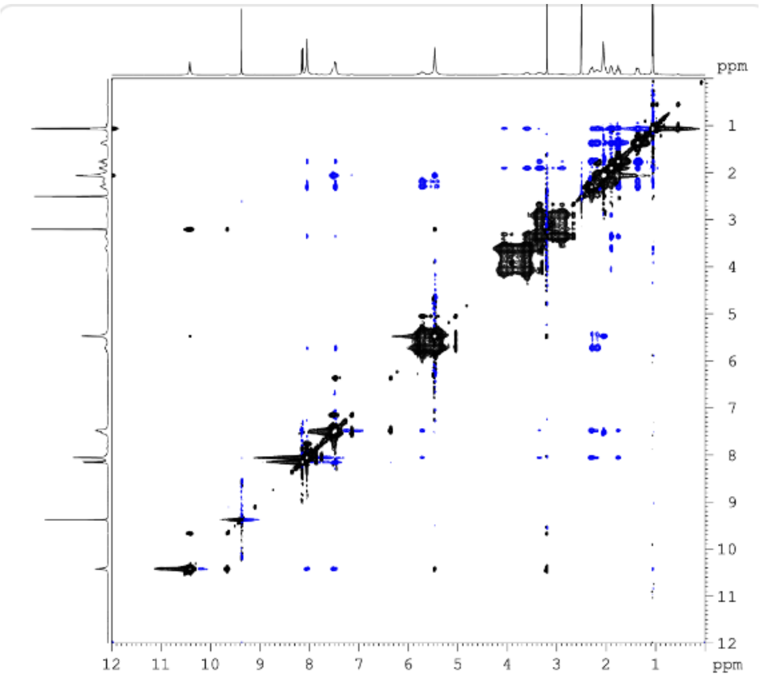

1H-13C HMBC -TNG908

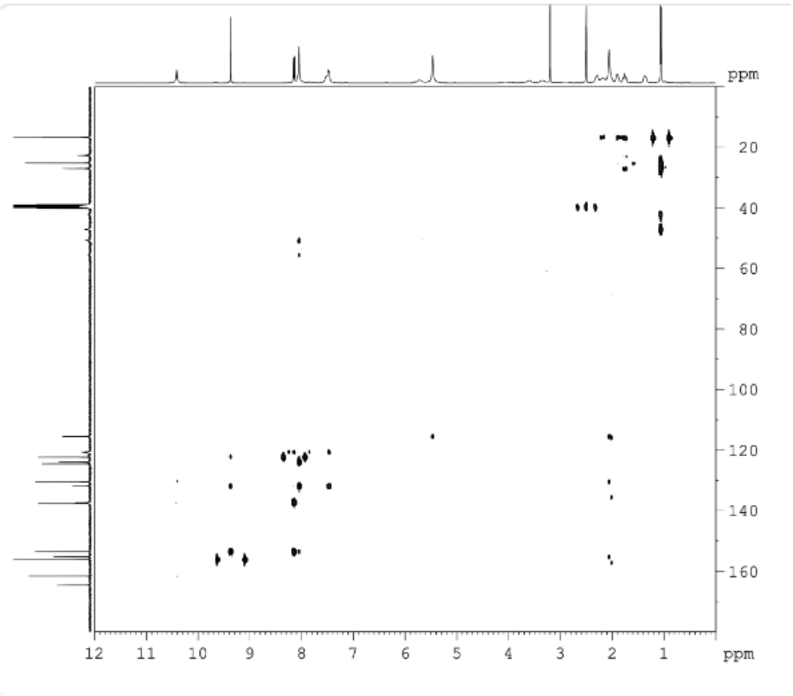

1H-13C HSQC-TNG908

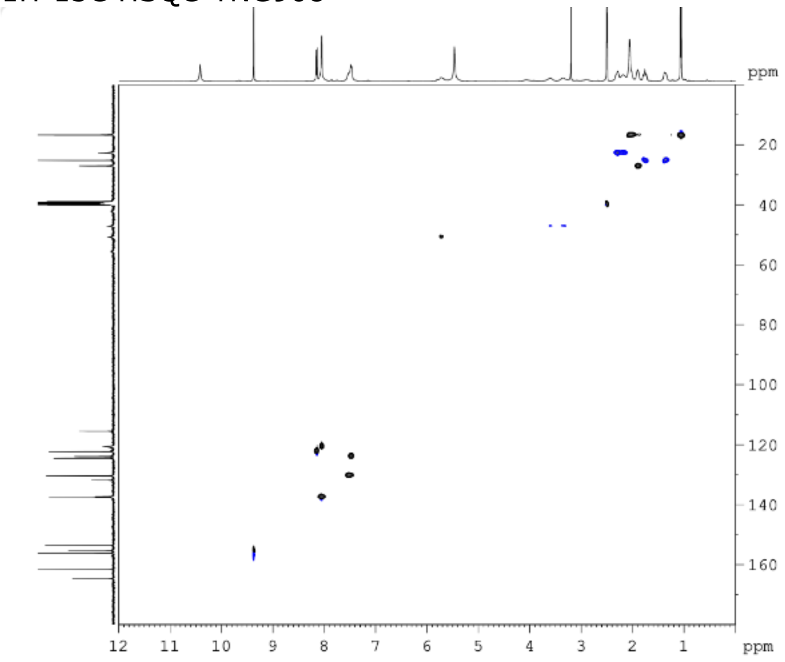

C13 NMR-TNG908

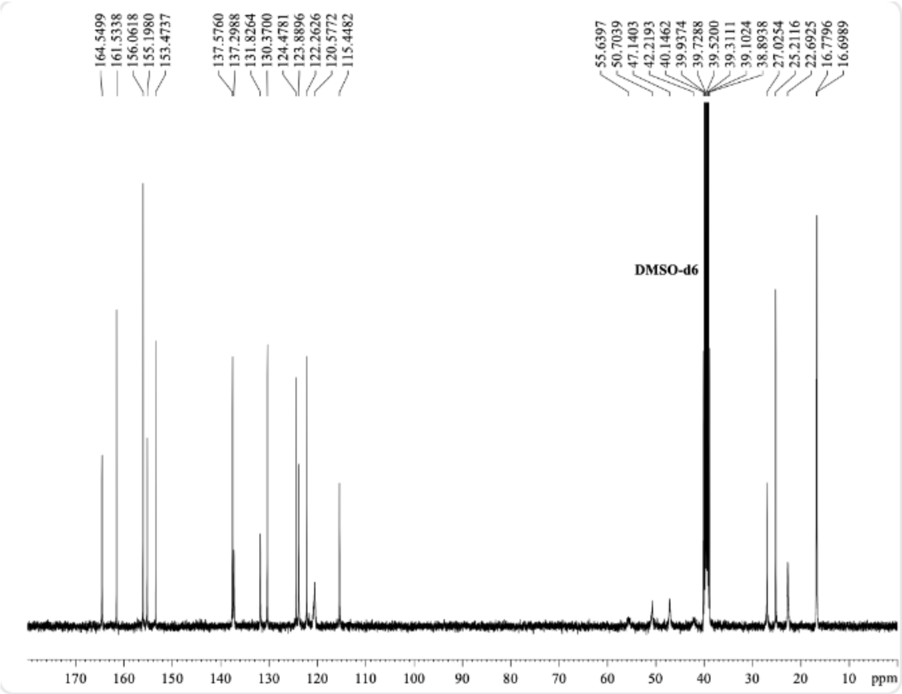

FT-IR – TNG908

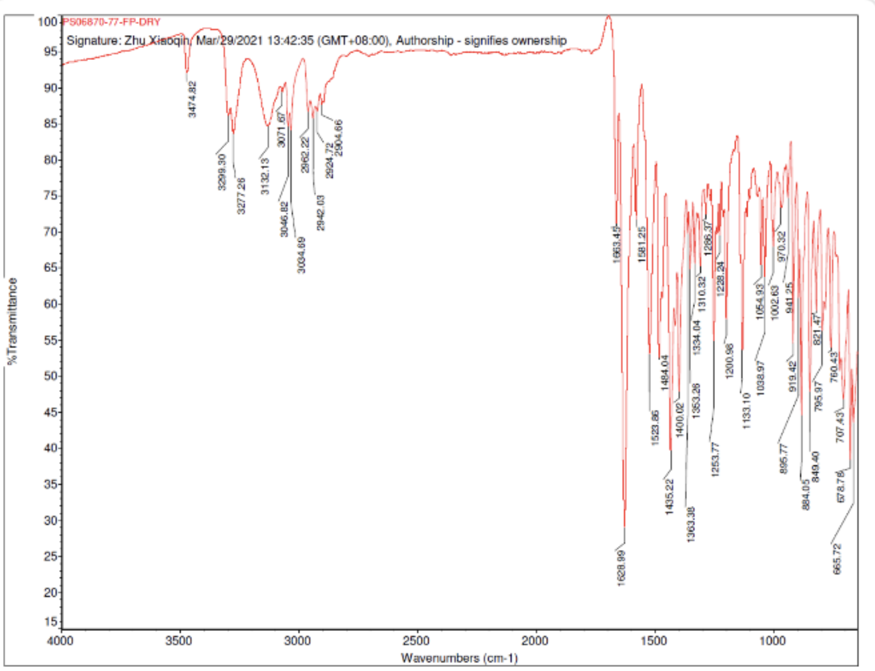

High resolution MS – TNG908

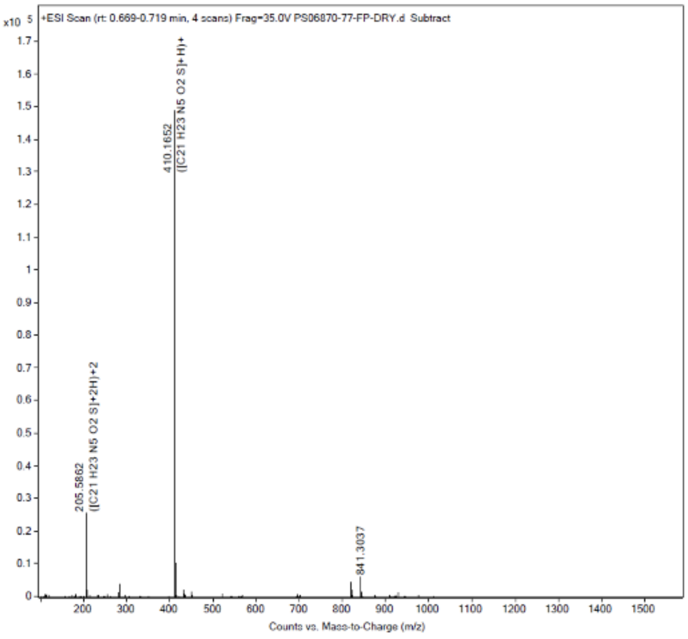

Carbon, hydrogen and nitrogen analysis of TNG908 drug substance was performed on an Elementar vario EL III instrument. The elemental analysis of TNG908 is presented in Table SI-1 and is consistent with its molecular formula, $C_{21}H_{23}N_5O_2S$.

Table SI-1. Elemental Analysis Results for TNG908

Analysis	Carbon (%)	Hydrogen (%)	Nitrogen (%)
Found value[a]	61.81	5.41	16.93
Theoretical value[b]	61.59	5.66	17.10
Absolute difference	0.2	0.3	0.2

[a]The found value was based on an average of 2 determinations.
[b]The theoretical value was based on a 100% pure sample. The presence of free water and residual water was not taken into consideration.

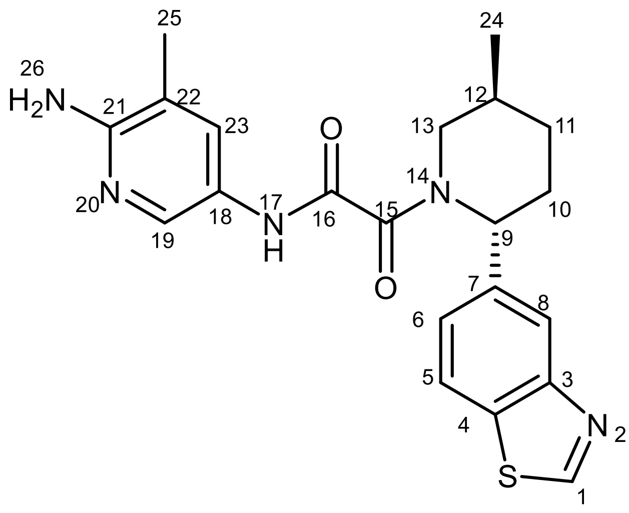

Table SI-2. ^{1}H, ^{13}C and 2D NMR Spectroscopic Data for TNG908 in DMSO-d_6

Number	δ_H (ppm) (mult, J in Hz)[a]	δ_C (ppm)	COSY	HMBC[b] (^{1}H-^{13}C)	ROESY
1	9.37 (s)	156.06	-	3, 4, 5	-
2(N)	-	-	-	-	-
3	-	153.47	-	-	-
4	-	131.83	-	-	-
5	8.15 (d, J=8.4 Hz)	122.26	6	3, 7	6
6	7.48 (ov)	123.89	5, 8	4, 8, 9	5, 9, 10a, 10b, 11a, 13b
7	-	137.30	-	-	-
8	8.05 (s)	120.58	6	4, 6, 9, 9'	9, 10a, 10b, 11a, 13b
9	5.72 (m)	50.70	-	-	6, 8, 10a, 10b
9’	5.53 (ov)	55.64	-	-	10a, 10b
10a	2.29 (br)	22.69	10b, 11a, 11b	-	6, 8, 9, 9', 10b, 11a, 11b, 24
10b	2.18 (br)		10a, 11a	-	6, 8, 9, 9', 10a, 11b, 24
11a	1.75 (m)	25.21	10a, 10b, 11b, 12	10, 12,24	6, 8, 10a, 11b, 13b, 24
11b	1.36 (m)		10a, 11a, 12	-	10a, 10b, 11a, 12, 24
12	1.90 (br)	27.03	11a, 11b, 24	24	11b, 13a, 13b, 13a', 13b', 24
13a	3.60 (br)	47.14	-	-	12, 24
13b	3.36 (br)		-	-	6, 8, 11a, 12
13a’	4.07 (br)	42.22	-	-	12
13b’	2.89 (br)		-	-	12
14(N)	-	-	-	-	-
15	-	164.55	-	-	-
16	-	161.53	-	-	-
17(NH)	10.42 (s)	-	-	16, 19, 23	-
18	-	124.48	-	-	-
19	8.05 (ov)	137.58	-	-	-
20(N)	-	-	-	-	-

21	-	155.20	-	-	-
22	-	115.45	-	-	-
23	7.52 (ov)	130.37	-	-	-
24	1.06 (d, *J*=7.0 Hz)	16.78	12	-	10a, 10b, 11a, 11b, 12, 13a
25	2.06 (s)	16.70	-	21, 22, 23	26
26	5.47 (s)	-	-	22	25

Abbreviations: NMR, nuclear magnetic resonance, DMSO, dimethyl sulfoxide; ppm, parts per million; COSY, correlation spectroscopy; HMBC, heteronuclear multiple bond correlation; ROESY, rotating-frame nuclear Overhauser effect correlation spectroscopy

Non-equivalent protons attached to the same atom are designed as "a" and "b".

'A rotamer exists in the solution and may appears at different chemical shifts in the 1H-NMR and 13C-NMR spectra.

a s=singlet, d=doublet, m=multiplet, dd=doublet of doublets, ov=overlapped, br=broad

b From proton to the indicated carbon

For any protonated carbon, assignment of either the proton or the carbon could be used to assign the other using the HSQC spectrum. Quaternary carbons were identified by comparison of the ^{13}C NMR spectrum with the HSQC spectrum, as only protonated carbons were observed in the HSQC spectrum. The pulse program used in the HSQC experiment has a function of multiplicity editing. In such kind of HSQC spectrum, black and blue dots indicate CH/CH_3 and CH_2, respectively.

H-24 was identified by chemical shift, integration and peak multiplicity.

H-25 was identified by chemical shift, integration and peak multiplicity.

H-17 was identified by chemical shift, integration, peak multiplicity and the absence of HSQC correlation.

C-16 was identified by chemical shift and an HMBC correlation from H-17.

H-26 was identified by chemical shift, integration, peak multiplicity and the absence of HSQC correlations.

H-9 and H-9' were identified by chemical shift, integration and peak multiplicity.

C-9 was identified by HSQC correlation from H-9. C-9' was identified by chemical shift.

C-22 was identified by an HMBC correlation from H-25 and H-26.

The protonated C-23 was identified by an HMBC correlation from H-25.

C-21 was identified by chemical shift and an HMBC correlation from H-25.

H-12 was identified by a COSY correlation from H-24.

H-11a and H-11b were identified by chemical shift and COSY correlations from H-12.

H-10a and H-10b were identified by chemical shift, COSY correlations from H-11a, and the two protons indicated to the same carbon in HSQC correlation.

H-13a, H-13b, H-13a' and H-13b' were identified by chemical shift and ROESY correlation from H-12.

H-8 was identified by chemical shift, peak multiplicity and HMBC correlations from H-9 and H-9'.

H-6 was identified by a COSY correlation from H-8.

H-5 was identified by a COSY correlation from H-6.

C-4 was identified by HMBC correlations from H-6 and H-8.

H-1 was identified by chemical shift and an HMBC correlation from C-4'.

C-3 was identified by HMBC correlations from H-1 and H-5.

C-7 was identified by an HMBC correlation from H-5.

C-19 was identified by chemical shift and an HMBC correlation from H-17.

The remaining carbons were C-15 and C-18, they were distinguished by chemical shift.

Small molecule crystal structure of TNG908

The single crystal X-ray diffraction studies were carried out on a Bruker Kappa Photon II CPAD diffractometer equipped with Cu $_{K\alpha}$ radiation (λ = 1.54178). Crystals of **TNG908** were grown by dissolving approximately 1mg of sample in 350µL of heated ACN that was allowed to slowly cool and sit undisturbed over several weeks. A 0.472 x 0.074 x 0.060 mm colorless block was mounted on a Cryoloop with Paratone oil. Data were collected in a nitrogen gas stream at 100(2) K using φ and ω scans. Crystal-to-detector distance was 60 mm using variable exposure time (10s-60s) depending on θ with a scan width of 1.0°. Data collection was 99.8% complete to 68.00° in θ. A total of 21266 reflections were collected covering the indices, -6<=h<=5, -17<=k<=17, -17<=l<=17. 7481 reflections were found to be symmetry independent, with a R_{int} of 0.0237. Indexing and unit cell refinement indicated a primitive, triclinic lattice. The space group was found to be P1. The data were integrated using the Bruker SAINT software program and scaled using the SADABS software program. Solution by direct methods (SHELXT) produced a complete phasing model for refinement. All nonhydrogen atoms were refined anisotropically by full-matrix least-squares (SHELXL-2014). All carbon bonded hydrogen atoms were placed using a riding model. Their positions were constrained relative to their parent atom using the appropriate HFIX command in SHELXL-2014. All other hydrogen atoms (H-bonding) were located in the difference map. Their relative positions were restrained using DFIX commands and their thermals freely refined. The absolute stereochemistry of the molecule was established by anomalous dispersion using the Parson's method with a Flack parameter of -0.004(8). Crystallographic data are summarized in Table SI-3-SI-8.

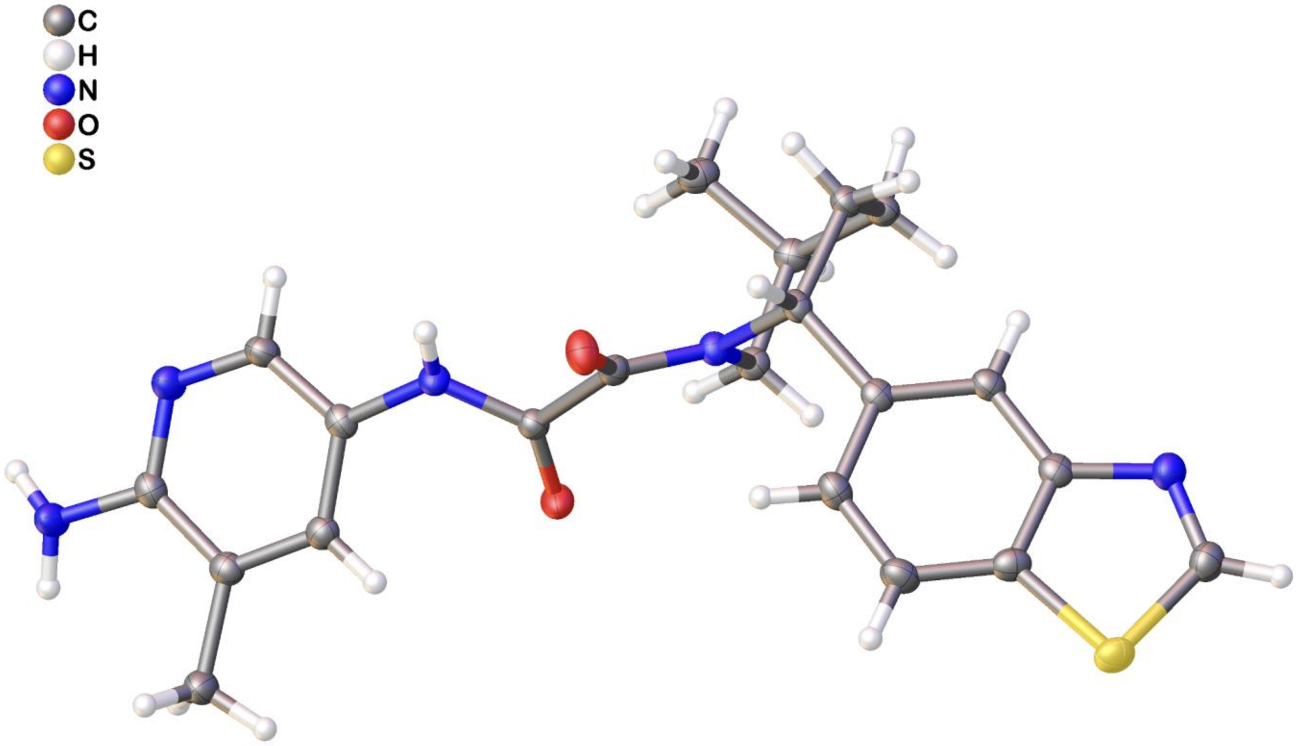

Figure SI-1. Small molecule crystal structure of TNG908.

Table SI-3. Small molecule crystal structure parameters, TNG908.

Empirical formula	C21 H23 N5 O2 S
Molecular formula	C21 H23 N5 O2 S
Formula weight	409.50
Temperature	100.0 K
Wavelength	1.54178 Å
Crystal system	Triclinic
Space group	P1
Unit cell dimensions	a = 5.0705(2) Å α = 87.522(2)°
	b = 13.9249(7) Å β = 85.730(2)°
	c = 13.9877(7) Å γ = 81.474(2)°
Volume	973.47(8) $Å^3$
Z	2
Density (calculated)	1.397 Mg/m^3
Absorption coefficient	1.715 mm^{-1}
F(000)	432
Crystal size	0.472 x 0.074 x 0.06 mm^3
Crystal color, habit	Colorless Needle
Theta range for data collection	3.170 to 74.689°.
Index ranges	-6<=h<=5, -17<=k<=17, -17<=l<=17
Reflections collected	21266
Independent reflections	7481 [R(int) = 0.0237, R(sigma) = 0.0250]
Completeness to theta = 68.000°	99.8 %
Absorption correction	Semi-empirical from equivalents
Max. and min. transmission	0.3260 and 0.2254
Refinement method	Full-matrix least-squares on F^2
Data / restraints / parameters	7481 / 3 / 551
Goodness-of-fit on F^2	1.026
Final R indices [I>2sigma(I)]	R1 = 0.0292, wR2 = 0.0765
R indices (all data)	R1 = 0.0298, wR2 = 0.0771
Absolute structure parameter	-0.004(8)
Extinction coefficient	n/a
Largest diff. peak and hole	0.191 and -0.183 e.$Å^{-3}$

Table SI-4. Atomic coordinates (x 10^4) and equivalent isotropic displacement parameters ($Å^2$x 10^3) for TNG908. U(eq) is defined as one third of the trace of the orthogonalized U^{ij} tensor.

	x	y	z	U(eq)
S(1)	13406(1)	10837(1)	-2955(1)	25(1)
O(1)	3009(4)	8449(1)	128(1)	24(1)
O(2)	8306(4)	7342(1)	1301(1)	23(1)
N(1)	6527(4)	7563(2)	-694(1)	17(1)
N(2)	12562(5)	9392(2)	-3941(2)	23(1)
N(3)	3942(4)	7173(2)	1672(2)	18(1)

N(4)	2547(5)	5929(2)	3983(2)	22(1)
N(5)	3856(5)	6060(2)	5513(2)	25(1)
C(1)	8873(5)	6806(2)	-737(2)	18(1)
C(2)	8428(5)	5984(2)	-1377(2)	20(1)
C(3)	7851(5)	6405(2)	-2382(2)	20(1)
C(4)	5523(5)	7245(2)	-2342(2)	18(1)
C(5)	5812(5)	8030(2)	-1632(2)	17(1)
C(6)	7784(5)	8727(2)	-1962(2)	18(1)
C(7)	9239(5)	8680(2)	-2843(2)	18(1)
C(8)	10946(5)	9371(2)	-3090(2)	19(1)
C(9)	11143(5)	10106(2)	-2454(2)	22(1)
C(10)	9693(6)	10159(2)	-1567(2)	24(1)
C(11)	8037(6)	9472(2)	-1337(2)	22(1)
C(12)	6222(5)	5430(2)	-937(2)	24(1)
C(13)	13907(6)	10108(2)	-3950(2)	24(1)
C(14)	5044(5)	7845(2)	110(2)	17(1)
C(15)	5987(5)	7411(2)	1087(2)	17(1)
C(16)	4170(5)	6869(2)	2653(2)	18(1)
C(17)	5791(5)	7268(2)	3250(2)	20(1)
C(18)	5764(5)	7006(2)	4211(2)	20(1)
C(19)	4080(5)	6323(2)	4556(2)	20(1)
C(20)	2601(5)	6213(2)	3053(2)	20(1)
C(21)	7366(6)	7455(2)	4889(2)	25(1)
S(1')	11083(1)	-836(1)	12802(1)	22(1)
O(1')	-575(4)	1852(1)	9942(1)	20(1)
O(2')	4081(4)	3085(2)	8680(1)	26(1)
N(1')	2428(4)	2549(1)	10697(1)	17(1)
N(2')	8196(5)	388(2)	13947(2)	25(1)
N(3')	-219(4)	2965(2)	8388(2)	19(1)
N(4')	953(5)	4364(2)	6122(2)	22(1)
N(5')	-763(5)	4366(2)	4639(2)	26(1)
C(1')	4303(5)	3251(2)	10764(2)	18(1)
C(2')	3094(5)	4051(2)	11455(2)	19(1)
C(3')	2413(5)	3591(2)	12437(2)	21(1)
C(4')	620(5)	2808(2)	12362(2)	20(1)
C(5')	1730(5)	2044(2)	11621(2)	18(1)
C(6')	4131(5)	1319(2)	11906(2)	19(1)
C(7')	4982(5)	1236(2)	12830(2)	20(1)
C(8')	7146(5)	536(2)	13048(2)	21(1)
C(9')	8447(5)	-72(2)	12331(2)	20(1)
C(10')	7596(5)	-10(2)	11400(2)	22(1)
C(11')	5446(5)	682(2)	11204(2)	21(1)
C(12')	695(5)	4675(2)	11026(2)	24(1)
C(13')	10225(6)	-303(2)	13904(2)	24(1)

C(14')	1153(5)	2387(2)	9921(2)	17(1)
C(15')	1875(5)	2869(2)	8933(2)	18(1)
C(16')	-301(5)	3313(2)	7420(2)	18(1)
C(17')	-1992(5)	2939(2)	6830(2)	20(1)
C(18')	-2215(5)	3266(2)	5894(2)	20(1)
C(19')	-690(5)	4009(2)	5566(2)	21(1)
C(20')	1164(5)	4014(2)	7028(2)	21(1)
C(21')	-3903(6)	2848(2)	5222(2)	26(1)

Table SI-5. Bond lengths [Å] and angles [°] for TNG908.

S(1)-C(9)	1.734(3)	C(7)-C(8)	1.404(4)
S(1)-C(13)	1.739(3)	C(8)-C(9)	1.403(4)
O(1)-C(14)	1.230(3)	C(9)-C(10)	1.393(4)
O(2)-C(15)	1.224(3)	C(10)-H(10)	0.9500
N(1)-C(1)	1.467(3)	C(10)-C(11)	1.377(4)
N(1)-C(5)	1.484(3)	C(11)-H(11)	0.9500
N(1)-C(14)	1.346(3)	C(12)-H(12A)	0.9800
N(2)-C(8)	1.396(3)	C(12)-H(12B)	0.9800
N(2)-C(13)	1.288(4)	C(12)-H(12C)	0.9800
N(3)-H(3)	0.82(4)	C(13)-H(13)	0.9500
N(3)-C(15)	1.344(3)	C(14)-C(15)	1.549(3)
N(3)-C(16)	1.426(3)	C(16)-C(17)	1.400(4)
N(4)-C(19)	1.343(3)	C(16)-C(20)	1.373(4)
N(4)-C(20)	1.343(3)	C(17)-H(17)	0.9500
N(5)-H(5A)	0.89(4)	C(17)-C(18)	1.377(4)
N(5)-H(5B)	0.83(4)	C(18)-C(19)	1.416(4)
N(5)-C(19)	1.373(3)	C(18)-C(21)	1.505(4)
C(1)-H(1A)	0.9900	C(20)-H(20)	0.9500
C(1)-H(1B)	0.9900	C(21)-H(21A)	0.9800
C(1)-C(2)	1.536(3)	C(21)-H(21B)	0.9800
C(2)-H(2)	1.0000	C(21)-H(21C)	0.9800
C(2)-C(3)	1.532(3)	S(1')-C(9')	1.732(3)
C(2)-C(12)	1.529(3)	S(1')-C(13')	1.739(3)
C(3)-H(3A)	0.9900	O(1')-C(14')	1.229(3)
C(3)-H(3B)	0.9900	O(2')-C(15')	1.224(3)
C(3)-C(4)	1.533(3)	N(1')-C(1')	1.471(3)
C(4)-H(4A)	0.9900	N(1')-C(5')	1.489(3)
C(4)-H(4B)	0.9900	N(1')-C(14')	1.348(3)
C(4)-C(5)	1.538(3)	N(2')-C(8')	1.397(3)
C(5)-H(5)	1.0000	N(2')-C(13')	1.300(4)
C(5)-C(6)	1.529(3)	N(3')-H(3')	0.76(4)
C(6)-C(7)	1.387(3)	N(3')-C(15')	1.340(3)
C(6)-C(11)	1.410(3)	N(3')-C(16')	1.420(3)
C(7)-H(7)	0.9500	N(4')-C(19')	1.340(4)

N(4')-C(20')	1.344(3)	C(18')-C(19')	1.422(3)
N(5')-H(5'A)	0.85(4)	C(18')-C(21')	1.506(4)
N(5')-H(5'B)	0.82(4)	C(20')-H(20')	0.9500
N(5')-C(19')	1.369(3)	C(21')-H(21D)	0.9800
C(1')-H(1'A)	0.9900	C(21')-H(21E)	0.9800
C(1')-H(1'B)	0.9900	C(21')-H(21F)	0.9800
C(1')-C(2')	1.533(3)		
C(2')-H(2')	1.0000	C(9)-S(1)-C(13)	88.83(13)
C(2')-C(3')	1.528(3)	C(1)-N(1)-C(5)	115.09(19)
C(2')-C(12')	1.531(3)	C(14)-N(1)-C(1)	125.2(2)
C(3')-H(3'A)	0.9900	C(14)-N(1)-C(5)	119.7(2)
C(3')-H(3'B)	0.9900	C(13)-N(2)-C(8)	110.0(2)
C(3')-C(4')	1.532(4)	C(15)-N(3)-H(3)	117(2)
C(4')-H(4'A)	0.9900	C(15)-N(3)-C(16)	123.9(2)
C(4')-H(4'B)	0.9900	C(16)-N(3)-H(3)	118(2)
C(4')-C(5')	1.534(3)	C(19)-N(4)-C(20)	118.0(2)
C(5')-H(5')	1.0000	H(5A)-N(5)-H(5B)	121(3)
C(5')-C(6')	1.527(3)	C(19)-N(5)-H(5A)	115(2)
C(6')-C(7')	1.388(4)	C(19)-N(5)-H(5B)	116(3)
C(6')-C(11')	1.413(3)	N(1)-C(1)-H(1A)	109.5
C(7')-H(7')	0.9500	N(1)-C(1)-H(1B)	109.5
C(7')-C(8')	1.398(4)	N(1)-C(1)-C(2)	110.5(2)
C(8')-C(9')	1.402(4)	H(1A)-C(1)-H(1B)	108.1
C(9')-C(10')	1.397(4)	C(2)-C(1)-H(1A)	109.5
C(10')-H(10')	0.9500	C(2)-C(1)-H(1B)	109.5
C(10')-C(11')	1.379(4)	C(1)-C(2)-H(2)	107.8
C(11')-H(11')	0.9500	C(3)-C(2)-C(1)	109.21(19)
C(12')-H(12D)	0.9800	C(3)-C(2)-H(2)	107.8
C(12')-H(12E)	0.9800	C(12)-C(2)-C(1)	111.6(2)
C(12')-H(12F)	0.9800	C(12)-C(2)-H(2)	107.8
C(13')-H(13')	0.9500	C(12)-C(2)-C(3)	112.3(2)
C(14')-C(15')	1.554(3)	C(2)-C(3)-H(3A)	109.4
C(16')-C(17')	1.400(4)	C(2)-C(3)-H(3B)	109.4
C(16')-C(20')	1.384(3)	C(2)-C(3)-C(4)	111.24(19)
C(17')-H(17')	0.9500	H(3A)-C(3)-H(3B)	108.0
C(17')-C(18')	1.375(4)	C(4)-C(3)-H(3A)	109.4
C(4)-C(3)-H(3B)	109.4	H(12B)-C(12)-H(12C)	109.5
C(3)-C(4)-H(4A)	108.7	S(1)-C(13)-H(13)	121.5
C(3)-C(4)-H(4B)	108.7	N(2)-C(13)-S(1)	117.0(2)
C(3)-C(4)-C(5)	114.1(2)	N(2)-C(13)-H(13)	121.5
H(4A)-C(4)-H(4B)	107.6	O(1)-C(14)-N(1)	124.3(2)
C(5)-C(4)-H(4A)	108.7	O(1)-C(14)-C(15)	117.2(2)
C(5)-C(4)-H(4B)	108.7	N(1)-C(14)-C(15)	118.4(2)
N(1)-C(5)-C(4)	109.62(19)	O(2)-C(15)-N(3)	125.0(2)

N(1)-C(5)-H(5)	107.1	O(2)-C(15)-C(14)	123.0(2)
N(1)-C(5)-C(6)	109.70(19)	N(3)-C(15)-C(14)	111.9(2)
C(4)-C(5)-H(5)	107.1	C(17)-C(16)-N(3)	122.5(2)
C(6)-C(5)-C(4)	115.7(2)	C(20)-C(16)-N(3)	119.2(2)
C(6)-C(5)-H(5)	107.1	C(20)-C(16)-C(17)	118.1(2)
C(7)-C(6)-C(5)	123.5(2)	C(16)-C(17)-H(17)	120.0
C(7)-C(6)-C(11)	119.4(2)	C(18)-C(17)-C(16)	120.0(2)
C(11)-C(6)-C(5)	117.1(2)	C(18)-C(17)-H(17)	120.0
C(6)-C(7)-H(7)	120.4	C(17)-C(18)-C(19)	117.7(2)
C(6)-C(7)-C(8)	119.1(2)	C(17)-C(18)-C(21)	121.7(2)
C(8)-C(7)-H(7)	120.4	C(19)-C(18)-C(21)	120.5(2)
N(2)-C(8)-C(7)	124.9(2)	N(4)-C(19)-N(5)	116.5(2)
N(2)-C(8)-C(9)	115.1(2)	N(4)-C(19)-C(18)	122.5(2)
C(9)-C(8)-C(7)	120.1(2)	N(5)-C(19)-C(18)	121.0(2)
C(8)-C(9)-S(1)	109.2(2)	N(4)-C(20)-C(16)	123.7(2)
C(10)-C(9)-S(1)	129.6(2)	N(4)-C(20)-H(20)	118.2
C(10)-C(9)-C(8)	121.2(2)	C(16)-C(20)-H(20)	118.2
C(9)-C(10)-H(10)	121.1	C(18)-C(21)-H(21A)	109.5
C(11)-C(10)-C(9)	117.8(2)	C(18)-C(21)-H(21B)	109.5
C(11)-C(10)-H(10)	121.1	C(18)-C(21)-H(21C)	109.5
C(6)-C(11)-H(11)	118.8	H(21A)-C(21)-H(21B)	109.5
C(10)-C(11)-C(6)	122.4(2)	H(21A)-C(21)-H(21C)	109.5
C(10)-C(11)-H(11)	118.8	H(21B)-C(21)-H(21C)	109.5
C(2)-C(12)-H(12A)	109.5	C(9')-S(1')-C(13')	88.88(13)
C(2)-C(12)-H(12B)	109.5	C(1')-N(1')-C(5')	114.60(19)
C(2)-C(12)-H(12C)	109.5	C(14')-N(1')-C(1')	126.9(2)
H(12A)-C(12)-H(12B)	109.5	C(14')-N(1')-C(5')	118.2(2)
H(12A)-C(12)-H(12C)	109.5	C(13')-N(2')-C(8')	109.5(2)
C(15')-N(3')-H(3')	118(3)	C(6')-C(5')-H(5')	107.4
C(15')-N(3')-C(16')	127.2(2)	C(7')-C(6')-C(5')	122.6(2)
C(16')-N(3')-H(3')	115(3)	C(7')-C(6')-C(11')	119.3(2)
C(19')-N(4')-C(20')	119.2(2)	C(11')-C(6')-C(5')	118.0(2)
H(5'A)-N(5')-H(5'B)	114(4)	C(6')-C(7')-H(7')	120.2
C(19')-N(5')-H(5'A)	118(3)	C(6')-C(7')-C(8')	119.6(2)
C(19')-N(5')-H(5'B)	121(3)	C(8')-C(7')-H(7')	120.2
N(1')-C(1')-H(1'A)	109.6	N(2')-C(8')-C(7')	124.8(2)
N(1')-C(1')-H(1'B)	109.6	N(2')-C(8')-C(9')	115.3(2)
N(1')-C(1')-C(2')	110.07(19)	C(7')-C(8')-C(9')	119.8(2)
H(1'A)-C(1')-H(1'B)	108.2	C(8')-C(9')-S(1')	109.38(19)
C(2')-C(1')-H(1'A)	109.6	C(10')-C(9')-S(1')	129.2(2)
C(2')-C(1')-H(1'B)	109.6	C(10')-C(9')-C(8')	121.5(2)
C(1')-C(2')-H(2')	107.9	C(9')-C(10')-H(10')	121.2
C(3')-C(2')-C(1')	109.4(2)	C(11')-C(10')-C(9')	117.7(2)
C(3')-C(2')-H(2')	107.9	C(11')-C(10')-H(10')	121.2

C(3')-C(2')-C(12')	113.4(2)	C(6')-C(11')-H(11')	118.9
C(12')-C(2')-C(1')	110.0(2)	C(10')-C(11')-C(6')	122.2(2)
C(12')-C(2')-H(2')	107.9	C(10')-C(11')-H(11')	118.9
C(2')-C(3')-H(3'A)	109.3	C(2')-C(12')-H(12D)	109.5
C(2')-C(3')-H(3'B)	109.3	C(2')-C(12')-H(12E)	109.5
C(2')-C(3')-C(4')	111.5(2)	C(2')-C(12')-H(12F)	109.5
H(3'A)-C(3')-H(3'B)	108.0	H(12D)-C(12')-H(12E)	109.5
C(4')-C(3')-H(3'A)	109.3	H(12D)-C(12')-H(12F)	109.5
C(4')-C(3')-H(3'B)	109.3	H(12E)-C(12')-H(12F)	109.5
C(3')-C(4')-H(4'A)	108.9	S(1')-C(13')-H(13')	121.5
C(3')-C(4')-H(4'B)	108.9	N(2')-C(13')-S(1')	117.0(2)
C(3')-C(4')-C(5')	113.3(2)	N(2')-C(13')-H(13')	121.5
H(4'A)-C(4')-H(4'B)	107.7	O(1')-C(14')-N(1')	123.3(2)
C(5')-C(4')-H(4'A)	108.9	O(1')-C(14')-C(15')	116.5(2)
C(5')-C(4')-H(4'B)	108.9	N(1')-C(14')-C(15')	120.2(2)
N(1')-C(5')-C(4')	108.90(19)	O(2')-C(15')-N(3')	125.4(2)
N(1')-C(5')-H(5')	107.4	O(2')-C(15')-C(14')	124.4(2)
N(1')-C(5')-C(6')	109.47(19)	N(3')-C(15')-C(14')	110.1(2)
C(4')-C(5')-H(5')	107.4	C(17')-C(16')-N(3')	118.3(2)
C(6')-C(5')-C(4')	115.8(2)	C(20')-C(16')-N(3')	123.7(2)
C(20')-C(16')-C(17')	118.0(2)	N(4')-C(20')-C(16')	122.4(2)
C(16')-C(17')-H(17')	119.5	N(4')-C(20')-H(20')	118.8
C(18')-C(17')-C(16')	121.1(2)	C(16')-C(20')-H(20')	118.8
C(18')-C(17')-H(17')	119.5	C(18')-C(21')-H(21D)	109.5
C(17')-C(18')-C(19')	116.8(2)	C(18')-C(21')-H(21E)	109.5
C(17')-C(18')-C(21')	122.7(2)	C(18')-C(21')-H(21F)	109.5
C(19')-C(18')-C(21')	120.4(2)	H(21D)-C(21')-H(21E)	109.5
N(4')-C(19')-N(5')	116.8(2)	H(21D)-C(21')-H(21F)	109.5
N(4')-C(19')-C(18')	122.5(2)	H(21E)-C(21')-H(21F)	109.5
N(5')-C(19')-C(18')	120.6(2)		

Table SI-6. Anisotropic displacement parameters (Å^{2}x 10^3) for TangoTNG908. The anisotropic displacement factor exponent takes the form: $-2\pi^2[h^2 a^{*2}U^{11} + ... + 2 h k a^* b^* U^{12}]$

	U^{11}	U^{22}	U^{33}	U^{23}	U^{13}	U^{12}
S(1)	27(1)	20(1)	28(1)	2(1)	-3(1)	-8(1)
O(1)	23(1)	28(1)	19(1)	2(1)	1(1)	4(1)
O(2)	17(1)	35(1)	19(1)	-1(1)	-2(1)	-6(1)
N(1)	17(1)	18(1)	16(1)	1(1)	-2(1)	0(1)
N(2)	24(1)	25(1)	20(1)	2(1)	-1(1)	-6(1)
N(3)	15(1)	23(1)	16(1)	1(1)	-3(1)	-3(1)
N(4)	28(1)	22(1)	18(1)	2(1)	-3(1)	-9(1)

N(5)	32(1)	28(1)	17(1)	4(1)	-4(1)	-13(1)
C(1)	16(1)	18(1)	20(1)	0(1)	-2(1)	0(1)
C(2)	19(1)	18(1)	21(1)	-1(1)	-2(1)	-1(1)
C(3)	21(1)	20(1)	19(1)	-3(1)	-2(1)	-3(1)
C(4)	20(1)	20(1)	16(1)	0(1)	-3(1)	-4(1)
C(5)	18(1)	19(1)	14(1)	0(1)	-2(1)	-1(1)
C(6)	18(1)	17(1)	17(1)	1(1)	-4(1)	0(1)
C(7)	22(1)	17(1)	16(1)	-1(1)	-4(1)	-2(1)
C(8)	19(1)	20(1)	19(1)	1(1)	-4(1)	-1(1)
C(9)	23(1)	17(1)	25(1)	2(1)	-6(1)	-4(1)
C(10)	31(1)	20(1)	22(1)	-2(1)	-5(1)	-5(1)
C(11)	28(1)	21(1)	18(1)	-1(1)	-1(1)	-1(1)
C(12)	23(1)	21(1)	29(1)	3(1)	-6(1)	-5(1)
C(13)	25(1)	24(1)	22(1)	4(1)	0(1)	-5(1)
C(14)	18(1)	18(1)	17(1)	1(1)	-2(1)	-5(1)
C(15)	18(1)	17(1)	16(1)	-1(1)	-1(1)	-3(1)
C(16)	19(1)	18(1)	17(1)	-1(1)	-2(1)	1(1)
C(17)	20(1)	20(1)	20(1)	-1(1)	0(1)	-3(1)
C(18)	19(1)	20(1)	20(1)	-2(1)	-2(1)	-2(1)
C(19)	23(1)	21(1)	17(1)	-1(1)	-1(1)	-1(1)
C(20)	23(1)	21(1)	18(1)	-2(1)	-4(1)	-6(1)
C(21)	27(1)	31(1)	19(1)	0(1)	-3(1)	-11(1)
S(1')	24(1)	18(1)	25(1)	2(1)	-4(1)	-1(1)
O(1')	22(1)	21(1)	19(1)	-1(1)	-2(1)	-7(1)
O(2')	19(1)	41(1)	19(1)	3(1)	0(1)	-8(1)
N(1')	20(1)	18(1)	15(1)	1(1)	-1(1)	-4(1)
N(2')	28(1)	26(1)	21(1)	1(1)	-6(1)	-3(1)
N(3')	16(1)	23(1)	17(1)	2(1)	0(1)	-6(1)
N(4')	24(1)	22(1)	20(1)	1(1)	-1(1)	-6(1)
N(5')	31(1)	31(1)	18(1)	4(1)	-3(1)	-11(1)
C(1')	18(1)	20(1)	18(1)	1(1)	-2(1)	-6(1)
C(2')	19(1)	19(1)	21(1)	0(1)	-2(1)	-4(1)
C(3')	20(1)	22(1)	19(1)	-2(1)	-2(1)	-1(1)
C(4')	19(1)	21(1)	18(1)	1(1)	0(1)	-2(1)
C(5')	21(1)	20(1)	14(1)	2(1)	-1(1)	-6(1)
C(6')	20(1)	16(1)	20(1)	1(1)	-1(1)	-6(1)
C(7')	23(1)	19(1)	18(1)	0(1)	1(1)	-4(1)
C(8')	23(1)	21(1)	19(1)	1(1)	-2(1)	-7(1)
C(9')	22(1)	15(1)	23(1)	3(1)	-1(1)	-3(1)
C(10')	28(1)	17(1)	20(1)	-1(1)	0(1)	-3(1)
C(11')	27(1)	19(1)	18(1)	0(1)	-4(1)	-5(1)
C(12')	24(1)	19(1)	27(1)	1(1)	-5(1)	-1(1)
C(13')	28(1)	22(1)	23(1)	4(1)	-7(1)	-4(1)
C(14')	18(1)	16(1)	18(1)	-2(1)	1(1)	-1(1)

C(15')	19(1)	18(1)	16(1)	-1(1)	-1(1)	-3(1)
C(16')	18(1)	19(1)	15(1)	0(1)	0(1)	-1(1)
C(17')	20(1)	20(1)	21(1)	0(1)	1(1)	-3(1)
C(18')	18(1)	22(1)	18(1)	-2(1)	-2(1)	-2(1)
C(19')	21(1)	24(1)	17(1)	-1(1)	0(1)	-3(1)
C(20')	22(1)	22(1)	19(1)	-3(1)	-2(1)	-4(1)
C(21')	28(1)	33(1)	20(1)	0(1)	-3(1)	-11(1)

Table SI-7. Hydrogen coordinates (x 10^4) and isotropic displacement parameters (Å^{2}x 10 ^{3}) for TangoTNG908.

	x	y	z	U(eq)
H(3)	2440(70)	7310(20)	1480(20)	19(8)
H(5A)	5170(80)	6200(30)	5850(30)	28(9)
H(5B)	3120(80)	5570(30)	5650(30)	32(9)
H(1A)	9209	6540	-82	22
H(1B)	10464	7092	-996	22
H(2)	10126	5516	-1434	24
H(3A)	7410	5888	-2781	24
H(3B)	9471	6641	-2688	24
H(4A)	3845	6978	-2161	22
H(4B)	5369	7552	-2991	22
H(5)	4014	8430	-1525	21
H(7)	9083	8185	-3273	22
H(10)	9843	10652	-1134	29
H(11)	7028	9500	-736	26
H(12A)	6714	5148	-310	36
H(12B)	5997	4910	-1361	36
H(12C)	4541	5877	-859	36
H(13)	15118	10232	-4476	29
H(17)	6909	7720	2993	24
H(20)	1491	5945	2649	24
H(21A)	8595	7835	4521	37
H(21B)	8391	6940	5267	37
H(21C)	6155	7881	5320	37
H(3')	-1510(80)	2800(20)	8600(30)	24(9)
H(5'A)	10(80)	4860(30)	4490(30)	33(9)
H(5'B)	-2060(80)	4330(30)	4330(30)	29(9)
H(1'A)	4692	3544	10121	22
H(1'B)	6004	2913	10997	22
H(2')	4480	4478	11531	23
H(3'A)	1491	4102	12866	25
H(3'B)	4086	3299	12724	25
H(4'A)	-1167	3123	12187	24

H(4'B)	396	2478	12998	24
H(5')	261	1664	11510	22
H(7')	4099	1652	13312	24
H(10')	8467	-430	10920	26
H(11')	4830	733	10576	25
H(12D)	-646	4263	10905	35
H(12E)	1284	4985	10421	35
H(12F)	-89	5175	11477	35
H(13')	11194	-507	14451	29
H(17')	-3003	2451	7082	24
H(20')	2364	4258	7414	25
H(21D)	-2741	2487	4728	39
H(21E)	-5083	3376	4918	39
H(21F)	-4983	2409	5584	39

Table SI-8. Hydrogen bonds for TNG908 [Å and °].

D-H...A	d(D-H)	d(H...A)	d(D...A)	<(DHA)
N(3)-H(3)...O(2)#1	0.82(4)	2.12(4)	2.916(3)	163(3)
N(5)-H(5B)...N(4')	0.83(4)	2.19(4)	3.023(3)	175(4)
N(3')-H(3')...O(2')#1	0.76(4)	2.21(4)	2.870(3)	146(3)
N(5')-H(5'A)...N(4)	0.85(4)	2.17(4)	3.013(3)	170(3

Symmetry transformations used to generate equivalent atoms:

#1 x-1,y,z

Biochemical fluorescence anisotropy peptide displacement assay.

A fluorescence anisotropy (FA) assay was established to measure binding of C-terminal 5′-TAMRA labeled histone H4 peptide (1-21) with PRMT5/MEP50. The test compound competes with the peptide to bind to PRMT5/MEP50 protein and thus would act to disrupt binding by the labeled histone H4 peptide. The assay buffer consisted of 30 mM Bicine (pH 8.0), 150 mM NaCl, 1.5 mM DTT, 0.003% Tween-20. The two peptides were utilized for these studies were Me_0: Ac-SGRGKGGKGLGKGGAKRHRKV-K(5-TAMRA)-NH_2 and Me_2: Ac-SGR(Sym Me2)GKGGKGLGKGGAKRHRKV-K(5-TAMRA)-NH_2. Me_0 peptide is not methylated and used to determine the compound potency in the absence of cofactor and presence of 50 µM 5′-methylthioadenosine (MTA). Its K_d with apoPRMT5 = 35.6 nM and with PRMT5•MTA = 4.6 nM. Me_2 peptide is symmetrically methylated at Arginine 3 and used to determine the compound potency in the presence of 50 µM S-adenosyl methionine (SAM). Its K_d with PRMT5•SAM = 79 nM. Inhibitor potency was assessed at equilibrium by measuring the dose dependent displacement of a fixed concentration of the peptide from PRMT5/MEP50. Following incubation at RT for 30 minutes, the plate was read on an Envision plate reader. For data analysis, fluorescence anisotropy (FA) detected equals 1000 * (S - G * P) / (S + G * 2 * P) where: S = detector 2 or channel 2 signal, P = detector 1 or channel 1 signal, G = G-factor. Fluorescence anisotropy is normalized to %inhibition using: %inhibition=(Signal-MinAVG)/(MaxAVG-MinAVG)*100, where MinAVG = the average value of Min value and MaxAVG = the average value of Max value. Curves are fitted by XL-Fit as %inhibition vs. log [compound concentration] using a 4 parameter logistic equation with fixed 0% and 100% inhibition limits. The apparent K_i values of **TNG908** were calculated using the Cheng-Prusoff equation for competitive inhibitor ($K_i = IC_{50}/(1+[S]/K_M)$ where [S] = the concentration of the substrate used in the binding assay and K_M = the Michaelis constant, the substrate concentration at which the reaction rate is 50% of the V_{max})[1].

Double titration K_i measurement of TNG908 against apo-PRMT5 and PRMT5·MTA complex.
To measure the K_i of TNG908 to the apo-PRMT5 and PRMT5·MTA complexes, double titrations of PRMT5 and **TNG908** using the FA assay were performed. Both assays were run at 25 nM Me0 peptide tracer concentrations in assay buffer. 30 mM Bicine (pH 8.0), 150 mM NaCl, 1.5 mM DTT, 0.003% Tween-20. For apo-PRMT5, 110, 66.6, 44.4, 29.6, 17.7 and 13.2 nM enzyme concentrations were used and **TNG908** was titrated from 500 nM with 2-fold serial dilution. For PRMT5·MTA complex, 18.8, 12.5, 8.3, 5.6, 3.7 and 2.5 nM enzyme concentrations were used in the presence of 50 µM MTA and TNG908 was titrated from 100 nM with 2-fold serial dilution. After mixing with PRMT5, **TNG908** and Me_0 peptide, the mixtures were incubated at room temperature for 1h. The signals were read on an Envision plate reader. Fluorescence polarization is normalized to %bound. Minimum signal is the control mixture without PRMT5 protein, and the maximum signal is the control mixture of Me_0 tracer with 1 µM PRMT5 (apo) or 0.5 µM PRMT5 (PRMT5·MTA complex). %Bound =(Signal-MinAVG)/(MaxAVG-MinAVG)*100. KinTek Explorer software was used to global fit the data to obtain K_d values of **TNG908** and Me_0 peptide tracer bound to apo PRMT5 or PRMT5·MTA complex.

Biochemical methyl transferase FlashPlate assay.
A FlashPlate-based radioactive assay was used to measure purified humanPRMT5/MEP50 methyltransferase activity. Each well of the FlashPlate is embedded with a thin layer of polystyrene-based scintillant and streptavidin molecules. PRMT5/MEP50 transfers the methyl group from 3H-SAM to biotinylated histone H4 (1-21) peptide. Streptavidin on the FlashPlate captures biotinylated 3H-methylated peptide and brings it in proximity with the embedded scintillant, resulting in conversion of radioactive energy to light. A liquid scintillation counter is used to detect and quantify the light. This assay permitted determination of PRMT5 enzyme activity and its inhibition in the presence and absence of compounds. The assay reactions were also conducted in the presence and absence of 0.3 µM of MTA to determine whether the compounds exhibit MTA-cooperative activity.
To test the inhibition of **TNG908** against PRMT5/MEP50, the reaction was performed in 30 mM bicine pH 8.0, 150 mM NaCl, 1.5 mM DTT and 0.003% Tween-20 with 10 µM biotinylated histone H4 (1-21) and 0.25 µM 3H-SAM in a total volume of 12.5 µl PRMT5/MEP50 complex at final concentration of 5 nM. This mixture was preincubated with compound and 0.25 µM 3H-SAM in the presence or absence of 0.3 µM MTA for 30 minutes at room temperature. Histone H4 (1–21) at final concentration of 10 µM was added to initiate the reaction. Following 2.5h incubation at room temperature, the reaction was stopped by adding 0.1 mM cold SAM and 0.1 mM SAH. The reaction was transferred into a FlashPlate and incubated for 1h at room temperature. The plate was centrifuged before reading on MicroBeta2 scintillation counter. The IC_{50} value for each compound was calculated from each 10-point dose−response curve for samples plus and minus MTA using XLfit. To calculate the apparent binding affinity ($K_{i,app}$) of **TNG908** against PRMT5/MEP50 with or without MTA, the Cheng-Prusoff equation for competitive inhibitor was applied.

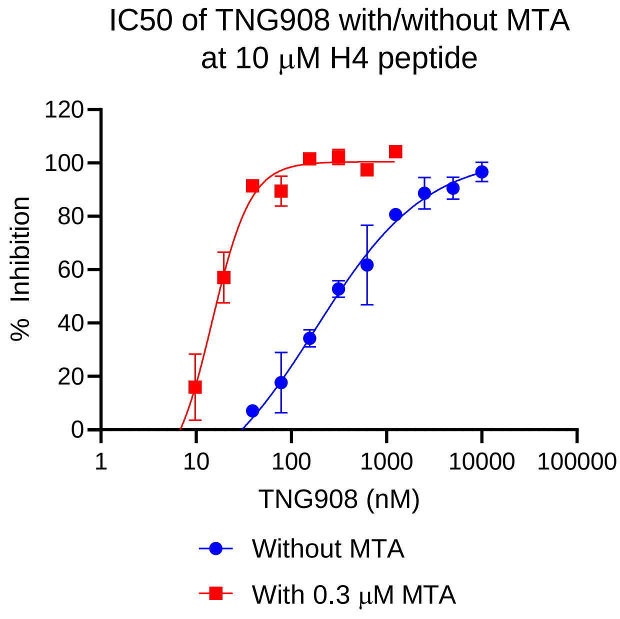

Figure SI-2: TNG908 inhibits PRMT5 and shows enhanced potency with MTA·PRMT5 complex (average of N=3).

Table SI-9: IC50s of TNG908 against PRMT5/MEP50 at 10 μM H4 peptide in the absence and presence of 0.3 μM MTA

	IC50 (nM)	Ki (nM)
No MTA	262 ± 52	3.2
With 0.3 μM MTA	21.2 ± 9.3	0.26

Reversibility measurement using Zeba™ spin column activity recovery assay.
Reversibility and activity recovery experiments were performed by first preincubating enzyme [E] and inhibitor [I] together to form E·I complex in the absence and presence of MTA. Then, the [E·I] complex along with all free enzyme and inhibitor passes through Zeba™ Spin Desalting Columns (7K MWCO, ThermoFisher) to obtain [E·I] complex in flow-through only. Under excess [I], no PRMT5 methyltransferase activity could be observed due to no free enzyme in the reaction. However, PRMT5 activity recovery could be observed as a function of the time if the binding of inhibitor is reversible as the bound inhibitor would be dissociated from the [E·I] complex to give free E, as shown in scheme SI-1. If the release of the inhibitor is slow, a lag phase of enzyme activity recovery could be observed, from which the off rate (k_{off}) of the compound could be calculated. If the inhibitor is a rapidly equilibrating, but tight binding compound, no lag phase would be observed, but the affinity of compound (K_i) could be estimatedd by comparing the recovered slope with the slope of no inhibitor control (enzyme alone).

SI Scheme SI-1: Spin column assay to measure the reversibility and binding affinity of TNG908

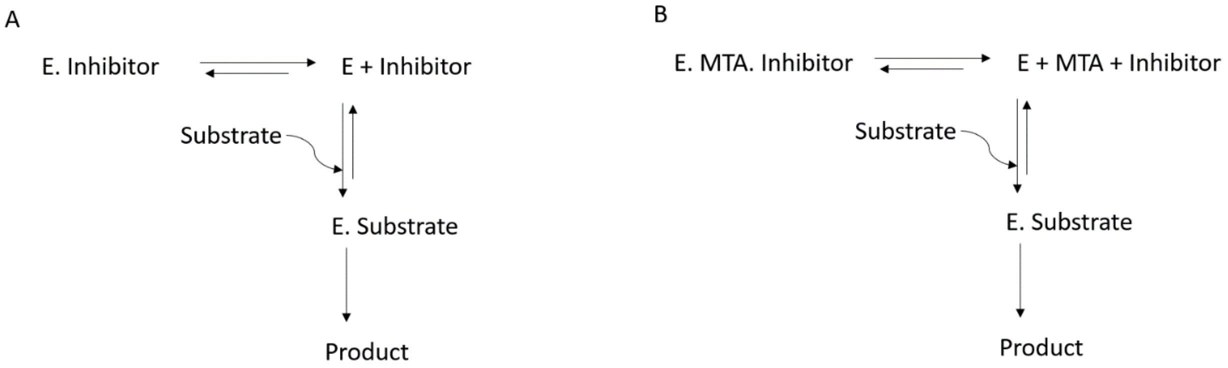

To measure reversibility and calculate apparent binding affinity ($K_{i,app}$) of **TNG908** against PRMT5/MEP50, the reaction was performed in 30 mM bicine pH 8.0, 150 mM NaCl, 1.5 mM DTT and 0.003% Tween-20. PRMT5/MEP50 complex at final concentration of 5 nM was preincubated with compound in the presence or absence of MTA for 2h at room temperature. The samples flow through the spin column. Biotinylated histone H4 (1–21) at final concentration of 1 μM and SAM at final concentration of 10 μM were added to initiate the reaction. Following 5h time course at room temperature, the reaction was stopped at specific timepoints by adding 0.5 mM cold SAM and 0.05 mM SAH in assay buffer. The reaction was transferred into a FlashPlate and incubated for 1h at room temperature. The plate was centrifuged before reading on MicroBeta2 scintillation counter.
For the spin column assay, all data were fitted to simple linear regression in Prism 9.3 to obtain the slopes for each condition. Fractions of enzyme recovery were first calculated by using slope of (E + I) arm divided by slope of Max signal (E + DMSO) arm in the presence or absence of MTA. The total [EI] complex after equilibrium were then calculated by using enzyme concentration [E_{total}] x (1 - %fraction of enzyme recovery). The reversibility and linear activity of **TNG908** post excess compound removal allows us to use the simple fast binding equilibrium: [EI] = [E] + [I] to calculate the binding affinity of the compound. The apparent binding affinity ($K_{i,app}$) of compound at 1 μM peptide condition was calculated by using the equation $K_{i,app} = (E_{total} - EI) \times (I_{total} - EI)/EI$ in Excel. The real Ki of a compound can be calculated by using the equation $K_{i,app} = K_i \times (1 + [S]/K_m)$ where [S] of peptide is 1 μM and K_M of peptide is 0.125 μM

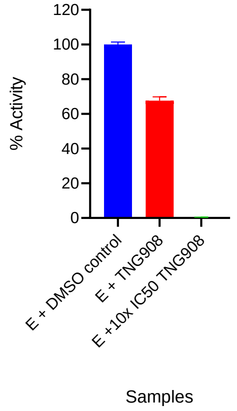

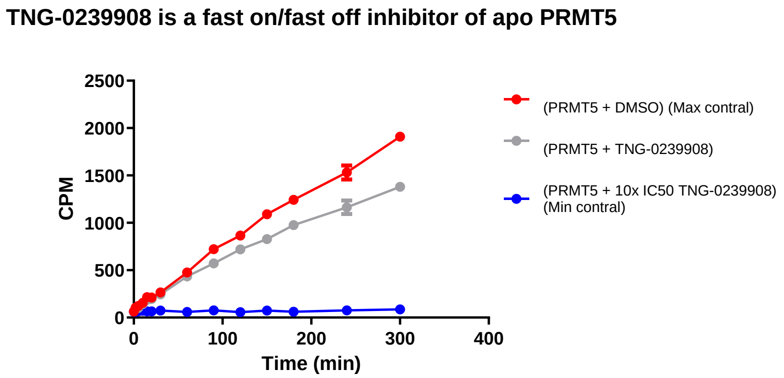

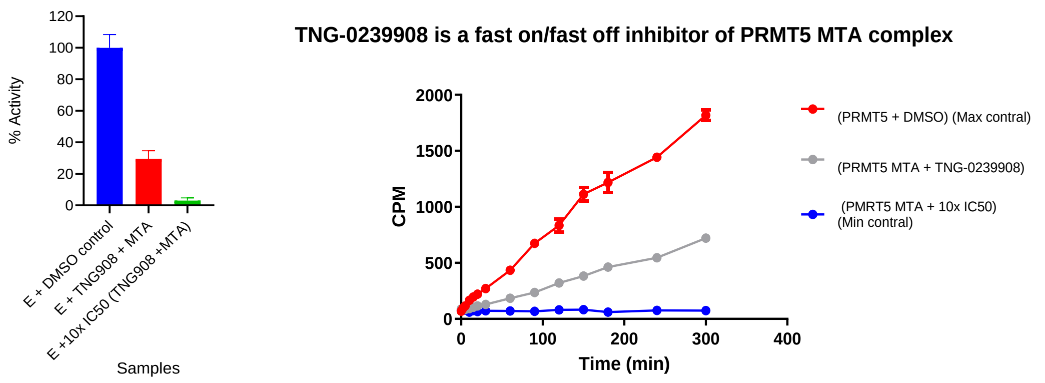

Figure SI-3: (B) PRMT5 activity recovery from PRMT5·TNG908 binary complex. (C) PRMT5 activity recovery from PRMT5·TNG908·MTA ternary complex.

SI Table SI-10: % activity recovery of TNG908 and TNG908 with MTA and their calculated Ki.

	% Activity recovered	Ki, app (nM) calculated	Ki, real (nM)
TNG908	68%, 74%	9.3 ± 2.5	1.03
TNG908 + MTA	30%, 33%	0.73 ± 0.11	0.081

Methyltransferase panel data for TNG908

Table SI-11. TNG908 inhibition profile across 38 methyltransferases

Methyltransferase	Substrate	TNG908, % Enzyme Activity (@10μM)	TNG908, % Enzyme Activity (@1μM)	Control IC_{50} (M)
ASH1L	Nucleosomes	116	103	1.4E-08
DNMT	Poly dl-dC	72	88	8.4E-08
DNMT3a	Lambda DNA	86	92	3.0E-07
DNMT3b	Lambda DNA	99	97	1.2E-07
DNMT3b/3L	Lambda DNA	96	100	8.1E-08
DOT1L	Nucleosomes	75	83	1.7E-07
EZH1 Complex	Core Histone	93	103	2.5E-05
EZH2 Complex	Core Histone	105	90	3.5E-05
EZH2(Y641F) Complex	Core Histone	88	91	4.4E-05
G9a	Histone H3 1-21	90	87	1.3E-06
GLP	Histone H3 1-21	92	92	5.0E-07
MLL1 Complex	Nucleosomes	94	102	6.6E-07
MLL2 Complex	Nucleosomes	83	100	1.1E-05
MLL3 Complex	Nucleosomes	88	88	1.1E-05
MLL4 Complex	Nucleosomes	102	98	1.3E-06
NSD1	Nucleosomes	118	106	3.0E-06
NSD2	Nucleosomes	88	92	2.5E-06
NSD2 (E1099K)	Nucleosomes	83	99	6.6E-07
NSD2 (T1150A)	Nucleosomes	90	93	1.1E-06
NSD3	Nucleosomes	102	98	5.9E-08
PRDM9	Histone H3	88	90	5.1E-06

PRMT1	Histone H4	93	103	2.5E-07
PRMT3	Histone H4	75	80	1.1E-06
PRMT4	Histone H3	92	93	1.5E-07
PRMT5/MEP50 Complex	Histone H2A	6	26	1.3E-06
PRMT5 (C449S) /MEP50 Complex	Histone H2A	9	25	3.1E-06
PRMT6	GST-GAR	98	94	2.0E-07
PRMT7	GST-GAR	87	95	2.6E-07
PRMT8	Histone H4	98	106	8.2E-08
SET1b Complex	Core Histone	99	96	4.3E-06
SET7/9	Core Histone	95	93	9.9E-05
SET8	Nucleosomes	96	93	1.0E-07
SETD2	Nucleosomes	91	92	1.1E-06
SMYD2	Histone H4	103	104	1.0E-07
SMYD3	MEKK2	106	98	3.2E-05
SUV39H1	Histone H3	104	100	9.7E-05
SUV39H2	Histone H3	108	108	5.0E-05
SUV420H1TV2	Nucleosomes	96	91	3.7E-04

Eurofins SAFETYscan panel for TNG908

Table SI-12. TNG908 E/IC50 ELECT data across 78 assays

Target Class	Assay Name	Assay Target	Mode	Result Type	RC50 (uM)	Max Response
GPCR	Calcium Flux	ADORA2A	Agonist	EC50	>10	0
GPCR	Calcium Flux	ADRA1A	Agonist	EC50	>10	0.81
GPCR	Calcium Flux	AVPR1A	Agonist	EC50	>10	0
GPCR	Calcium Flux	CCKAR	Agonist	EC50	>10	1.1
GPCR	Calcium Flux	CHRM1	Agonist	EC50	>10	1.41
GPCR	Calcium Flux	CHRM3	Agonist	EC50	>10	0
GPCR	Calcium Flux	EDNRA	Agonist	EC50	>10	0
GPCR	Calcium Flux	HRH1	Agonist	EC50	>10	0.82
GPCR	Calcium Flux	HTR2A	Agonist	EC50	>10	3.8
GPCR	Calcium Flux	HTR2B	Agonist	EC50	>10	0
GPCR	Calcium Flux	ADORA2A	Antagonist	IC50	>10	20.4
GPCR	Calcium Flux	ADRA1A	Antagonist	IC50	>10	11.72
GPCR	Calcium Flux	AVPR1A	Antagonist	IC50	>10	12.43
GPCR	Calcium Flux	CCKAR	Antagonist	IC50	>10	0
GPCR	Calcium Flux	CHRM1	Antagonist	IC50	>10	7.57
GPCR	Calcium Flux	CHRM3	Antagonist	IC50	>10	0
GPCR	Calcium Flux	EDNRA	Antagonist	IC50	>10	0
GPCR	Calcium Flux	HRH1	Antagonist	IC50	>10	7.87
GPCR	Calcium Flux	HTR2A	Antagonist	IC50	>10	11.26
GPCR	Calcium Flux	HTR2B	Antagonist	IC50	4.44355	74.11
GPCR	cAMP	ADRA2A	Agonist	EC50	>10	4.96

GPCR	cAMP	ADRB1	Agonist	EC50	>10	0.39
GPCR	cAMP	ADRB2	Agonist	EC50	>10	0.31
GPCR	cAMP	CHRM2	Agonist	EC50	>10	0
GPCR	cAMP	CNR1	Agonist	EC50	>10	2.1
GPCR	cAMP	CNR2	Agonist	EC50	>10	10.58
GPCR	cAMP	DRD1	Agonist	EC50	>10	0
GPCR	cAMP	DRD2S	Agonist	EC50	>10	10.39
GPCR	cAMP	HRH2	Agonist	EC50	>10	0
GPCR	cAMP	HTR1A	Agonist	EC50	>10	7.7
GPCR	cAMP	HTR1B	Agonist	EC50	>10	10.25
GPCR	cAMP	OPRD1	Agonist	EC50	>10	6.3
GPCR	cAMP	OPRK1	Agonist	EC50	>10	7.78
GPCR	cAMP	OPRM1	Agonist	EC50	>10	4.49
GPCR	cAMP	ADRA2A	Antagonist	IC50	>10	5.34
GPCR	cAMP	ADRB1	Antagonist	IC50	>10	26.93
GPCR	cAMP	ADRB2	Antagonist	IC50	>10	24.35
GPCR	cAMP	CHRM2	Antagonist	IC50	>10	1.33
GPCR	cAMP	CNR1	Antagonist	IC50	>10	0
GPCR	cAMP	CNR2	Antagonist	IC50	>10	0
GPCR	cAMP	DRD1	Antagonist	IC50	>10	30.84
GPCR	cAMP	DRD2S	Antagonist	IC50	>10	0
GPCR	cAMP	HRH2	Antagonist	IC50	>10	14.48
GPCR	cAMP	HTR1A	Antagonist	IC50	>10	0
GPCR	cAMP	HTR1B	Antagonist	IC50	>10	0
GPCR	cAMP	OPRD1	Antagonist	IC50	>10	48.49
GPCR	cAMP	OPRK1	Antagonist	IC50	>10	10.45
GPCR	cAMP	OPRM1	Antagonist	IC50	>10	0.07
Ion Channel	Ion Channel	CAV1.2	Blocker	IC50	>10	0
Ion Channel	Ion Channel	GABAA	Blocker	IC50	>10	13.88
Ion Channel	Ion Channel	hERG	Blocker	IC50	>10	24
Ion Channel	Ion Channel	HTR3A	Blocker	IC50	>10	0
Ion Channel	Ion Channel	KvLQT1/minK	Blocker	IC50	>10	21.22
Ion Channel	Ion Channel	nAChR(a4/b2)	Blocker	IC50	>10	23.2
Ion Channel	Ion Channel	NAV1.5	Blocker	IC50	>10	23.69
Ion Channel	Ion Channel	NMDAR (1A/2B)	Blocker	IC50	>10	6.17
Ion Channel	Ion Channel	GABAA	Opener	EC50	>10	0.02
Ion Channel	Ion Channel	HTR3A	Opener	EC50	>10	0
Ion Channel	Ion Channel	KvLQT1/minK	Opener	EC50	>10	0
Ion Channel	Ion Channel	nAChR(a4/b2)	Opener	EC50	>10	2.74
Ion Channel	Ion Channel	NMDAR (1A/2B)	Opener	EC50	>10	0
Kinases	Binding	INSR	Inhibitor	IC50	>10	0
Kinases	Binding	LCK	Inhibitor	IC50	>10	18.98
Kinases	Binding	ROCK1	Inhibitor	IC50	>10	0
Kinases	Binding	VEGFR2	Inhibitor	IC50	>10	0.28

NHR	NHR Nuclear Translocation	AR	Agonist	EC50	>10	0
NHR	NHR Nuclear Translocation	AR	Antagonist	IC50	>10	0
NHR	NHR Protein Interaction	GR	Agonist	EC50	>10	0.89
NHR	NHR Protein Interaction	GR	Antagonist	IC50	>10	32.71
Non-Kinase Enzymes	Enzymatic	AChE	Inhibitor	IC50	>10	4.32
Non-Kinase Enzymes	Enzymatic	COX1	Inhibitor	IC50	>10	0
Non-Kinase Enzymes	Enzymatic	COX2	Inhibitor	IC50	>10	0
Non-Kinase Enzymes	Enzymatic	MAOA	Inhibitor	IC50	>10	7.83
Non-Kinase Enzymes	Enzymatic	PDE3A	Inhibitor	IC50	>10	3.91
Non-Kinase Enzymes	Enzymatic	PDE4D2	Inhibitor	IC50	>10	8.69
Transporter	Transporter	DAT	Blocker	IC50	>10	17.63
Transporter	Transporter	NET	Blocker	IC50	>10	14.48
Transporter	Transporter	SERT	Blocker	IC50	>10	9.87

MDCKII and MDR1-MDCKII assays.

Wild type (WT) MDCKII cells or MDR1-MDCKII cells (both obtained from Piet Borst at the Netherlands Cancer Institute) were seeded onto the polycarbonate membranes in the 96- well insert system at 4.44 x 10^5 cells/mL and cultured for 4-7 days until confluence before being used for the transport studies. Test compounds were diluted with the transport buffer (HPSS with 10 mM HEPES, pH 7.4) from DMSO stock solution to a concentration of 2 μM (DMSO < 1%) and applied to the apical or basolateral side of the cell monolayer. The plate was incubated for 2.5 h in CO_2 incubator at 37±1 °C, with 5% CO_2 at saturated humidity without shaking. Permeation of the test compounds from A to B or B to A direction was determined in duplicate. In addition, the efflux ratio of each compound was also calculated. For each transport assay, digoxin (P-gp efflux substrate) was tested at 10.0 μM bidirectionally, while nadolol (low permeability marker) and metoprolol (high permeability marker) were tested at 2.00 μM in A to B direction in duplicate. Test and reference compounds were quantified by LC-MS/MS analysis based on the peak area ratio of analyte/internal standard (IS). After transport assay, Lucifer yellow fluorescence rejection assay was performed to confirm the integrity of the cell monolayer.

Human liver microsome metabolic stability assay.

Test and reference compounds (testosterone, diclofenac and propafenone) at 1 μM were incubated individually in human liver microsome (0.5 mg protein/mL, from mixed-gender donors) supplemented with 1 mM NADPH at 37 °C for 60 minutes while shaking. Aliquots of 60 μl were taken at 5, 15, 30, 45 and 60 minutes of incubation and reactions were stopped by adding 180 ul of quenching solution. After which all sampling plates were shaken for 10 minutes, then centrifuged at 4000 rpm for 20 minutes at 4 °C. Supernatants were transferred to HPLC water (1:3) and mixed for 10 minutes prior to the LC-MS/MS analysis. Test and reference compounds were quantified by LC-MS/MS analysis based on the peak area ratio of analyte/internal standard (IS).

Kinetic solubility assay.

Medium:

The preparation of 50 mM phosphate buffer (PB) with pH 7.4:

The preparation of 50 mM NaH_2PO_4: Dissolved 3.000 g of NaH_2PO_4 in 500 mL H_2O, and the pH measured was about 4.5.

The preparation of 50 mM NaH_2PO_4: Dissolved 3.549 g of NaH_2PO_4 in 500 mL H_2O, and the pH measured was about 9.4.

The preparation of 50 mM PB (pH 7.4): 15 mL of 50 mM NaH_2PO_4 was added to a 50 mL tube and then adjusted to pH 7.4 +/- 0.05 with 50 mM NaH_2PO_4.

Procedure:
10μL of 10mM DMSO stock solution of test and control compounds was added into each well of a 96-well plate, respectively.
Added 490 μL of medium into the well of the 96-well plate, respectively.
Vortexed the solubility samples for at least 2 minutes.
Shook the 96-well plate on a shaker at room temperature at the speed of 800 rpm for 24h.
Centrifuged at 25 °C for 10 minutes (eq 4000 rpm).
Transferred the supernatant into a filter plate, and then collected the filtrates into a new 96-well plate by centrifuging for at least 5 minutes.
The concentrations of the filtrates were quantified by LC-UV system.

Human Ether-a-go-go-Related Gene (hERG) assay.
CHO cells stably expressing hERG potassium channels from Sophion Biosciences were used for this test. The cells were cultured in a humidified and air-controlled (5% CO2) incubator at 37 °C. The CHO cells which were at least two days after plating and more than 75% confluent would be used for experiments. Before testing, cells were harvested using TrypLE and resuspended in the physiological solution at the room temperature. For the electrophysiological recordings the following solutions were used (Table SI-13).

Table SI-13. Composition of Physiological, External, and Internal Solutions

Reagent	Physiological Solution (mM)	External Solution (mM)	Internal Solution (mM)
NaCl	140	80	10
KCl	4	4	10
KF	-	-	110
$CaCl_2$	2	2	-
$MgCl_2$	1	1	-
Glucose	5	5	-
NMDG	-	60	-
HEPES	10	10	10
EGTA	-	-	10
pH	7.4 with NaOH	7.4 with NaOH	7.4 with KOH
Osmolarity	~298 mOsm	~289 mOsm	~280 mOsm

The physiological solution and external solution were prepared at least one month. The intracellular solution was prepared in batches aliquoted and stored at 4°C until used. Test compounds were dissolved in 100% DMSO to obtain stock solutions for different test concentrations. Then the stock solutions were further diluted into external solution to achieve final concentrations for testing. Visual check for precipitation was conducted before testing. Final DMSO concentration in external solution was not more than 0.30% for the test compounds. Voltage command protocol: From this holding potential of -80 mV, the voltage was first stepped to -50 mV for 80 ms for leak subtraction, and then stepped to +20 mV for 4800 ms to open hERG channels. After that, the voltage was stepped back down to -50 mV for 5000 ms, causing a "rebound" or tail current, which was measured and collected for data analysis. Finally, the voltage was stepped back to the holding potential (-80 mV, 1000 ms). This voltage command protocol was repeated every 20000 msec. This command protocol was performed continuously during the test (vehicle control and test compound). hERG SyncroPatch assay was conducted at room temperature. The Setup, Prime Chip, Catch and Seal Cells, Amplifier Settings, Voltage and Application Protocols were

established with Biomek Software (Nanion). One addition of 40 µL of the vehicle was applied, followed by 300s for a baseline period. Then the doses of the compounds were added with 40 µL. The exposure of test compound at each concentration was no less than 300s. The recording for the whole process had to pass the quality control, or the well was abandoned and the compound was retested, all automatically set by PatchControl. Five concentrations (0.30 µM, 1.00 µM, 3.00 µM, 10.00 µM and 30.00 µM) were tested for each compound. A minimum 2 replicates per concentration were obtained. Data analysis was carried out using DataControl, Excel 2013 (Microsoft) and GraphPad Prism 5.0. Within each well recording, percent of control values were calculated for each test compound concentration current response based on peak current in presence of
reference control (current response/ peak current) ×100%. The Dose-Response curves were fit to the standard Hill equation as shown below: Ipost cpd/Ipre cpd=Bottom+(Top-Bottom)/(1+10^((LogIC_{50}-X)*HillSlope)) Where X is the logarithm of concentration, Ipost cpd/Ipre cpd is the normalized peak current amplitude, Top is 1 and Bottom is equal to 0. Curve-fitting and IC50 calculations were performed by GraphPad Prism 5.0. If the inhibition obtained at the lowest concentration tested was over 50%, or at the highest concentration tested was less than 50%, we reported the IC_{50} as less than lowest concentration, or higher than highest concentration, respectively.

In vivo pharmacokinetic studies
The animal studies were conducted in accordance with the testing facilities local IACUC guidelines that are in compliance with the Animal Welfare Act, the Guide for the Care and Use of Laboratory Animals.

The plasma pharmacokinetic properties of test compound (TC) were determined following intravenous (IV) bolus administration at a dose of 1 mg/kg in 20% wt/vol HPβCD, 1% vol/vol DMSO and PO administration at 3 mg/kg in 20% wt/vol HPβCD, 1% vol/vol DMSO, to male Sprague Dawley rats, or male beagle dogs, or cynomolgus monkeys (fed for IV, fasted for PO). Plasma samples were collected from 3 animals/group at 0.05, 0.25, 0.5, 1, 2, 3, 4, 8, and 24hafter dosing. Concentrations of TC in plasma samples were determined by a liquid chromatography tandem mass spectrometry (LC-MS/MS) method. The IV and PO PK curves for TNG908 in vivo studies in rat, dog, and monkey are shown in Figure SI-4.

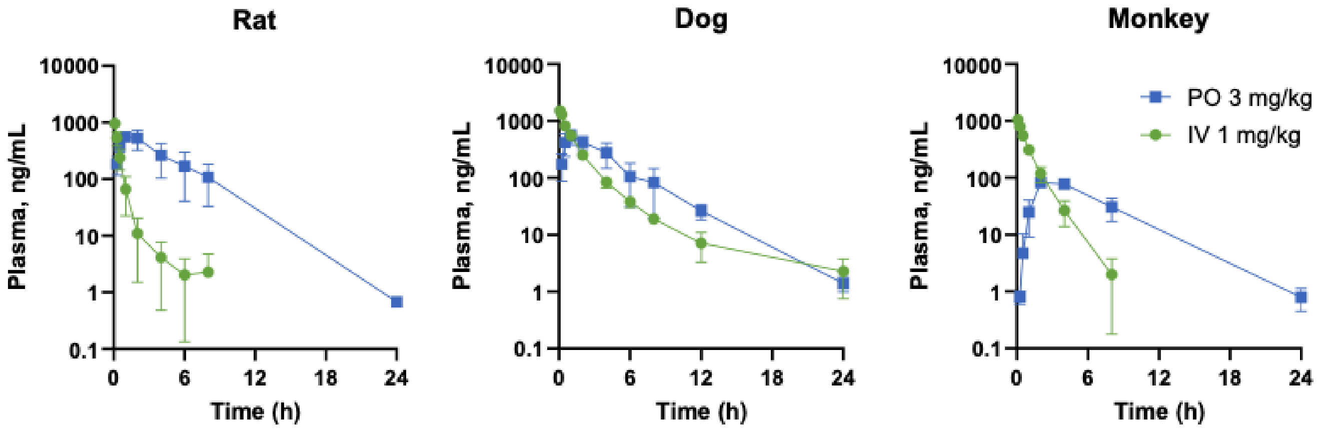

Figure SI-4: In vivo PK curves for TNG908 in rat, dog and monkey. TNG908 was dosed IV at 1 mg/kg and PO at 3 mg/kg.

CSF study in cynomolgus monkeys. Cynomolgus monkeys (N=3/sex) each with a subcutaneously implanted cannula in the cisterna magna were employed in this study. The

cerebrospinal fluid (CSF) samples were collected by inserting a syringe needle into the port at designated time points. Animals were administered 10 mg/kg TNG908 orally (dosing volume of 2 ml/kg; in 0.5% (w/v) methylcellulose (MC)) and serial samples of plasma and CSF were collected at 0.5, 1, 2, 3, 4, 8 and 24 hours post dose. The concentrations of **TNG908** in plasma and CSF were determined by using an LC MS/MS method. Brain penetration was estimated as the ratio of the area under the CSF concentration-time curve to the area under the plasma concentration-time curve (Table SI-14 and SI-15). The animal studies were conducted in accordance with the testing facilities local IACUC guidelines that are in compliance with the Animal Welfare Act, the Guide for the Care and Use of Laboratory Animals.

Table SI-14. Data for CSF non-human primate study.

Matrix	Plasma		CSF	
Dose Route	PO		PO	
Dose Level (mg/kg)	10		10	
PK Parameters	Mean	SD	Mean	SD
C_{max} (ng/mL)	2287	851	206	79.1
T_{max} (h)	3.33	1.15	4.67	3.06
$T_{1/2}$ (h)	2.53	0.477	4.34	ND
$AUC_{0\text{-}last}$ (ng•h/mL)	13919	5621	1649	681
$AUC_{0\text{-}inf}$ (ng•h/mL)	13976	5695	1313	ND
AUC Ratio [a]	--	--	0.118	0.00561

a: AUC Ratio = CSF $AUC_{0\text{-}last}$ / Plasma $AUC_{0\text{-}last}$.

"ND" means not determined.

"--" means not calculated.

Table SI-15. Individual and Mean Total Plasma and CSF Concentrations (ng/mL) of TNG908 in Male Cynomolgus Monkeys Following Single Oral Administration of TNG908 at 10 mg/kg

Time (h)	Mean Total Plasma Concentration (ng/mL)	SD	CV (%)	Mean CSF Concentration (ng/mL)	SD	CV (%)
0.500	114	91.9	80.3	11.0	ND	ND
1.00	1117	1557	139	61.8	80.6	131
2.00	1408	1501	107	121	133	110
3.00	1187	595	50.1	125	94.0	75.5
4.00	1703	552	32.4	167	45.4	27.3
8.00	1153	908	78.7	126	93.1	74.1
24.0	13.6	16.9	124	9.19	4.94	53.8

Kp,uu,CSF calculation[2]

AUC

$K_{p,uu,CSF} = AUC_{CSF}/AUC_{u,plasma}$

Plasma Protein Binding Procedure

The binding of TNG908 (2 uM) to cynomolgus monkey plasma protein was measured using the rapid equilibrium dialysis (RED) device per manufacturer's instruction (part number 90,007 (Thermo Fisher Scientific, USA). The incubation was carried out at 37°C for 4 hours. The TNG908 concentration in the donor and receiver chambers was determined by a fit-for-purpose LCMSMS method and the unbound free fraction of TNG908 was calculated as the ratio of receiver chamber over the donor chamber.
TNG908 monkey plasma protein binding – fu, plasma = 13.3%

SDMA quantification in PKPD studies

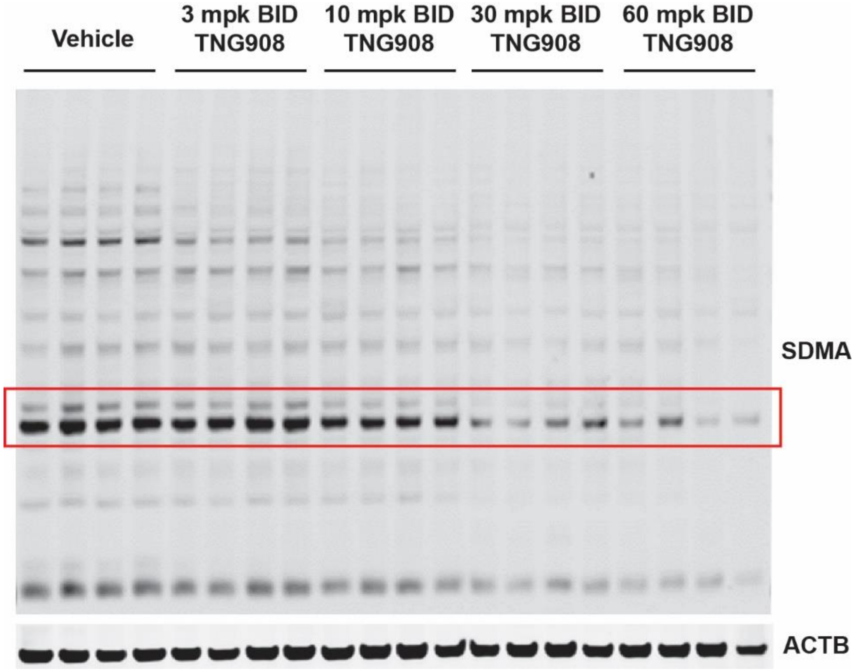

Figure SI-5: TNG908 treatment drives dose-dependent PD inhibition in the LN18 MTAP-null GBM xenograft model. Immunoblot analysis tumors treated as indicated for 10 days. The quantified protein species is outlined in red. It migrates at ~30-32 kDa, a molecular weight which is consistent with SNRPB/B'.

Accession codes
Structure factors and atomic coordinates for PRMT5:MEP50 + MTA (PDB 8VEO) and for PRMT5:MEP50 + MTA with compound 1 (PDB 8VET), compound 23 (PDB 8VEU), compound 24 (PDB 8VEW), compound 28 (PDB 8VEX), or TNG908 (PDB 8VEY) have been deposited in the Protein Data Bank (www.rcsb.org).

PRMT5:MEP50 expression and purification.
Recombinant protein production of the human PRMT5:MEP50 complex was performed in the Hi5 insect cell expression system using protein constructs that were described previously[3]. The Avi-tagged PRMT5:MEP50 complex was biotinylated enzymatically using BirA following partial purification by a nickel affinity column chromatography as reported[2]. The non-Avi-tagged PRMT5:MEP50 complex was purified similarly to past reports, but a final purification step using Superdex200 was added following purification on Ni-NTA and FLAG affinity columns. The final purification buffer consisted of 10 mM HEPES (pH 8.0), 150 mM sodium chloride, 10% glycerol, and 1 mM TCEP. Protein was concentrated to >15 mg/mL and flash-frozen before storage at -80 °C.

PRMT5:MEP50 crystallography.
Crystals of the MTA-bound PRMT5:MEP50 complex were routinely grown from 15-17 mg/mL protein samples containing 1 mM MTA via hanging drop vapor diffusion at 18 °C. Well solutions consisted of 10% (w/v) PEG 4000, 0.1 M sodium citrate (pH 6.0), and 0.2 M magnesium chloride. In some cases, 0.2 M sodium acetate was used instead of 0.2 M magnesium chloride. Once crystals of the MTA-bound PRMT5:MEP50 complex had grown, they were harvested and soaked with 1 mM inhibitor for 2 – 5 h at room temperature before being flash-cooled and stored for data collection. Cryo solutions consisted of the mother liquor supplemented with 30% ethylene glycol. In the case of compound 1, the MTA + inhibitor-bound complex was prepared by cocrystallization. PRMT5:MEP50 samples contained 1 mM MTA and 2 mM compound 1 and crystals were grown via vapor diffusion at 18 °C using a well solution consisting of 10% PEG 4000, 0.1 M MES (pH 6.5), and 0.2 M magnesium chloride. These crystals were transferred into a cryo solution consisting of mother liquor plus 20% glycerol prior to flash-cooling in liquid nitrogen.
Data collection occurred at multiple synchrotron sources as noted in Table SI-14. Data reduction and scaling were performed with XDS and AIMLESS[4,5] and structures were refined using REFMAC from the CCP4 software suite[6]. Model building was performed using COOT[7]. Initial phases were obtained from previously determined structures of PRMT5:MEP50. All final models exhibited good geometry. Final coordinates and structure factors are deposited in the PDB with the accession codes noted in Table SI-15.

Table SI-15. X-ray data collection and refinement statistics for PRMT5-MEP50 crystal structures.

Compound	-	1	23	24	28	TNG908
Cofactor	MTA	MTA	MTA	MTA	MTA	MTA
PDB ID	8VEO	8VET	8VEU	8VEW	8VEX	8VEY
Data Collection						
Synchrotron source	SSRF	APS	CLS	Spring8	SSRF	Diamond
Beamline	BL17U1	21-ID-D	08ID-1	BL45XU	BL18U	I03
Space group	I222	I222	I222	I222	I222	I222
Cell dimensions						
a, *b*, *c* (Å)	101.7,138.9,178.4	103.2,137.0,178.9	103.4,138.8,179.7	100.6,137.7,177.7	99.5,136.4,177.7	102.3,136.7,177.8
Resolution (Å)	54.79 - 2.03	44.69 – 2.63	46.77 – 2.46	45.65 – 2.69	45.19 – 2.79	60.23 – 2.44
Unique reflections	80230	37944	47213	34631	30399	46656
Redundancy	6.7 (6.4)	13.5 (13.0)	6.7 (6.7)	13.5 (13.1)	6.6 (6.6)	13.9 (13.9)
Completeness (%)†	98.5 (96.0)	99.8 (99.8)	99.9 (87.5)	100 (99.9)	99.9 (99.8)	99.9 (99.9)
R_{merge}†	0.045 (0.797)	0.088 (1.45)	0.078 (0.852)	0.150 (1.33)	0.081 (0.700)	0.085 (1.13)

I/σ(I)†	18.0 (2.80)	19.6 (1.9)	14.5 (2.2)	15.1 (2.4)	15.1 (2.5)	18.3 (2.3)
$CC_{1/2}$	0.999 (0.914)	0.999 (0.894)	0.999 (0.875)	0.999 (0.902)	0.999 (0.945)	0.999 (0.932)
Refinement						
Reflections used	79772	36007	44850	32905	28776	44386
R_{work}/R_{free}	0.227 / 0.279	0.224 / 0.277	0.201 / 0.250	0.220 / 0.269	0.226 / 0.270	0.223 / 0.261
Average B-value ($Å^2$)	68	82	68	70	89	72
Number of atoms						
Protein	7407	7399	7369	7346	7353	7352
Cofactor/Inhibitor	20	44	46	47	47	49
Solvent/Other	174	34	173	49	67	223
R.m.s. deviations						
Bond lengths (Å)	0.011	0.002	0.006	0.004	0.006	0.002
Bond angles (°)	1.04	1.31	1.48	1.39	1.44	1.18
PDB ID code						

† Values in parentheses are for the highest resolution shell.

Surface plasmon resonance studies.

As a hit triage step post-HTS, compounds were tested for binding to the PRMT5:MEP50 complex via surface plasmon resonance using a Biacore 8K equipped with a SA sensor chip (Cytiva). Biotinylated PRMT5:MEP50 diluted to 114 μg/mL in a buffer consisting of 20 mM HEPES (pH 8.0), 100 mM sodium chloride, 1 mM TCEP and 0.05% P20 was loaded onto a streptavidin-coated biosensor chip at a flow rate of 10 mL/min for a contact time of 600 s. Capture levels of 4000 – 4500 RUs were routinely achieved. Compound screening was performed at 25 °C using the same buffer as used for immobilization but with 2% DMSO and 50 μM MTA added. GSK591 was used as a control molecule to test the activity of surfaces before each run. Compound binding was tested in 10-point dose response using a top concentration of 50 μM and a flow rate of 80 μL/min, an association time of 90 s, and a dissociation time of 360 s. Top concentrations were adjusted as needed to accommodate tighter binding when observed.

REFERENCES

(1) Yung-Chi, C.; Prusoff, W. H. Relationship between the Inhibition Constant (KI) and the Concentration of Inhibitor Which Causes 50 per Cent Inhibition (I50) of an Enzymatic Reaction. Biochem. Pharmacol. 1973, 22 (23), 3099–3108. https://doi.org/10.1016/0006-2952(73)90196-2.

(2) Gupta, A.; Chatelain, P.; Massingham, R.; Jonsson, E. N.; Hammarlund-Udenaes, M. BRAIN DISTRIBUTION OF CETIRIZINE ENANTIOMERS: COMPARISON OF THREE DIFFERENT TISSUE-TO-PLASMA PARTITION COEFFICIENTS: Kp, Kp,u, AND Kp,Uu. Drug Metab. Dispos. 2006, 34 (2), 318–323. https://doi.org/10.1124/dmd.105.007211.

(3) Chan-Penebre, E.; Kuplast, K. G.; Majer, C. R.; Boriack-Sjodin, P. A.; Wigle, T. J.; Johnston, L. D.; Rioux, N.; Munchhof, M. J.; Jin, L.; Jacques, S. L.; West, K. A.; Lingaraj, T.; Stickland, K.; Ribich, S. A.; Raimondi, A.; Scott, M. P.; Waters, N. J.; Pollock, R. M.; Smith, J. J.; Barbash, O.; Pappalardi, M.; Ho, T. F.; Nurse, K.; Oza, K. P.; Gallagher, K. T.; Kruger, R.; Moyer, M. P.; Copeland, R. A.; Chesworth, R.; Duncan, K. W. A Selective Inhibitor of PRMT5 with in Vivo and in Vitro Potency in MCL Models. Nat. Chem. Biol. 2015, 11 (6), 432–437. https://doi.org/10.1038/nchembio.1810.

(4) Kabsch. XDS. Acta Crystallographica 2010, No. d66, 125–132. https://doi.org/10.1107/s0907444909047337.

(5) Evans, P. R.; Murshudov, G. N. How Good Are My Data and What Is the Resolution? Acta Crystallogr. Sect. D: Biol. Crystallogr. 2013, 69 (7), 1204–1214. https://doi.org/10.1107/s0907444913000061.

(6) Murshudov, G. N.; Vagin, A. A.; Dodson, E. J. Refinement of Macromolecular Structures by the Maximum-Likelihood Method. Acta Crystallogr. Sect. D: Biol. Crystallogr. 1997, 53 (3), 240–255. https://doi.org/10.1107/s0907444996012255.

(7) Emsley, P.; Cowtan, K. Coot: Model-Building Tools for Molecular Graphics. Acta Crystallogr. Sect. D: Biol. Crystallogr. 2004, 60 (12), 2126–2132. https://doi.org/10.1107/s0907444904019158.

AUTHOR INFORMATION

Corresponding Author

* **Kevin M. Cottrell** – *Tango Therapeutics, Boston, MA 02215, United States;* Phone: (+1) 857-320-4900; Email: kcottrell@tangotx.com

Authors

Kimberly J. Briggs – Tango Therapeutics, Boston, MA 02215, United States
Douglas A. Whittington – Tango Therapeutics, Boston, MA 02215, United States
Haris Jahic – Tango Therapeutics, Boston, MA 02215, United States
Janid A. Ali – Tango Therapeutics, Boston, MA 02215, United States
Charles B. Davis – Tango Therapeutics, Boston, MA 02215, United States
Shanzhong Gong – Tango Therapeutics, Boston, MA 02215, United States
Deepali Gotur – Tango Therapeutics, Boston, MA 02215, United States
Lina Gu – Tango Therapeutics, Boston, MA 02215, United States
Patrick McCarren – Tango Therapeutics, Boston, MA 02215, United States
Matthew R. Tonini – Tango Therapeutics, Boston, MA 02215, United States
Alice Tsai – Tango Therapeutics, Boston, MA 02215, United States
Erik W. Wilker – Tango Therapeutics, Boston, MA 02215, United States
Hongling Yuan – Tango Therapeutics, Boston, MA 02215, United States
Minjie Zhang – Tango Therapeutics, Boston, MA 02215, United States
Wenhai Zhang – Tango Therapeutics, Boston, MA 02215, United States
Alan Huang – Tango Therapeutics, Boston, MA 02215, United States
John P. Maxwell – Tango Therapeutics, Boston, MA 02215, United States

Author Contributions

The manuscript was written through contributions of all authors. All authors have given approval to the final version of the manuscript.

ACKNOWLEDGMENTS

We thank the following teams/people for their valuable contributions to this work: Oleg Michurin, Tanya Galushka and colleagues (Enamine, Kyiv, Ukraine). Chen Wei, Wan Shuangyi and colleagues (IDSU, WuXi AppTec, China). Shang Deju and colleagues (CSU, WuXi AppTec, Tianjin, China). Jian Shen and colleagues (Viva Biotech, Ltd., Shanghai, China) for X-ray crystallography work. Dennis Wegener and colleagues (Evotec AG, Hamburg, Germany) for SPR studies. Gang Chen, Xiaoyu Zhu, Kang Yan, and colleagues (WuXi AppTec, Shanghai, China) for peptide displacement and cellular assays. Yingying Ma, Tan Pang and colleagues (Pharmaron Inc, Beijing, China) for in vivo pharmacology studies. Ying Zhou and colleagues (WuXi AppTec, Shanghai, China) for in vitro ADME and PK studies. Yuzhou Xu and colleagues (ChemPartner, Shanghai, China) for biochemical characterization studies of TNG908. Scott Throner for assistance in editing of this manuscript. All research described in this manuscript was funded by Tango Therapeutics.

ABBREVIATIONS

PRMT5, protein arginine methyltransferase 5; MTAP, methylthioadenosine phosphorylase; MTA, methylthioadenosine; CNS, central nervous system; shRNA, short hairpin ribonucleic acid; TAMRA, tetramethylrhodamine; PSA, polar surface area; SAR, structure activity relationship; hERG, human Ether-a-go-go-Related Gene; MDCK, Madin-Darby Canine Kidney cell; Mdr1, multidrug resistance 1; IV, intravenous; PO, per os (oral); BID, bis in die (twice daily).
